# Linguistic Phylogenies Support Back-Migration from Beringia to Asia

**DOI:** 10.1371/journal.pone.0091722

**Published:** 2014-03-12

**Authors:** Mark A. Sicoli, Gary Holton

**Affiliations:** 1 Department of Linguistics, Georgetown University, Washington, District of Columbia, United States of America; 2 Alaska Native Language Center, University of Alaska Fairbanks, Fairbanks, Alaska, United States of America; University of Florence, Italy

## Abstract

Recent arguments connecting Na-Dene languages of North America with Yeniseian languages of Siberia have been used to assert proof for the origin of Native Americans in central or western Asia. We apply phylogenetic methods to test support for this hypothesis against an alternative hypothesis that Yeniseian represents a back-migration to Asia from a Beringian ancestral population. We coded a linguistic dataset of typological features and used neighbor-joining network algorithms and Bayesian model comparison based on Bayes factors to test the fit between the data and the linguistic phylogenies modeling two dispersal hypotheses. Our results support that a Dene-Yeniseian connection more likely represents radiation out of Beringia with back-migration into central Asia than a migration from central or western Asia to North America.

## Introduction

The aboriginal populations of America and Asia are linked through prehistoric migrations via the Bering Land Bridge. Our understanding of these migrations has been derived primarily from archaeological and biological data rather than from linguistics as most migrations preceded the generally accepted 8–10,000-year limit of the traditional comparative method of historical linguistics [1], [2]. DNA evidence supports at least three migrations with the earliest 15–40,000 BP referred to generically as the *Paleoindian* and associated with the greatest distribution of language and cultural groups across North, Meso, and South America; the second 12–14,000 BP is the *Na-Dene* distributed in North America from Alaska to the Pacific Northwest and from Canada to the U.S. Southwest; and the third ca. 9000 BP is *Eskimo-Aleut* with circumpolar distribution [3], [4]. Linguists have classified Eskimo-Aleut and Na-Dene as separate language stocks, and the rest of the languages of the Americas as belonging to numerous stocks, but have otherwise been mostly silent on questions that connect Asian and the American populations because, with the exception of Eskimo-Aleut, the dates of these earlier connections lie beyond the traditionally accepted limit for comparative reconstruction. Linguistic claims of more distant relationships have relied instead on the more controversial method of mass (or multilateral) comparison of lexical items subjectively judged as similar [5]. Using such methods a Dene-Yeniseian (DY) connection linking Asia to North America has been suggested for nearly 100 years [6], but only recently has a stronger case been made using methods of linguistic reconstruction [7], which has been peer reviewed with cautious optimism urging alternative methods for its evaluation [8], [9]. The hypothesis of a DY language family prompted claims of proof for the origin of Native Americans in central or western Asia [5], the relationship fitting into a popular narrative for the peopling of the Americas.

Our goal here is not to address the validity of the Dene-Yeniseian hypothesis nor the type of linguistic data used to support it. Rather, we address the questions of what it means for migration theories if the DY connection is true and how we can rigorously test hypotheses relating linguistic dispersals with population migrations. We show that Bayesian analysis and neighbor-joining network modeling applied to linguistic datasets provide new insight into the implications of the DY hypothesis. We use typological data to infer linguistic phylogenies that test two dispersal hypotheses. First, Ruhlen’s conjecture that “the origin of the Yeniseian-Na-Dene population can plausibly be traced to West Asia” [5], and second, that a relationship between Yeniseian and Na-Dene represents radiation out of Beringia. We use Bayesian model comparison based on Bayes factors [10] to test the fit between the linguistic phylogenies modeling the two dispersal hypotheses. Our results support an argument that, if the Dene-Yeniseian connection is true, it more likely reflects radiation out of Beringia with both eastward migrations into North America and westward migration into Asia rather than a unidirectional migration from Asia to North America.

## Materials and Methods

In the last decade, computational phylogenetic tools developed primarily in evolutionary biology have been incorporated into the field of historical linguistics bringing new methods to bear on questions of prehistoric migrations [11], [12], [13], language contact [14], language classification [15], [16], and language universals [17], [18], thereby potentially pushing the upper-limit of historical linguistic inference into the Terminal Pleistocene [19], [20], [21]. Greenhill and Gray [12] advocate the use of a phylogenetic framework to test how linguistic data match migration hypotheses, observing that without such rigorous testing migration scenarios “are little more than plausible narratives.” They argue for the use of Bayesian likelihood modeling over parsimony and use Austronesian lexical cognate sets to test between competing dispersal hypotheses for the Austronesian expansion throughout the Pacific. The use of lexical cognate data closely aligns with data used to infer family relationships in the traditional comparative method of historical linguistics, and the relatively shallow time depth of Austronesian expansion makes lexical cognate data appropriate for Greenhill and Gray’s study. However lexical cognates can be problematic due to a lack of lexical retention at deeper time depths and for families that have undergone extensive lexical borrowing. Wichmann and Saunders [22] review data and methods and propose that “[i]f one goal of linguistic phylogenetics is to infer more ancient relationships than those distinguishable by words alone, typological data may be the only choice.” Dunn and his collaborators [19], [20] pioneered the use of typological databases in modeling evolutionary history using parsimony methods to argue that a trace of phylogenetic signal is detectable from typological data of Papuan languages reflecting a time period in which Australia and New Guinea were joined by a land bridge in the late-Pleistocene continent Sahul. The use of typological data was motivated for Papuan because of the lack of retention of lexical cognates. In contrast, our motivation for using typological data in examining the prehistory of Na-Dene is an abundance of close cognates and inconsistency among isoglosses that have been argued to reflect a long history of lexical borrowing through language contact among related languages [23]. Our focus on typology specifically also takes up the challenge of using alternative methods to consider the position of Yeniseian within the proposed Dene-Yeniseian family which has been otherwise inferred primarily on the basis of lexicon and templatic morphology [7]. The abundance of cognates within Na-Dene presents a challenge when comparing the linguistics with the archaeology. Estimates of time-depth based on lexical comparison are less than 8500 years [24], but the archaeology of Alaska shows temporal horizons well beyond 10,000 years with striking technological continuities with the historically known Na-Dene populations [25].

We applied both Bayesian likelihood modeling and a neighbor joining distance method in evaluating typological features of DY, using a binary coding schema that indicates the presence or absence of phonological and morphological features. Unknown features for a taxon were coded with a question mark. Our data matrix consists of 116 characters for 40 taxa: 2 Yeniseian languages (Ket-Kott), 37 Na-Dene (Tlingit-Eyak-Athabascan) languages, and the isolate Haida included for its potential as an outgroup. The characters we coded for were based on categories represented in Joel Sherzer’s *An areal-typological study of American Indian languages north of Mexico* [26], with some expansion to include more contrasts between Yeniseian and Na-Dene. Na-Dene character values were first determined from the Sherzer monograph, then checked against other published and unpublished sources in the Alaska Native Language Archive and revised where more current data was available. Yeniseian language character values were determined from a published grammar for the extinct Kott [27], and published grammars for Ket [27], [28] with the Ket coding checked by a Yeniseian specialist. Uncertainty was coded with a question mark. Of the 116 characters, 26 were excluded as uninformative—either all lacking a feature or, to a lesser degree, all possessing a feature—leaving 90 informative characters. Supporting Information for this paper includes the list of features coded as characters (File S1) and the nexus file containing the data matrix (File S2). The neighbor joining analyses used the NeighborNet algorithm of SplitsTree4 [29], an agglomerative clustering algorithm that constructs a splits graph by iteratively combining taxa clusters given the character agreement and disagreement. The Bayesian analysis used the Markov Chain Monte Carlo (MCMC) method implemented in MrBayes [30]. We compared the models using multiple methods of harmonic mean estimation and marginal likelihood scores calculated by the stepping-stone method available through the MrBayes software from which Bayes factor values could be compared. We summarized the MCMC results of the most likely model through both a consensus tree and a consensus network.

## Results

**Figure 1:**
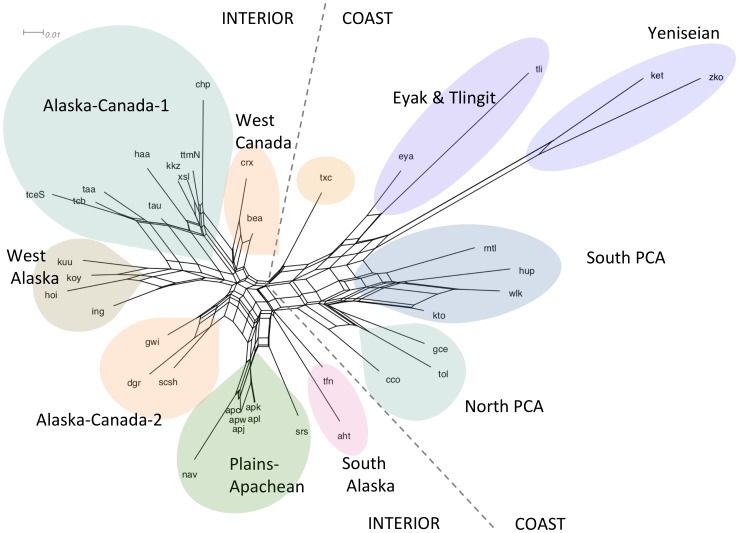
NeighborNet Splits Graph for Dene-Yeniseian Typological Features. The splits graph shows several clear clusters with rectilinear webbing within clusters showing regions of conflicting signals for specific taxa. Primary divisions in the splits graph are indicated with dashed lines separating primarily coastally distributed languages on the right with interior languages on the left. Colored shading highlights clusters. Within the coastal region of the network there are groupings for Pacific Coast Athabascan (PCA), Tlingit and Eyak, with Tlingit’s branch length long relative to Eyak. The Yeniseian languages Ket and Kott group tightly with each other on the right side of the network and show a long branch length indicating a high degree of differences from the others. In Interior we see several clusters: Plains-Apachean, including Sarsi (Tsuut’ina) in Canada; two groupings labeled Alaska-Canada-1 and Alaska-Canada-2 plus the smaller West Alaska and South Alaska groups. The clusters generally agree with established divisions between Na-Dene subfamilies and the rectilinear webbing is suggestive of the long history of language contact within Na-Dene. The average delta score is 0.367 and the average Q-residual score is 0.0492.

Fig. 1 is the NeighborNet splits graph which shows several clear clusters even though rectilinear webbing suggests regions of conflicting signals for specific taxa within clusters. We can see a primary division between groups which we label Coast languages on the right and Interior languages on the left. Within Coast there are clear groupings for North and South Pacific Coast Athabascan (PCA), Tlingit and Eyak, with Tlingit’s long branch length relative to Eyak’s shorter branch length supporting Eyak’s closer affiliation with Athabascan languages. The Yeniseian languages Ket and Kott group tightly with each other within the region of the network characterizing the Coast distribution and show a long branch length indicating a high degree of difference from the others. In Interior we see several clusters: Plains-Apachean, including Sarsi (Tsuut’ina) in Canada; two groupings labeled Alaska-Canada-1 and Alaska-Canada-2 plus the smaller West Alaska and South Alaska groups. The clusters generally agree with established divisions between Na-Dene subfamilies [31] and the rectilinear webbing is suggestive of the long history of language contact within Na-Dene, particularly within Northern Athabascan (Canada and Alaska) [31], [23].

We used the SplitsTree program to calculate the average delta score and Q-residual for the network as indicators to the extent of tree-likeness exhibited by the data. In general the closer to zero the scores the more a tree fits the data. The DY average delta score is 0.367 and the Q-residual is 0.0492. This is comparable to what Gray, Bryant and Greenhill [32] reported for Austronesian and Indo-European using typological data. They reported an average delta score for Austronesian typological data of 0.44 and average Q-residual of 0.05. Their figures for Indo-European typological data were 0.40 average delta score and 0.04 average Q-residual. Using the delta score the DY typological data appear slightly more tree-like than typological data for these other families, while Q-residual scores appear less tree-like than Indo-European but comparable to Austronesian. Taxon specific measures of tree-likeness give us a sense of how each language is contributing toward the rectilinear patterning in the network. The taxon specific delta and Q-residual scores are provided in Table 1 sorted on both delta and Q-residual scores, which, while showing some variation, are generally parallel. These scores show how the languages of the Plains-Apachean group, which form one of the clearest clusters in the splits graph, are in the most tree-like relation showing some of the lowest delta scores and Q-residuals by taxon. However, within this tree Navajo stands out as in a less clear tree-like relation by both measures, and Tsuut’ina (Sarsi) in Canada stands out by delta score but not Q-residual. The Yeniseian language Ket scores low or in the middle depending on the measure, while its sister Kott scores high by both measures. We in turn excluded each of these taxa to consider the position of each Yeniseian language separately from the other. When each Yeniseian language was excluded in turn, it slightly increased the scores for the remaining Yeniseian language and slightly affected its position in the network. When Kott was excluded, Ket sat between southern and northern PCA; while when Ket was excluded, Kott then sat between Eyak (eya) and Tsetsaut (txe). To achieve a more quantified analysis of these data, their clustering, and the uncertainty in the network, we used the same data matrix to apply a Bayesian phylogenetic method.

**Table 1:** Taxon-specific Delta Scores and Q-residuals for Yeniseian and Na-Dene languages sorted on delta score on the left and q-residual on the right.

Delta Score	Language/Delta	Language/Q-residual	Q-residual
0.320	Chiricahua Apache	Upper Tanana	0.0364
0.320	Western Apache	Jicarila	0.0366
0.324	Kiowa Apache	Chiricahua Apache	0.0368
0.324	Lipan Apache	Western Apache	0.0368
0.324	Jicarila	Beaver	0.0369
0.329	Ket	Tsuut'ina	0.0382
0.334	Hupa	Dena'ina	0.0385
0.337	Wailaki	Kiowa Apache	0.0392
0.339	N Tutchone	Lipan Apache	0.0392
0.345	Slave	Ahtna	0.0395
0.346	Tanana	Tsetsaut	0.0402
0.347	Mattole	Mattole	0.0424
0.352	Upper Tanana	Eyak	0.0428
0.359	Kaska	Tanana	0.0435
0.360	Eyak	Hare	0.0440
0.361	Tlingit	Gwich'in	0.0445
0.362	Navajo	Slave	0.0447
0.364	Han	N Tutchone	0.0450
0.364	Kato	Kaska	0.0450
0.364	Tanacross	Han	0.0475
0.368	Tolowa	Ket	0.0481
0.371	Koyukon	Carrier	0.0489
0.373	Hare	Tolowa	0.0496
0.373	S Tutchone	Navajo	0.0497
0.374	Tsuut'ina	Galice	0.0506
0.383	Holikachuk	Hupa	0.0510
0.385	Galice	Kato	0.0526
0.387	Carrier	Koyukon	0.0536
0.387	Ahtna	Tlingit	0.0555
0.388	Gwich'in	Dogrib	0.0556
0.390	Dene Sułine	Tanacross	0.0562
0.395	Dogrib	Dene Sułine	0.0583
0.395	Beaver	Upper Kuskokwim	0.0609
0.398	Tsetsaut	Deg Xinag	0.0609
0.399	Dena'ina	Wailaki	0.0618
0.402	Upper Kuskokwim	Holikachuk	0.0625
0.413	Kott	Chasta Costa	0.0652
0.422	Deg Xinag	Kott	0.0807
0.424	Chasta Costa	S Tutchone	0.0828

Higher numbers indicate less tree-like relationships.

Implementing our analysis in MrBayes we used a reversible substitution model with gamma distributed rate variability. We did several Markov chain runs modeling different priors and used multiple likelihood measures to evaluate the models representing the different hypotheses. The likelihood measures used were based on the stepping-stone method to infer marginal likelihoods to calculate the Bayes Factor. We also compared the harmonic mean provided in the summary of the parameters by MrBayes though this is known to be less reliable [33]. In our first set of runs we used a non-clock model, producing an unrooted tree. We included the unrelated linguistic isolate Haida in the dataset, which consistently was inferred to be outside of DY in the trees. We subsequently set a prior constraint to take Haida as the outgroup to root the tree and run clock models. To select between strict or relaxed clock models, we did MCMC runs specifying either a strict clock (uniform) model and a relaxed clock (TK02 continuous autocorrelated) model [34] and then evaluated the two models comparing their harmonic means and marginal likelihoods. The Bayes factor based on comparison of marginal likelihoods failed to distinguish the models at about 1 log unit, but the strict clock model showed a harmonic mean 5 log units above the relaxed clock models, well beyond the threshold of 3 log units suggested by Kass and Raftery [10], providing substantial evidence in favor of the strict clock model. We thus chose to use a strict clock model consistently for subsequent runs in which we varied taxonomic constraints that would test between dispersal hypotheses.

We ran the MCMC algorithm for 2,000,000 generations sampling every 500 generations to generate 4,001 trees and used a burn-in of 25% to sample 3,001 trees which was adequate for convergence and long enough for representative independent samples of the tree space, as verified by the Tracer algorithm of the BEAST software package [35]. We expect the two different migration hypotheses to exhibit different tree topologies. The out of central/western Asia hypothesis assumes that the Yeniseian languages (and potentially their extinct relatives) branched off of the Dene-Yeniseian family with Na-Dene subsequently diversifying. The tree topology for this hypothesis would place the Yeniseian languages outside of Na-Dene: [Yeniseian[Na-Dene]. The radiation out of Beringia hypothesis does not assume that Yeniseian necessarily branched first. To compare these two hypotheses we did separate MCMC runs where in one we imposed a prior taxonomic constraint that grouped the Na-Dene languages as an ingroup excluding Yeniseian. This constraint had the effect of creating a Na-Dene clade in 100% of the trees joining Yeniseian to the tree outside Na-Dene. We then calculated marginal likelihoods and the harmonic means to select the better model based on Bayes factors, effectively judging whether this topology was a better fit for the data than a MCMC run that did not include this constraint. The stepping-stone method was applied three times for each model with the marginal likelihoods of the three runs averaged.

**Figure 2:**
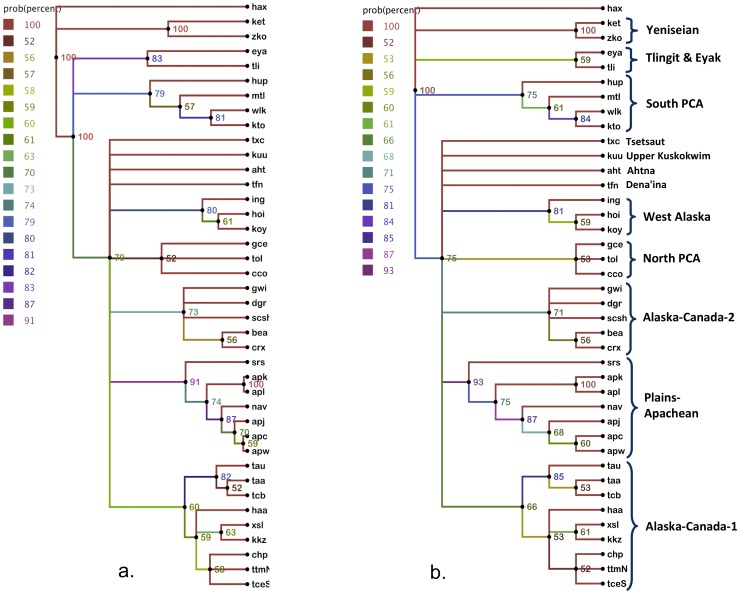
Consensus Tree Summaries of MCMC Runs. Splits in these trees occurred in greater than 50% of 3001 trees sampled. Numbers at nodes and line shading indicate clade credibility in percentages. Bracketing and labels highlight groupings. The unrelated isolate Haida is included as an outgroup to root the tree. Tree (a) on the left was produced under a taxonomic constraint in which Yeniseian Split off before the diversification of Na-Dene. It was a substantially weaker hypothesis than tree (b) on the right in which there is no hierarchical relationship between Yeniseian, Tlingit and South PCA. In comparison with tree (a), tree (b) had substantial support with a Bayes factor 8.5 log units greater.

The result showed that the topology that modeled the out-of-central Asia hypotheses did not explain the data better. In fact the model without this constraint showed an average marginal likelihood over 8.5 log units higher than the model with the constraint, providing strong support for the radiation out-of-Beringia hypothesis. Comparison of the harmonic means between the runs was less conclusive at less than 2 log units but in the same direction. The Bayes factors indicate that a model placing Yeniseian outside a Na-Dene clade fits the data significantly worse than the model without this constraint. The two consensus trees resulting from these models are provided in Fig. 2. In tree (a) Na-Dene is constrained as an ingroup, while tree (b) does not use the constraint. These are majority rules consensus trees that include only clades with support in greater than 50% of the trees. The tree in (b) is much better supported than the tree in (a) and is also in general agreement with the groupings highlighted in Fig. 1. In this tree Yeniseian, Tlingit, Eyak and South PCA are at the same phylogenetic level without being in a hierarchical relationship with each other. The terminal output of these Bayes runs is included in File S3.

To overcome any lingering doubts about the effect of including Haida on the results, we also conducted MCMC runs removing Haida from the analysis to just leave the Yeniseian and Na-Dene languages at issue in the hypotheses we are testing. We continued to use a strict clock and did two runs testing the presence or absence of the same taxonomic constraint that joined Yeniseian outside of Na-Dene. Again the constrained tree was not better than the tree without this constraint. The harmonic means were less than 1 log unit apart not supporting either model. However, the difference between marginal likelihoods generated through the more reliable stepping-stone method was 9.7 log units higher for the topology without the taxonomic constraint. The Bayes factor clearly does not support the hypothesis that Yeniseian split off before the diversification of Na-Dene, which speaks against Ruhlen’s conjecture [5] that Yeniseian represented an early separation away from what came to diversify as Na-Dene. We also conducted several MCMC runs excluding additional taxa to explore the data further. We excluded the Yeniseian languages in turn with similar results to the NeighborNet exclusions discussed above. When Kott was excluded, Ket sat in a clade with Southern PCA; while when Ket was excluded, Kott sat at the highest branching level where Yeniseian is in Fig. 2b. We also conducted a run with Eyak excluded, which did not change the position of Tlingit. The terminal output from these runs is included in File S4.

### Consensus Network

**Figure 3:**
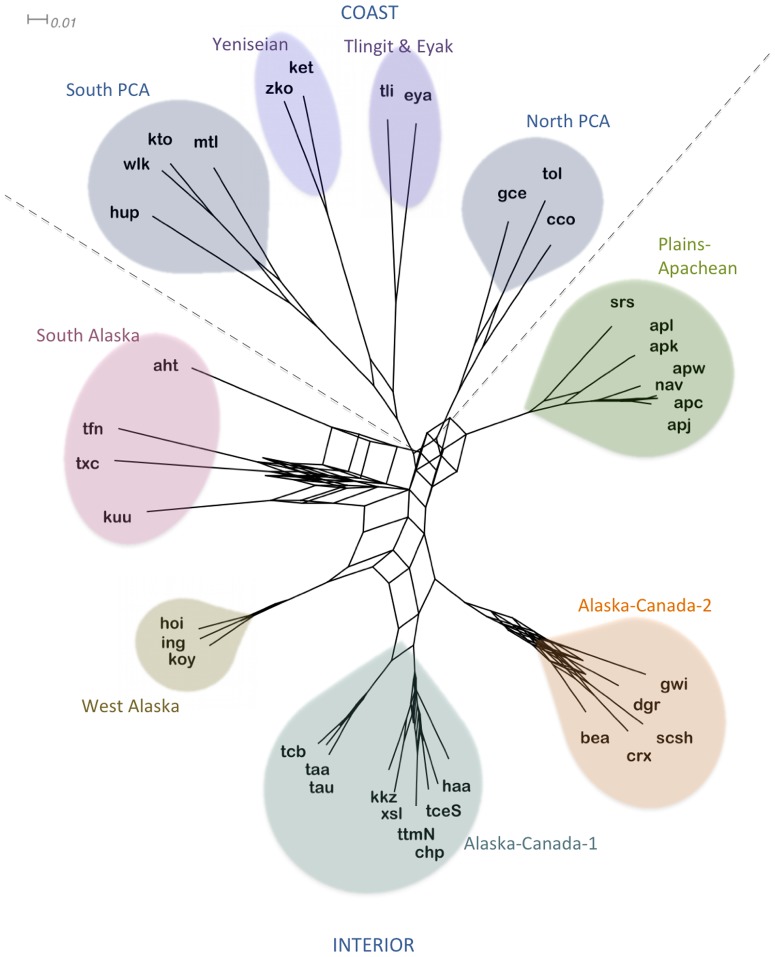
Consensus Network Summary of MCMC Run. Network summarizes all splits with at least 10% support in 3001 trees sampled. Longer branch lengths indicate higher probabilities for splits. Rectilinear webbing indicates lower frequency splits. Primary divisions in the network are indicated with dashed lines separating Coast languages in the upper portion and Interior languages in the lower portion. Colored shading highlights cluster groupings.

**Figure 4:**
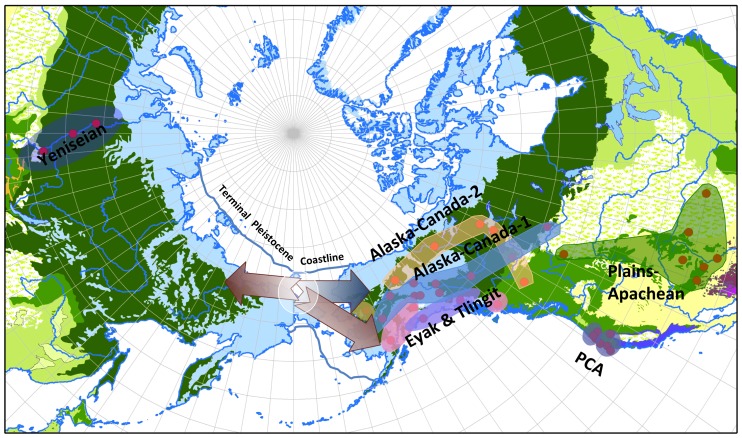
Dene-Yeniseian Out-of-Beringia. This polar projection map of Asia and North America shows the approximate terminal Pleistocene shoreline. The center of geographic distribution of Yeniseian and Na-Dene language is in Beringia. From this center burgundy arrows extend toward the North American coast and into Siberia. A blue arrow indicates Interior dispersals of Na-Dene.

A consensus tree is one way to summarize a Bayes run but can be problematic in that it leaves out information from trees with less than 50% support, effectively hiding them from the consensus visualization [20]. For this reason we constructed a consensus network which allows for better visualization of the extent of support for alternative dispersal scenarios which place Yeniseian elsewhere in the phylogeny. Consensus networks are better representations of samples of trees because they are able to visualize conflicting evolutionary hypotheses by representing each split by parallel edges proportional in length to the probability assigned to the split [36]. Like the NeighborNet, the consensus network in Fig. 3 shows a major split between Coast and Interior languages. The Yeniseian languages lie within the Coast region of the network with no webbing showing evolutionary scenarios that link to the Interior languages or placing Yeniseian outside of Tlingit in the phylogenies. The clusters have been shaded in the same colors used in the NeighborNet and are plotted on the map in Fig. 4.

## Discussion

Regardless of the ultimate fate of the DY hypothesis, our work demonstrates the utility of using computational phylogenetic tools to explore the implications of proposals for deep linguistic relationships. While the focus of attention on the DY hypothesis has centered on the potential existence of a linguistic connection between Asia and America, the work described here focuses instead on the implications of such a connection for human migration. Those implications can in turn be compared with evidence from the complementary fields of archaeology and biology.

Should the DY hypothesis hold true, our application of computational phylogenetic methods supports an Out-of-Beringia population dispersal (Fig. 4) rather than the Out-of-Central/Western-Asia dispersal proposed by Ruhlen [5]. Bayesian comparison of models using Bayes factors based on marginal likelihood calculations provides no support for the Out-of-Central/Western-Asia hypotheses modeled by a taxonomic constraint that places Yeniseian as diverging early from a Na-Dene clade. Rather, the phylogeny with the strongest Bayes factor supports an early radiation from the center of the geographical distribution of the language family [37] in Beringia with migrations dispersing populations both along the North American Coast and back into Siberia, and subsequently population chains into the North American interior (Fig. 4). While we propose the first linguistically grounded argument for radiation out of Beringia, Tamm et al. [38] have proposed a strikingly parallel set of claims using mtDNA markers to argue for a “Beringian Standstill” before both a rapid early coastal migration into North America and back-migrations from Beringia into Asia. Here we have from linguistic data independent of archaeology or biology contributed to a theory of population dispersal that, while not contradicting the popular narrative of pedestrian hunters entering the New World through Beringia, complicates it with the insight that this was not a one-way trip.

There are several clear directions for future work. First, it would be desirable to expand on the typological data set by adding more characters. The findings we have discussed here are based on less than 100 informative characters, and we expect that additional data would make model comparison more robust. Such an expansion is challenging, because many of the Na-Dene and Yeniseian languages are extinct or endangered, which makes it difficult or impossible to expand the dataset evenly. Moreover, the radically templatic character of Na-Dene morphology complicates typological categorization. Another potential for further research is to bring lexical data in where possible. Using a small number of lexical characters Wichmann et al. [39] report more tree-like delta scores for Na-Dene and Yeniseian separately than we find for the combined DY network based on typological characters. This suggests that lexical characters may provide additional insights into the structure of the DY network. We are currently building a lexical dataset as well and plan to create a partitioned data matrix that could model both lexical and typological data together. Currently though we do not have lexical data for as many languages as we have typological data. Finally, there are implications for future work beyond the question of the DY connection. Our modeling has also generated several hypotheses regarding the dispersal of Na-Dene speakers across Coastal and Interior North America developing inquiry in historical linguistics with new methodologies that contribute a uniquely linguistic perspective on questions of prehistory.

## Supporting Information

File S1 **Sicoli-Holton-DYCharacters-Taxa Information.**
(PDF)

File S2 **Sicoli-Holton-DY-Typological.nex.**
(NEX)

File S3 **MCMC Runs for** **Fig. 2** **(with Haida Outgroup).**
(PDF)

File S4 **MCMC Runs (without Haida Outgroup in matrix).**
(PDF)
