# Supplementary material for: Linguistic Phylogenies Support Back-Migration from Beringia to Asia
Source: PLoS One. 2014 Mar 12;9(3):e91722. doi: 10.1371/journal.pone.0091722 (PMC3951421; doi:10.1371/journal.pone.0091722)
Supplement: File S1 — Sicoli-Holton-DYCharacters-Taxa Information. (PDF) [file pone.0091722.s001.pdf]

Mark A. Sicoli & Gary Holton
Dene-Yeniseian Typological Characters

This 116 character set is modified from features described in Sherzer (1976) An areal-typological study of American Indian languages north of Mexico. Amsterdam: North Holland Publishing Company.

PHONOLOGY

VOWELS
1. Three Vowel
2. 1-1-1
3. 2-1
4. Four Vowel
5. 2-2
6. 2-1-1
7. 1-2-1
8. Five Vowel
9. 3-2
10. 3-1-1
11. 2-2-1
12. Six Vowel
13. 2-2-2
14. 2-3-1
15. 3-2-1
16. Seven Vowel
17. 2-2-2-1
18. 3-3-1
19. Voiceless Vowel
20. Nasal Vowel
21. Pitch Phonemic
22. not a,e,i,o,u
23. vowel length contrast
24. mid or mid-high vowel

STOPS
25. one stop series: voiceless
26. two stop series: voiceless/voiced
27. two stop series: voiceless/glotalized
28. three stop series: voiceless/voiced/glottalized
29. four stop series
30. glottalized stop series
31. labial stop present
32. labial order (not nasals)
33. dental order
34. k order
35. t̤ (retroflex order)
36. c/tʃ
37. k/č
38. k/q
39. either k/č or k/q
40. tθ
41. q
42. kw
43. qw

FRICATIVES
44. one fricative series: voiceless
45. two fricative series: voiceless/voiced
46. two fricative series: voiceless/glottalized
47. three fricative series: voiceless/voiced/glottalized
48. glottalized fricatives
49. pharyngeal fricatives
50. labial fricative
51. θ
52. ð
53. s/ʃ
54. z
55. x
56. xw
57. x.
58. x.w
59. ɣ
60. ɣw
61. h
62. hw

LATERALS
63. l
64. ł
65. tł
66. tł'
67. dl
68. l'
69. ł'
70. ly
71. ły

NASALS
72. voiceless nasal
73. glottalized nasal
74. ɲ
75. ŋ

RHOTICS
76. r
77. voiceless r
78. glottalized r
79. r/l

SEMIVOWELS
80. voiceless semivowel
81. glottalized semivowel

MORPHOLOGY

NOMINAL MORPHOLOGY

82. nominal case
83. possessive pronoun prefix
84. possessive pronoun suffix
85. possessive pronouns independent morpheme
86. alienable/inalienable?
87. reduplication = distributive or plural
88. reduplication = diminutive
89. augmentative-diminutive consonant symbolism
90. masculine/feminine gender
91. animate/inaminate gender
92. plural in pronouns
93. plural in nouns
94. inclusive/exclusive plural in pronouns
95. dual in pronouns
96. dual in nouns
97. inclusive/exclusive dual in pronouns
98. demonstratives for visible/invisible objects
99. numerals classified by form or shape of object
100. locative prefixes
101. locative suffixes
102. locative prepositions
103. locative postpositions

VERBAL MORPHOLOGY

104. nominal incorporation
105. subject person marker prefixes
106. subject person marker suffixes
107. subject person markers independent pronouns
108. reduplication in verb = distribution, repetition
109. reduplication in verb = diminutive
110. prefixes mark tense-aspect
111. suffixes mark tense-aspect
112. evidential or source of information marked
113. instrumental markers
114. locative-directional markers
115. locative-directional markers prefix
116. locative-directional markers suffix

Dene-Yeniseian Taxa

	LAT	LONG	CODE	NAME
1.	53	-132	hax	Haida
2.	66.5647	-145.2739	gwi	Gwich'in
3.	62.7672	-115.868	dgr	Dogrib
4.	66.2569	-128.633	scsh	Hare
5.	60.8171	-115.784	xsl	Slave
6.	56.9499	-119.515	bea	Beaver
7.	53.9171	-122.75	crx	Carrier
8.	54.4641	-110.183	chp	Dene Sułine
9.	54.9944	-129.954	txc	Tsetsaut
10.	64.7881	-141.2	haa	Han
11.	62.6822	-159.5619	ing	Deg Xinag
12.	63.0133	-154.375	kuu	Upper Kuskokwim
13.	62.9036	-160.0647	hoi	Holikachuk
14.	64.8809	-157.701	koy	Koyukon
15.	65.1533	-149.3369	taa	Tanana
16.	62.2714	-145.3822	aht	Ahtna
17.	59.9719	-154.8478	tfn	Dena'ina
18.	60.0629	-128.711	kkz	Kaska
19.	63.3853	-143.3464	tcb	Tanacross
20.	62.9822	-141.9517	tau	Upper Tanana
21.	62.8303	-136.582	ttm	N Tutchone
22.	61.6097	-137.499	tce	S Tutchone
23.	60.3	-145	eya	Eyak
24.	59	-135	tli	Tlingit
25.	42.25	-123.16	gce	Galice
26.	41.930156	-124.141745	tol	Tolowa
27.	42.4	-124	cco	Chasta Costa
28.	41.05	-124.4	hup	Hupa
29.	40.1	-124.1	mtl	Mattole
30.	40.64	-122.307	wlk	Wailaki
31.	39.4	-124.4	kto	Kato
32.	33.15	-108	apc	Chiricahua Apache
33.	33.45	-110	apw	Western Apache
34.	36.35	-104	apj	Jicarila
35.	36.1	-108	nav	Navajo
36.	35.07	-98.246	apk	Kiowa Apache
37.	31.79	-106.42	apl	Lipan Apache
38.	51.045	-114.0572	srs	Tsuuti'ina
39.	64	87	ket	Ket
40.	57	94	zko	Kott

MATRIX

```
                   10        20        30        40        50        60        70        80        90        100       110
          .    |    .    |    .    |    .    |    .    |    .    |    .    |    .    |    .    |    .    |    .    |    .
hax   1010000000000000000000?000010111010l???0100100001000001111001011111100001010000000100100000100000?0001010?000111101
gwi   0000001100100000000111?1000101?01101???0010010000000111000101011111000000001001000100100000l?0000000100?1010011?01??
dgr   0001001000000000000110?1000101?01101???0010010000100111000101011111000000001001100100100000l?0100000100110100111011?
scsh  0000000000100000000110?1000101?00101???0000010000100111000101011111000000001001010100100000l?0?00000100?1010011?01??
xsl   0000000000100000000110?1000101?01101???1000010000011111000101011111000000101001000100100000l?0?00000100?1010011?01??
bea   0000000000100000000111?1000101?01101???0000010000000111000101011111000000001000000010010000011 0?00000100?10100111011l
crx   0000000000100000000111?1000101?00101???0000010000000111000101011111000000111001000100100000110100000100110100111?011?
chp   0000000000001000000111?1000101?01101???1101010000011111111101111110000000010010001001000001?0100000100110100111?011?
txc   0000000000000001100001?1000101?10101???0010010000100111000101011111000000000000000100100000l?0?00000100?1010011?01??
haa   0000000000000001100111?1000101?01111???1000010000011111000101011111000000001001100100100000l?0?00000100?1010011?01??
ing   0000000100100000000001?1000101?01111???1100010000011111000101011111000010010000000100100000l?0?00000100?1010011?01??
kuu   0000000000001010000001??000101?00111???0100010000000111000101011111000010000000000100???????????????????101?01??????
hoi   0000000000001010000001??000101?01101???1000010000011111010101011111000010010000000100???????????????????101?01??????
koy   0000000000001010000001?1000101?00101???1100010000011011000101011111000010000000000100100000l?0000000100?1010011?01??
taa   0000000000001001000011?1000101?01111???1000010000011111000101011111000010000000100100100000l00000000100110100111?01??
aht   0000000110000000000100?0000101?11101???0100010000000011000101111111000000010000000100100000l?0100000100?1010011?01??
tfn   0001001000000000000001?1000101?01101???0100010000000011010101011111000000000000000100100000l?0?00000100?1010011?01??
kkz   0001010000000000000110?1000101?01101???1000010000011111000101011111000000001001000100100000l?0?00000100?1010011?01??
tcb   0000000000001010000111?1000101?01101???1000010000011111000101011111000000000000100100l???????????????????1?????????
tau   0000000000001100000111?1000101?01101???1000010000011111000101011111000000000000000100100000l?0?00000100?1010011?01??
ttmN  0000000100100000000111?1000101?01101???1000010000011111000111011111000000001001000100100000l?0?00000100?1010011?01??
tceS  0000000000001010000111?1000101?01101???1000010000011111000111011111000000001001001????????????????????????1?????????
eya   0000000000000001100101?1000101?01101???0100??00000001010110010111110000000000000000100100000?00000??0001010000011?0???
tli   0000000000000001100011?1000101?01101???0111001010000101111001101111010000000000000000100100000100000110100110000011?1110
gce   0001001000000000000101?1000101?01101???0?100100000000110000001011010000000000000001001001000001000000??0100010000010?0110
tol   0000000100100000000101?1000101?01101???0?110100000000101100101011000000000000000000100100000?00000??0100010000010?0110
cco   0000000000001010000001?1000101?01101???0101010000010111100101011110000000000000000100100000?00000??0100010000010?0110
hup   0000010000000000000000?0000101?01101???0100100000000101100001011110000000001000000010010010011000011010001000011l0110
mtl   0000000000100000000001?1000101?01101???0010100000000101100101011010000000001000000010010000011010000000100100001l?0110
wlk   0000000000100000000000?0000101?01101???0100100000000100000100011000000000001000000010010000011000000000?00010000011?0110
kto   0000000000100000000000?0000101?01101???0110010000000110000101011010000000001000000010010000011000000001000100001l10110
apc   0000001000000000000110?0000101?01101???0000010000000111000101011111000000000000000100100000000100000100010000101 0110
apw   0000001000000000000110?0000101?01101???0000010000000111000101011111000000000000000100100000000100000100010000101 0110
apj   0000001000000000000110?0000101?00101???0000010000000111000101011111000000000000000100100000000100000100010000101 0110
nav   0000001000000000000110?0000101?01101???0010010000000111100111111111000000000000000101001000000000100000100010000101 0110
apk   0001001000000000000110?0000101?00101???0000010000000111000101011111000000000000000100100000100100000100010000111 0110
apl   0001001000000000000110?0000101?00101???0000010000000111000101011111000000000000000100100000100100000100010000111 0110
srs   0001100000000000000010?0000101?01101???0000010000000111000101011111000000000000000100100000100000000000001000010?0?00
ket   000000000000000101001111010000111100011010010000000001000000010010000000000100000001000000111100000000101110000110000 0
zko   000000000000000100001101000000011110111101001000000100101010001010000000100011100100011000001111000000001011111001000000
```
