# Supplementary material for: Linguistic Phylogenies Support Back-Migration from Beringia to Asia
Source: PLoS One. 2014 Mar 12;9(3):e91722. doi: 10.1371/journal.pone.0091722 (PMC3951421; doi:10.1371/journal.pone.0091722)
Supplement: File S3 — MCMC Runs for Fig. 2 (with Haida Outgroup). (PDF) [file pone.0091722.s003.pdf]

Sicoli-Holton: Linguistic phylogenies support back-migration from Beringia to Asia

MCMC Runs for Fig. 2 (with Haida Outgroup)

(2a) With Na-Dene Ingroup Constraint	2
(2b) Without Na-Dene Ingroup Constraint	40

```
                            MrBayes v3.2.1 x64

                      (Bayesian Analysis of Phylogeny)

              Distributed under the GNU General Public License


               Type "help" or "help <command>" for information
                     on the commands that are available.

                   Type "about" for authorship and general
                       information about the program.


MrBayes > exe /Users/msicoli/DY-Typlogical_binary_bayes-2.nex

   Executing file "/Users/msicoli/DY-Typlogical_binary_bayes-2.nex"
   UNIX line termination
   Longest line length = 123
   Parsing file
   Expecting NEXUS formatted file
   Reading data block
      Allocated taxon set
      Allocated matrix
      Defining new matrix with 40 taxa and 116 characters
      Data is Standard
      Missing data coded as ?
      Gaps coded as -
      Data matrix is not interleaved
      Taxon  1 -> hax
      Taxon  2 -> gwi
      Taxon  3 -> dgr
      Taxon  4 -> scsh
      Taxon  5 -> xsl
      Taxon  6 -> bea
      Taxon  7 -> crx
      Taxon  8 -> chp
      Taxon  9 -> txc
      Taxon 10 -> haa
      Taxon 11 -> ing
      Taxon 12 -> kuu
      Taxon 13 -> hoi
      Taxon 14 -> koy
      Taxon 15 -> taa
      Taxon 16 -> aht
      Taxon 17 -> tfn
      Taxon 18 -> kkz
      Taxon 19 -> tcb
      Taxon 20 -> tau
      Taxon 21 -> ttmN
      Taxon 22 -> tceS
      Taxon 23 -> eya
      Taxon 24 -> tli
      Taxon 25 -> gce
      Taxon 26 -> tol
      Taxon 27 -> cco
```

```
      Taxon 28 -> hup
      Taxon 29 -> mtl
      Taxon 30 -> wlk
      Taxon 31 -> kto
      Taxon 32 -> apc
      Taxon 33 -> apw
      Taxon 34 -> apj
      Taxon 35 -> nav
      Taxon 36 -> apk
      Taxon 37 -> apl
      Taxon 38 -> srs
      Taxon 39 -> ket
      Taxon 40 -> zko
      Successfully read matrix
      Setting default partition (does not divide up characters)
      Setting model defaults
      Seed (for generating default start values) = 1388071270
      Adding dummy characters (unobserved site patterns) for division 1
      WARNING: There are 26 characters incompatible with the specified
               coding bias. These characters will be excluded.
      Setting output file names to "/Users/msicoli/DY-Typlogical_binary_bayes-2.nex.run<i>.<p
t>"
   Exiting data block
   Reached end of file

MrBayes > lset nst=6 rates=gamma

   Setting Rates to Gamma
   Successfully set likelihood model parameters
   Adding dummy characters (unobserved site patterns) for division 1
   WARNING: There are 26 characters incompatible with the specified
            coding bias. These characters will be excluded.

MrBayes > prset brlenspr=clock:uniform

   Setting Brlenspr to Clock:Uniform
   Successfully set prior model parameters
   Adding dummy characters (unobserved site patterns) for division 1
   WARNING: There are 26 characters incompatible with the specified
            coding bias. These characters will be excluded.

MrBayes > outgroup 1

   Setting outgroup to taxon "hax"

MrBayes > constraint ingroup = 2-38

   Defining constraint called 'ingroup'

MrBayes > prset topologypr = constraints(ingroup)

   Setting Topologypr to Constraints
   Successfully set prior model parameters
   Adding dummy characters (unobserved site patterns) for division 1
   WARNING: There are 26 characters incompatible with the specified
            coding bias. These characters will be excluded.

MrBayes > mcmcp ngen=2000000 printfreq=10000 samplefreq=500 nruns=1 nchains=4 savebrlens=yes
```

```
filename=DY-26Dec-strict-Hout-Ynotin

   Setting number of generations to 2000000
   Setting print frequency to 10000
   Setting sample frequency to 500
   Setting number of runs to 1
   Setting number of chains to 4
   Setting chain output file names to "DY-26Dec-strict-Hout-Ynotin.<p/t>"
   Successfully set chain parameters

MrBayes > mcmc

   Running Markov chain
   MCMC stamp = 5611881771
   Seed = 1248079053
   Swapseed = 1388071270
   Model settings:

      Data not partitioned --
         Datatype  = Standard
         Coding    = Variable
         # States  = Variable, up to 10
                     State frequencies are fixed to be equal
         Rates     = Gamma
                     Gamma shape parameter is uniformly dist-
                     ributed on the interval (0.00,200.00).
                     Gamma distribution is approximated using 4 categories.
                     Likelihood summarized over all rate categories in each generation.

   Active parameters:

      Parameters
      ------------------
      Statefreq        1
      Shape            2
      Ratemultiplier   3
      Topology         4
      Brlens           5
      Clockrate        6
      ------------------

      1 --  Parameter  = Alpha_symdir
            Type       = Symmetric diricihlet/beta distribution alpha_i parameter
            Prior      = Symmetric dirichlet with fixed(-1.00) variance parameter

      2 --  Parameter  = Alpha
            Type       = Shape of scaled gamma distribution of site rates
            Prior      = Uniform(0.00,200.00)

      3 --  Parameter  = Ratemultiplier
            Type       = Partition-specific rate multiplier
            Prior      = Fixed(1.0)

      4 --  Parameter  = Tau
            Type       = Topology
            Prior      = Prior on topologies obeys constraints
            Subparam.  = V
```

```
   5 --  Parameter  = V
         Type       = Branch lengths
         Prior      = Clock:Uniform
                      Tree age has an Exponential(1.000) distribution
                      Node ages are not constrained

   6 --  Parameter  = Clockrate
         Type       = Base rate of clock
         Prior      = Fixed(1.000000)
                      The clock rate is constant (strict clock)


Number of taxa = 40
Number of characters = 116

The MCMC sampler will use the following moves:
   With prob.  Chain will use move
      2.38 %   Multiplier(Alpha)
     11.90 %   ExtSprClock(Tau,V)
     23.81 %   NNIClock(Tau,V)
     11.90 %   ParsSPRClock(Tau,V)
     47.62 %   NodesliderClock(V)
      2.38 %   TreeStretch(V)

Division 1 has 81 unique site patterns
Initializing conditional likelihoods
Using standard non-SSE likelihood calculator for division 1 (single-precision)

Initial log likelihoods and log prior probs:
   Chain 1 -- -1621.647120 -- -93.249778
   Chain 2 -- -1592.249470 -- -93.249778
   Chain 3 -- -1633.133426 -- -93.249778
   Chain 4 -- -1575.340102 -- -93.249778


Chain results (2000000 generations requested):

    0 -- [-1621.647] (-1592.249) (-1633.133) (-1575.340)
10000 -- (-1028.739) (-1036.015) (-1042.466) [-1020.536] -- 0:19:54
20000 -- (-1045.088) (-1012.996) (-1046.210) [-1000.570] -- 0:19:48
30000 -- (-1020.521) (-1002.542) (-1016.358) [-1003.863] -- 0:19:42
40000 -- (-1023.325) (-1013.502) [-995.452] (-1014.276) -- 0:19:36
50000 -- (-1017.019) (-1015.170) [-990.457] (-1020.263) -- 0:19:30
60000 -- (-1025.688) [-1001.691] (-1020.365) (-1017.846) -- 0:19:24
70000 -- (-1010.197) [-1009.446] (-1003.460) (-1016.008) -- 0:18:50
80000 -- [-1011.420] (-1014.188) (-1019.493) (-1014.499) -- 0:18:48
90000 -- (-1006.714) [-1003.648] (-1019.475) (-1006.100) -- 0:18:44
100000 -- (-1011.789) (-1015.559) (-1016.683) [-1009.579] -- 0:18:41
110000 -- [-1005.796] (-1017.809) (-1024.687) (-1014.136) -- 0:18:36
120000 -- (-1016.930) (-1007.096) (-1021.067) [-1004.768] -- 0:18:32
130000 -- [-1023.588] (-1027.211) (-1019.895) (-1012.184) -- 0:18:27
140000 -- (-1005.673) (-1010.089) (-1015.651) [-1009.047] -- 0:18:22
150000 -- (-1020.893) (-1006.895) [-991.682] (-1033.428) -- 0:18:05
160000 -- (-1015.701) (-1011.841) [-1013.735] (-1002.196) -- 0:18:01
170000 -- (-1017.437) (-1044.298) (-1005.647) [-1009.599] -- 0:17:56
180000 -- (-1021.359) (-1002.082) (-1016.087) [-1002.729] -- 0:17:51
190000 -- (-1022.265) (-1009.941) (-1010.601) [-1000.119] -- 0:17:46
200000 -- (-1029.121) (-1027.367) [-1003.992] (-1021.071) -- 0:17:33
```

```
210000 -- (-1008.409) (-1009.661) (-1018.253) [-1014.320] -- 0:17:45
220000 -- (-1023.948) [-995.109] (-1022.535) (-1010.661) -- 0:17:39
230000 -- (-1003.654) (-1010.333) [-1003.976] (-1012.312) -- 0:17:34
240000 -- (-1006.234) (-997.865) (-1017.299) [-1008.493] -- 0:17:28
250000 -- (-1011.312) (-1016.119) [-1026.958] (-1017.007) -- 0:17:23
260000 -- (-999.963) (-1027.636) (-1015.583) [-1006.645] -- 0:17:17
270000 -- (-1025.851) [-1004.928] (-1026.834) (-1000.745) -- 0:17:05
280000 -- (-1025.845) (-996.241) (-1013.331) [-1014.689] -- 0:16:59
290000 -- (-1009.085) [-1011.515] (-1017.801) (-1007.360) -- 0:16:54
300000 -- (-1011.249) (-1016.287) [-1004.812] (-1029.991) -- 0:16:43
310000 -- (-1015.481) (-1007.481) [-1007.868] (-1019.198) -- 0:16:37
320000 -- (-1012.726) (-1016.447) [-1016.620] (-1015.538) -- 0:16:32
330000 -- (-1011.591) (-1014.093) (-1024.598) [-1027.289] -- 0:16:26
340000 -- (-1007.399) (-1009.124) (-1004.371) [-1003.718] -- 0:16:21
350000 -- (-1005.471) (-1005.793) [-998.373] (-1033.099) -- 0:16:11
360000 -- (-1026.010) [-1004.184] (-1015.620) (-1005.896) -- 0:16:05
370000 -- (-1024.046) (-1019.174) [-996.148] (-1011.345) -- 0:16:00
380000 -- [-1009.413] (-1008.610) (-1006.646) (-1013.255) -- 0:15:54
390000 -- [-1011.596] (-1013.265) (-1016.090) (-1029.346) -- 0:15:45
400000 -- [-1005.069] (-1007.953) (-1022.807) (-1017.452) -- 0:15:44
410000 -- (-1022.164) (-1002.936) (-1019.030) [-1004.159] -- 0:15:38
420000 -- (-1017.577) (-1009.683) (-1018.047) [-1006.836] -- 0:15:32
430000 -- (-1014.977) (-1014.136) [-1007.215] (-1002.965) -- 0:15:23
440000 -- [-1001.969] (-1005.414) (-1002.851) (-1020.846) -- 0:15:18
450000 -- [-1010.898] (-1024.177) (-1013.844) (-1004.979) -- 0:15:12
460000 -- [-1007.375] (-1024.528) (-1013.980) (-1010.060) -- 0:15:07
470000 -- (-1016.171) [-1021.828] (-1029.044) (-1009.960) -- 0:15:01
480000 -- (-1027.665) (-1025.530) (-1005.897) [-1011.579] -- 0:14:53
490000 -- (-1042.298) (-1013.445) [-993.752] (-1022.066) -- 0:14:47
500000 -- [-1006.274] (-1006.998) (-1020.058) (-1012.929) -- 0:14:42
510000 -- (-1013.059) (-1020.123) [-1018.778] (-1033.993) -- 0:14:39
520000 -- [-1005.962] (-1015.984) (-1013.603) (-1010.642) -- 0:14:33
530000 -- (-1033.233) (-1008.576) [-1018.045] (-1023.188) -- 0:14:28
540000 -- [-1005.473] (-1022.940) (-1019.235) (-1020.007) -- 0:14:22
550000 -- (-1024.444) (-1005.461) [-1003.346] (-1019.444) -- 0:14:16
560000 -- (-1013.884) (-1027.105) [-1002.710] (-1017.864) -- 0:14:08
570000 -- [-1004.944] (-1005.922) (-1003.306) (-1025.436) -- 0:14:02
580000 -- (-1011.828) (-1003.024) [-1004.551] (-1024.916) -- 0:13:57
590000 -- (-1008.870) [-1008.526] (-1014.506) (-1016.521) -- 0:13:51
600000 -- [-1006.229] (-1004.051) (-1014.935) (-1022.570) -- 0:13:46
610000 -- (-1009.967) (-1008.053) [-1002.212] (-1009.617) -- 0:13:40
620000 -- [-1014.093] (-1006.169) (-1011.886) (-1009.182) -- 0:13:34
630000 -- [-1006.522] (-1032.301) (-1025.145) (-1017.590) -- 0:13:28
640000 -- (-1011.318) (-1015.783) (-1010.288) [-1014.531] -- 0:13:23
650000 -- (-1020.133) [-1009.421] (-1021.844) (-1011.591) -- 0:13:17
660000 -- [-1005.344] (-1020.831) (-1028.511) (-1029.863) -- 0:13:09
670000 -- (-996.378) (-1021.608) (-1008.217) [-993.651] -- 0:13:04
680000 -- (-1018.575) (-1032.091) (-1015.942) [-1019.182] -- 0:12:58
690000 -- [-1008.255] (-1012.471) (-1031.978) (-1026.745) -- 0:12:54
700000 -- [-1003.531] (-999.518) (-1007.559) (-1003.923) -- 0:12:48
710000 -- (-1012.906) [-1004.163] (-1034.484) (-1008.115) -- 0:12:43
720000 -- [-1014.379] (-1010.820) (-1032.175) (-1016.982) -- 0:12:37
730000 -- (-1013.607) (-1007.597) (-1018.865) [-1014.726] -- 0:12:31
740000 -- (-1016.461) [-1011.403] (-1007.873) (-1000.126) -- 0:12:25
750000 -- [-1003.508] (-1008.402) (-1021.492) (-1031.679) -- 0:12:20
760000 -- (-1008.858) [-1005.331] (-1009.687) (-1014.012) -- 0:12:12
770000 -- (-1024.606) (-1008.203) (-1004.744) [-1011.825] -- 0:12:06
780000 -- [-1004.854] (-1020.586) (-1011.578) (-1005.325) -- 0:12:01
```

```
790000 -- (-1017.457) (-1018.735) (-1007.303) [-998.554] -- 0:11:55
800000 -- (-1009.723) (-1021.077) [-998.028] (-997.442) -- 0:11:49
810000 -- (-1003.867) (-1008.095) (-1011.783) [-1013.459] -- 0:11:46
820000 -- (-1035.514) [-1010.655] (-1010.580) (-1001.138) -- 0:11:39
830000 -- [-997.851] (-1002.037) (-1018.289) (-1008.216) -- 0:11:33
840000 -- (-1006.676) (-1010.737) (-1010.589) [-1010.792] -- 0:11:27
850000 -- (-1018.364) (-1008.317) (-1014.378) [-1001.501] -- 0:11:21
860000 -- (-1021.436) [-1010.950] (-1005.742) (-1037.870) -- 0:11:16
870000 -- (-1001.760) (-1013.312) (-1029.281) [-1001.023] -- 0:11:10
880000 -- (-1006.871) (-1007.812) [-1015.737] (-1009.957) -- 0:11:04
890000 -- (-1008.353) (-1019.206) (-1031.399) [-1001.545] -- 0:10:58
900000 -- (-1012.013) [-1004.281] (-1016.681) (-1021.461) -- 0:10:51
910000 -- [-1005.411] (-1008.072) (-1008.114) (-1012.823) -- 0:10:45
920000 -- (-1021.312) (-1014.828) [-1002.497] (-1012.104) -- 0:10:39
930000 -- (-1010.929) (-1023.528) (-1011.387) [-1009.112] -- 0:10:33
940000 -- (-996.431) [-1003.137] (-1026.287) (-1014.202) -- 0:10:28
950000 -- (-1024.055) [-1009.419] (-1020.389) (-1024.903) -- 0:10:22
960000 -- (-1041.328) (-1010.456) (-1023.506) [-997.472] -- 0:10:15
970000 -- (-1014.609) [-1001.060] (-1023.159) (-1024.159) -- 0:10:09
980000 -- (-1011.301) (-1054.548) [-1012.624] (-1010.832) -- 0:10:03
990000 -- [-1002.612] (-1014.374) (-1019.434) (-1002.944) -- 0:09:58
1000000 -- (-1003.871) (-1015.941) [-995.716] (-1025.840) -- 0:09:52
1010000 -- (-1002.151) [-1002.526] (-1014.542) (-1003.980) -- 0:09:46
1020000 -- (-1005.365) (-1007.142) (-1014.304) [-1011.118] -- 0:09:40
1030000 -- (-1018.665) (-1009.684) (-1015.946) [-1008.804] -- 0:09:34
1040000 -- [-999.383] (-1021.723) (-1028.371) (-1007.834) -- 0:09:28
1050000 -- (-1004.099) (-1024.959) (-1001.189) [-1007.905] -- 0:09:22
1060000 -- [-988.730] (-1004.434) (-1015.640) (-1006.683) -- 0:09:16
1070000 -- [-999.262] (-1004.973) (-1006.749) (-1005.523) -- 0:09:10
1080000 -- (-1013.961) (-1020.744) [-1031.183] (-1010.001) -- 0:09:04
1090000 -- (-1006.529) [-998.654] (-1007.874) (-1024.007) -- 0:08:58
1100000 -- (-1024.376) (-1002.335) (-1029.549) [-1009.602] -- 0:08:52
1110000 -- (-1012.109) (-1014.298) (-1019.636) [-1000.842] -- 0:08:46
1120000 -- (-1009.900) (-1010.068) (-1014.760) [-1001.766] -- 0:08:40
1130000 -- (-1017.094) (-1021.225) (-1004.484) [-1008.108] -- 0:08:35
1140000 -- [-1006.385] (-1007.006) (-1005.314) (-1017.679) -- 0:08:29
1150000 -- (-1003.342) [-1001.221] (-994.584) (-1025.503) -- 0:08:23
1160000 -- [-1006.083] (-1003.030) (-1022.906) (-1013.355) -- 0:08:16
1170000 -- (-1004.192) (-1015.177) [-999.339] (-1009.493) -- 0:08:10
1180000 -- (-1020.845) (-1015.033) (-1018.270) [-1002.128] -- 0:08:05
1190000 -- [-1003.126] (-1016.569) (-1002.137) (-1019.438) -- 0:07:59
1200000 -- [-996.806] (-999.814) (-1002.764) (-1013.049) -- 0:07:53
1210000 -- [-1001.722] (-1026.365) (-1016.175) (-1018.902) -- 0:07:47
1220000 -- (-1015.995) [-1001.441] (-1009.432) (-1012.153) -- 0:07:41
1230000 -- (-998.193) (-1006.988) (-1050.419) [-1010.159] -- 0:07:35
1240000 -- (-1024.412) (-1008.028) [-1003.617] (-1023.794) -- 0:07:29
1250000 -- (-1031.945) [-1006.180] (-1008.428) (-1006.075) -- 0:07:23
1260000 -- (-1014.436) (-1011.536) [-994.617] (-1007.333) -- 0:07:17
1270000 -- (-1016.975) (-1015.365) (-1025.255) [-1003.032] -- 0:07:11
1280000 -- [-1002.847] (-1017.189) (-1022.756) (-1020.052) -- 0:07:05
1290000 -- (-1011.274) (-1016.510) [-1013.226] (-1002.231) -- 0:06:59
1300000 -- (-1020.554) [-1006.932] (-1010.887) (-1011.382) -- 0:06:54
1310000 -- (-1017.449) [-1013.921] (-997.515) (-1032.533) -- 0:06:48
1320000 -- (-1023.736) [-1013.262] (-1012.495) (-1017.343) -- 0:06:42
1330000 -- [-1000.716] (-1001.966) (-1013.990) (-1009.810) -- 0:06:36
1340000 -- [-1004.158] (-998.993) (-1026.247) (-1011.871) -- 0:06:30
1350000 -- (-1012.705) (-1021.766) [-1011.275] (-1020.113) -- 0:06:24
1360000 -- [-1010.105] (-1019.915) (-1025.295) (-1003.399) -- 0:06:18
```

```
1370000 -- [-1006.823] (-1009.164) (-1025.643) (-1000.132) -- 0:06:12
1380000 -- (-1009.512) (-1009.387) [-1011.366] (-1001.077) -- 0:06:06
1390000 -- [-1004.520] (-999.380) (-1004.833) (-1012.013) -- 0:06:00
1400000 -- (-1017.817) (-1002.724) (-1025.263) [-1001.223] -- 0:05:54
1410000 -- (-1007.228) (-1007.793) (-1012.588) [-1013.835] -- 0:05:48
1420000 -- (-1014.598) [-1014.575] (-1027.825) (-1014.534) -- 0:05:43
1430000 -- (-1024.554) (-1005.007) [-996.712] (-1008.030) -- 0:05:38
1440000 -- [-1002.040] (-1017.625) (-1014.425) (-1021.520) -- 0:05:32
1450000 -- (-1005.877) [-1006.654] (-1015.587) (-1006.798) -- 0:05:26
1460000 -- (-1024.343) [-1002.998] (-1009.389) (-1039.435) -- 0:05:20
1470000 -- (-1024.162) (-1004.402) (-1027.136) [-1015.784] -- 0:05:14
1480000 -- [-1000.024] (-1007.262) (-1003.811) (-1022.153) -- 0:05:08
1490000 -- (-1008.800) [-1001.139] (-1043.617) (-1029.495) -- 0:05:02
1500000 -- [-1007.570] (-1023.487) (-1013.776) (-1017.848) -- 0:04:56
1510000 -- (-1005.390) [-1015.025] (-1010.639) (-1016.422) -- 0:04:50
1520000 -- (-1025.913) [-1002.642] (-1023.501) (-1001.147) -- 0:04:44
1530000 -- (-1006.004) [-1001.935] (-1010.789) (-1010.275) -- 0:04:38
1540000 -- (-1012.211) [-1003.551] (-1019.575) (-1010.811) -- 0:04:32
1550000 -- (-999.041) [-995.241] (-1002.874) (-1014.930) -- 0:04:26
1560000 -- [-1020.326] (-1024.716) (-1020.863) (-1011.956) -- 0:04:20
1570000 -- (-1025.741) (-1008.577) (-1001.349) [-1002.617] -- 0:04:14
1580000 -- (-1020.624) (-1013.267) [-997.932] (-1007.265) -- 0:04:09
1590000 -- (-1004.218) (-1001.517) (-1013.014) [-1008.366] -- 0:04:02
1600000 -- [-1009.095] (-1021.691) (-996.973) (-1007.550) -- 0:03:57
1610000 -- (-999.023) [-994.537] (-1018.087) (-1013.087) -- 0:03:51
1620000 -- [-1004.278] (-1016.845) (-1028.289) (-1026.997) -- 0:03:45
1630000 -- (-1032.487) [-1001.177] (-1010.564) (-1018.761) -- 0:03:39
1640000 -- [-1006.698] (-1000.865) (-1013.335) (-1005.854) -- 0:03:33
1650000 -- [-996.261] (-1025.257) (-1013.089) (-1009.581) -- 0:03:27
1660000 -- (-1020.400) (-1011.972) (-1015.072) [-1005.024] -- 0:03:21
1670000 -- (-1017.090) [-1003.633] (-1019.213) (-1013.811) -- 0:03:15
1680000 -- (-1019.155) (-1008.609) [-1004.857] (-1018.926) -- 0:03:09
1690000 -- (-1025.212) [-1004.459] (-998.700) (-1012.996) -- 0:03:03
1700000 -- (-1014.646) (-1015.331) [-1000.895] (-1011.521) -- 0:02:57
1710000 -- (-1030.510) (-1028.287) (-1021.928) [-1001.915] -- 0:02:51
1720000 -- [-1004.680] (-1017.617) (-1013.717) (-1015.702) -- 0:02:45
1730000 -- [-1002.523] (-1028.054) (-1007.812) (-1009.944) -- 0:02:39
1740000 -- (-998.520) [-1020.882] (-1003.923) (-1016.380) -- 0:02:34
1750000 -- [-1015.786] (-1011.427) (-1003.391) (-1016.979) -- 0:02:28
1760000 -- (-1017.366) (-1017.620) (-1024.564) [-996.259] -- 0:02:22
1770000 -- (-1034.838) [-996.995] (-1016.746) (-1024.201) -- 0:02:16
1780000 -- (-1013.279) (-1008.245) [-1011.540] (-1013.103) -- 0:02:10
1790000 -- [-1002.172] (-1020.978) (-1030.276) (-1020.570) -- 0:02:04
1800000 -- (-1014.146) (-1028.866) [-1004.756] (-1013.892) -- 0:01:58
1810000 -- (-1007.502) [-1007.299] (-1012.769) (-1014.627) -- 0:01:52
1820000 -- (-1030.790) (-1007.458) (-1013.781) [-1010.303] -- 0:01:46
1830000 -- (-1014.613) (-1036.172) (-1006.008) [-1004.677] -- 0:01:40
1840000 -- (-1009.741) (-1023.893) (-1016.996) [-1008.143] -- 0:01:34
1850000 -- [-1009.368] (-1031.814) (-1024.418) (-1005.719) -- 0:01:28
1860000 -- (-1029.335) (-1017.162) (-1022.860) [-1006.332] -- 0:01:22
1870000 -- (-1017.730) [-1008.731] (-1005.959) (-1031.870) -- 0:01:16
1880000 -- (-1005.006) [-995.934] (-1038.465) (-1014.510) -- 0:01:11
1890000 -- [-1003.061] (-1006.095) (-1032.343) (-1037.317) -- 0:01:05
1900000 -- (-1007.907) (-1004.618) (-1018.096) [-1009.068] -- 0:00:59
1910000 -- (-1024.493) (-1011.079) (-1013.591) [-998.495] -- 0:00:53
1920000 -- (-1017.997) [-1010.508] (-1012.281) (-1034.992) -- 0:00:47
1930000 -- (-1014.825) (-1020.206) (-1011.872) [-996.885] -- 0:00:41
1940000 -- (-1006.821) (-1011.509) (-1029.874) [-997.373] -- 0:00:35
```

```
   1950000 -- (-1014.504) (-998.218) [-1008.689] (-1015.510) -- 0:00:29
   1960000 -- [-1000.227] (-1024.644) (-1018.482) (-1002.619) -- 0:00:23
   1970000 -- [-1009.259] (-1022.689) (-1020.227) (-1005.425) -- 0:00:17
   1980000 -- (-1006.788) [-1005.542] (-1000.144) (-1030.185) -- 0:00:11
   1990000 -- [-1009.699] (-1013.423) (-1016.056) (-1009.990) -- 0:00:05
   2000000 -- (-999.693) [-999.653] (-1018.502) (-1022.327) -- 0:00:00

   Continue with analysis? (yes/no): n

   Analysis completed in 19 mins 45 seconds
   Analysis used 1148.54 seconds of CPU time
   Log likelihood of best state for "cold" chain was -983.67

   Acceptance rates for the moves in the "cold" chain:
      With prob.   (last 100)   chain accepted proposals by move
         34.5 %     ( 31 %)     Multiplier(Alpha)
         18.6 %     ( 17 %)     ExtSprClock(Tau,V)
         44.4 %     ( 58 %)     NNIClock(Tau,V)
         15.9 %     ( 27 %)     ParsSPRClock(Tau,V)
         70.1 %     ( 74 %)     NodesliderClock(V)
         71.1 %     ( 31 %)     TreeStretch(V)

   Chain swap information:

                1       2       3       4
        ----------------------------------
      1 |            0.46    0.20    0.07
      2 |  333336            0.57    0.27
      3 |  332812  332788            0.60
      4 |  333315  334718  333031

   Upper diagonal: Proportion of successful state exchanges between chains
   Lower diagonal: Number of attempted state exchanges between chains

   Chain information:

     ID -- Heat
    -----------
      1 -- 1.00  (cold chain)
      2 -- 0.91
      3 -- 0.83
      4 -- 0.77

   Heat = 1 / (1 + T * (ID - 1))
      (where T = 0.10 is the temperature and ID is the chain number)


MrBayes > sumt relburnin=yes

   Using relative burnin (a fraction of samples discarded).
   Summarizing trees in file "DY-26Dec-strict-Hout-Ynotin.t"
   Using relative burnin ('relburnin=yes'), discarding the first 25 % of sampled trees
   Writing statistics to files DY-26Dec-strict-Hout-Ynotin.<parts|tstat|vstat|trprobs|con>
   Examining file ...
   Found one tree block in file "DY-26Dec-strict-Hout-Ynotin.t" with 4001 trees in last block

   Tree reading status:
```

```
0      10      20      30      40      50      60      70      80      90     100
v-------v-------v-------v-------v-------v-------v-------v-------v-------v-------v
*********************************************************************************

Read 4001 trees from last tree block (sampling 3001 of them)

General explanation:

In an unrooted tree, a taxon bipartition (split) is specified by removing a
branch, thereby dividing the species into those to the left and those to the
right of the branch. Here, taxa to one side of the removed branch are denoted
'.' and those to the other side are denoted '*'. Specifically, the '.' symbol
is used for the taxa on the same side as the outgroup.

In a rooted or clock tree, the tree is rooted using the model and not by
reference to an outgroup. Each bipartition therefore corresponds to a clade,
that is, a group that includes all the descendants of a particular branch in
the tree.  Taxa that are included in each clade are denoted using '*', and
taxa that are not included are denoted using the '.' symbol.

The output first includes a key to all the bipartitions with frequency larger
or equual to (Minpartfreq) in at least one run. Minpartfreq is a paramiter to
sumt command and currently it is set to 0.10.  This is followed by a table
with statistics for the informative bipartitions (those including at least
two taxa), sorted from highest to lowest probability. For each bipartition,
the table gives the number of times the partition or split was observed in all
runs (#obs) and the posterior probability of the bipartition (Probab.), which
is the same as the split frequency. If several runs are summarized, this is
followed by the minimum split frequency (Min(s)), the maximum frequency
(Max(s)), and the standard deviation of frequencies (Stddev(s)) across runs.
The latter value should approach 0 for all bipartitions as MCMC runs converge.

This is followed by a table summarizing branch lengths, node heights (if a
clock model was used) and relaxed clock parameters (if a relaxed clock model
was used). The mean, variance, and 95 % credible interval are given for each
of these parameters. If several runs are summarized, the potential scale
reduction factor (PSRF) is also given; it should approach 1 as runs converge.
Node heights will take calibration points into account, if such points were
used in the analysis.

Note that Stddev may be unreliable if the partition is not present in all
runs (the last column indicates the number of runs that sampled the partition
if more than one run is summarized). The PSRF is not calculated at all if
the partition is not present in all runs.The PSRF is also sensitive to small
sample sizes and it should only be considered a rough guide to convergence
since some of the assumptions allowing one to interpret it as a true potential
scale reduction factor are violated in MrBayes.

List of taxa in bipartitions:

   1 -- hax
   2 -- gwi
   3 -- dgr
   4 -- scsh
   5 -- xsl
   6 -- bea
   7 -- crx
   8 -- chp
```

```
   9 -- txc
  10 -- haa
  11 -- ing
  12 -- kuu
  13 -- hoi
  14 -- koy
  15 -- taa
  16 -- aht
  17 -- tfn
  18 -- kkz
  19 -- tcb
  20 -- tau
  21 -- ttmN
  22 -- tceS
  23 -- eya
  24 -- tli
  25 -- gce
  26 -- tol
  27 -- cco
  28 -- hup
  29 -- mtl
  30 -- wlk
  31 -- kto
  32 -- apc
  33 -- apw
  34 -- apj
  35 -- nav
  36 -- apk
  37 -- apl
  38 -- srs
  39 -- ket
  40 -- zko

Key to taxon bipartitions (saved to file "DY-26Dec-strict-Hout-Ynotin.parts"):

 ID -- Partition
-----------------------------------------------
  0 -- ****************************************
  1 -- *.......................................
  2 -- .*......................................
  3 -- ..*.....................................
  4 -- ...*....................................
  5 -- ....*...................................
  6 -- .....*..................................
  7 -- ......*.................................
  8 -- .......*................................
  9 -- ........*...............................
 10 -- .........*..............................
 11 -- ..........*.............................
 12 -- ...........*............................
 13 -- ............*...........................
 14 -- .............*..........................
 15 -- ..............*.........................
 16 -- ...............*........................
 17 -- ................*.......................
 18 -- .................*......................
 19 -- ..................*.....................
 20 -- ...................*....................
```

```
21 -- ....................*...................
22 -- .....................*..................
23 -- ......................*.................
24 -- .......................*................
25 -- ........................*...............
26 -- .........................*..............
27 -- ..........................*.............
28 -- ...........................*............
29 -- ............................*...........
30 -- .............................*..........
31 -- ..............................*.........
32 -- ...............................*........
33 -- ................................*.......
34 -- .................................*......
35 -- ..................................*.....
36 -- ...................................*....
37 -- ....................................*...
38 -- .....................................*..
39 -- ......................................*.
40 -- .......................................*
41 -- ......................................**
42 -- .*************************************..
43 -- ...................................**...
44 -- ...............................*******..
45 -- ...............................****.....
46 -- ......................**................
47 -- ..............*...**....................
48 -- .............................**.........
49 -- ..........*.**..........................
50 -- ...........................****.........
51 -- ...............................******...
52 -- .***.**.................................
53 -- .*********************..***....*******..
54 -- ...............................***......
55 -- ....*............*......................
56 -- ............**..........................
57 -- ....*..*.*....*..*****..................
58 -- ....*..*.*.......*..**..................
59 -- ...............................**.......
60 -- .......*............**..................
61 -- ............................***.........
62 -- .....**.................................
63 -- ..............*...*.....................
64 -- ........................***.............
65 -- ....................**..................
66 -- *.....................................**
67 -- ........................**..............
68 -- ....*..*.**.***..*****..................
69 -- .........................**.............
70 -- .*******.*....*..*****..................
71 -- .**.....................................
72 -- .**************************....*******..
73 -- **************************************..
74 -- ..........****..........................
75 -- ..................**....................
76 -- .*********************..................
77 -- ..**....................................
78 -- ........................***....*******..
```

```
 79 -- ..........*.***...**....................
 80 -- ...*..*.................................
 81 -- ...............**.......................
 82 -- ...*.**.................................
 83 -- .*********************..**************..
 84 -- ....*....*.......*......................
 85 -- ..........*.*...........................
 86 -- ...........................*.**.........
 87 -- .*******.**.***..*****..................
 88 -- .......*............*...................
 89 -- .***************************************
 90 -- .*.*.**.................................
 91 -- ................................**......
 92 -- .......*.............*..................
 93 -- .***..*.................................
 94 -- ...............................*.*......
 95 -- .*...**.................................
 96 -- .*********************.........*******..
 97 -- ....*..*.........*..**..................
 98 -- ...................................***..
 99 -- ........*.......*.......................
100 -- ........*..*............................
101 -- ..............*....*....................
102 -- .***....................................
103 -- ...............*...............*******..
104 -- .......*.*..........**..................
105 -- ...........*....*.......................
106 -- ........*..*....*.......................
107 -- ........*......*........................
108 -- ......................**...****.........
109 -- ........*..*...**.......................
110 -- ...............................**.*.....
111 -- ..........*..*..........................
112 -- ............................**..........
113 -- .*******.*....*..*****.........*******..
-----------------------------------------------
```

```
Summary statistics for informative taxon bipartitions (clades)
   (saved to file "DY-26Dec-strict-Hout-Ynotin.tstat"):

 ID   #obs    Probab.
---------------------
 41  3001    1.000000
 42  3001    1.000000
 43  3000    0.999667
 44  2742    0.913695
 45  2596    0.865045
 46  2489    0.829390
 47  2453    0.817394
 48  2427    0.808730
 49  2386    0.795068
 50  2363    0.787404
 51  2223    0.740753
 52  2189    0.729424
 53  2101    0.700100
 54  2095    0.698101
 55  1902    0.633789
 56  1840    0.613129
```

57	1815	0.604798
58	1780	0.593136
59	1779	0.592802
60	1753	0.584139
61	1711	0.570143
62	1678	0.559147
63	1550	0.516495
64	1550	0.516495
65	1344	0.447851
66	1315	0.438187
67	1308	0.435855
68	1259	0.419527
69	1233	0.410863
70	1232	0.410530
71	1224	0.407864
72	1208	0.402532
73	1103	0.367544
74	940	0.313229
75	938	0.312562
76	898	0.299234
77	896	0.298567
78	854	0.284572
79	800	0.266578
80	796	0.265245
81	790	0.263246
82	767	0.255581
83	766	0.255248
84	717	0.238920
85	667	0.222259
86	660	0.219927
87	598	0.199267
88	595	0.198267
89	583	0.194269
90	572	0.190603
91	571	0.190270
92	566	0.188604
93	562	0.187271
94	554	0.184605
95	543	0.180940
96	512	0.170610
97	504	0.167944
98	489	0.162946
99	484	0.161280
100	437	0.145618
101	434	0.144618
102	423	0.140953
103	411	0.136954
104	403	0.134289
105	388	0.129290
106	367	0.122293
107	367	0.122293
108	349	0.116295
109	336	0.111963
110	317	0.105631
111	314	0.104632
112	302	0.100633
113	302	0.100633

Summary statistics for branch and node parameters
(saved to file "DY-26Dec-strict-Hout-Ynotin.vstat"):

Parameter	Mean	Variance	95% HPD Interval Lower	95% HPD Interval Upper	Median
length[1]	0.149021	0.001667	0.072231	0.222138	0.151446
length[2]	0.037315	0.000264	0.011224	0.070794	0.035081
length[3]	0.038527	0.000369	0.007472	0.075756	0.034763
length[4]	0.033894	0.000254	0.007696	0.066035	0.031258
length[5]	0.026910	0.000176	0.005262	0.053406	0.024800
length[6]	0.033928	0.000429	0.004540	0.077011	0.028675
length[7]	0.026087	0.000173	0.004540	0.052278	0.023711
length[8]	0.033943	0.000199	0.011678	0.062474	0.032088
length[9]	0.077738	0.000760	0.030209	0.130939	0.075027
length[10]	0.038054	0.000193	0.012807	0.064171	0.036822
length[11]	0.038798	0.000323	0.008381	0.073478	0.036493
length[12]	0.064047	0.000692	0.016491	0.115895	0.061844
length[13]	0.028292	0.000182	0.005960	0.054777	0.026238
length[14]	0.028895	0.000191	0.007661	0.058405	0.026620
length[15]	0.023359	0.000173	0.003215	0.050568	0.020581
length[16]	0.077707	0.000732	0.028646	0.129054	0.076062
length[17]	0.072818	0.000698	0.025545	0.123010	0.070354
length[18]	0.028083	0.000181	0.006291	0.054987	0.026166
length[19]	0.020803	0.000138	0.003021	0.043345	0.018432
length[20]	0.025653	0.000191	0.004518	0.054617	0.022889
length[21]	0.026936	0.000145	0.004764	0.048626	0.025352
length[22]	0.028276	0.000161	0.006865	0.052930	0.026578
length[23]	0.089295	0.001117	0.031513	0.158939	0.086111
length[24]	0.090682	0.001172	0.028811	0.158415	0.087102
length[25]	0.065840	0.000718	0.020920	0.119430	0.062619
length[26]	0.056992	0.000558	0.015092	0.101862	0.054247
length[27]	0.064051	0.000686	0.016303	0.112871	0.060695
length[28]	0.080657	0.001067	0.025057	0.144264	0.077095
length[29]	0.064110	0.000724	0.019062	0.115421	0.060244
length[30]	0.036410	0.000333	0.007256	0.071157	0.032948
length[31]	0.036934	0.000366	0.007550	0.074490	0.033083
length[32]	0.007055	0.000044	0.000003	0.020263	0.005045
length[33]	0.007046	0.000042	0.000003	0.019914	0.005019
length[34]	0.011777	0.000070	0.000212	0.027996	0.009934
length[35]	0.023291	0.000135	0.004389	0.045547	0.021076
length[36]	0.004294	0.000020	0.000005	0.012891	0.002917
length[37]	0.004297	0.000021	0.000005	0.012891	0.002917
length[38]	0.052303	0.000442	0.016462	0.092904	0.049761
length[39]	0.067992	0.000609	0.024310	0.114645	0.065959
length[40]	0.067992	0.000609	0.024310	0.114645	0.065959
length[41]	0.083565	0.000789	0.033112	0.138799	0.081689
length[42]	0.011059	0.000103	0.000007	0.031243	0.008255
length[43]	0.031191	0.000209	0.006132	0.058524	0.029229
length[44]	0.035025	0.000345	0.004089	0.072495	0.033027
length[45]	0.015662	0.000112	0.000032	0.036319	0.013492
length[46]	0.046171	0.000652	0.000026	0.092932	0.042852
length[47]	0.020892	0.000159	0.000051	0.044779	0.018939
length[48]	0.029713	0.000343	0.000032	0.064265	0.026263
length[49]	0.024321	0.000280	0.000588	0.056311	0.020545
length[50]	0.050135	0.000652	0.005136	0.098935	0.047728

length[51]	0.021255	0.000222	0.000000	0.050305	0.018035
length[52]	0.021715	0.000244	0.000025	0.051070	0.019143
length[53]	0.019680	0.000151	0.000500	0.042915	0.017724
length[54]	0.012477	0.000096	0.000010	0.030689	0.010411
length[55]	0.016073	0.000110	0.000049	0.035806	0.014099
length[56]	0.015520	0.000136	0.000013	0.037846	0.012913
length[57]	0.016845	0.000103	0.000020	0.036035	0.015330
length[58]	0.014319	0.000090	0.000002	0.032932	0.012389
length[59]	0.010344	0.000073	0.000006	0.027120	0.008221
length[60]	0.009909	0.000055	0.000015	0.024532	0.008123
length[61]	0.032698	0.000573	0.000020	0.078916	0.027677
length[62]	0.021247	0.000211	0.000047	0.049778	0.018108
length[63]	0.011110	0.000092	0.000001	0.032586	0.008380
length[64]	0.026648	0.000295	0.000242	0.059169	0.023628
length[65]	0.010455	0.000070	0.000003	0.027521	0.008473
length[66]	0.009861	0.000081	0.000001	0.028046	0.007320
length[67]	0.027749	0.000400	0.000049	0.064813	0.023567
length[68]	0.019607	0.000104	0.003609	0.039578	0.017873
length[69]	0.029171	0.000425	0.000003	0.070205	0.025185
length[70]	0.023988	0.000182	0.000012	0.048041	0.022047
length[71]	0.017695	0.000143	0.000033	0.040986	0.015014
length[72]	0.013520	0.000104	0.000019	0.033348	0.011502
length[73]	0.009464	0.000082	0.000011	0.027601	0.006911
length[74]	0.025928	0.000291	0.000155	0.059801	0.022213
length[75]	0.013537	0.000158	0.000004	0.039193	0.009806
length[76]	0.012032	0.000069	0.000399	0.028022	0.010446
length[77]	0.018103	0.000164	0.000149	0.042929	0.015221
length[78]	0.016401	0.000142	0.000052	0.038212	0.013832
length[79]	0.016363	0.000177	0.000030	0.045584	0.012601
length[80]	0.014973	0.000135	0.000151	0.037706	0.012228
length[81]	0.022266	0.000253	0.000025	0.051567	0.018872
length[82]	0.016329	0.000186	0.000085	0.041764	0.012490
length[83]	0.012805	0.000104	0.000020	0.033383	0.010634
length[84]	0.007727	0.000055	0.000003	0.021404	0.005682
length[85]	0.009915	0.000089	0.000008	0.027907	0.007351
length[86]	0.022317	0.000336	0.000004	0.055773	0.018183
length[87]	0.014097	0.000126	0.000018	0.034915	0.011341
length[88]	0.006395	0.000036	0.000001	0.018014	0.004654
length[89]	0.004851	0.000025	0.000004	0.013239	0.003163
length[90]	0.018262	0.000176	0.000112	0.044099	0.015558
length[91]	0.006172	0.000034	0.000078	0.017461	0.004189
length[92]	0.005610	0.000030	0.000011	0.016308	0.003758
length[93]	0.014938	0.000133	0.000002	0.036110	0.012744
length[94]	0.006281	0.000033	0.000044	0.018665	0.004719
length[95]	0.014357	0.000136	0.000025	0.037513	0.011685
length[96]	0.011795	0.000097	0.000016	0.030274	0.009212
length[97]	0.006223	0.000042	0.000007	0.021221	0.004177
length[98]	0.011134	0.000079	0.000025	0.028473	0.008597
length[99]	0.018275	0.000259	0.000102	0.052328	0.013932
length[100]	0.018077	0.000227	0.000034	0.047424	0.014468
length[101]	0.006733	0.000054	0.000001	0.022404	0.004621
length[102]	0.010688	0.000100	0.000010	0.027235	0.008518
length[103]	0.019679	0.000181	0.000353	0.044838	0.017205
length[104]	0.005498	0.000030	0.000004	0.015555	0.004063
length[105]	0.017864	0.000195	0.000050	0.043065	0.015390
length[106]	0.018512	0.000188	0.000026	0.043973	0.016105
length[107]	0.016268	0.000172	0.000026	0.040762	0.012532
length[108]	0.009846	0.000081	0.000012	0.028003	0.007204

length[109]	0.013688	0.000129	0.000100	0.036721	0.010733
length[110]	0.005015	0.000029	0.000005	0.015598	0.003206
length[111]	0.007695	0.000059	0.000002	0.022708	0.005417
length[112]	0.028003	0.000725	0.000054	0.080112	0.019495
length[113]	0.014249	0.000100	0.000114	0.033999	0.011688
height[0]	0.156820	0.001784	0.078873	0.234185	0.159286
height[1]	0.000000	0.000000	0.000000	0.000000	0.000000
height[2]	0.000000	0.000000	0.000000	0.000000	0.000000
height[3]	0.000000	0.000000	0.000000	0.000000	0.000000
height[4]	0.000000	0.000000	0.000000	0.000000	0.000000
height[5]	0.000000	0.000000	0.000000	0.000000	0.000000
height[6]	0.000000	0.000000	0.000000	0.000000	0.000000
height[7]	0.000000	0.000000	0.000000	0.000000	0.000000
height[8]	0.000000	0.000000	0.000000	0.000000	0.000000
height[9]	0.000000	0.000000	0.000000	0.000000	0.000000
height[10]	0.000000	0.000000	0.000000	0.000000	0.000000
height[11]	0.000000	0.000000	0.000000	0.000000	0.000000
height[12]	0.000000	0.000000	0.000000	0.000000	0.000000
height[13]	0.000000	0.000000	0.000000	0.000000	0.000000
height[14]	0.000000	0.000000	0.000000	0.000000	0.000000
height[15]	0.000000	0.000000	0.000000	0.000000	0.000000
height[16]	0.000000	0.000000	0.000000	0.000000	0.000000
height[17]	0.000000	0.000000	0.000000	0.000000	0.000000
height[18]	0.000000	0.000000	0.000000	0.000000	0.000000
height[19]	0.000000	0.000000	0.000000	0.000000	0.000000
height[20]	0.000000	0.000000	0.000000	0.000000	0.000000
height[21]	0.000000	0.000000	0.000000	0.000000	0.000000
height[22]	0.000000	0.000000	0.000000	0.000000	0.000000
height[23]	0.000000	0.000000	0.000000	0.000000	0.000000
height[24]	0.000000	0.000000	0.000000	0.000000	0.000000
height[25]	0.000000	0.000000	0.000000	0.000000	0.000000
height[26]	0.000000	0.000000	0.000000	0.000000	0.000000
height[27]	0.000000	0.000000	0.000000	0.000000	0.000000
height[28]	0.000000	0.000000	0.000000	0.000000	0.000000
height[29]	0.000000	0.000000	0.000000	0.000000	0.000000
height[30]	0.000000	0.000000	0.000000	0.000000	0.000000
height[31]	0.000000	0.000000	0.000000	0.000000	0.000000
height[32]	0.000000	0.000000	0.000000	0.000000	0.000000
height[33]	0.000000	0.000000	0.000000	0.000000	0.000000
height[34]	0.000000	0.000000	0.000000	0.000000	0.000000
height[35]	0.000000	0.000000	0.000000	0.000000	0.000000
height[36]	0.000000	0.000000	0.000000	0.000000	0.000000
height[37]	0.000000	0.000000	0.000000	0.000000	0.000000
height[38]	0.000000	0.000000	0.000000	0.000000	0.000000
height[39]	0.000000	0.000000	0.000000	0.000000	0.000000
height[40]	0.000000	0.000000	0.000000	0.000000	0.000000
height[41]	0.067992	0.000609	0.024310	0.114645	0.065959
height[42]	0.141341	0.001419	0.067422	0.205022	0.145099
height[43]	0.004281	0.000020	0.000005	0.012770	0.002917
height[44]	0.053039	0.000342	0.020080	0.088245	0.051486
height[45]	0.023192	0.000106	0.006107	0.043449	0.021450
height[46]	0.084963	0.000938	0.028866	0.143800	0.083234
height[47]	0.027546	0.000156	0.007971	0.054617	0.025780
height[48]	0.032323	0.000215	0.007256	0.059388	0.030329
height[49]	0.038862	0.000245	0.011268	0.068063	0.037434
height[50]	0.083481	0.000886	0.029882	0.138897	0.081104
height[51]	0.035366	0.000176	0.012133	0.060569	0.033874
height[52]	0.053903	0.000319	0.022109	0.088277	0.053051

height[53]	0.112288	0.000875	0.053280	0.161754	0.114688
height[54]	0.012070	0.000052	0.001183	0.026022	0.010610
height[55]	0.023238	0.000117	0.006090	0.045362	0.021779
height[56]	0.025763	0.000141	0.005809	0.048093	0.024152
height[57]	0.052377	0.000247	0.022749	0.080302	0.052209
height[58]	0.043490	0.000191	0.018538	0.069192	0.042577
height[59]	0.004792	0.000021	0.000003	0.013665	0.003414
height[60]	0.032005	0.000132	0.012350	0.054066	0.031159
height[61]	0.057803	0.000455	0.018761	0.096093	0.056069
height[62]	0.022643	0.000127	0.003093	0.044245	0.020810
height[63]	0.018462	0.000094	0.002962	0.037406	0.016665
height[64]	0.072050	0.000555	0.027783	0.114274	0.071492
height[65]	0.023267	0.000112	0.006279	0.043142	0.021456
height[66]	0.144380	0.001577	0.070668	0.217572	0.146430
height[67]	0.053607	0.000433	0.015092	0.090608	0.051747
height[68]	0.064398	0.000355	0.033779	0.101876	0.062733
height[69]	0.053603	0.000436	0.018318	0.093609	0.051536
height[70]	0.071347	0.000427	0.030631	0.106287	0.072726
height[71]	0.031559	0.000201	0.008133	0.059098	0.029277
height[72]	0.128446	0.001260	0.057051	0.190155	0.131043
height[73]	0.151020	0.001621	0.075716	0.222711	0.153386
height[74]	0.059943	0.000470	0.020006	0.100306	0.058630
height[75]	0.020132	0.000122	0.003021	0.040456	0.018180
height[76]	0.101123	0.000646	0.048505	0.142494	0.102860
height[77]	0.031641	0.000190	0.007815	0.057848	0.029938
height[78]	0.094250	0.000689	0.047780	0.144646	0.094428
height[79]	0.050883	0.000294	0.020615	0.082825	0.049033
height[80]	0.025551	0.000134	0.007696	0.049627	0.023524
height[81]	0.065526	0.000463	0.029278	0.108143	0.063638
height[82]	0.037122	0.000214	0.012800	0.065197	0.035473
height[83]	0.130663	0.001227	0.062344	0.187474	0.136262
height[84]	0.035449	0.000150	0.013317	0.058154	0.033652
height[85]	0.028830	0.000176	0.005256	0.052964	0.027352
height[86]	0.062723	0.000498	0.023960	0.104471	0.061056
height[87]	0.077580	0.000493	0.037065	0.117100	0.076215
height[88]	0.028856	0.000128	0.011165	0.051318	0.028065
height[89]	0.150856	0.001735	0.066047	0.220686	0.154328
height[90]	0.043581	0.000235	0.017986	0.072589	0.042282
height[91]	0.006946	0.000030	0.000090	0.017741	0.005178
height[92]	0.028649	0.000126	0.009347	0.048488	0.027106
height[93]	0.044330	0.000225	0.018636	0.073373	0.043547
height[94]	0.006529	0.000025	0.000130	0.016044	0.005274
height[95]	0.040516	0.000221	0.014266	0.069173	0.039093
height[96]	0.102403	0.000774	0.045180	0.146079	0.104675
height[97]	0.040255	0.000178	0.015671	0.064084	0.039728
height[98]	0.037310	0.000195	0.012862	0.065803	0.035321
height[99]	0.066238	0.000588	0.024187	0.111913	0.064915
height[100]	0.062714	0.000538	0.023678	0.108604	0.060137
height[101]	0.020730	0.000098	0.004400	0.039674	0.019319
height[102]	0.042927	0.000249	0.013160	0.070348	0.041114
height[103]	0.072805	0.000622	0.028826	0.119901	0.070774
height[104]	0.038953	0.000164	0.017486	0.064967	0.038606
height[105]	0.060349	0.000475	0.023550	0.103502	0.057746
height[106]	0.068859	0.000502	0.033260	0.112444	0.066800
height[107]	0.073714	0.000598	0.031600	0.121944	0.073715
height[108]	0.129556	0.001227	0.059111	0.183467	0.132371
height[109]	0.081265	0.000548	0.045171	0.126128	0.079243
height[110]	0.017989	0.000077	0.004771	0.035068	0.016609

height[111]	0.028995	0.000172	0.008107	0.053429	0.026667
height[112]	0.052437	0.000472	0.013294	0.093269	0.051143
height[113]	0.088344	0.000667	0.037493	0.131650	0.090559

Clade credibility values:

```
/--------------------------------------------------------------------- hax (1)
|
|                                                   /----------------- gwi (2)
|                                                   |
|                                                   |----------------- dgr (3)
|                                                   |
|                /----------------73----------------+----------------- scsh (4)
|                |                                  |
|                |                                  |       /--------- bea (6)
|                |                                  \---56--+
|                |                                          \--------- crx (7)
|                |
|                |                                          /--------- xsl (5)
|                |                                  /---63--+
|                |                                  |       \--------- kkz (18)
|                |                                  |
|                |                                  |       /--------- chp (8)
|                |                                  |       |
|                |                         /---59---+---58--+--------- ttmN (21)
|                |                         |        |       |
|                |                         |        |       \--------- tceS (22)
|                |                         |        |
|                |                         |        \----------------- haa (10)
|                |------------60-----------+
|                |                         |                /--------- taa (15)
|                |                         |        /---52--+
|                |                         |        |       \--------- tcb (19)
|                |                         \---82---+
|                |                                  \----------------- tau (20)
|                |
|                |---------------------------------------------------- txc (9)
|                |
|                |                                  /----------------- ing (11)
|        /---70--+                                  |
|        |       |----------------80----------------+       /--------- hoi (13)
|        |       |                                  \---61--+
|        |       |                                          \--------- koy (14)
|        |       |
|        |       |---------------------------------------------------- kuu (12)
+        |       |
|        |       |---------------------------------------------------- aht (16)
|        |       |
|        |       |---------------------------------------------------- tfn (17)
|        |       |
|        |       |                                          /--------- gce (25)
|        |       |                                          |
|        |       |--------------------52--------------------+--------- tol (26)
|        |       |                                          |
```

```
|        |       |                                          \--------- cco (27)
|        |       |
|        |       |                                          /--------- apc (32)
|        |       |                                  /---59--+
|---100--+       |                                  |       \--------- apw (33)
|        |       |                         /---70---+
|        |       |                         |        \----------------- apj (34)
|        |       |                 /---87--+
|        |       |                 |       \-------------------------- nav (35)
|        |       |        /---74---+
|        |       |        |        |                        /--------- apk (36)
|        |       |        |        \-----------100----------+
|        |       \---91---+                                 \--------- apl (37)
|        |                |
|        |                \------------------------------------------- srs (38)
|        |
|        |                                                  /--------- eya (23)
|        |-------------------------83-----------------------+
|        |                                                  \--------- tli (24)
|        |
|        |                                 /-------------------------- hup (28)
|        |                                 |
|        \----------------79---------------+        /----------------- mtl (29)
|                                          |        |
|                                          \---57---+       /--------- wlk (30)
|                                                   \---81--+
|                                                           \--------- kto (31)
|
|                                                           /--------- ket (39)
\----------------------------100----------------------------+
                                                            \--------- zko (40)
```

Phylogram (based on median node depths):

```
/--------------------------------------------------------------------- hax (1)
|
|                                             /----------------------- gwi (2)
|                                             |
|                                             |----------------------- dgr (3)
|                                             |
|                  /--------------------------+----------------------- scsh (4)
|                  |                          |
|                  |                          |             /--------- bea (6)
|                  |                          \-------------+
|                  |                                        \--------- crx (7)
|                  |
|                  |                                        /--------- xsl (5)
|                  |                               /--------+
|                  |                               |        \--------- kkz (18)
|                  |                               |
|                  |                               |    /------------- chp (8)
|                  |                               |    |
|                  |                          /----+----+------------- ttmN (21)
|                  |                          |    |    |
|                  |                          |    |    \------------- tceS (22)
|                  |                          |    |
|                  |                          |    \------------------ haa (10)
```

```
|                  |--------------------------+
|                  |                          |               /------- taa (15)
|                  |                          |           /---+
|                  |                          |           |   \------- tcb (19)
|                  |                          \-----------+
|                  |                                      \----------- tau (20)
|                  |
|                  |-------------------------------------------------- txc (9)
|                  |
|                  |                                 /---------------- ing (11)
|     /------------+                                 |
|     |            |---------------------------------+     /---------- hoi (13)
|     |            |                                 \-----+
|     |            |                                       \---------- koy (14)
|     |            |
|     |            |-------------------------------------------------- kuu (12)
+     |            |
|     |            |-------------------------------------------------- aht (16)
|     |            |
|     |            |-------------------------------------------------- tfn (17)
|     |            |
|     |            |                  /------------------------------- gce (25)
|     |            |                  |
|     |            |------------------+------------------------------- tol (26)
|     |            |                  |
|     |            |                  \------------------------------- cco (27)
|     |            |
|     |            |                                                /- apc (32)
|     |            |                                            /---+
|-----+            |                                            |   \- apw (33)
|     |            |                                        /---+
|     |            |                                        |   \----- apj (34)
|     |            |                                  /-----+
|     |            |                                  |     \--------- nav (35)
|     |            |                           /------+
|     |            |                           |      |             /- apk (36)
|     |            |                           |      \-------------+
|     |            \---------------------------+                    \- apl (37)
|     |                                        |
|     |                                        \---------------------- srs (38)
|     |
|     |                          /------------------------------------ eya (23)
|     |--------------------------+
|     |                          \------------------------------------ tli (24)
|     |
|     |                           /----------------------------------- hup (28)
|     |                           |
|     \---------------------------+          /------------------------ mtl (29)
|                                 |          |
|                                 \----------+          /------------- wlk (30)
|                                            \----------+
|                                                       \------------- kto (31)
|
|                                       /----------------------------- ket (39)
\---------------------------------------+
                                        \----------------------------- zko (40)

---------|--------|-------|--------|--------|-------|--------|-------|
```

```
          0.14      0.12     0.10      0.08      0.06     0.04      0.02     -0.00

   [Expected changes per site]

   Calculating tree probabilities...

   Credible sets of trees (3001 trees sampled):
      50 % credible set contains 1501 trees
      90 % credible set contains 2701 trees
      95 % credible set contains 2851 trees
      99 % credible set contains 2971 trees


MrBayes > sump relburnin=yes

   Using relative burnin (a fraction of samples discarded).
   Summarizing parameters in file DY-26Dec-strict-Hout-Ynotin.p
   Writing summary statistics to file DY-26Dec-strict-Hout-Ynotin.pstat
   Using relative burnin ('relburnin=yes'), discarding the first 25 % of samples

   Below is a rough plot of the generation (x-axis) versus the log
   probability of observing the data (y-axis). You can use this
   graph to determine what the burn in for your analysis should be.
   When the log probability starts to plateau you may be at station-
   arity. Sample trees and parameters after the log probability
   plateaus. Of course, this is not a guarantee that you are at sta-
   analysis should be. When the log probability starts to plateau
   tionarity. When possible, run multiple analyses starting from dif-
   ferent random trees; if the inferences you make for independent
   analyses are the same, this is reasonable evidence that the chains
   have converged. You can use MrBayes to run several independent
   analyses simultaneously. During such a run, MrBayes will monitor
   the convergence of topologies. After the run has been completed,
   the 'sumt' and 'sump' functions will provide additional conver-
   gence diagnostics for all the parameters in your model. Remember
   that the burn in is the number of samples to discard. There are
   a total of ngen / samplefreq samples taken during a MCMC analysis.

   +------------------------------------------------------------+ -1002.152
   |                                                *           |
   |                                                            |
   |             *                *                             |
   |            *                                               |
   |    *     *     * *   *    *                     *    *     |
   |           *                                  *        * *  |
   |                             *   * *         * *     *     *|
   |* **             *        *       * *    **        *    *   |
   |        **     *    **  **                        *         |
   | *     *      *                         *   *       *       |
   |                   *            *                         * |
   |     *                      *        * *                    |
   |                               *      *                     |
   |                                           *                |
   |      *                *                                    |
   +------+-----+-----+-----+-----+-----+-----+-----+-----+-----+ -1009.401
   ^                                                            ^
   500000                                                      2000000
```

```
   Estimated marginal likelihoods for run sampled in file "DY-26Dec-strict-Hout-Ynotin.p":
      (Use the harmonic mean for Bayes factor comparisons of models)
      (Values are saved to the file /Users/msicoli/DY-Typlogical_binary_bayes-2.nex.lstat)

   Arithmetic mean   Harmonic mean
   --------------------------------
     -994.93         -1029.49
   --------------------------------


   Model parameter summaries for run sampled in file "DY-26Dec-strict-Hout-Ynotin":
      Based on a total of 3001 samples out of a total of 4001 samples
         from this analysis.
      Parameter summaries saved to file "/Users/msicoli/DY-Typlogical_binary_bayes-2.nex.pstat".

                                         95% HPD Interval
                                       --------------------
   Parameter      Mean      Variance     Lower       Upper       Median     ESS*
   ------------------------------------------------------------------------------
   TH          0.156820    0.001784    0.078873    0.234185    0.159286    384.87
   TL          2.527047    0.419954    1.269098    3.627597    2.598859    375.61
   alpha       1.211592    7.561679    0.126431    2.664872    0.928030   2141.27
   ------------------------------------------------------------------------------
   * Convergence diagnostic (ESS = Estimated Sample Size); ESS value
     below 100 may indicate that the parameter is undersampled.



MrBayes > ssp ngen=100000 diagnfreq=1000 filename=YND-Typ-Hout-YnotinND-ss

   Setting number of generations to 100000
   Setting diagnosing frequency to 1000

MrBayes > ss

   Setting chain output file names to "YND-Typ-Hout-YnotinND-ss.<p/t>"
   Running Markov chain
   MCMC stamp = 7233609125
   Seed = 208011611
   Swapseed = 1272127178
   Model settings:

      Data not partitioned --
         Datatype  = Standard
         Coding    = Variable
         # States  = Variable, up to 10
                     State frequencies are fixed to be equal
         Rates     = Gamma
                     Gamma shape parameter is uniformly dist-
                     ributed on the interval (0.00,200.00).
                     Gamma distribution is approximated using 4 categories.
                     Likelihood summarized over all rate categories in each generation.

   Active parameters:

      Parameters
      ------------------
```

```
   Statefreq        1
   Shape            2
   Ratemultiplier   3
   Topology         4
   Brlens           5
   Clockrate        6
   ------------------

   1 --  Parameter  = Alpha_symdir
         Type       = Symmetric diricihlet/beta distribution alpha_i parameter
         Prior      = Symmetric dirichlet with fixed(-1.00) variance parameter

   2 --  Parameter  = Alpha
         Type       = Shape of scaled gamma distribution of site rates
         Prior      = Uniform(0.00,200.00)

   3 --  Parameter  = Ratemultiplier
         Type       = Partition-specific rate multiplier
         Prior      = Fixed(1.0)

   4 --  Parameter  = Tau
         Type       = Topology
         Prior      = Prior on topologies obeys constraints
         Subparam.  = V

   5 --  Parameter  = V
         Type       = Branch lengths
         Prior      = Clock:Uniform
                      Tree age has an Exponential(1.000) distribution
                      Node ages are not constrained

   6 --  Parameter  = Clockrate
         Type       = Base rate of clock
         Prior      = Fixed(1.000000)
                      The clock rate is constant (strict clock)


Number of taxa = 40
Number of characters = 116

The MCMC sampler will use the following moves:
   With prob.  Chain will use move
      2.38 %   Multiplier(Alpha)
     11.90 %   ExtSprClock(Tau,V)
     23.81 %   NNIClock(Tau,V)
     11.90 %   ParsSPRClock(Tau,V)
     47.62 %   NodesliderClock(V)
      2.38 %   TreeStretch(V)

Division 1 has 81 unique site patterns
Initializing conditional likelihoods
Using standard non-SSE likelihood calculator for division 1 (single-precision)

Initial log likelihoods and log prior probs:
   Chain 1 -- -999.653079 -- -8.415782
   Chain 2 -- -999.693164 -- -35.880936
   Chain 3 -- -1022.327222 -- -32.409647
   Chain 4 -- -1018.502047 -- -36.524456
```

```
Starting stepping-stone sampling to estimate marginal likelihood.
50 steps will be used with 1500 generations (3 samples) within each step.
Total of 76500 generations (153 samples) will be collected while first
1500 generations (3 samples) will be discarded as initial burnin.
Additionally at the begining of each step 0 generations (0 samples)
will be discarded as burnin.
Sampling from posterior to prior, i.e. first step samples from close to
posterior.
NOTE: Number of generation of each step is reduced to the closest multiple
of sampling frequency. That is why, in total it will be taken 76500 gene-
rations instead of requested 100000.


Chain results (76500 generations requested):

    0 -- [-999.653] (-999.693) (-1022.327) (-1018.502)

Sampling step 1 out of 50 steps...

Sampling step 2 out of 50 steps...

Sampling step 3 out of 50 steps...

Sampling step 4 out of 50 steps...

Sampling step 5 out of 50 steps...

Sampling step 6 out of 50 steps...

10000 -- [-1014.688] (-1036.589) (-1027.784) (-1025.635) -- 0:00:46
Sampling step 7 out of 50 steps...

Sampling step 8 out of 50 steps...

Sampling step 9 out of 50 steps...

Sampling step 10 out of 50 steps...

Sampling step 11 out of 50 steps...

Sampling step 12 out of 50 steps...

Sampling step 13 out of 50 steps...

20000 -- (-1104.824) [-1079.987] (-1054.040) (-1059.171) -- 0:00:39
Sampling step 14 out of 50 steps...

Sampling step 15 out of 50 steps...

Sampling step 16 out of 50 steps...

Sampling step 17 out of 50 steps...

Sampling step 18 out of 50 steps...

Sampling step 19 out of 50 steps...
```

```
30000 -- (-1209.043) (-1184.110) [-1177.874] (-1288.336) -- 0:00:31
Sampling step 20 out of 50 steps...

Sampling step 21 out of 50 steps...

Sampling step 22 out of 50 steps...

Sampling step 23 out of 50 steps...

Sampling step 24 out of 50 steps...

Sampling step 25 out of 50 steps...

Sampling step 26 out of 50 steps...

40000 -- (-1246.398) [-1217.938] (-1401.095) (-1334.210) -- 0:00:23
Sampling step 27 out of 50 steps...

Sampling step 28 out of 50 steps...

Sampling step 29 out of 50 steps...

Sampling step 30 out of 50 steps...

Sampling step 31 out of 50 steps...

Sampling step 32 out of 50 steps...

Sampling step 33 out of 50 steps...

50000 -- [-1331.447] (-1391.716) (-1377.776) (-1371.829) -- 0:00:16
Sampling step 34 out of 50 steps...

Sampling step 35 out of 50 steps...

Sampling step 36 out of 50 steps...

Sampling step 37 out of 50 steps...

Sampling step 38 out of 50 steps...

Sampling step 39 out of 50 steps...

60000 -- (-1451.355) (-1343.767) [-1521.399] (-1416.850) -- 0:00:10
Sampling step 40 out of 50 steps...

Sampling step 41 out of 50 steps...

Sampling step 42 out of 50 steps...

Sampling step 43 out of 50 steps...

Sampling step 44 out of 50 steps...

Sampling step 45 out of 50 steps...

Sampling step 46 out of 50 steps...
```

```
70000 -- (-2313.645) (-1450.966) [-1508.777] (-2264.034) -- 0:00:04
Sampling step 47 out of 50 steps...

Sampling step 48 out of 50 steps...

Sampling step 49 out of 50 steps...

Sampling step 50 out of 50 steps...


Analysis completed in 49 seconds
Analysis used 47.91 seconds of CPU time
Log likelihood of best state for "cold" chain was -988.36

Marginal likelihood (in natural log units) estimated using stepping-stone sampling based on
50 steps with 1500 generations (3 samples) within each step.

    Run   Marginal likelihood (ln)
    ------------------------------
      1     -1118.81
    ------------------------------
More statistics on stepping-stone sampling is dumped to YND-Typ-Hout-YnotinND-ss.ss file.

Plot of average standard deviation of split frequencies across steps.
Points at -1.0 (y-axis) indicate that there were no splits
above minimum frequency for corresponding step.
+------------------------------------------------------------+ 0.000
|                                                            |
|                                                            |
|                                                            |
|                                                            |
|                                                            |
|                                                            |
|                                                            |
|                                                            |
|                                                            |
|                                                            |
|                                                            |
|                                                            |
|                                                            |
|                                                            |
|***** **** ***** **** ***** **** ***** **** ***** **** *****|
+------+-----+-----+-----+-----+-----+-----+-----+-----+-----+ 0.000
^                                                           ^
1                                                          50


Acceptance rates for the moves in the "cold" chain:
   With prob.   (last 100)   chain accepted proposals by move
      48.3 %     ( 56 %)     Multiplier(Alpha)
      44.0 %     ( 68 %)     ExtSprClock(Tau,V)
      78.0 %     (100 %)     NNIClock(Tau,V)
      34.2 %     ( 32 %)     ParsSPRClock(Tau,V)
      85.9 %     ( 98 %)     NodesliderClock(V)
      52.3 %     ( 87 %)     TreeStretch(V)

Chain swap information:
```

```
               1      2      3      4
          ------------------------------
        1 |          0.22   0.07   0.02
        2 |  12788          0.32   0.09
        3 |  12613  12830          0.41
        4 |  12848  12764  12657

     Upper diagonal: Proportion of successful state exchanges between chains
     Lower diagonal: Number of attempted state exchanges between chains

     Chain information:

       ID -- Heat
      -----------
        1 -- 1.00  (cold chain)
        2 -- 0.91
        3 -- 0.83
        4 -- 0.77

     Heat = 1 / (1 + T * (ID - 1))
        (where T = 0.10 is the temperature and ID is the chain number)


  MrBayes > ss

     Running Markov chain
     MCMC stamp = 7068357459
     Seed = 1721729764
     Swapseed = 370168243
     Model settings:

        Data not partitioned --
           Datatype  = Standard
           Coding    = Variable
           # States  = Variable, up to 10
                       State frequencies are fixed to be equal
           Rates     = Gamma
                       Gamma shape parameter is uniformly dist-
                       ributed on the interval (0.00,200.00).
                       Gamma distribution is approximated using 4 categories.
                       Likelihood summarized over all rate categories in each generation.

     Active parameters:

        Parameters
        ------------------
        Statefreq        1
        Shape            2
        Ratemultiplier   3
        Topology         4
        Brlens           5
        Clockrate        6
        ------------------

        1 --  Parameter  = Alpha_symdir
              Type       = Symmetric diricihlet/beta distribution alpha_i parameter
              Prior      = Symmetric dirichlet with fixed(-1.00) variance parameter
```

```
   2 --  Parameter  = Alpha
         Type       = Shape of scaled gamma distribution of site rates
         Prior      = Uniform(0.00,200.00)

   3 --  Parameter  = Ratemultiplier
         Type       = Partition-specific rate multiplier
         Prior      = Fixed(1.0)

   4 --  Parameter  = Tau
         Type       = Topology
         Prior      = Prior on topologies obeys constraints
         Subparam.  = V

   5 --  Parameter  = V
         Type       = Branch lengths
         Prior      = Clock:Uniform
                        Tree age has an Exponential(1.000) distribution
                        Node ages are not constrained

   6 --  Parameter  = Clockrate
         Type       = Base rate of clock
         Prior      = Fixed(1.000000)
                        The clock rate is constant (strict clock)


Number of taxa = 40
Number of characters = 116

The MCMC sampler will use the following moves:
   With prob.  Chain will use move
      2.38 %   Multiplier(Alpha)
     11.90 %   ExtSprClock(Tau,V)
     23.81 %   NNIClock(Tau,V)
     11.90 %   ParsSPRClock(Tau,V)
     47.62 %   NodesliderClock(V)
      2.38 %   TreeStretch(V)

Division 1 has 81 unique site patterns
Initializing conditional likelihoods
Using standard non-SSE likelihood calculator for division 1 (single-precision)

Initial log likelihoods and log prior probs:
   Chain 1 -- -1680.778432 -- -40.364893
   Chain 2 -- -2231.300002 -- -144.822360
   Chain 3 -- -2285.777987 -- -202.533686
   Chain 4 -- -2300.315466 -- -201.910542


Starting stepping-stone sampling to estimate marginal likelihood.
50 steps will be used with 1500 generations (3 samples) within each step.
Total of 76500 generations (153 samples) will be collected while first
1500 generations (3 samples) will be discarded as initial burnin.
Additionally at the begining of each step 0 generations (0 samples)
will be discarded as burnin.
Sampling from posterior to prior, i.e. first step samples from close to
posterior.
```

```
   There are results from a previous run saved using the same filename(s).
   Do you want to overwrite these results? (yes/no): n

   Please specify a different file name before running the mcmc analysis.
      You can do that using 'mcmc filename=<name>'. You can also move or
      rename the old result files.
   Error preparing print files
   Error in command "Ss"

MrBayes > ss

   Running Markov chain
   MCMC stamp = 2928313836
   Seed = 1721729764
   Swapseed = 370168243
   Model settings:

      Data not partitioned --
         Datatype  = Standard
         Coding    = Variable
         # States  = Variable, up to 10
                     State frequencies are fixed to be equal
         Rates     = Gamma
                     Gamma shape parameter is uniformly dist-
                     ributed on the interval (0.00,200.00).
                     Gamma distribution is approximated using 4 categories.
                     Likelihood summarized over all rate categories in each generation.

   Active parameters:

      Parameters
      ------------------
      Statefreq        1
      Shape            2
      Ratemultiplier   3
      Topology         4
      Brlens           5
      Clockrate        6
      ------------------

      1 --  Parameter  = Alpha_symdir
            Type       = Symmetric diricihlet/beta distribution alpha_i parameter
            Prior      = Symmetric dirichlet with fixed(-1.00) variance parameter

      2 --  Parameter  = Alpha
            Type       = Shape of scaled gamma distribution of site rates
            Prior      = Uniform(0.00,200.00)

      3 --  Parameter  = Ratemultiplier
            Type       = Partition-specific rate multiplier
            Prior      = Fixed(1.0)

      4 --  Parameter  = Tau
            Type       = Topology
            Prior      = Prior on topologies obeys constraints
            Subparam.  = V
```

```
   5 --  Parameter  = V
         Type       = Branch lengths
         Prior      = Clock:Uniform
                      Tree age has an Exponential(1.000) distribution
                      Node ages are not constrained

   6 --  Parameter  = Clockrate
         Type       = Base rate of clock
         Prior      = Fixed(1.000000)
                      The clock rate is constant (strict clock)


Number of taxa = 40
Number of characters = 116

The MCMC sampler will use the following moves:
   With prob.  Chain will use move
      2.38 %   Multiplier(Alpha)
     11.90 %   ExtSprClock(Tau,V)
     23.81 %   NNIClock(Tau,V)
     11.90 %   ParsSPRClock(Tau,V)
     47.62 %   NodesliderClock(V)
      2.38 %   TreeStretch(V)

Division 1 has 81 unique site patterns
Initializing conditional likelihoods
Using standard non-SSE likelihood calculator for division 1 (single-precision)

Initial log likelihoods and log prior probs:
   Chain 1 -- -1680.778432 -- -40.364893
   Chain 2 -- -2231.300002 -- -144.822360
   Chain 3 -- -2285.777987 -- -202.533686
   Chain 4 -- -2300.315466 -- -201.910542


Starting stepping-stone sampling to estimate marginal likelihood.
50 steps will be used with 1500 generations (3 samples) within each step.
Total of 76500 generations (153 samples) will be collected while first
1500 generations (3 samples) will be discarded as initial burnin.
Additionally at the begining of each step 0 generations (0 samples)
will be discarded as burnin.
Sampling from posterior to prior, i.e. first step samples from close to
posterior.


Chain results (76500 generations requested):

    0 -- [-1680.778] (-2231.300) (-2285.778) (-2300.315)

Sampling step 1 out of 50 steps...

Sampling step 2 out of 50 steps...

Sampling step 3 out of 50 steps...

Sampling step 4 out of 50 steps...

Sampling step 5 out of 50 steps...
```

```
Sampling step 6 out of 50 steps...

10000 -- [-1044.196] (-1021.140) (-1043.047) (-1032.717) -- 0:00:46
Sampling step 7 out of 50 steps...

Sampling step 8 out of 50 steps...

Sampling step 9 out of 50 steps...

Sampling step 10 out of 50 steps...

Sampling step 11 out of 50 steps...

Sampling step 12 out of 50 steps...

Sampling step 13 out of 50 steps...

20000 -- (-1113.119) (-1109.920) [-1067.840] (-1075.641) -- 0:00:36
Sampling step 14 out of 50 steps...

Sampling step 15 out of 50 steps...

Sampling step 16 out of 50 steps...

Sampling step 17 out of 50 steps...

Sampling step 18 out of 50 steps...

Sampling step 19 out of 50 steps...

30000 -- [-1122.192] (-1107.338) (-1337.333) (-1138.509) -- 0:00:29
Sampling step 20 out of 50 steps...

Sampling step 21 out of 50 steps...

Sampling step 22 out of 50 steps...

Sampling step 23 out of 50 steps...

Sampling step 24 out of 50 steps...

Sampling step 25 out of 50 steps...

Sampling step 26 out of 50 steps...

40000 -- (-1311.252) (-1365.492) (-1334.048) [-1205.317] -- 0:00:23
Sampling step 27 out of 50 steps...

Sampling step 28 out of 50 steps...

Sampling step 29 out of 50 steps...

Sampling step 30 out of 50 steps...

Sampling step 31 out of 50 steps...

Sampling step 32 out of 50 steps...
```

```
Sampling step 33 out of 50 steps...

50000 -- (-1361.075) (-1397.984) [-1355.495] (-1411.142) -- 0:00:16
Sampling step 34 out of 50 steps...

Sampling step 35 out of 50 steps...

Sampling step 36 out of 50 steps...

Sampling step 37 out of 50 steps...

Sampling step 38 out of 50 steps...

Sampling step 39 out of 50 steps...

60000 -- [-1412.985] (-1420.651) (-1454.691) (-1428.179) -- 0:00:10
Sampling step 40 out of 50 steps...

Sampling step 41 out of 50 steps...

Sampling step 42 out of 50 steps...

Sampling step 43 out of 50 steps...

Sampling step 44 out of 50 steps...

Sampling step 45 out of 50 steps...

Sampling step 46 out of 50 steps...

70000 -- [-1462.462] (-2239.231) (-2297.458) (-2235.560) -- 0:00:04
Sampling step 47 out of 50 steps...

Sampling step 48 out of 50 steps...

Sampling step 49 out of 50 steps...

Sampling step 50 out of 50 steps...


Analysis completed in 49 seconds
Analysis used 48.09 seconds of CPU time
Log likelihood of best state for "cold" chain was -994.86

Marginal likelihood (in natural log units) estimated using stepping-stone sampling based on
50 steps with 1500 generations (3 samples) within each step.

    Run   Marginal likelihood (ln)
    ------------------------------
      1     -1117.61
    ------------------------------
More statistics on stepping-stone sampling is dumped to YND-Typ-Hout-YnotinND-ss.ss file.

Plot of average standard deviation of split frequencies across steps.
Points at -1.0 (y-axis) indicate that there were no splits
above minimum frequency for corresponding step.
+------------------------------------------------------------+ 0.000
```

```
      |                                                            |
      |                                                            |
      |                                                            |
      |                                                            |
      |                                                            |
      |                                                            |
      |                                                            |
      |                                                            |
      |                                                            |
      |                                                            |
      |                                                            |
      |                                                            |
      |                                                            |
      |                                                            |
      |***** **** ***** **** ***** **** ***** **** ***** **** *****|
      +------+-----+-----+-----+-----+-----+-----+-----+-----+-----+ 0.000
      ^                                                            ^
      1                                                            50


      Acceptance rates for the moves in the "cold" chain:
         With prob.   (last 100)   chain accepted proposals by move
            43.4 %     ( 51 %)     Multiplier(Alpha)
            43.2 %     ( 54 %)     ExtSprClock(Tau,V)
            78.5 %     (100 %)     NNIClock(Tau,V)
            33.4 %     ( 35 %)     ParsSPRClock(Tau,V)
            85.8 %     ( 97 %)     NodesliderClock(V)
            55.5 %     ( 87 %)     TreeStretch(V)

      Chain swap information:

                  1      2      3      4
           ------------------------------
         1 |          0.20   0.05   0.01
         2 |  12695          0.33   0.08
         3 |  12671  12460          0.36
         4 |  12990  12941  12743

      Upper diagonal: Proportion of successful state exchanges between chains
      Lower diagonal: Number of attempted state exchanges between chains

      Chain information:

        ID -- Heat
       -----------
         1 -- 1.00  (cold chain)
         2 -- 0.91
         3 -- 0.83
         4 -- 0.77

      Heat = 1 / (1 + T * (ID - 1))
         (where T = 0.10 is the temperature and ID is the chain number)


   MrBayes > ss

      Running Markov chain
      MCMC stamp = 6626483590
```

```
Seed = 1467775986
Swapseed = 171795935
Model settings:

   Data not partitioned --
      Datatype  = Standard
      Coding    = Variable
      # States  = Variable, up to 10
                  State frequencies are fixed to be equal
      Rates     = Gamma
                  Gamma shape parameter is uniformly dist-
                  ributed on the interval (0.00,200.00).
                  Gamma distribution is approximated using 4 categories.
                  Likelihood summarized over all rate categories in each generation.

Active parameters:

   Parameters
   ------------------
   Statefreq        1
   Shape            2
   Ratemultiplier   3
   Topology         4
   Brlens           5
   Clockrate        6
   ------------------

   1 --  Parameter  = Alpha_symdir
         Type       = Symmetric diricihlet/beta distribution alpha_i parameter
         Prior      = Symmetric dirichlet with fixed(-1.00) variance parameter

   2 --  Parameter  = Alpha
         Type       = Shape of scaled gamma distribution of site rates
         Prior      = Uniform(0.00,200.00)

   3 --  Parameter  = Ratemultiplier
         Type       = Partition-specific rate multiplier
         Prior      = Fixed(1.0)

   4 --  Parameter  = Tau
         Type       = Topology
         Prior      = Prior on topologies obeys constraints
         Subparam.  = V

   5 --  Parameter  = V
         Type       = Branch lengths
         Prior      = Clock:Uniform
                      Tree age has an Exponential(1.000) distribution
                      Node ages are not constrained

   6 --  Parameter  = Clockrate
         Type       = Base rate of clock
         Prior      = Fixed(1.000000)
                      The clock rate is constant (strict clock)


Number of taxa = 40
Number of characters = 116
```

```
The MCMC sampler will use the following moves:
   With prob.  Chain will use move
      2.38 %   Multiplier(Alpha)
     11.90 %   ExtSprClock(Tau,V)
     23.81 %   NNIClock(Tau,V)
     11.90 %   ParsSPRClock(Tau,V)
     47.62 %   NodesliderClock(V)
      2.38 %   TreeStretch(V)

Division 1 has 81 unique site patterns
Initializing conditional likelihoods
Using standard non-SSE likelihood calculator for division 1 (single-precision)

Initial log likelihoods and log prior probs:
   Chain 1 -- -1620.166632 -- -3.810897
   Chain 2 -- -2300.560644 -- -166.199913
   Chain 3 -- -2156.594090 -- -129.082657
   Chain 4 -- -2293.230285 -- -186.740403


Starting stepping-stone sampling to estimate marginal likelihood.
50 steps will be used with 1500 generations (3 samples) within each step.
Total of 76500 generations (153 samples) will be collected while first
1500 generations (3 samples) will be discarded as initial burnin.
Additionally at the begining of each step 0 generations (0 samples)
will be discarded as burnin.
Sampling from posterior to prior, i.e. first step samples from close to
posterior.


Chain results (76500 generations requested):

    0 -- [-1620.167] (-2300.561) (-2156.594) (-2293.230)

Sampling step 1 out of 50 steps...

Sampling step 2 out of 50 steps...

Sampling step 3 out of 50 steps...

Sampling step 4 out of 50 steps...

Sampling step 5 out of 50 steps...

Sampling step 6 out of 50 steps...

10000 -- (-1031.570) (-1031.802) [-1018.638] (-1030.281) -- 0:00:39
Sampling step 7 out of 50 steps...

Sampling step 8 out of 50 steps...

Sampling step 9 out of 50 steps...

Sampling step 10 out of 50 steps...

Sampling step 11 out of 50 steps...
```

```
Sampling step 12 out of 50 steps...

Sampling step 13 out of 50 steps...

20000 -- (-1088.182) (-1143.689) (-1100.753) [-1091.231] -- 0:00:36
Sampling step 14 out of 50 steps...

Sampling step 15 out of 50 steps...

Sampling step 16 out of 50 steps...

Sampling step 17 out of 50 steps...

Sampling step 18 out of 50 steps...

Sampling step 19 out of 50 steps...

30000 -- (-1158.099) (-1140.219) (-1239.290) [-1143.877] -- 0:00:29
Sampling step 20 out of 50 steps...

Sampling step 21 out of 50 steps...

Sampling step 22 out of 50 steps...

Sampling step 23 out of 50 steps...

Sampling step 24 out of 50 steps...

Sampling step 25 out of 50 steps...

Sampling step 26 out of 50 steps...

40000 -- (-1374.730) [-1266.760] (-1340.195) (-1343.404) -- 0:00:22
Sampling step 27 out of 50 steps...

Sampling step 28 out of 50 steps...

Sampling step 29 out of 50 steps...

Sampling step 30 out of 50 steps...

Sampling step 31 out of 50 steps...

Sampling step 32 out of 50 steps...

Sampling step 33 out of 50 steps...

50000 -- [-1335.118] (-1420.152) (-1393.019) (-1430.997) -- 0:00:16
Sampling step 34 out of 50 steps...

Sampling step 35 out of 50 steps...

Sampling step 36 out of 50 steps...

Sampling step 37 out of 50 steps...

Sampling step 38 out of 50 steps...
```

```
Sampling step 39 out of 50 steps...

60000 -- [-1466.984] (-1352.105) (-1466.098) (-1425.234) -- 0:00:10
Sampling step 40 out of 50 steps...

Sampling step 41 out of 50 steps...

Sampling step 42 out of 50 steps...

Sampling step 43 out of 50 steps...

Sampling step 44 out of 50 steps...

Sampling step 45 out of 50 steps...

Sampling step 46 out of 50 steps...

70000 -- (-2221.032) (-2280.157) [-1945.455] (-2305.220) -- 0:00:04
Sampling step 47 out of 50 steps...

Sampling step 48 out of 50 steps...

Sampling step 49 out of 50 steps...

Sampling step 50 out of 50 steps...


Analysis completed in 48 seconds
Analysis used 47.53 seconds of CPU time
Log likelihood of best state for "cold" chain was -990.81

Marginal likelihood (in natural log units) estimated using stepping-stone sampling based on
50 steps with 1500 generations (3 samples) within each step.

    Run   Marginal likelihood (ln)
    ------------------------------
      1     -1118.19
    ------------------------------
More statistics on stepping-stone sampling is dumped to YND-Typ-Hout-YnotinND-ss.ss file.

Plot of average standard deviation of split frequencies across steps.
Points at -1.0 (y-axis) indicate that there were no splits
above minimum frequency for corresponding step.
+------------------------------------------------------------+ 0.000
|                                                            |
|                                                            |
|                                                            |
|                                                            |
|                                                            |
|                                                            |
|                                                            |
|                                                            |
|                                                            |
|                                                            |
|                                                            |
|                                                            |
|                                                            |
|                                                            |
```

```
   |***** **** ***** **** ***** **** ***** **** ***** **** *****|
   +------+-----+-----+-----+-----+-----+-----+-----+-----+-----+ 0.000
   ^                                                            ^
   1                                                            50


   Acceptance rates for the moves in the "cold" chain:
      With prob.   (last 100)   chain accepted proposals by move
         41.9 %     ( 50 %)     Multiplier(Alpha)
         42.4 %     ( 59 %)     ExtSprClock(Tau,V)
         77.5 %     (100 %)     NNIClock(Tau,V)
         33.5 %     ( 35 %)     ParsSPRClock(Tau,V)
         85.7 %     ( 95 %)     NodesliderClock(V)
         53.1 %     ( 85 %)     TreeStretch(V)

   Chain swap information:

                1      2      3      4
        ------------------------------
      1 |          0.21   0.05   0.02
      2 |  12607          0.31   0.10
      3 |  12680  12749          0.41
      4 |  12875  12718  12871

   Upper diagonal: Proportion of successful state exchanges between chains
   Lower diagonal: Number of attempted state exchanges between chains

   Chain information:

     ID -- Heat
    -----------
      1 -- 1.00  (cold chain)
      2 -- 0.91
      3 -- 0.83
      4 -- 0.77

   Heat = 1 / (1 + T * (ID - 1))
      (where T = 0.10 is the temperature and ID is the chain number)


MrBayes >
```

```
Last login: Thu Dec 26 10:15:42 on ttys000
msicoli-m1:~ msicoli$ mb


                            MrBayes v3.2.1 x64

                      (Bayesian Analysis of Phylogeny)

              Distributed under the GNU General Public License


               Type "help" or "help <command>" for information
                     on the commands that are available.

                   Type "about" for authorship and general
                       information about the program.


MrBayes > exe /Users/msicoli/DY-Typlogical_binary_bayes-2.nex

   Executing file "/Users/msicoli/DY-Typlogical_binary_bayes-2.nex"
   UNIX line termination
   Longest line length = 123
   Parsing file
   Expecting NEXUS formatted file
   Reading data block
      Allocated taxon set
      Allocated matrix
      Defining new matrix with 40 taxa and 116 characters
      Data is Standard
      Missing data coded as ?
      Gaps coded as -
      Data matrix is not interleaved
      Taxon  1 -> hax
      Taxon  2 -> gwi
      Taxon  3 -> dgr
      Taxon  4 -> scsh
      Taxon  5 -> xsl
      Taxon  6 -> bea
      Taxon  7 -> crx
      Taxon  8 -> chp
      Taxon  9 -> txc
      Taxon 10 -> haa
      Taxon 11 -> ing
      Taxon 12 -> kuu
      Taxon 13 -> hoi
      Taxon 14 -> koy
      Taxon 15 -> taa
      Taxon 16 -> aht
      Taxon 17 -> tfn
      Taxon 18 -> kkz
      Taxon 19 -> tcb
      Taxon 20 -> tau
      Taxon 21 -> ttmN
      Taxon 22 -> tceS
      Taxon 23 -> eya
```

```
      Taxon 24 -> tli
      Taxon 25 -> gce
      Taxon 26 -> tol
      Taxon 27 -> cco
      Taxon 28 -> hup
      Taxon 29 -> mtl
      Taxon 30 -> wlk
      Taxon 31 -> kto
      Taxon 32 -> apc
      Taxon 33 -> apw
      Taxon 34 -> apj
      Taxon 35 -> nav
      Taxon 36 -> apk
      Taxon 37 -> apl
      Taxon 38 -> srs
      Taxon 39 -> ket
      Taxon 40 -> zko
      Successfully read matrix
      Setting default partition (does not divide up characters)
      Setting model defaults
      Seed (for generating default start values) = 1388074477
      Adding dummy characters (unobserved site patterns) for division 1
      WARNING: There are 26 characters incompatible with the specified
               coding bias. These characters will be excluded.
      Setting output file names to "/Users/msicoli/DY-Typlogical_binary_bayes-2.nex.run<i>.<p|
t>"
   Exiting data block
   Reached end of file

MrBayes > lset nst=6 rates=gamma

   Setting Rates to Gamma
   Successfully set likelihood model parameters
   Adding dummy characters (unobserved site patterns) for division 1
   WARNING: There are 26 characters incompatible with the specified
            coding bias. These characters will be excluded.

MrBayes > prset brlenspr=clock:uniform

   Setting Brlenspr to Clock:Uniform
   Successfully set prior model parameters
   Adding dummy characters (unobserved site patterns) for division 1
   WARNING: There are 26 characters incompatible with the specified
            coding bias. These characters will be excluded.

MrBayes > outgroup 1

   Setting outgroup to taxon "hax"

MrBayes > mcmcp ngen=2000000 printfreq=10000 samplefreq=500 nruns=1 nchains=4 savebrlens=yes
filename=DY-26Dec-strict-Hout2

   Setting number of generations to 2000000
   Setting print frequency to 10000
   Setting sample frequency to 500
   Setting number of runs to 1
```

```
   Setting number of chains to 4
   Setting chain output file names to "DY-26Dec-strict-Hout2.<p/t>"
   Successfully set chain parameters

MrBayes > mcmc

   Running Markov chain
   MCMC stamp = 5449400826
   Seed = 1134458959
   Swapseed = 1388074477
   Model settings:

      Data not partitioned --
         Datatype  = Standard
         Coding    = Variable
         # States  = Variable, up to 10
                     State frequencies are fixed to be equal
         Rates     = Gamma
                     Gamma shape parameter is uniformly dist-
                     ributed on the interval (0.00,200.00).
                     Gamma distribution is approximated using 4 categories.
                     Likelihood summarized over all rate categories in each generation.

   Active parameters:

      Parameters
      ------------------
      Statefreq        1
      Shape            2
      Ratemultiplier   3
      Topology         4
      Brlens           5
      Clockrate        6
      ------------------

      1 --  Parameter  = Alpha_symdir
            Type       = Symmetric diricihlet/beta distribution alpha_i parameter
            Prior      = Symmetric dirichlet with fixed(-1.00) variance parameter

      2 --  Parameter  = Alpha
            Type       = Shape of scaled gamma distribution of site rates
            Prior      = Uniform(0.00,200.00)

      3 --  Parameter  = Ratemultiplier
            Type       = Partition-specific rate multiplier
            Prior      = Fixed(1.0)

      4 --  Parameter  = Tau
            Type       = Topology
            Prior      = All topologies equally probable a priori
            Subparam.  = V

      5 --  Parameter  = V
            Type       = Branch lengths
            Prior      = Clock:Uniform
                         Tree age has an Exponential(1.000) distribution
```

```
                      Node ages are not constrained

   6 --  Parameter  = Clockrate
         Type       = Base rate of clock
         Prior      = Fixed(1.000000)
                      The clock rate is constant (strict clock)


Number of taxa = 40
Number of characters = 116

The MCMC sampler will use the following moves:
   With prob.  Chain will use move
      2.38 %   Multiplier(Alpha)
     11.90 %   ExtSprClock(Tau,V)
     23.81 %   NNIClock(Tau,V)
     11.90 %   ParsSPRClock(Tau,V)
     47.62 %   NodesliderClock(V)
      2.38 %   TreeStretch(V)

Division 1 has 81 unique site patterns
Initializing conditional likelihoods
Using standard non-SSE likelihood calculator for division 1 (single-precision)

Initial log likelihoods and log prior probs:
   Chain 1 -- -1722.625492 -- -93.249778
   Chain 2 -- -1718.765922 -- -93.249778
   Chain 3 -- -1763.021428 -- -93.249778
   Chain 4 -- -1699.399305 -- -93.249778


Chain results (2000000 generations requested):

    0 -- [-1722.625] (-1718.766) (-1763.021) (-1699.399)
10000 -- (-1014.497) [-1017.643] (-1026.649) (-1008.923) -- 0:19:54
20000 -- (-1023.607) [-1013.409] (-1018.200) (-1008.476) -- 0:18:09
30000 -- [-1009.691] (-1014.904) (-1004.985) (-1006.906) -- 0:17:30
40000 -- (-1000.719) [-1010.092] (-1032.720) (-1021.775) -- 0:17:58
50000 -- (-1007.770) (-1020.555) [-1002.901] (-1038.949) -- 0:17:33
60000 -- [-1020.223] (-1009.007) (-1025.374) (-1003.929) -- 0:17:14
70000 -- (-1015.731) [-1009.247] (-1022.190) (-1022.128) -- 0:17:27
80000 -- (-1039.822) [-1021.826] (-1035.211) (-1000.508) -- 0:17:12
90000 -- (-1004.443) (-1013.859) (-1030.251) [-1020.982] -- 0:16:58
100000 -- (-1018.126) [-1004.591] (-1039.582) (-1022.379) -- 0:16:47
110000 -- (-1017.186) [-1009.560] (-1012.467) (-1005.066) -- 0:16:36
120000 -- (-1019.255) (-1025.618) [-1017.007] (-1016.738) -- 0:16:42
130000 -- [-1006.890] (-1028.668) (-1031.914) (-1004.633) -- 0:16:32
140000 -- (-1010.654) (-1015.918) [-1017.700] (-1013.347) -- 0:16:23
150000 -- (-1010.203) [-1006.789] (-1017.817) (-1018.112) -- 0:16:14
160000 -- [-1015.614] (-1019.639) (-1006.314) (-1025.479) -- 0:16:17
170000 -- (-1008.275) (-1017.001) [-1015.255] (-1016.792) -- 0:16:08
180000 -- (-1011.301) (-1012.343) [-1001.411] (-997.635) -- 0:16:00
190000 -- (-1037.016) (-1049.374) (-1018.325) [-1007.851] -- 0:16:02
200000 -- (-1023.995) (-1010.542) [-1000.898] (-1025.257) -- 0:15:54
210000 -- (-1014.727) (-1008.522) [-1001.171] (-1016.649) -- 0:15:46
220000 -- (-1018.374) (-1008.238) [-1005.388] (-1020.628) -- 0:15:46
```

```
230000 -- (-1014.622) [-1011.231] (-1009.556) (-1025.645) -- 0:15:38
240000 -- (-1021.399) (-1013.098) (-1021.066) [-1002.608] -- 0:15:31
250000 -- [-1009.901] (-1016.430) (-1015.178) (-1019.061) -- 0:15:24
260000 -- [-1001.343] (-1018.803) (-1012.355) (-1012.520) -- 0:15:16
270000 -- (-1025.207) (-1023.010) [-1006.626] (-995.819) -- 0:15:16
280000 -- (-1014.451) (-1021.193) (-1021.774) [-1014.267] -- 0:15:09
290000 -- (-1011.260) (-1005.571) [-1000.168] (-1010.005) -- 0:15:02
300000 -- (-1019.810) (-1014.776) [-1017.279] (-1028.504) -- 0:14:55
310000 -- [-1016.670] (-1012.246) (-1004.450) (-1008.476) -- 0:14:48
320000 -- (-1012.119) (-1024.311) [-1002.453] (-1026.813) -- 0:14:47
330000 -- (-1002.951) (-1011.099) [-1007.507] (-1004.218) -- 0:14:40
340000 -- (-1018.460) [-1000.701] (-1019.192) (-1015.460) -- 0:14:33
350000 -- (-1030.001) (-1016.537) (-1030.786) [-1020.008] -- 0:14:27
360000 -- (-1014.125) (-1025.268) (-1015.349) [-997.903] -- 0:14:21
370000 -- (-1006.544) (-1011.025) (-1010.609) [-1006.273] -- 0:14:19
380000 -- (-1011.312) (-1012.179) (-1009.748) [-1009.483] -- 0:14:12
390000 -- (-1008.134) (-1000.728) (-1038.844) [-998.146] -- 0:14:06
400000 -- (-1008.622) [-1009.413] (-1031.074) (-1005.924) -- 0:14:00
410000 -- [-1017.732] (-1008.041) (-1008.604) (-1026.424) -- 0:13:57
420000 -- [-1009.748] (-1043.303) (-1032.086) (-1008.092) -- 0:13:55
430000 -- [-1008.585] (-1024.107) (-1023.771) (-1009.831) -- 0:13:48
440000 -- (-994.937) [-995.373] (-1023.578) (-1028.488) -- 0:13:42
450000 -- (-1022.909) [-1029.937] (-1027.270) (-1005.861) -- 0:13:36
460000 -- (-1010.687) [-1008.994] (-1011.370) (-1034.851) -- 0:13:33
470000 -- (-1015.857) [-993.718] (-1041.442) (-1034.086) -- 0:13:27
480000 -- (-1013.218) [-1007.604] (-1038.061) (-1043.153) -- 0:13:21
490000 -- (-1012.241) [-1009.545] (-1029.787) (-1012.800) -- 0:13:18
500000 -- (-1033.317) [-1005.032] (-1011.921) (-1024.422) -- 0:13:12
510000 -- (-1025.198) [-1003.453] (-1019.197) (-1006.196) -- 0:13:05
520000 -- (-1010.933) (-1033.139) (-1023.603) [-1013.213] -- 0:12:59
530000 -- [-1005.216] (-1010.108) (-1019.806) (-1026.298) -- 0:12:53
540000 -- (-1019.579) [-1000.290] (-1019.684) (-1035.709) -- 0:12:50
550000 -- (-1017.249) [-1019.785] (-1024.485) (-1009.594) -- 0:12:44
560000 -- (-1005.997) (-1017.492) (-1039.417) [-1008.990] -- 0:12:38
570000 -- (-1027.129) (-1024.355) [-1013.148] (-1009.800) -- 0:12:32
580000 -- (-1037.588) (-1009.415) (-1015.059) [-1004.082] -- 0:12:29
590000 -- [-1019.528] (-1028.921) (-1020.814) (-1014.146) -- 0:12:23
600000 -- (-1017.486) (-1010.667) (-1013.812) [-1002.857] -- 0:12:17
610000 -- (-1019.989) (-1015.176) (-1030.724) [-1024.469] -- 0:12:11
620000 -- (-1019.025) (-1019.801) (-1013.647) [-992.001] -- 0:12:07
630000 -- (-1003.820) (-1035.340) (-1009.398) [-1003.366] -- 0:12:01
640000 -- (-1007.256) (-1029.314) (-1034.223) [-1019.918] -- 0:11:56
650000 -- (-1002.209) (-1012.760) (-1022.167) [-1012.788] -- 0:11:50
660000 -- (-1017.339) (-1015.947) (-1006.872) [-1016.495] -- 0:11:46
670000 -- (-1012.967) (-1024.409) [-1009.490] (-1007.301) -- 0:11:40
680000 -- (-1014.410) [-1013.084] (-1020.691) (-1021.829) -- 0:11:34
690000 -- (-1013.685) (-1013.432) (-1015.364) [-1027.233] -- 0:11:29
700000 -- (-1012.444) (-1016.996) [-1007.776] (-1016.672) -- 0:11:25
710000 -- (-1018.351) (-1016.554) (-1019.551) [-1013.677] -- 0:11:19
720000 -- (-1010.182) (-1024.488) (-1017.271) [-1005.163] -- 0:11:13
730000 -- [-1019.983] (-1015.347) (-1013.144) (-1027.756) -- 0:11:09
740000 -- [-1012.556] (-1021.014) (-1013.157) (-1010.041) -- 0:11:04
750000 -- [-1002.333] (-1042.517) (-1026.245) (-1002.412) -- 0:10:58
760000 -- (-1014.582) [-1013.034] (-1041.131) (-1005.686) -- 0:10:52
770000 -- (-1039.470) (-1004.469) (-1030.238) [-1007.420] -- 0:10:48
780000 -- (-1009.201) (-1037.511) (-1025.141) [-1009.637] -- 0:10:42
```

```
790000 -- (-1016.466) (-1018.118) [-1003.285] (-1029.998) -- 0:10:38
800000 -- (-1014.863) (-1009.890) [-1005.414] (-1005.042) -- 0:10:33
810000 -- [-1025.364] (-1027.859) (-1027.980) (-1020.011) -- 0:10:28
820000 -- [-1014.305] (-1014.964) (-1019.369) (-1002.666) -- 0:10:24
830000 -- (-1014.827) (-1008.108) [-1002.654] (-1016.085) -- 0:10:20
840000 -- (-1029.498) (-1012.174) [-1006.895] (-1024.971) -- 0:10:15
850000 -- (-1013.892) (-1016.781) (-1016.324) [-1009.748] -- 0:10:11
860000 -- (-1029.290) [-1003.779] (-1007.581) (-1016.285) -- 0:10:08
870000 -- (-1024.128) [-1005.391] (-1025.886) (-1010.730) -- 0:10:03
880000 -- (-1013.029) (-1009.380) [-1012.462] (-1009.819) -- 0:10:02
890000 -- (-1026.780) (-1004.365) [-1011.945] (-1015.154) -- 0:09:59
900000 -- (-1020.583) (-1024.687) (-1012.239) [-1028.622] -- 0:09:56
910000 -- (-1024.604) (-1014.801) [-1007.021] (-1013.390) -- 0:09:51
920000 -- (-1012.418) (-1015.645) [-1009.110] (-1035.222) -- 0:09:46
930000 -- (-1019.296) [-1008.446] (-1022.754) (-1017.964) -- 0:09:42
940000 -- [-1027.978] (-1021.167) (-1028.275) (-1011.273) -- 0:09:38
950000 -- [-1002.200] (-1020.174) (-1020.285) (-1023.198) -- 0:09:33
960000 -- (-1017.045) (-1011.769) [-996.005] (-996.040) -- 0:09:28
970000 -- (-1012.987) (-1016.354) (-1015.832) [-1006.497] -- 0:09:24
980000 -- (-1019.631) (-1009.587) (-1005.708) [-1020.112] -- 0:09:22
990000 -- (-1031.461) [-1003.500] (-1015.792) (-1019.421) -- 0:09:16
1000000 -- (-1006.903) [-999.303] (-1025.471) (-1017.260) -- 0:09:11
1010000 -- (-1002.770) (-1015.160) [-1004.507] (-1011.023) -- 0:09:05
1020000 -- (-1024.943) [-1008.650] (-1019.406) (-1013.561) -- 0:09:01
1030000 -- [-1011.039] (-1021.357) (-1024.012) (-1002.341) -- 0:08:56
1040000 -- [-998.166] (-1037.070) (-1019.062) (-1015.013) -- 0:08:50
1050000 -- (-1024.994) (-1018.720) [-1012.092] (-1007.999) -- 0:08:45
1060000 -- (-1017.209) (-1025.473) (-1011.543) [-998.992] -- 0:08:39
1070000 -- (-1001.184) (-1031.488) (-1022.035) [-1011.884] -- 0:08:34
1080000 -- [-999.890] (-1017.439) (-1006.941) (-1024.010) -- 0:08:28
1090000 -- (-1021.220) (-1016.798) [-1004.127] (-1010.098) -- 0:08:23
1100000 -- (-1028.519) (-1015.129) (-1017.016) [-1006.862] -- 0:08:19
1110000 -- [-1008.586] (-1021.477) (-1007.312) (-1015.056) -- 0:08:13
1120000 -- (-1022.880) (-1022.586) [-1016.760] (-1014.455) -- 0:08:10
1130000 -- (-1022.089) (-1011.151) (-1013.911) [-1007.817] -- 0:08:04
1140000 -- (-1011.862) (-1023.743) (-1023.192) [-1007.247] -- 0:07:59
1150000 -- (-1008.716) (-1013.480) [-1015.629] (-1017.107) -- 0:07:53
1160000 -- (-1026.803) [-1003.091] (-1022.967) (-1027.048) -- 0:07:47
1170000 -- (-1036.550) [-1010.462] (-1018.270) (-1002.477) -- 0:07:41
1180000 -- (-1035.019) (-1009.291) [-1011.227] (-1017.696) -- 0:07:35
1190000 -- (-1003.703) (-1021.246) [-992.147] (-1022.614) -- 0:07:31
1200000 -- [-1006.231] (-1010.943) (-1024.686) (-1006.088) -- 0:07:27
1210000 -- (-1014.149) [-1006.125] (-1014.461) (-1021.700) -- 0:07:23
1220000 -- (-1013.580) (-1011.293) [-1010.426] (-1013.179) -- 0:07:18
1230000 -- (-1017.776) [-993.012] (-1017.211) (-1019.230) -- 0:07:13
1240000 -- (-1003.576) (-1019.999) (-1023.753) [-1002.404] -- 0:07:08
1250000 -- (-1014.268) (-1038.050) [-1004.300] (-1003.180) -- 0:07:03
1260000 -- (-1012.634) (-1004.616) [-1006.035] (-1045.469) -- 0:06:57
1270000 -- (-1020.266) (-1017.449) [-1019.072] (-1028.054) -- 0:06:52
1280000 -- (-1028.537) [-1003.297] (-1039.640) (-1002.714) -- 0:06:46
1290000 -- (-1016.104) (-1015.200) [-1003.932] (-1016.838) -- 0:06:41
1300000 -- (-1015.873) (-1037.335) (-1006.258) [-1009.561] -- 0:06:35
1310000 -- (-1023.971) (-1009.974) [-1000.289] (-1015.560) -- 0:06:30
1320000 -- (-1033.882) [-1003.444] (-1009.753) (-1023.020) -- 0:06:24
1330000 -- (-1033.917) [-1014.760] (-1008.434) (-1039.305) -- 0:06:19
1340000 -- (-1006.140) [-1014.717] (-1017.292) (-1012.027) -- 0:06:13
```

```
1350000 -- (-1011.184) (-1015.369) [-1002.331] (-997.588) -- 0:06:08
1360000 -- (-1026.054) (-1015.572) [-1009.717] (-1021.713) -- 0:06:02
1370000 -- [-1007.505] (-1016.394) (-1017.961) (-1007.594) -- 0:05:57
1380000 -- (-1022.354) (-1006.005) [-1013.648] (-1017.233) -- 0:05:51
1390000 -- (-1008.898) (-1015.117) (-1006.989) [-1007.117] -- 0:05:47
1400000 -- (-1008.579) (-1015.636) [-1007.892] (-1030.924) -- 0:05:41
1410000 -- (-1012.460) [-1009.506] (-1012.407) (-1016.835) -- 0:05:36
1420000 -- [-1019.580] (-1016.090) (-1013.985) (-1011.427) -- 0:05:30
1430000 -- [-1006.685] (-1017.280) (-1011.769) (-1010.057) -- 0:05:25
1440000 -- (-1016.079) (-1014.731) (-1004.142) [-994.172] -- 0:05:19
1450000 -- [-1012.352] (-1031.939) (-1020.686) (-1012.811) -- 0:05:14
1460000 -- (-1012.028) (-1024.041) (-1019.289) [-1006.290] -- 0:05:08
1470000 -- (-1019.205) [-1009.648] (-1018.238) (-1008.048) -- 0:05:03
1480000 -- (-1018.225) [-1012.728] (-1029.428) (-1000.139) -- 0:04:57
1490000 -- (-1014.965) [-1009.722] (-1025.361) (-1030.039) -- 0:04:52
1500000 -- (-1033.445) (-1038.528) (-1016.186) [-1003.708] -- 0:04:46
1510000 -- (-1007.107) (-1013.430) [-1018.213] (-1007.367) -- 0:04:41
1520000 -- [-1002.590] (-1026.793) (-1008.335) (-1010.402) -- 0:04:35
1530000 -- (-1024.057) [-1004.905] (-1015.341) (-1009.141) -- 0:04:29
1540000 -- [-1005.775] (-1020.795) (-1016.775) (-998.706) -- 0:04:24
1550000 -- (-1011.082) (-1023.640) [-1001.394] (-1019.481) -- 0:04:18
1560000 -- (-1020.186) (-1053.092) [-1017.984] (-1013.622) -- 0:04:12
1570000 -- (-1009.287) (-1029.065) [-1016.064] (-1012.643) -- 0:04:07
1580000 -- [-1008.630] (-1017.484) (-1002.180) (-1017.557) -- 0:04:01
1590000 -- [-1010.918] (-1032.854) (-1018.106) (-1003.309) -- 0:03:55
1600000 -- (-1033.674) (-1024.527) [-1015.395] (-998.339) -- 0:03:50
1610000 -- (-1011.190) [-1015.395] (-1041.313) (-1008.084) -- 0:03:44
1620000 -- [-1005.289] (-1023.030) (-1006.672) (-1022.442) -- 0:03:38
1630000 -- (-1027.760) [-1012.656] (-1035.150) (-1006.086) -- 0:03:33
1640000 -- (-1006.304) [-1010.373] (-1005.114) (-1028.728) -- 0:03:27
1650000 -- (-1007.852) (-1021.388) [-1005.000] (-1022.408) -- 0:03:21
1660000 -- (-1006.677) (-1021.786) [-1013.038] (-1019.849) -- 0:03:16
1670000 -- (-1007.426) [-1003.562] (-1015.111) (-1024.981) -- 0:03:10
1680000 -- [-1008.550] (-1019.964) (-1021.046) (-1019.105) -- 0:03:04
1690000 -- (-1027.676) [-1027.736] (-1029.223) (-1014.990) -- 0:02:59
1700000 -- [-1004.450] (-1020.914) (-1017.062) (-1009.281) -- 0:02:53
1710000 -- (-1011.764) (-1005.248) (-1005.678) [-996.778] -- 0:02:47
1720000 -- [-1005.713] (-1026.591) (-1005.158) (-1013.104) -- 0:02:41
1730000 -- [-1014.851] (-1022.463) (-1001.089) (-1021.421) -- 0:02:36
1740000 -- (-1017.310) (-1036.472) [-1001.289] (-1014.162) -- 0:02:30
1750000 -- [-1020.465] (-1008.300) (-1015.938) (-1019.812) -- 0:02:24
1760000 -- (-1017.193) [-1001.525] (-1028.825) (-1019.178) -- 0:02:18
1770000 -- (-1023.535) [-999.780] (-1013.175) (-1017.314) -- 0:02:12
1780000 -- (-1039.640) (-1019.189) [-1017.200] (-1012.867) -- 0:02:06
1790000 -- (-1013.331) [-1008.762] (-1004.943) (-1032.596) -- 0:02:01
1800000 -- (-1022.281) [-1002.268] (-1013.067) (-1009.214) -- 0:01:55
1810000 -- (-1013.634) [-1019.998] (-1008.382) (-1016.517) -- 0:01:49
1820000 -- (-1009.430) [-1007.118] (-1043.366) (-1031.810) -- 0:01:43
1830000 -- (-1023.136) (-1003.273) [-1005.204] (-1005.465) -- 0:01:37
1840000 -- (-1031.059) [-1002.310] (-1019.906) (-1016.444) -- 0:01:32
1850000 -- (-1022.153) [-1006.872] (-1015.255) (-1019.787) -- 0:01:26
1860000 -- [-1004.272] (-1029.433) (-1012.534) (-1002.912) -- 0:01:20
1870000 -- (-1034.550) (-1018.303) [-1002.689] (-1011.167) -- 0:01:14
1880000 -- (-1023.759) (-1019.318) [-995.984] (-1013.254) -- 0:01:08
1890000 -- [-1002.934] (-1012.205) (-1013.271) (-1006.149) -- 0:01:03
1900000 -- [-1009.840] (-1022.766) (-1023.288) (-1028.966) -- 0:00:57
```

```
   1910000 -- (-1008.129) (-1026.136) [-1006.252] (-1012.397) -- 0:00:51
   1920000 -- (-1006.634) (-1026.334) [-1007.913] (-1007.945) -- 0:00:45
   1930000 -- (-1011.529) (-1015.872) (-1021.593) [-1014.711] -- 0:00:40
   1940000 -- [-1013.537] (-1027.924) (-1007.607) (-1033.956) -- 0:00:34
   1950000 -- (-1011.710) [-1006.317] (-1028.062) (-1005.585) -- 0:00:28
   1960000 -- (-1007.740) (-1023.011) (-1034.228) [-995.187] -- 0:00:22
   1970000 -- (-1003.515) (-1010.789) (-1018.642) [-1008.914] -- 0:00:17
   1980000 -- [-1007.155] (-1017.143) (-1034.538) (-1008.662) -- 0:00:11
   1990000 -- [-1018.314] (-1012.037) (-1021.902) (-1016.872) -- 0:00:05
   2000000 -- (-1007.355) [-1001.127] (-1009.912) (-1020.594) -- 0:00:00

   Continue with analysis? (yes/no): n

   Analysis completed in 19 mins 5 seconds
   Analysis used 1054.63 seconds of CPU time
   Log likelihood of best state for "cold" chain was -985.06

   Acceptance rates for the moves in the "cold" chain:
      With prob.   (last 100)   chain accepted proposals by move
         34.3 %     ( 16 %)     Multiplier(Alpha)
         19.0 %     ( 26 %)     ExtSprClock(Tau,V)
         44.9 %     ( 35 %)     NNIClock(Tau,V)
         15.6 %     ( 16 %)     ParsSPRClock(Tau,V)
         70.3 %     ( 74 %)     NodesliderClock(V)
         71.0 %     ( 32 %)     TreeStretch(V)

   Chain swap information:

                1       2       3       4
        ----------------------------------
      1 |            0.45    0.19    0.07
      2 |  332602            0.56    0.27
      3 |  334113  333671            0.60
      4 |  333117  332681  333816

   Upper diagonal: Proportion of successful state exchanges between chains
   Lower diagonal: Number of attempted state exchanges between chains

   Chain information:

     ID -- Heat
    -----------
      1 -- 1.00  (cold chain)
      2 -- 0.91
      3 -- 0.83
      4 -- 0.77

   Heat = 1 / (1 + T * (ID - 1))
      (where T = 0.10 is the temperature and ID is the chain number)


MrBayes > sumt relburnin=yes

   Using relative burnin (a fraction of samples discarded).
   Summarizing trees in file "DY-26Dec-strict-Hout2.t"
   Using relative burnin ('relburnin=yes'), discarding the first 25 % of sampled trees
```

```
Writing statistics to files DY-26Dec-strict-Hout2.<parts|tstat|vstat|trprobs|con>
Examining file ...
Found one tree block in file "DY-26Dec-strict-Hout2.t" with 4001 trees in last block

Tree reading status:

0      10      20      30      40      50      60      70      80      90     100
v-------v-------v-------v-------v-------v-------v-------v-------v-------v-------v
*********************************************************************************

Read 4001 trees from last tree block (sampling 3001 of them)

General explanation:

In an unrooted tree, a taxon bipartition (split) is specified by removing a
branch, thereby dividing the species into those to the left and those to the
right of the branch. Here, taxa to one side of the removed branch are denoted
'.' and those to the other side are denoted '*'. Specifically, the '.' symbol
is used for the taxa on the same side as the outgroup.

In a rooted or clock tree, the tree is rooted using the model and not by
reference to an outgroup. Each bipartition therefore corresponds to a clade,
that is, a group that includes all the descendants of a particular branch in
the tree.  Taxa that are included in each clade are denoted using '*', and
taxa that are not included are denoted using the '.' symbol.

The output first includes a key to all the bipartitions with frequency larger
or equual to (Minpartfreq) in at least one run. Minpartfreq is a paramiter to
sumt command and currently it is set to 0.10.  This is followed by a table
with statistics for the informative bipartitions (those including at least
two taxa), sorted from highest to lowest probability. For each bipartition,
the table gives the number of times the partition or split was observed in all
runs (#obs) and the posterior probability of the bipartition (Probab.), which
is the same as the split frequency. If several runs are summarized, this is
followed by the minimum split frequency (Min(s)), the maximum frequency
(Max(s)), and the standard deviation of frequencies (Stddev(s)) across runs.
The latter value should approach 0 for all bipartitions as MCMC runs converge.

This is followed by a table summarizing branch lengths, node heights (if a
clock model was used) and relaxed clock parameters (if a relaxed clock model
was used). The mean, variance, and 95 % credible interval are given for each
of these parameters. If several runs are summarized, the potential scale
reduction factor (PSRF) is also given; it should approach 1 as runs converge.
Node heights will take calibration points into account, if such points were
used in the analysis.

Note that Stddev may be unreliable if the partition is not present in all
runs (the last column indicates the number of runs that sampled the partition
if more than one run is summarized). The PSRF is not calculated at all if
the partition is not present in all runs.The PSRF is also sensitive to small
sample sizes and it should only be considered a rough guide to convergence
since some of the assumptions allowing one to interpret it as a true potential
scale reduction factor are violated in MrBayes.

List of taxa in bipartitions:
```

```
   1 -- hax
   2 -- gwi
   3 -- dgr
   4 -- scsh
   5 -- xsl
   6 -- bea
   7 -- crx
   8 -- chp
   9 -- txc
  10 -- haa
  11 -- ing
  12 -- kuu
  13 -- hoi
  14 -- koy
  15 -- taa
  16 -- aht
  17 -- tfn
  18 -- kkz
  19 -- tcb
  20 -- tau
  21 -- ttmN
  22 -- tceS
  23 -- eya
  24 -- tli
  25 -- gce
  26 -- tol
  27 -- cco
  28 -- hup
  29 -- mtl
  30 -- wlk
  31 -- kto
  32 -- apc
  33 -- apw
  34 -- apj
  35 -- nav
  36 -- apk
  37 -- apl
  38 -- srs
  39 -- ket
  40 -- zko

Key to taxon bipartitions (saved to file "DY-26Dec-strict-Hout2.parts"):

 ID -- Partition
-----------------------------------------------
  0 -- ****************************************
  1 -- *.......................................
  2 -- .*......................................
  3 -- ..*.....................................
  4 -- ...*....................................
  5 -- ....*...................................
  6 -- .....*..................................
  7 -- ......*.................................
  8 -- .......*................................
  9 -- ........*...............................
 10 -- .........*..............................
```

```
11 -- ..........*.............................
12 -- ...........*............................
13 -- ............*...........................
14 -- .............*..........................
15 -- ..............*.........................
16 -- ...............*........................
17 -- ................*.......................
18 -- .................*......................
19 -- ..................*.....................
20 -- ...................*....................
21 -- ....................*...................
22 -- .....................*..................
23 -- ......................*.................
24 -- .......................*................
25 -- ........................*...............
26 -- .........................*..............
27 -- ..........................*.............
28 -- ...........................*............
29 -- ............................*...........
30 -- .............................*..........
31 -- ..............................*.........
32 -- ...............................*........
33 -- ................................*.......
34 -- .................................*......
35 -- ..................................*.....
36 -- ...................................*....
37 -- ....................................*...
38 -- .....................................*..
39 -- ......................................*.
40 -- .......................................*
41 -- ......................................**
42 -- ...................................**...
43 -- ...............................*******..
44 -- ...............................****.....
45 -- ..............*...**....................
46 -- .............................**.........
47 -- ..........*.**..........................
48 -- ...........................****.........
49 -- ...............................******...
50 -- .*********************..***....*******..
51 -- .***.**.................................
52 -- ...............................***......
53 -- ....*..*.*....*..*****..................
54 -- ............................***.........
55 -- ....*............*......................
56 -- ...............................**.......
57 -- ............**..........................
58 -- ......................**................
59 -- .....**.................................
60 -- ....*..*.*.......*..**..................
61 -- ..............*...*.....................
62 -- ........................***.............
63 -- .......*............**..................
64 -- .*******.*....*..*****..................
65 -- ........................**..............
66 -- ....................**..................
```

```
 67 -- .........................**.............
 68 -- .**.....................................
 69 -- *.....................**................
 70 -- ..........****..........................
 71 -- ....*..*.**.***..*****..................
 72 -- ..................**....................
 73 -- ..**....................................
 74 -- .*********************..................
 75 -- ...........................****.......**
 76 -- ........................***....*******..
 77 -- ...*..*.................................
 78 -- ...*.**.................................
 79 -- ...............**.......................
 80 -- *.....................*.................
 81 -- ..........*.*...........................
 82 -- **************************************..
 83 -- ....*....*.......*......................
 84 -- ...........................*.**.........
 85 -- ..........*.***...**....................
 86 -- .*********************.........*******..
 87 -- .......*.............*..................
 88 -- .*.*.**.................................
 89 -- ...............................*.*......
 90 -- ................................**......
 91 -- .*...**.................................
 92 -- .**************************....*******..
 93 -- .***..*.................................
 94 -- .*******.**.***..*****..................
 95 -- .......*............*...................
 96 -- ....*..*.........*..**..................
 97 -- ........*.......*.......................
 98 -- .*********************..**************..
 99 -- ...................................***..
100 -- *.....................**...****.......**
101 -- .*******.*....*..*****.........*******..
102 -- ........*..*............................
103 -- ...............*...............*******..
104 -- ..............*....*....................
105 -- .***....................................
106 -- ***************************....*******..
107 -- *..........................****.........
108 -- ....*...............*...................
109 -- *..........................****.......**
110 -- ...........*....*.......................
111 -- ........*......**.......................
112 -- ...............................**.*.....
113 -- ........*......*........................
114 -- .........*....*...**....................
115 -- ..........*..*..........................
116 -- .......*.*..........**..................
117 -- ........*..*....*.......................
118 -- ........*..*...**.......................
119 -- ..........****............*.............
-----------------------------------------------
```

Summary statistics for informative taxon bipartitions (clades)

(saved to file "DY-26Dec-strict-Hout2.tstat"):

ID	#obs	Probab.
41	3001	1.000000
42	2997	0.998667
43	2776	0.925025
44	2616	0.871709
45	2564	0.854382
46	2527	0.842053
47	2441	0.813396
48	2250	0.749750
49	2241	0.746751
50	2238	0.745751
51	2118	0.705765
52	2043	0.680773
53	1984	0.661113
54	1836	0.611796
55	1826	0.608464
56	1789	0.596135
57	1760	0.586471
58	1758	0.585805
59	1695	0.564812
60	1588	0.529157
61	1586	0.528491
62	1583	0.527491
63	1572	0.523825
64	1448	0.482506
65	1406	0.468510
66	1289	0.429523
67	1202	0.400533
68	1170	0.389870
69	1067	0.355548
70	1066	0.355215
71	1053	0.350883
72	941	0.313562
73	931	0.310230
74	924	0.307897
75	921	0.306898
76	880	0.293236
77	806	0.268577
78	801	0.266911
79	783	0.260913
80	731	0.243585
81	730	0.243252
82	660	0.219927
83	658	0.219260
84	654	0.217927
85	637	0.212263
86	580	0.193269
87	580	0.193269
88	555	0.184938
89	555	0.184938
90	555	0.184938
91	552	0.183939
92	551	0.183605

93	529	0.176275
94	520	0.173276
95	518	0.172609
96	506	0.168610
97	503	0.167611
98	487	0.162279
99	460	0.153282
100	456	0.151949
101	443	0.147617
102	430	0.143286
103	412	0.137288
104	410	0.136621
105	397	0.132289
106	385	0.128291
107	377	0.125625
108	373	0.124292
109	369	0.122959
110	368	0.122626
111	356	0.118627
112	356	0.118627
113	355	0.118294
114	352	0.117294
115	350	0.116628
116	335	0.111629
117	333	0.110963
118	310	0.103299
119	304	0.101300

Summary statistics for branch and node parameters
(saved to file "DY-26Dec-strict-Hout2.vstat"):

			95% HPD Interval		
Parameter	Mean	Variance	Lower	Upper	Median
length[1]	0.114690	0.001561	0.046758	0.189851	0.112184
length[2]	0.037719	0.000299	0.009372	0.071696	0.034830
length[3]	0.038457	0.000364	0.009583	0.079175	0.034806
length[4]	0.033569	0.000275	0.007822	0.065881	0.030545
length[5]	0.026235	0.000179	0.004924	0.052110	0.023775
length[6]	0.033752	0.000470	0.003749	0.078995	0.028476
length[7]	0.025887	0.000182	0.004309	0.051382	0.023730
length[8]	0.034227	0.000205	0.010211	0.062754	0.032086
length[9]	0.077158	0.000804	0.027178	0.132055	0.075157
length[10]	0.037659	0.000207	0.012679	0.065648	0.036050
length[11]	0.038082	0.000362	0.007724	0.074951	0.034214
length[12]	0.062372	0.000733	0.014161	0.113485	0.059574
length[13]	0.027903	0.000186	0.005500	0.054491	0.025951
length[14]	0.028521	0.000198	0.006827	0.057731	0.026316
length[15]	0.021910	0.000167	0.002753	0.047922	0.019282
length[16]	0.076915	0.000842	0.026595	0.131616	0.072972
length[17]	0.072553	0.000779	0.026308	0.127393	0.069223
length[18]	0.028093	0.000201	0.005710	0.056587	0.025475
length[19]	0.019343	0.000128	0.002314	0.041904	0.016640
length[20]	0.023942	0.000180	0.004196	0.051066	0.021459

length[21]	0.026523	0.000150	0.006277	0.050705	0.024866
length[22]	0.028194	0.000166	0.006379	0.053605	0.026544
length[23]	0.089763	0.001185	0.028842	0.155279	0.084992
length[24]	0.097726	0.001500	0.032100	0.169992	0.092044
length[25]	0.065260	0.000779	0.019843	0.120791	0.061391
length[26]	0.055730	0.000580	0.015464	0.100974	0.051400
length[27]	0.063306	0.000726	0.018758	0.115970	0.058863
length[28]	0.081304	0.001142	0.024979	0.147505	0.076382
length[29]	0.064301	0.000801	0.016244	0.116733	0.060134
length[30]	0.034788	0.000324	0.006890	0.068988	0.031184
length[31]	0.035213	0.000354	0.006197	0.071030	0.031235
length[32]	0.006951	0.000043	0.000009	0.020028	0.004990
length[33]	0.006800	0.000040	0.000009	0.019794	0.004962
length[34]	0.011398	0.000064	0.000352	0.026904	0.009724
length[35]	0.022444	0.000132	0.005222	0.045708	0.020115
length[36]	0.004342	0.000022	0.000000	0.013039	0.002807
length[37]	0.004345	0.000023	0.000000	0.013039	0.002807
length[38]	0.051479	0.000481	0.014390	0.093336	0.048531
length[39]	0.059751	0.000504	0.022647	0.103790	0.057580
length[40]	0.059751	0.000504	0.022647	0.103790	0.057580
length[41]	0.070885	0.000721	0.025591	0.122416	0.067885
length[42]	0.030203	0.000211	0.006118	0.059235	0.028214
length[43]	0.035030	0.000369	0.002052	0.072378	0.032108
length[44]	0.015472	0.000122	0.000267	0.037439	0.012855
length[45]	0.021505	0.000167	0.000419	0.045781	0.018992
length[46]	0.030395	0.000409	0.000094	0.069053	0.026526
length[47]	0.024819	0.000283	0.000465	0.060492	0.021054
length[48]	0.041246	0.000626	0.000054	0.087436	0.037724
length[49]	0.021246	0.000239	0.000097	0.050454	0.018013
length[50]	0.021798	0.000172	0.000910	0.046751	0.019361
length[51]	0.021060	0.000245	0.000023	0.051507	0.017708
length[52]	0.012227	0.000093	0.000005	0.031174	0.010038
length[53]	0.016983	0.000104	0.000048	0.036030	0.015111
length[54]	0.032612	0.000527	0.000060	0.076564	0.027509
length[55]	0.015789	0.000111	0.000004	0.036111	0.013879
length[56]	0.009842	0.000062	0.000005	0.025727	0.007865
length[57]	0.015909	0.000148	0.000041	0.039271	0.013159
length[58]	0.040801	0.000650	0.000190	0.089050	0.037102
length[59]	0.021012	0.000230	0.000007	0.049674	0.018149
length[60]	0.014023	0.000094	0.000093	0.032587	0.012137
length[61]	0.010117	0.000071	0.000005	0.026398	0.007941
length[62]	0.028288	0.000347	0.000063	0.062623	0.024674
length[63]	0.009801	0.000050	0.000051	0.023250	0.008030
length[64]	0.023673	0.000194	0.001482	0.051344	0.021431
length[65]	0.028735	0.000408	0.000005	0.066771	0.024481
length[66]	0.010704	0.000077	0.000003	0.027257	0.008682
length[67]	0.028777	0.000400	0.000086	0.068514	0.025368
length[68]	0.018480	0.000166	0.000468	0.043841	0.015597
length[69]	0.021364	0.000231	0.000039	0.049665	0.018736
length[70]	0.026802	0.000354	0.000425	0.065570	0.022725
length[71]	0.019790	0.000115	0.002908	0.041277	0.017915
length[72]	0.011833	0.000123	0.000021	0.033207	0.008391
length[73]	0.017745	0.000152	0.000013	0.042564	0.015125
length[74]	0.012165	0.000083	0.000085	0.030510	0.010059
length[75]	0.015644	0.000112	0.000243	0.034374	0.014219
length[76]	0.017258	0.000156	0.000018	0.041698	0.014182

length[77]	0.015887	0.000151	0.000005	0.039879	0.012950
length[78]	0.015930	0.000168	0.000015	0.040396	0.012838
length[79]	0.022179	0.000282	0.000103	0.052978	0.018689
length[80]	0.029266	0.000479	0.000008	0.070831	0.025023
length[81]	0.009888	0.000091	0.000009	0.027921	0.007263
length[82]	0.009709	0.000090	0.000008	0.029636	0.006715
length[83]	0.007901	0.000058	0.000004	0.023411	0.005605
length[84]	0.022325	0.000382	0.000005	0.061856	0.016491
length[85]	0.015775	0.000154	0.000026	0.039717	0.012779
length[86]	0.012277	0.000105	0.000010	0.030884	0.009944
length[87]	0.006579	0.000043	0.000004	0.019482	0.004637
length[88]	0.016522	0.000134	0.000055	0.038879	0.014046
length[89]	0.005722	0.000029	0.000005	0.016330	0.004049
length[90]	0.006533	0.000042	0.000016	0.020109	0.004647
length[91]	0.014065	0.000161	0.000033	0.038443	0.010886
length[92]	0.011984	0.000080	0.000083	0.029067	0.009958
length[93]	0.015576	0.000158	0.000026	0.041743	0.012752
length[94]	0.013541	0.000120	0.000032	0.034062	0.011075
length[95]	0.006407	0.000041	0.000010	0.018694	0.004690
length[96]	0.006124	0.000037	0.000006	0.017777	0.004591
length[97]	0.017060	0.000228	0.000075	0.044928	0.013024
length[98]	0.013056	0.000122	0.000050	0.034693	0.010835
length[99]	0.012244	0.000131	0.000037	0.034026	0.008806
length[100]	0.007126	0.000041	0.000013	0.018698	0.005381
length[101]	0.014644	0.000109	0.000023	0.035127	0.012685
length[102]	0.019280	0.000253	0.000022	0.052568	0.015649
length[103]	0.019626	0.000186	0.000300	0.046315	0.016683
length[104]	0.006156	0.000038	0.000001	0.018551	0.004179
length[105]	0.010207	0.000130	0.000018	0.031294	0.006259
length[106]	0.007271	0.000046	0.000005	0.020715	0.005171
length[107]	0.017070	0.000157	0.000160	0.041097	0.014082
length[108]	0.014143	0.000093	0.000066	0.031639	0.012650
length[109]	0.009321	0.000073	0.000074	0.026634	0.007065
length[110]	0.020725	0.000345	0.000072	0.060340	0.015990
length[111]	0.014317	0.000141	0.000082	0.038873	0.010755
length[112]	0.004615	0.000027	0.000003	0.015539	0.002859
length[113]	0.016750	0.000203	0.000074	0.045753	0.012514
length[114]	0.008107	0.000054	0.000020	0.021533	0.006378
length[115]	0.006998	0.000049	0.000007	0.020890	0.005173
length[116]	0.005152	0.000033	0.000006	0.014949	0.003368
length[117]	0.017478	0.000217	0.000188	0.048083	0.013133
length[118]	0.013844	0.000123	0.000041	0.036865	0.011630
length[119]	0.025956	0.000306	0.000303	0.060228	0.021317
height[0]	0.143615	0.001725	0.068256	0.212151	0.147314
height[1]	0.000000	0.000000	0.000000	0.000000	0.000000
height[2]	0.000000	0.000000	0.000000	0.000000	0.000000
height[3]	0.000000	0.000000	0.000000	0.000000	0.000000
height[4]	0.000000	0.000000	0.000000	0.000000	0.000000
height[5]	0.000000	0.000000	0.000000	0.000000	0.000000
height[6]	0.000000	0.000000	0.000000	0.000000	0.000000
height[7]	0.000000	0.000000	0.000000	0.000000	0.000000
height[8]	0.000000	0.000000	0.000000	0.000000	0.000000
height[9]	0.000000	0.000000	0.000000	0.000000	0.000000
height[10]	0.000000	0.000000	0.000000	0.000000	0.000000
height[11]	0.000000	0.000000	0.000000	0.000000	0.000000
height[12]	0.000000	0.000000	0.000000	0.000000	0.000000

height[13]	0.000000	0.000000	0.000000	0.000000	0.000000
height[14]	0.000000	0.000000	0.000000	0.000000	0.000000
height[15]	0.000000	0.000000	0.000000	0.000000	0.000000
height[16]	0.000000	0.000000	0.000000	0.000000	0.000000
height[17]	0.000000	0.000000	0.000000	0.000000	0.000000
height[18]	0.000000	0.000000	0.000000	0.000000	0.000000
height[19]	0.000000	0.000000	0.000000	0.000000	0.000000
height[20]	0.000000	0.000000	0.000000	0.000000	0.000000
height[21]	0.000000	0.000000	0.000000	0.000000	0.000000
height[22]	0.000000	0.000000	0.000000	0.000000	0.000000
height[23]	0.000000	0.000000	0.000000	0.000000	0.000000
height[24]	0.000000	0.000000	0.000000	0.000000	0.000000
height[25]	0.000000	0.000000	0.000000	0.000000	0.000000
height[26]	0.000000	0.000000	0.000000	0.000000	0.000000
height[27]	0.000000	0.000000	0.000000	0.000000	0.000000
height[28]	0.000000	0.000000	0.000000	0.000000	0.000000
height[29]	0.000000	0.000000	0.000000	0.000000	0.000000
height[30]	0.000000	0.000000	0.000000	0.000000	0.000000
height[31]	0.000000	0.000000	0.000000	0.000000	0.000000
height[32]	0.000000	0.000000	0.000000	0.000000	0.000000
height[33]	0.000000	0.000000	0.000000	0.000000	0.000000
height[34]	0.000000	0.000000	0.000000	0.000000	0.000000
height[35]	0.000000	0.000000	0.000000	0.000000	0.000000
height[36]	0.000000	0.000000	0.000000	0.000000	0.000000
height[37]	0.000000	0.000000	0.000000	0.000000	0.000000
height[38]	0.000000	0.000000	0.000000	0.000000	0.000000
height[39]	0.000000	0.000000	0.000000	0.000000	0.000000
height[40]	0.000000	0.000000	0.000000	0.000000	0.000000
height[41]	0.059751	0.000504	0.022647	0.103790	0.057580
height[42]	0.004291	0.000020	0.000000	0.012862	0.002799
height[43]	0.052506	0.000391	0.018758	0.089932	0.050783
height[44]	0.022168	0.000101	0.005530	0.042340	0.020352
height[45]	0.025920	0.000152	0.005343	0.049727	0.023884
height[46]	0.031511	0.000216	0.008847	0.061441	0.028986
height[47]	0.038541	0.000274	0.010939	0.071239	0.036168
height[48]	0.082706	0.000911	0.033302	0.141484	0.079900
height[49]	0.034740	0.000195	0.011145	0.062004	0.032974
height[50]	0.112457	0.001029	0.049418	0.164957	0.115739
height[51]	0.053876	0.000355	0.020435	0.087831	0.052835
height[52]	0.011611	0.000050	0.001498	0.025551	0.010211
height[53]	0.051447	0.000285	0.021463	0.083135	0.051502
height[54]	0.058069	0.000506	0.019831	0.101331	0.055134
height[55]	0.022734	0.000119	0.004853	0.044907	0.021000
height[56]	0.004520	0.000020	0.000009	0.012884	0.003269
height[57]	0.024809	0.000144	0.005451	0.048723	0.022736
height[58]	0.083106	0.001006	0.029347	0.144767	0.080010
height[59]	0.022765	0.000135	0.003992	0.044651	0.020975
height[60]	0.043937	0.000200	0.018330	0.070262	0.043529
height[61]	0.017417	0.000093	0.002688	0.037108	0.015173
height[62]	0.073170	0.000635	0.031324	0.123658	0.071956
height[63]	0.032437	0.000149	0.010301	0.054070	0.031486
height[64]	0.069388	0.000484	0.029558	0.107289	0.070605
height[65]	0.054199	0.000498	0.018387	0.098328	0.050695
height[66]	0.023530	0.000124	0.005012	0.045384	0.021884
height[67]	0.051551	0.000426	0.016341	0.090321	0.048828
height[68]	0.032339	0.000208	0.009372	0.061799	0.029714

height[69]	0.117329	0.001185	0.051837	0.177879	0.119372
height[70]	0.059559	0.000531	0.019933	0.103005	0.057675
height[71]	0.064987	0.000409	0.028596	0.103240	0.064787
height[72]	0.018344	0.000108	0.002405	0.039250	0.015915
height[73]	0.031559	0.000209	0.009568	0.062441	0.028934
height[74]	0.100420	0.000788	0.049890	0.151110	0.102589
height[75]	0.121329	0.001180	0.057984	0.181059	0.122126
height[76]	0.093965	0.000743	0.042176	0.141629	0.095349
height[77]	0.024688	0.000126	0.006003	0.046482	0.023070
height[78]	0.037039	0.000253	0.011856	0.067020	0.034917
height[79]	0.065248	0.000592	0.026523	0.113557	0.062119
height[80]	0.094323	0.001050	0.035321	0.150242	0.090415
height[81]	0.028674	0.000170	0.007724	0.053799	0.027195
height[82]	0.137368	0.001792	0.063428	0.209699	0.140613
height[83]	0.035086	0.000162	0.012260	0.057985	0.034426
height[84]	0.061716	0.000575	0.023507	0.111166	0.058239
height[85]	0.050818	0.000299	0.021331	0.084731	0.049046
height[86]	0.101956	0.000898	0.048482	0.155381	0.104448
height[87]	0.028802	0.000128	0.009234	0.049750	0.027249
height[88]	0.041174	0.000260	0.012655	0.071047	0.039911
height[89]	0.006993	0.000023	0.000209	0.016347	0.005874
height[90]	0.006664	0.000023	0.000283	0.016094	0.005388
height[91]	0.038296	0.000224	0.012667	0.066811	0.036454
height[92]	0.122721	0.001408	0.058329	0.187093	0.125378
height[93]	0.046347	0.000231	0.020482	0.077273	0.045994
height[94]	0.077461	0.000595	0.035394	0.121766	0.077012
height[95]	0.028139	0.000126	0.007407	0.049753	0.027115
height[96]	0.041049	0.000196	0.014016	0.063807	0.040304
height[97]	0.068888	0.000580	0.028223	0.115748	0.067094
height[98]	0.131470	0.001418	0.061363	0.190722	0.134013
height[99]	0.035417	0.000207	0.010697	0.063466	0.033324
height[100]	0.138863	0.001472	0.066643	0.203026	0.141218
height[101]	0.086977	0.000820	0.034344	0.131870	0.089189
height[102]	0.060361	0.000560	0.020664	0.106271	0.058869
height[103]	0.070221	0.000646	0.025111	0.114759	0.066478
height[104]	0.020445	0.000117	0.004725	0.044006	0.018918
height[105]	0.043866	0.000293	0.014129	0.074901	0.041152
height[106]	0.135788	0.001348	0.065826	0.198742	0.138454
height[107]	0.112265	0.001471	0.049285	0.179215	0.112184
height[108]	0.021432	0.000107	0.004909	0.040280	0.019817
height[109]	0.130216	0.001417	0.066253	0.199277	0.135479
height[110]	0.060485	0.000631	0.021547	0.108690	0.054666
height[111]	0.081271	0.000648	0.035389	0.123360	0.082046
height[112]	0.016476	0.000057	0.003733	0.031068	0.015177
height[113]	0.069656	0.000547	0.030115	0.113276	0.068482
height[114]	0.038404	0.000191	0.014797	0.064711	0.037013
height[115]	0.029836	0.000154	0.008347	0.052876	0.027295
height[116]	0.038711	0.000174	0.013265	0.061813	0.037877
height[117]	0.071439	0.000719	0.025148	0.115518	0.069818
height[118]	0.079636	0.000640	0.036595	0.131423	0.076217
height[119]	0.067803	0.000607	0.025060	0.112853	0.067232

Clade credibility values:

```
/---------------------------------------------------------------------- hax (1)
|
|                                                          /---------- ket (39)
|----------------------------100---------------------------+
|                                                          \---------- zko (40)
|
|                                      /------------------------------ hup (28)
|                                      |
|------------------75------------------+         /-------------------- mtl (29)
|                                      |         |
|                                      \----61---+         /---------- wlk (30)
|                                                \----84---+
|                                                          \---------- kto (31)
|
|                                                /-------------------- gwi (2)
|                                                |
|                                                |-------------------- dgr (3)
|                                                |
|         /------------------71------------------+-------------------- scsh (4)
|         |                                      |
|         |                                      |         /---------- bea (6)
|         |                                      \----56---+
|         |                                                \---------- crx (7)
|         |
|         |                                                /---------- xsl (5)
|         |                                      /----61---+
|         |                                      |         \---------- kkz (18)
|         |                                      |
|         |                                      |         /---------- chp (8)
|         |                                      |         |
|         |                            /----53---+----52---+---------- ttmN (21)
|         |                            |         |         |
|         |                            |         |         \---------- tceS (22)
|         |                            |         |
|         |                            |         \-------------------- haa (10)
|         |-------------66-------------+
|         |                            |                   /---------- taa (15)
|         |                            |         /----53---+
|         |                            |         |         \---------- tcb (19)
+         |                            \----85---+
|         |                                      \-------------------- tau (20)
|         |
|         |----------------------------------------------------------- txc (9)
|         |
|         |                                      /-------------------- ing (11)
|----75---+                                      |
|         |------------------81------------------+         /---------- hoi (13)
|         |                                      \----59---+
|         |                                                \---------- koy (14)
|         |
|         |----------------------------------------------------------- kuu (12)
|         |
|         |----------------------------------------------------------- aht (16)
|         |
```

```
|         |----------------------------------------------------------- tfn (17)
|         |
|         |                                                /---------- gce (25)
|         |                                                |
|         |-----------------------53-----------------------+---------- tol (26)
|         |                                                |
|         |                                                \---------- cco (27)
|         |
|         |                                                /---------- apc (32)
|         |                                      /----60---+
|         |                                      |         \---------- apw (33)
|         |                            /----68---+
|         |                            |         \-------------------- apj (34)
|         |                   /---87---+
|         |                   |        \------------------------------ nav (35)
|         |         /----75---+
|         |         |         |                            /---------- apk (36)
|         |         |         \-------------100------------+
|         \----93---+                                      \---------- apl (37)
|                   |
|                   \------------------------------------------------- srs (38)
|
|                                                          /---------- eya (23)
\---------------------------59-----------------------------+
                                                           \---------- tli (24)
```

Phylogram (based on median node depths):

```
/--------------------------------------------------------------------- hax (1)
|
|                                         /--------------------------- ket (39)
|-----------------------------------------+
|                                         \--------------------------- zko (40)
|
|                               /------------------------------------- hup (28)
|                               |
|-------------------------------+          /-------------------------- mtl (29)
|                               |          |
|                               \----------+           /-------------- wlk (30)
|                                          \-----------+
|                                                      \-------------- kto (31)
|
|                                           /------------------------- gwi (2)
|                                           |
|                                           |------------------------- dgr (3)
|                                           |
|              /----------------------------+------------------------- scsh (4)
|              |                            |
|              |                            |              /---------- bea (6)
|              |                            \--------------+
|              |                                           \---------- crx (7)
|              |
|              |                                           /---------- xsl (5)
|              |                                 /---------+
|              |                                 |         \---------- kkz (18)
```

```
|              |                                 |
|              |                                 |    /--------------- chp (8)
|              |                                 |    |
|              |                             /---+----+--------------- ttmN (21)
|              |                             |   |    |
|              |                             |   |    \--------------- tceS (22)
|              |                             |   |
|              |                             |   \-------------------- haa (10)
|              |-----------------------------+
|              |                             |                /------- taa (15)
|              |                             |            /---+
|              |                             |            |   \------- tcb (19)
+              |                             \------------+
|              |                                          \----------- tau (20)
|              |
|              |------------------------------------------------------ txc (9)
|              |
|              |                                    /----------------- ing (11)
|--------------+                                    |
|              |------------------------------------+     /----------- hoi (13)
|              |                                    \-----+
|              |                                          \----------- koy (14)
|              |
|              |------------------------------------------------------ kuu (12)
|              |
|              |------------------------------------------------------ aht (16)
|              |
|              |------------------------------------------------------ tfn (17)
|              |
|              |                   /---------------------------------- gce (25)
|              |                   |
|              |-------------------+---------------------------------- tol (26)
|              |                   |
|              |                   \---------------------------------- cco (27)
|              |
|              |                                                   /-- apc (32)
|              |                                                /--+
|              |                                                |  \-- apw (33)
|              |                                           /----+
|              |                                           |    \----- apj (34)
|              |                                      /----+
|              |                                      |    \---------- nav (35)
|              |                             /--------+
|              |                             |        |             /- apk (36)
|              |                             |        \-------------+
|              \-----------------------------+                      \- apl (37)
|                                            |
|                                            \------------------------ srs (38)
|
|                               /------------------------------------- eya (23)
\-------------------------------+
                                \------------------------------------- tli (24)

----|--------|---------|--------|--------|---------|--------|--------|
  0.14     0.12      0.10     0.08     0.06      0.04     0.02     -0.00
```

```
   [Expected changes per site]

   Calculating tree probabilities...

   Credible sets of trees (3001 trees sampled):
      50 % credible set contains 1501 trees
      90 % credible set contains 2701 trees
      95 % credible set contains 2851 trees
      99 % credible set contains 2971 trees


MrBayes > sump relburnin=yes

   Using relative burnin (a fraction of samples discarded).
   Summarizing parameters in file DY-26Dec-strict-Hout2.p
   Writing summary statistics to file DY-26Dec-strict-Hout2.pstat
   Using relative burnin ('relburnin=yes'), discarding the first 25 % of samples

   Below is a rough plot of the generation (x-axis) versus the log
   probability of observing the data (y-axis). You can use this
   graph to determine what the burn in for your analysis should be.
   When the log probability starts to plateau you may be at station-
   arity. Sample trees and parameters after the log probability
   plateaus. Of course, this is not a guarantee that you are at sta-
   analysis should be. When the log probability starts to plateau
   tionarity. When possible, run multiple analyses starting from dif-
   ferent random trees; if the inferences you make for independent
   analyses are the same, this is reasonable evidence that the chains
   have converged. You can use MrBayes to run several independent
   analyses simultaneously. During such a run, MrBayes will monitor
   the convergence of topologies. After the run has been completed,
   the 'sumt' and 'sump' functions will provide additional conver-
   gence diagnostics for all the parameters in your model. Remember
   that the burn in is the number of samples to discard. There are
   a total of ngen / samplefreq samples taken during a MCMC analysis.

   +------------------------------------------------------------+ -1004.822
   |                      *      *                              |
   |           *                                                |
   |            *     *                           *             |
   |  *   *                **                            * *    |
   |                 *       *         *                        |
   |   **        *      *           *    **                   **|
   |                            * **                  *         |
   | *   *                                              *   *   |
   |*        **                *      * *      **   *  *  *     |
   |               **  *             *      *        *          |
   |       *      *                              *              |
   |        *            *    *            *  *                 |
   |                                         *                  |
   |                                               *            |
   |                                                         *  |
   +------+-----+-----+-----+-----+-----+-----+-----+-----+-----+ -1011.995
   ^                                                            ^
   500000                                                      2000000
```

```
   Estimated marginal likelihoods for run sampled in file "DY-26Dec-strict-Hout2.p":
      (Use the harmonic mean for Bayes factor comparisons of models)
      (Values are saved to the file /Users/msicoli/DY-Typlogical_binary_bayes-2.nex.lstat)

   Arithmetic mean   Harmonic mean
   --------------------------------
       -996.18         -1027.76
   --------------------------------


   Model parameter summaries for run sampled in file "DY-26Dec-strict-Hout2":
      Based on a total of 3001 samples out of a total of 4001 samples
         from this analysis.
      Parameter summaries saved to file "/Users/msicoli/DY-
Typlogical_binary_bayes-2.nex.pstat".

                                             95% HPD Interval
                                           --------------------
   Parameter      Mean      Variance      Lower       Upper       Median     ESS*
   ------------------------------------------------------------------------------
   TH          0.143615    0.001725    0.068256    0.212151    0.147314    243.95
   TL          2.439625    0.459764    1.230095    3.583763    2.512555    223.79
   alpha       1.086565    0.816773    0.128740    2.635880    0.896767    413.73
   ------------------------------------------------------------------------------
   * Convergence diagnostic (ESS = Estimated Sample Size); ESS value
     below 100 may indicate that the parameter is undersampled.



MrBayes > ssp ngen=100000 diagnfreq=1000 filename=YND-Typ-Hout2-ss

   Setting number of generations to 100000
   Setting diagnosing frequency to 1000

MrBayes > ss

   Setting chain output file names to "YND-Typ-Hout2-ss.<p/t>"
   Running Markov chain
   MCMC stamp = 6022182788
   Seed = 780705211
   Swapseed = 1290899819
   Model settings:

      Data not partitioned --
         Datatype  = Standard
         Coding    = Variable
         # States  = Variable, up to 10
                     State frequencies are fixed to be equal
         Rates     = Gamma
                     Gamma shape parameter is uniformly dist-
                     ributed on the interval (0.00,200.00).
                     Gamma distribution is approximated using 4 categories.
                     Likelihood summarized over all rate categories in each generation.

   Active parameters:
```

```
   Parameters
   ------------------
   Statefreq        1
   Shape            2
   Ratemultiplier   3
   Topology         4
   Brlens           5
   Clockrate        6
   ------------------

   1 --  Parameter  = Alpha_symdir
         Type       = Symmetric diricihlet/beta distribution alpha_i parameter
         Prior      = Symmetric dirichlet with fixed(-1.00) variance parameter

   2 --  Parameter  = Alpha
         Type       = Shape of scaled gamma distribution of site rates
         Prior      = Uniform(0.00,200.00)

   3 --  Parameter  = Ratemultiplier
         Type       = Partition-specific rate multiplier
         Prior      = Fixed(1.0)

   4 --  Parameter  = Tau
         Type       = Topology
         Prior      = All topologies equally probable a priori
         Subparam.  = V

   5 --  Parameter  = V
         Type       = Branch lengths
         Prior      = Clock:Uniform
                      Tree age has an Exponential(1.000) distribution
                      Node ages are not constrained

   6 --  Parameter  = Clockrate
         Type       = Base rate of clock
         Prior      = Fixed(1.000000)
                      The clock rate is constant (strict clock)


Number of taxa = 40
Number of characters = 116

The MCMC sampler will use the following moves:
   With prob.  Chain will use move
      2.38 %   Multiplier(Alpha)
     11.90 %   ExtSprClock(Tau,V)
     23.81 %   NNIClock(Tau,V)
     11.90 %   ParsSPRClock(Tau,V)
     47.62 %   NodesliderClock(V)
      2.38 %   TreeStretch(V)

Division 1 has 81 unique site patterns
Initializing conditional likelihoods
Using standard non-SSE likelihood calculator for division 1 (single-precision)
```

```
Initial log likelihoods and log prior probs:
   Chain 1 -- -1001.127181 -- -12.625325
   Chain 2 -- -1007.355276 -- -30.294350
   Chain 3 -- -1009.911630 -- -29.733445
   Chain 4 -- -1020.593917 -- -43.326549


Starting stepping-stone sampling to estimate marginal likelihood.
50 steps will be used with 1500 generations (3 samples) within each step.
Total of 76500 generations (153 samples) will be collected while first
1500 generations (3 samples) will be discarded as initial burnin.
Additionally at the begining of each step 0 generations (0 samples)
will be discarded as burnin.
Sampling from posterior to prior, i.e. first step samples from close to
posterior.
NOTE: Number of generation of each step is reduced to the closest multiple
of sampling frequency. That is why, in total it will be taken 76500 gene-
rations instead of requested 100000.


Chain results (76500 generations requested):

    0 -- [-1001.127] (-1007.355) (-1009.912) (-1020.594)

Sampling step 1 out of 50 steps...

Sampling step 2 out of 50 steps...

Sampling step 3 out of 50 steps...

Sampling step 4 out of 50 steps...

Sampling step 5 out of 50 steps...

Sampling step 6 out of 50 steps...

10000 -- (-1052.155) (-1046.580) [-1031.839] (-1040.015) -- 0:00:39
Sampling step 7 out of 50 steps...

Sampling step 8 out of 50 steps...

Sampling step 9 out of 50 steps...

Sampling step 10 out of 50 steps...

Sampling step 11 out of 50 steps...

Sampling step 12 out of 50 steps...

Sampling step 13 out of 50 steps...

20000 -- (-1051.856) (-1100.553) [-1047.227] (-1131.271) -- 0:00:33
Sampling step 14 out of 50 steps...

Sampling step 15 out of 50 steps...
```

```
Sampling step 16 out of 50 steps...

Sampling step 17 out of 50 steps...

Sampling step 18 out of 50 steps...

Sampling step 19 out of 50 steps...

30000 -- (-1296.567) (-1245.607) (-1147.229) [-1142.151] -- 0:00:27
Sampling step 20 out of 50 steps...

Sampling step 21 out of 50 steps...

Sampling step 22 out of 50 steps...

Sampling step 23 out of 50 steps...

Sampling step 24 out of 50 steps...

Sampling step 25 out of 50 steps...

Sampling step 26 out of 50 steps...

40000 -- (-1462.189) [-1320.794] (-1322.051) (-1349.255) -- 0:00:22
Sampling step 27 out of 50 steps...

Sampling step 28 out of 50 steps...

Sampling step 29 out of 50 steps...

Sampling step 30 out of 50 steps...

Sampling step 31 out of 50 steps...

Sampling step 32 out of 50 steps...

Sampling step 33 out of 50 steps...

50000 -- (-1444.965) (-1431.647) [-1346.870] (-1403.867) -- 0:00:16
Sampling step 34 out of 50 steps...

Sampling step 35 out of 50 steps...

Sampling step 36 out of 50 steps...

Sampling step 37 out of 50 steps...

Sampling step 38 out of 50 steps...

Sampling step 39 out of 50 steps...

60000 -- (-1503.649) (-1454.469) [-1457.306] (-1492.975) -- 0:00:10
Sampling step 40 out of 50 steps...

Sampling step 41 out of 50 steps...
```

```
Sampling step 42 out of 50 steps...

Sampling step 43 out of 50 steps...

Sampling step 44 out of 50 steps...

Sampling step 45 out of 50 steps...

Sampling step 46 out of 50 steps...

70000 -- (-2210.742) (-2278.530) [-2175.981] (-2049.667) -- 0:00:04
Sampling step 47 out of 50 steps...

Sampling step 48 out of 50 steps...

Sampling step 49 out of 50 steps...

Sampling step 50 out of 50 steps...


Analysis completed in 48 seconds
Analysis used 44.62 seconds of CPU time
Log likelihood of best state for "cold" chain was -992.96

Marginal likelihood (in natural log units) estimated using stepping-stone sampling based on
50 steps with 1500 generations (3 samples) within each step.

    Run   Marginal likelihood (ln)
    ------------------------------
      1     -1127.56
    ------------------------------
More statistics on stepping-stone sampling is dumped to YND-Typ-Hout2-ss.ss file.

Plot of average standard deviation of split frequencies across steps.
Points at -1.0 (y-axis) indicate that there were no splits
above minimum frequency for corresponding step.
+------------------------------------------------------------+ 0.000
|                                                            |
|                                                            |
|                                                            |
|                                                            |
|                                                            |
|                                                            |
|                                                            |
|                                                            |
|                                                            |
|                                                            |
|                                                            |
|                                                            |
|                                                            |
|                                                            |
|***** **** ***** **** ***** **** ***** **** ***** **** *****|
+------+-----+-----+-----+-----+-----+-----+-----+-----+-----+ 0.000
^                                                            ^
1                                                            50
```

```
   Acceptance rates for the moves in the "cold" chain:
      With prob.   (last 100)   chain accepted proposals by move
         49.8 %     ( 61 %)     Multiplier(Alpha)
         43.2 %     ( 59 %)     ExtSprClock(Tau,V)
         78.4 %     (100 %)     NNIClock(Tau,V)
         33.1 %     ( 29 %)     ParsSPRClock(Tau,V)
         86.1 %     ( 97 %)     NodesliderClock(V)
         54.7 %     ( 85 %)     TreeStretch(V)

   Chain swap information:

                1      2      3      4
        ------------------------------
      1 |          0.21   0.04   0.01
      2 |  12713          0.31   0.08
      3 |  12860  12787          0.36
      4 |  12542  12762  12836

   Upper diagonal: Proportion of successful state exchanges between chains
   Lower diagonal: Number of attempted state exchanges between chains

   Chain information:

     ID -- Heat
    -----------
      1 -- 1.00  (cold chain)
      2 -- 0.91
      3 -- 0.83
      4 -- 0.77

   Heat = 1 / (1 + T * (ID - 1))
      (where T = 0.10 is the temperature and ID is the chain number)


MrBayes > ss

   Running Markov chain
   MCMC stamp = 6548936566
   Seed = 231125092
   Swapseed = 1717525018
   Model settings:

      Data not partitioned --
         Datatype  = Standard
         Coding    = Variable
         # States  = Variable, up to 10
                     State frequencies are fixed to be equal
         Rates     = Gamma
                     Gamma shape parameter is uniformly dist-
                     ributed on the interval (0.00,200.00).
                     Gamma distribution is approximated using 4 categories.
                     Likelihood summarized over all rate categories in each generation.

   Active parameters:
```

```
   Parameters
   ------------------
   Statefreq        1
   Shape            2
   Ratemultiplier   3
   Topology         4
   Brlens           5
   Clockrate        6
   ------------------

   1 --  Parameter  = Alpha_symdir
         Type       = Symmetric diricihlet/beta distribution alpha_i parameter
         Prior      = Symmetric dirichlet with fixed(-1.00) variance parameter

   2 --  Parameter  = Alpha
         Type       = Shape of scaled gamma distribution of site rates
         Prior      = Uniform(0.00,200.00)

   3 --  Parameter  = Ratemultiplier
         Type       = Partition-specific rate multiplier
         Prior      = Fixed(1.0)

   4 --  Parameter  = Tau
         Type       = Topology
         Prior      = All topologies equally probable a priori
         Subparam.  = V

   5 --  Parameter  = V
         Type       = Branch lengths
         Prior      = Clock:Uniform
                      Tree age has an Exponential(1.000) distribution
                      Node ages are not constrained

   6 --  Parameter  = Clockrate
         Type       = Base rate of clock
         Prior      = Fixed(1.000000)
                      The clock rate is constant (strict clock)


Number of taxa = 40
Number of characters = 116

The MCMC sampler will use the following moves:
   With prob.  Chain will use move
      2.38 %   Multiplier(Alpha)
     11.90 %   ExtSprClock(Tau,V)
     23.81 %   NNIClock(Tau,V)
     11.90 %   ParsSPRClock(Tau,V)
     47.62 %   NodesliderClock(V)
      2.38 %   TreeStretch(V)

Division 1 has 81 unique site patterns
Initializing conditional likelihoods
Using standard non-SSE likelihood calculator for division 1 (single-precision)

Initial log likelihoods and log prior probs:
```

```
   Chain 1 -- -1700.896933 -- -70.642544
   Chain 2 -- -2266.130972 -- -171.769429
   Chain 3 -- -2280.289285 -- -222.262241
   Chain 4 -- -2308.118309 -- -203.827352


Starting stepping-stone sampling to estimate marginal likelihood.
50 steps will be used with 1500 generations (3 samples) within each step.
Total of 76500 generations (153 samples) will be collected while first
1500 generations (3 samples) will be discarded as initial burnin.
Additionally at the begining of each step 0 generations (0 samples)
will be discarded as burnin.
Sampling from posterior to prior, i.e. first step samples from close to
posterior.


Chain results (76500 generations requested):

    0 -- [-1700.897] (-2266.131) (-2280.289) (-2308.118)

Sampling step 1 out of 50 steps...

Sampling step 2 out of 50 steps...

Sampling step 3 out of 50 steps...

Sampling step 4 out of 50 steps...

Sampling step 5 out of 50 steps...

Sampling step 6 out of 50 steps...

10000 -- (-1035.938) (-1029.248) [-1010.146] (-1029.447) -- 0:00:39
Sampling step 7 out of 50 steps...

Sampling step 8 out of 50 steps...

Sampling step 9 out of 50 steps...

Sampling step 10 out of 50 steps...

Sampling step 11 out of 50 steps...

Sampling step 12 out of 50 steps...

Sampling step 13 out of 50 steps...

20000 -- (-1128.993) (-1088.452) (-1098.992) [-1056.226] -- 0:00:33
Sampling step 14 out of 50 steps...

Sampling step 15 out of 50 steps...

Sampling step 16 out of 50 steps...

Sampling step 17 out of 50 steps...
```

```
Sampling step 18 out of 50 steps...

Sampling step 19 out of 50 steps...

30000 -- (-1180.920) (-1293.324) [-1152.954] (-1182.539) -- 0:00:26
Sampling step 20 out of 50 steps...

Sampling step 21 out of 50 steps...

Sampling step 22 out of 50 steps...

Sampling step 23 out of 50 steps...

Sampling step 24 out of 50 steps...

Sampling step 25 out of 50 steps...

Sampling step 26 out of 50 steps...

40000 -- [-1228.154] (-1381.908) (-1361.579) (-1382.597) -- 0:00:21
Sampling step 27 out of 50 steps...

Sampling step 28 out of 50 steps...

Sampling step 29 out of 50 steps...

Sampling step 30 out of 50 steps...

Sampling step 31 out of 50 steps...

Sampling step 32 out of 50 steps...

Sampling step 33 out of 50 steps...

50000 -- (-1399.794) (-1424.915) [-1466.781] (-1453.575) -- 0:00:15
Sampling step 34 out of 50 steps...

Sampling step 35 out of 50 steps...

Sampling step 36 out of 50 steps...

Sampling step 37 out of 50 steps...

Sampling step 38 out of 50 steps...

Sampling step 39 out of 50 steps...

60000 -- (-1627.987) (-1471.007) [-1466.831] (-1546.448) -- 0:00:09
Sampling step 40 out of 50 steps...

Sampling step 41 out of 50 steps...

Sampling step 42 out of 50 steps...

Sampling step 43 out of 50 steps...
```

```
Sampling step 44 out of 50 steps...

Sampling step 45 out of 50 steps...

Sampling step 46 out of 50 steps...

70000 -- (-2307.970) [-1665.981] (-2265.110) (-1983.707) -- 0:00:03
Sampling step 47 out of 50 steps...

Sampling step 48 out of 50 steps...

Sampling step 49 out of 50 steps...

Sampling step 50 out of 50 steps...


Analysis completed in 45 seconds
Analysis used 44.36 seconds of CPU time
Log likelihood of best state for "cold" chain was -1003.68

Marginal likelihood (in natural log units) estimated using stepping-stone sampling based on
50 steps with 1500 generations (3 samples) within each step.

    Run   Marginal likelihood (ln)
    ------------------------------
      1     -1127.07
    ------------------------------
More statistics on stepping-stone sampling is dumped to YND-Typ-Hout2-ss.ss file.

Plot of average standard deviation of split frequencies across steps.
Points at -1.0 (y-axis) indicate that there were no splits
above minimum frequency for corresponding step.
+------------------------------------------------------------+ 0.000
|                                                            |
|                                                            |
|                                                            |
|                                                            |
|                                                            |
|                                                            |
|                                                            |
|                                                            |
|                                                            |
|                                                            |
|                                                            |
|                                                            |
|                                                            |
|                                                            |
|***** **** ***** **** ***** **** ***** **** ***** **** *****|
+------+-----+-----+-----+-----+-----+-----+-----+-----+-----+ 0.000
^                                                            ^
1                                                           50


Acceptance rates for the moves in the "cold" chain:
   With prob.   (last 100)   chain accepted proposals by move
      43.8 %     ( 54 %)     Multiplier(Alpha)
```

```
      43.4 %     ( 64 %)     ExtSprClock(Tau,V)
      78.8 %     (100 %)     NNIClock(Tau,V)
      32.9 %     ( 25 %)     ParsSPRClock(Tau,V)
      86.0 %     ( 98 %)     NodesliderClock(V)
      53.6 %     ( 81 %)     TreeStretch(V)

   Chain swap information:

                1      2      3      4
        ------------------------------
      1 |          0.23   0.05   0.01
      2 |  12719          0.30   0.07
      3 |  12663  12921          0.35
      4 |  12905  12609  12683

   Upper diagonal: Proportion of successful state exchanges between chains
   Lower diagonal: Number of attempted state exchanges between chains

   Chain information:

     ID -- Heat
    -----------
      1 -- 1.00  (cold chain)
      2 -- 0.91
      3 -- 0.83
      4 -- 0.77

   Heat = 1 / (1 + T * (ID - 1))
      (where T = 0.10 is the temperature and ID is the chain number)


MrBayes > ss

   Running Markov chain
   MCMC stamp = 6390972662
   Seed = 1735696387
   Swapseed = 147486170
   Model settings:

      Data not partitioned --
         Datatype  = Standard
         Coding    = Variable
         # States  = Variable, up to 10
                     State frequencies are fixed to be equal
         Rates     = Gamma
                     Gamma shape parameter is uniformly dist-
                     ributed on the interval (0.00,200.00).
                     Gamma distribution is approximated using 4 categories.
                     Likelihood summarized over all rate categories in each generation.

   Active parameters:

      Parameters
      ------------------
      Statefreq        1
      Shape            2
```

```
   Ratemultiplier   3
   Topology         4
   Brlens           5
   Clockrate        6
   ------------------

   1 --  Parameter  = Alpha_symdir
         Type       = Symmetric diricihlet/beta distribution alpha_i parameter
         Prior      = Symmetric dirichlet with fixed(-1.00) variance parameter

   2 --  Parameter  = Alpha
         Type       = Shape of scaled gamma distribution of site rates
         Prior      = Uniform(0.00,200.00)

   3 --  Parameter  = Ratemultiplier
         Type       = Partition-specific rate multiplier
         Prior      = Fixed(1.0)

   4 --  Parameter  = Tau
         Type       = Topology
         Prior      = All topologies equally probable a priori
         Subparam.  = V

   5 --  Parameter  = V
         Type       = Branch lengths
         Prior      = Clock:Uniform
                      Tree age has an Exponential(1.000) distribution
                      Node ages are not constrained

   6 --  Parameter  = Clockrate
         Type       = Base rate of clock
         Prior      = Fixed(1.000000)
                      The clock rate is constant (strict clock)


Number of taxa = 40
Number of characters = 116

The MCMC sampler will use the following moves:
   With prob.  Chain will use move
      2.38 %   Multiplier(Alpha)
     11.90 %   ExtSprClock(Tau,V)
     23.81 %   NNIClock(Tau,V)
     11.90 %   ParsSPRClock(Tau,V)
     47.62 %   NodesliderClock(V)
      2.38 %   TreeStretch(V)

Division 1 has 81 unique site patterns
Initializing conditional likelihoods
Using standard non-SSE likelihood calculator for division 1 (single-precision)

Initial log likelihoods and log prior probs:
   Chain 1 -- -1750.544444 -- -81.031598
   Chain 2 -- -2269.986807 -- -189.910093
   Chain 3 -- -2295.411042 -- -191.476033
   Chain 4 -- -2277.901644 -- -199.106113
```

```
Starting stepping-stone sampling to estimate marginal likelihood.
50 steps will be used with 1500 generations (3 samples) within each step.
Total of 76500 generations (153 samples) will be collected while first
1500 generations (3 samples) will be discarded as initial burnin.
Additionally at the begining of each step 0 generations (0 samples)
will be discarded as burnin.
Sampling from posterior to prior, i.e. first step samples from close to
posterior.


Chain results (76500 generations requested):

    0 -- [-1750.544] (-2269.987) (-2295.411) (-2277.902)

Sampling step 1 out of 50 steps...

Sampling step 2 out of 50 steps...

Sampling step 3 out of 50 steps...

Sampling step 4 out of 50 steps...

Sampling step 5 out of 50 steps...

Sampling step 6 out of 50 steps...

10000 -- (-1031.442) [-1016.888] (-1049.916) (-1047.454) -- 0:00:39
Sampling step 7 out of 50 steps...

Sampling step 8 out of 50 steps...

Sampling step 9 out of 50 steps...

Sampling step 10 out of 50 steps...

Sampling step 11 out of 50 steps...

Sampling step 12 out of 50 steps...

Sampling step 13 out of 50 steps...

20000 -- [-1078.900] (-1159.212) (-1080.756) (-1083.329) -- 0:00:36
Sampling step 14 out of 50 steps...

Sampling step 15 out of 50 steps...

Sampling step 16 out of 50 steps...

Sampling step 17 out of 50 steps...

Sampling step 18 out of 50 steps...

Sampling step 19 out of 50 steps...
```

```
30000 -- (-1267.103) [-1118.587] (-1225.274) (-1241.654) -- 0:00:27
Sampling step 20 out of 50 steps...

Sampling step 21 out of 50 steps...

Sampling step 22 out of 50 steps...

Sampling step 23 out of 50 steps...

Sampling step 24 out of 50 steps...

Sampling step 25 out of 50 steps...

Sampling step 26 out of 50 steps...

40000 -- (-1356.317) [-1207.039] (-1354.253) (-1404.918) -- 0:00:21
Sampling step 27 out of 50 steps...

Sampling step 28 out of 50 steps...

Sampling step 29 out of 50 steps...

Sampling step 30 out of 50 steps...

Sampling step 31 out of 50 steps...

Sampling step 32 out of 50 steps...

Sampling step 33 out of 50 steps...

50000 -- (-1444.109) (-1362.327) (-1467.370) [-1339.991] -- 0:00:15
Sampling step 34 out of 50 steps...

Sampling step 35 out of 50 steps...

Sampling step 36 out of 50 steps...

Sampling step 37 out of 50 steps...

Sampling step 38 out of 50 steps...

Sampling step 39 out of 50 steps...

60000 -- (-1506.013) (-1465.573) (-1484.749) [-1449.323] -- 0:00:09
Sampling step 40 out of 50 steps...

Sampling step 41 out of 50 steps...

Sampling step 42 out of 50 steps...

Sampling step 43 out of 50 steps...

Sampling step 44 out of 50 steps...

Sampling step 45 out of 50 steps...
```

```
Sampling step 46 out of 50 steps...

70000 -- (-2300.140) [-1590.175] (-2286.516) (-2258.392) -- 0:00:03
Sampling step 47 out of 50 steps...

Sampling step 48 out of 50 steps...

Sampling step 49 out of 50 steps...

Sampling step 50 out of 50 steps...


Analysis completed in 45 seconds
Analysis used 44.39 seconds of CPU time
Log likelihood of best state for "cold" chain was -1002.80

Marginal likelihood (in natural log units) estimated using stepping-stone sampling based on
50 steps with 1500 generations (3 samples) within each step.

    Run   Marginal likelihood (ln)
    ------------------------------
      1     -1125.48
    ------------------------------
More statistics on stepping-stone sampling is dumped to YND-Typ-Hout2-ss.ss file.

Plot of average standard deviation of split frequencies across steps.
Points at -1.0 (y-axis) indicate that there were no splits
above minimum frequency for corresponding step.
+------------------------------------------------------------+ 0.000
|                                                            |
|                                                            |
|                                                            |
|                                                            |
|                                                            |
|                                                            |
|                                                            |
|                                                            |
|                                                            |
|                                                            |
|                                                            |
|                                                            |
|                                                            |
|                                                            |
|***** **** ***** **** ***** **** ***** **** ***** **** *****|
+------+-----+-----+-----+-----+-----+-----+-----+-----+-----+ 0.000
^                                                            ^
1                                                           50


Acceptance rates for the moves in the "cold" chain:
   With prob.   (last 100)   chain accepted proposals by move
      43.3 %     ( 51 %)     Multiplier(Alpha)
      43.2 %     ( 53 %)     ExtSprClock(Tau,V)
      78.8 %     (100 %)     NNIClock(Tau,V)
      33.0 %     ( 27 %)     ParsSPRClock(Tau,V)
      86.0 %     (100 %)     NodesliderClock(V)
```

```
         52.3 %     ( 91 %)     TreeStretch(V)

   Chain swap information:

                1      2      3      4
         ------------------------------
       1 |          0.18   0.05   0.01
       2 |  12763          0.31   0.08
       3 |  12778  12783          0.37
       4 |  12663  12704  12809

   Upper diagonal: Proportion of successful state exchanges between chains
   Lower diagonal: Number of attempted state exchanges between chains

   Chain information:

      ID -- Heat
     -----------
       1 -- 1.00  (cold chain)
       2 -- 0.91
       3 -- 0.83
       4 -- 0.77

   Heat = 1 / (1 + T * (ID - 1))
      (where T = 0.10 is the temperature and ID is the chain number)


MrBayes >
```
