# Supplementary material for: Linguistic Phylogenies Support Back-Migration from Beringia to Asia
Source: PLoS One. 2014 Mar 12;9(3):e91722. doi: 10.1371/journal.pone.0091722 (PMC3951421; doi:10.1371/journal.pone.0091722)
Supplement: File S4 — MCMC Runs (without Haida Outgroup in matrix). (PDF) [file pone.0091722.s004.pdf]

Sicoli-Holton: Linguistic phylogenies support back-migration from Beringia to Asia

MCMC Runs without Haida Outgroup in matrix

With Na-Dene Ingroup Constraint	2
Without Na-Dene Ingroup Constraint	27
Alternate Yeniseian without Kott and without Ket	53
Alternate Na-Dene without Eyak	84

```
                            MrBayes v3.2.1 x64

                      (Bayesian Analysis of Phylogeny)

              Distributed under the GNU General Public License


               Type "help" or "help <command>" for information
                     on the commands that are available.

                   Type "about" for authorship and general
                       information about the program.


MrBayes > exe /Users/msicoli/Yeniseian-NaDene-Typlogical_noHAX.nex

   Executing file "/Users/msicoli/Yeniseian-NaDene-Typlogical_noHAX.nex"
   UNIX line termination
   Longest line length = 123
   Parsing file
   Expecting NEXUS formatted file
   Reading data block
      Allocated taxon set
      Allocated matrix
      Defining new matrix with 39 taxa and 116 characters
      Data is Standard
      Missing data coded as ?
      Gaps coded as -
      Data matrix is not interleaved
      Taxon  1 -> gwi
      Taxon  2 -> dgr
      Taxon  3 -> scsh
      Taxon  4 -> xsl
      Taxon  5 -> bea
      Taxon  6 -> crx
      Taxon  7 -> chp
      Taxon  8 -> txc
      Taxon  9 -> haa
      Taxon 10 -> ing
      Taxon 11 -> kuu
      Taxon 12 -> hoi
      Taxon 13 -> koy
      Taxon 14 -> taa
      Taxon 15 -> aht
      Taxon 16 -> tfn
      Taxon 17 -> kkz
      Taxon 18 -> tcb
      Taxon 19 -> tau
      Taxon 20 -> ttmN
      Taxon 21 -> tceS
      Taxon 22 -> eya
      Taxon 23 -> tli
      Taxon 24 -> gce
      Taxon 25 -> tol
      Taxon 26 -> cco
      Taxon 27 -> hup
      Taxon 28 -> mtl
```

```
      Taxon 29 -> wlk
      Taxon 30 -> kto
      Taxon 31 -> apc
      Taxon 32 -> apw
      Taxon 33 -> apj
      Taxon 34 -> nav
      Taxon 35 -> apk
      Taxon 36 -> apl
      Taxon 37 -> srs
      Taxon 38 -> ket
      Taxon 39 -> zko
      Successfully read matrix
      Setting default partition (does not divide up characters)
      Setting model defaults
      Seed (for generating default start values) = 1388062882
      Adding dummy characters (unobserved site patterns) for division 1
      WARNING: There are 32 characters incompatible with the specified
               coding bias. These characters will be excluded.
      Setting output file names to "/Users/msicoli/Yeniseian-NaDene-Typlogical_noHAX.nex.run<i>.<p|
t>"
   Exiting data block
   Reached end of file

MrBayes > lset nst=6 rates=gamma

   Setting Rates to Gamma
   Successfully set likelihood model parameters
   Adding dummy characters (unobserved site patterns) for division 1
   WARNING: There are 32 characters incompatible with the specified
            coding bias. These characters will be excluded.

MrBayes > prset brlenspr=clock:uniform

   Setting Brlenspr to Clock:Uniform
   Successfully set prior model parameters
   Adding dummy characters (unobserved site patterns) for division 1
   WARNING: There are 32 characters incompatible with the specified
            coding bias. These characters will be excluded.

MrBayes > constraint ingroup = 1-37

   Defining constraint called 'ingroup'

MrBayes > prset topologypr = constraints(ingroup)

   Setting Topologypr to Constraints
   Successfully set prior model parameters
   Adding dummy characters (unobserved site patterns) for division 1
   WARNING: There are 32 characters incompatible with the specified
            coding bias. These characters will be excluded.

MrBayes > mcmcp ngen=2000000 printfreq=10000 samplefreq=500 nruns=1 nchains=4 savebrlens=yes
filename=DY-26Dec-strict-Yout

   Setting number of generations to 2000000
   Setting print frequency to 10000
   Setting sample frequency to 500
   Setting number of runs to 1
   Setting number of chains to 4
   Setting chain output file names to "DY-26Dec-strict-Yout.<p/t>"
   Successfully set chain parameters
```

```
MrBayes > mcmc

   Running Markov chain
   MCMC stamp = 4319925389
   Seed = 810769782
   Swapseed = 1388062882
   Model settings:

      Data not partitioned --
         Datatype  = Standard
         Coding    = Variable
         # States  = Variable, up to 10
                     State frequencies are fixed to be equal
         Rates     = Gamma
                     Gamma shape parameter is uniformly dist-
                     ributed on the interval (0.00,200.00).
                     Gamma distribution is approximated using 4 categories.
                     Likelihood summarized over all rate categories in each generation.

   Active parameters:

      Parameters
      ------------------
      Statefreq        1
      Shape            2
      Ratemultiplier   3
      Topology         4
      Brlens           5
      Clockrate        6
      ------------------

      1 --  Parameter  = Alpha_symdir
            Type       = Symmetric diricihlet/beta distribution alpha_i parameter
            Prior      = Symmetric dirichlet with fixed(-1.00) variance parameter

      2 --  Parameter  = Alpha
            Type       = Shape of scaled gamma distribution of site rates
            Prior      = Uniform(0.00,200.00)

      3 --  Parameter  = Ratemultiplier
            Type       = Partition-specific rate multiplier
            Prior      = Fixed(1.0)

      4 --  Parameter  = Tau
            Type       = Topology
            Prior      = Prior on topologies obeys constraints
            Subparam.  = V

      5 --  Parameter  = V
            Type       = Branch lengths
            Prior      = Clock:Uniform
                         Tree age has an Exponential(1.000) distribution
                         Node ages are not constrained

      6 --  Parameter  = Clockrate
            Type       = Base rate of clock
            Prior      = Fixed(1.000000)
                         The clock rate is constant (strict clock)
```

```
Number of taxa = 39
Number of characters = 116

The MCMC sampler will use the following moves:
   With prob.  Chain will use move
      2.38 %   Multiplier(Alpha)
     11.90 %   ExtSprClock(Tau,V)
     23.81 %   NNIClock(Tau,V)
     11.90 %   ParsSPRClock(Tau,V)
     47.62 %   NodesliderClock(V)
      2.38 %   TreeStretch(V)

Division 1 has 78 unique site patterns
Initializing conditional likelihoods
Using standard non-SSE likelihood calculator for division 1 (single-precision)

Initial log likelihoods and log prior probs:
   Chain 1 -- -1428.020263 -- -90.228070
   Chain 2 -- -1465.865890 -- -90.228070
   Chain 3 -- -1507.829723 -- -90.228070
   Chain 4 -- -1413.110454 -- -90.228070


Chain results (2000000 generations requested):

    0 -- [-1428.020] (-1465.866) (-1507.830) (-1413.110)
10000 -- (-956.661) [-939.240] (-961.285) (-990.588) -- 0:16:35
20000 -- (-953.753) [-946.552] (-936.552) (-961.812) -- 0:18:09
30000 -- (-939.339) [-947.392] (-949.050) (-951.806) -- 0:19:42
40000 -- [-936.517] (-945.195) (-948.670) (-950.461) -- 0:18:47
50000 -- (-943.559) (-964.183) [-941.975] (-939.836) -- 0:18:51
60000 -- (-939.353) (-953.256) [-942.406] (-945.524) -- 0:18:51
70000 -- (-941.974) (-938.978) [-941.328] (-936.462) -- 0:19:18
80000 -- (-942.158) (-946.194) (-945.313) [-931.431] -- 0:19:12
90000 -- (-955.578) (-948.217) (-939.845) [-937.468] -- 0:18:44
100000 -- (-941.252) (-958.785) (-947.829) [-942.538] -- 0:18:41
110000 -- (-931.237) (-954.540) (-948.089) [-957.763] -- 0:18:36
120000 -- (-935.957) (-960.215) (-966.662) [-942.530] -- 0:18:32
130000 -- (-945.721) (-948.508) [-938.421] (-932.228) -- 0:18:27
140000 -- (-949.544) (-937.795) (-947.729) [-925.346] -- 0:18:22
150000 -- (-951.360) (-942.294) [-943.788] (-939.384) -- 0:18:17
160000 -- (-958.394) (-943.516) [-933.970] (-948.273) -- 0:18:01
170000 -- (-945.436) [-944.757] (-944.179) (-947.381) -- 0:17:56
180000 -- (-946.648) [-937.378] (-942.479) (-952.588) -- 0:17:51
190000 -- (-951.638) (-930.265) [-940.257] (-927.867) -- 0:17:46
200000 -- (-956.614) (-955.130) (-954.071) [-937.399] -- 0:17:42
210000 -- (-952.975) (-957.294) [-935.846] (-952.279) -- 0:17:36
220000 -- (-958.620) (-949.538) (-946.000) [-926.777] -- 0:17:31
230000 -- [-947.648] (-943.248) (-949.371) (-940.165) -- 0:17:34
240000 -- (-956.921) (-961.981) [-932.126] (-947.126) -- 0:17:28
250000 -- (-958.487) (-953.354) (-957.314) [-947.301] -- 0:17:23
260000 -- (-948.357) (-938.603) [-935.215] (-936.103) -- 0:17:24
270000 -- (-941.035) (-944.919) [-941.173] (-952.686) -- 0:17:11
280000 -- (-955.508) (-941.948) [-937.136] (-961.092) -- 0:17:05
290000 -- (-931.322) (-941.578) [-941.169] (-955.148) -- 0:17:00
300000 -- [-936.619] (-944.377) (-943.901) (-958.164) -- 0:16:54
310000 -- (-947.575) [-939.666] (-943.495) (-929.893) -- 0:16:48
320000 -- [-946.062] (-956.364) (-938.728) (-956.746) -- 0:16:42
330000 -- (-962.891) (-939.246) [-936.199] (-947.624) -- 0:16:36
340000 -- (-964.655) (-964.656) (-957.760) [-940.143] -- 0:16:31
350000 -- (-950.653) [-940.270] (-945.548) (-945.307) -- 0:16:29
```

```
360000 -- (-967.134) (-952.410) [-924.421] (-949.378) -- 0:16:23
370000 -- (-946.769) [-948.157] (-953.179) (-934.029) -- 0:16:13
380000 -- (-945.542) (-946.100) [-930.504] (-965.427) -- 0:16:07
390000 -- [-943.409] (-959.099) (-943.216) (-957.242) -- 0:16:05
400000 -- (-951.635) (-942.127) [-938.168] (-957.550) -- 0:15:59
410000 -- (-942.316) (-931.299) (-932.134) [-924.663] -- 0:15:53
420000 -- (-945.186) [-953.568] (-948.481) (-960.233) -- 0:15:44
430000 -- [-947.354] (-926.698) (-950.433) (-945.152) -- 0:15:38
440000 -- (-947.651) (-948.526) [-943.261] (-962.302) -- 0:15:35
450000 -- (-961.279) (-943.161) (-954.033) [-942.448] -- 0:15:29
460000 -- (-965.775) (-954.757) (-964.364) [-946.222] -- 0:15:27
470000 -- (-957.604) [-943.749] (-939.783) (-948.781) -- 0:15:21
480000 -- (-938.665) (-939.128) [-933.938] (-950.623) -- 0:15:15
490000 -- (-941.166) (-940.595) (-949.494) [-943.891] -- 0:15:09
500000 -- (-955.149) (-938.509) [-935.292] (-938.046) -- 0:14:59
510000 -- (-938.704) (-973.313) (-937.718) [-950.574] -- 0:14:53
520000 -- (-952.978) [-934.681] (-945.533) (-956.133) -- 0:14:47
530000 -- (-944.239) (-962.122) (-942.791) [-941.981] -- 0:14:39
540000 -- (-955.511) [-939.510] (-954.834) (-947.752) -- 0:14:33
550000 -- [-930.472] (-936.596) (-949.263) (-947.559) -- 0:14:27
560000 -- (-946.997) (-945.138) (-949.391) [-950.474] -- 0:14:23
570000 -- [-941.057] (-948.909) (-948.288) (-935.630) -- 0:14:17
580000 -- (-956.686) (-958.445) (-965.463) [-941.371] -- 0:14:16
590000 -- (-956.403) (-935.579) (-941.279) [-955.455] -- 0:14:13
600000 -- (-965.409) (-949.490) (-940.922) [-932.924] -- 0:14:07
610000 -- (-948.417) (-945.902) (-947.985) [-925.888] -- 0:14:03
620000 -- (-936.017) [-943.401] (-950.686) (-947.564) -- 0:13:56
630000 -- (-954.573) (-951.758) [-938.822] (-955.029) -- 0:13:50
640000 -- [-936.942] (-967.257) (-934.698) (-948.270) -- 0:13:44
650000 -- (-946.879) (-937.670) [-939.830] (-951.332) -- 0:13:38
660000 -- (-966.595) (-953.876) (-937.519) [-941.373] -- 0:13:36
670000 -- (-934.119) [-943.653] (-973.770) (-942.533) -- 0:13:29
680000 -- (-941.440) (-940.264) (-965.192) [-943.277] -- 0:13:23
690000 -- (-957.701) (-953.645) [-939.908] (-943.903) -- 0:13:15
700000 -- (-956.708) [-936.047] (-949.250) (-947.300) -- 0:13:11
710000 -- (-955.844) (-947.796) [-933.697] (-938.944) -- 0:13:03
720000 -- (-942.242) (-932.853) [-933.258] (-944.764) -- 0:12:56
730000 -- [-933.600] (-942.810) (-964.002) (-945.608) -- 0:12:52
740000 -- [-951.307] (-945.463) (-952.726) (-941.148) -- 0:12:46
750000 -- [-942.197] (-948.696) (-958.047) (-948.306) -- 0:12:38
760000 -- (-952.956) [-932.217] (-953.203) (-945.281) -- 0:12:32
770000 -- (-951.126) [-937.996] (-961.204) (-956.869) -- 0:12:24
780000 -- [-942.038] (-952.265) (-958.698) (-944.965) -- 0:12:18
790000 -- (-942.014) (-956.199) [-938.823] (-943.796) -- 0:12:10
800000 -- (-956.080) [-939.505] (-969.418) (-936.897) -- 0:12:04
810000 -- (-940.772) [-936.974] (-952.856) (-949.069) -- 0:11:56
820000 -- (-968.960) [-944.222] (-945.530) (-938.820) -- 0:11:50
830000 -- (-957.292) (-952.909) (-972.062) [-937.183] -- 0:11:46
840000 -- (-953.750) (-939.265) [-938.609] (-951.872) -- 0:11:40
850000 -- (-954.720) (-946.566) (-945.641) [-956.029] -- 0:11:32
860000 -- [-952.504] (-957.265) (-936.190) (-942.814) -- 0:11:26
870000 -- (-966.589) (-937.699) [-943.110] (-946.914) -- 0:11:20
880000 -- (-936.914) (-948.494) [-947.911] (-938.014) -- 0:11:14
890000 -- (-970.012) (-954.130) (-946.865) [-937.508] -- 0:11:07
900000 -- (-965.870) (-934.626) (-958.652) [-934.549] -- 0:11:01
910000 -- (-945.323) (-945.783) [-945.862] (-951.481) -- 0:10:53
920000 -- [-944.094] (-954.000) (-958.869) (-939.347) -- 0:10:48
930000 -- (-948.623) (-942.336) [-929.047] (-973.866) -- 0:10:40
940000 -- (-944.452) [-940.111] (-963.354) (-945.005) -- 0:10:34
950000 -- (-943.715) [-935.242] (-959.485) (-959.983) -- 0:10:27
960000 -- (-937.469) (-934.587) [-937.805] (-953.959) -- 0:10:21
```

```
970000 -- [-937.250] (-951.277) (-940.329) (-933.825) -- 0:10:14
980000 -- (-950.020) [-939.588] (-952.639) (-952.575) -- 0:10:08
990000 -- [-944.306] (-945.235) (-946.712) (-961.864) -- 0:10:02
1000000 -- (-941.583) (-964.245) (-959.712) [-950.668] -- 0:09:58
1010000 -- (-946.181) (-938.012) [-943.257] (-933.815) -- 0:09:52
1020000 -- [-947.756] (-937.361) (-962.722) (-942.845) -- 0:09:46
1030000 -- (-950.679) (-942.785) [-935.143] (-943.399) -- 0:09:42
1040000 -- (-948.735) [-945.352] (-947.380) (-947.544) -- 0:09:36
1050000 -- (-936.558) (-942.708) [-950.389] (-950.988) -- 0:09:30
1060000 -- (-955.279) [-960.088] (-936.190) (-953.336) -- 0:09:24
1070000 -- [-936.587] (-951.411) (-957.258) (-957.110) -- 0:09:18
1080000 -- (-954.794) (-945.837) (-949.618) [-943.366] -- 0:09:12
1090000 -- [-943.513] (-970.021) (-938.748) (-963.868) -- 0:09:05
1100000 -- (-949.045) (-949.585) [-935.552] (-965.631) -- 0:08:59
1110000 -- (-940.104) [-942.832] (-943.005) (-960.150) -- 0:08:53
1120000 -- [-941.307] (-959.470) (-952.508) (-960.427) -- 0:08:46
1130000 -- (-944.933) (-956.858) [-934.887] (-930.151) -- 0:08:40
1140000 -- [-937.874] (-962.268) (-942.574) (-948.658) -- 0:08:33
1150000 -- (-945.986) [-956.585] (-950.500) (-935.874) -- 0:08:27
1160000 -- [-930.005] (-945.101) (-947.517) (-937.118) -- 0:08:21
1170000 -- [-931.648] (-946.005) (-947.993) (-946.162) -- 0:08:14
1180000 -- (-953.726) (-950.120) (-945.169) [-933.647] -- 0:08:08
1190000 -- (-945.917) [-936.644] (-941.833) (-956.338) -- 0:08:01
1200000 -- [-941.785] (-941.825) (-947.350) (-944.472) -- 0:07:56
1210000 -- [-939.283] (-945.913) (-961.139) (-950.880) -- 0:07:49
1220000 -- (-940.428) (-942.500) (-957.176) [-956.941] -- 0:07:43
1230000 -- (-951.847) (-953.003) [-939.577] (-946.709) -- 0:07:36
1240000 -- (-962.562) [-948.055] (-951.359) (-936.534) -- 0:07:31
1250000 -- (-950.229) [-945.850] (-937.218) (-955.748) -- 0:07:24
1260000 -- [-936.353] (-954.916) (-943.966) (-956.985) -- 0:07:18
1270000 -- (-936.325) [-935.337] (-955.291) (-951.999) -- 0:07:12
1280000 -- [-959.463] (-941.132) (-949.448) (-946.188) -- 0:07:06
1290000 -- [-939.965] (-953.091) (-946.539) (-943.268) -- 0:07:00
1300000 -- [-933.010] (-956.170) (-968.077) (-945.396) -- 0:06:54
1310000 -- (-950.338) (-933.592) [-938.278] (-941.479) -- 0:06:48
1320000 -- [-941.130] (-964.054) (-962.124) (-936.620) -- 0:06:42
1330000 -- [-933.245] (-958.594) (-938.576) (-944.456) -- 0:06:35
1340000 -- [-936.819] (-935.248) (-947.994) (-947.352) -- 0:06:30
1350000 -- (-954.746) (-951.183) (-951.192) [-933.690] -- 0:06:23
1360000 -- (-959.827) (-938.311) (-945.975) [-936.801] -- 0:06:17
1370000 -- (-946.953) (-936.817) (-938.938) [-935.051] -- 0:06:11
1380000 -- (-957.702) (-953.344) [-937.245] (-961.633) -- 0:06:05
1390000 -- [-938.604] (-943.406) (-946.778) (-950.486) -- 0:05:59
1400000 -- (-964.522) (-952.324) [-938.272] (-942.260) -- 0:05:53
1410000 -- (-951.496) [-945.116] (-975.790) (-954.268) -- 0:05:47
1420000 -- (-961.508) (-962.601) [-949.204] (-936.399) -- 0:05:41
1430000 -- (-939.174) (-947.373) [-938.820] (-956.200) -- 0:05:35
1440000 -- (-956.209) [-946.606] (-961.229) (-939.893) -- 0:05:29
1450000 -- (-958.095) (-940.851) (-958.556) [-947.457] -- 0:05:23
1460000 -- (-962.448) [-933.482] (-948.203) (-946.883) -- 0:05:17
1470000 -- (-946.528) (-947.070) (-947.565) [-933.865] -- 0:05:11
1480000 -- (-948.265) [-941.362] (-960.055) (-942.283) -- 0:05:05
1490000 -- (-940.081) [-938.230] (-956.180) (-960.492) -- 0:04:59
1500000 -- (-946.649) [-936.376] (-954.326) (-963.756) -- 0:04:53
1510000 -- (-936.026) [-932.688] (-956.255) (-966.857) -- 0:04:47
1520000 -- [-937.321] (-943.045) (-950.257) (-960.978) -- 0:04:42
1530000 -- (-949.562) [-928.693] (-961.269) (-945.591) -- 0:04:36
1540000 -- (-940.760) [-953.156] (-941.007) (-947.187) -- 0:04:30
1550000 -- [-941.511] (-949.557) (-947.982) (-931.924) -- 0:04:24
1560000 -- (-952.284) (-938.661) (-941.518) [-929.941] -- 0:04:18
1570000 -- (-938.187) (-962.548) (-932.809) [-945.196] -- 0:04:13
```

```
1580000 -- (-959.472) (-955.734) (-938.409) [-929.875] -- 0:04:06
1590000 -- [-938.736] (-946.975) (-938.167) (-952.272) -- 0:04:01
1600000 -- (-955.026) (-941.347) (-953.322) [-939.701] -- 0:03:55
1610000 -- (-960.395) (-959.308) [-952.808] (-956.877) -- 0:03:49
1620000 -- (-945.370) (-944.677) [-956.473] (-948.310) -- 0:03:43
1630000 -- (-943.516) (-968.468) [-939.168] (-966.979) -- 0:03:37
1640000 -- [-936.031] (-941.108) (-963.489) (-957.033) -- 0:03:31
1650000 -- (-959.348) (-940.399) [-947.727] (-941.791) -- 0:03:25
1660000 -- (-967.826) (-940.735) (-955.878) [-943.745] -- 0:03:19
1670000 -- (-948.996) (-949.344) [-944.915] (-958.872) -- 0:03:13
1680000 -- (-948.518) (-948.038) (-938.160) [-937.832] -- 0:03:07
1690000 -- (-939.468) [-941.043] (-942.795) (-949.851) -- 0:03:01
1700000 -- (-967.394) [-945.524] (-953.463) (-949.781) -- 0:02:55
1710000 -- (-953.447) (-940.040) [-931.270] (-940.059) -- 0:02:49
1720000 -- (-930.857) (-948.984) [-933.560] (-966.618) -- 0:02:44
1730000 -- (-945.393) [-934.088] (-962.576) (-948.582) -- 0:02:38
1740000 -- [-939.481] (-933.005) (-929.867) (-959.968) -- 0:02:32
1750000 -- (-943.706) (-955.810) [-957.021] (-952.004) -- 0:02:26
1760000 -- (-940.840) (-943.904) [-947.896] (-956.705) -- 0:02:20
1770000 -- (-949.227) (-940.314) (-947.551) [-931.106] -- 0:02:14
1780000 -- (-945.167) [-942.012] (-942.807) (-957.950) -- 0:02:08
1790000 -- [-937.575] (-953.041) (-949.270) (-945.022) -- 0:02:02
1800000 -- (-948.111) [-942.833] (-961.864) (-940.360) -- 0:01:56
1810000 -- (-952.176) (-953.928) (-953.776) [-935.684] -- 0:01:51
1820000 -- (-944.427) (-939.725) (-942.434) [-943.967] -- 0:01:45
1830000 -- [-946.194] (-955.043) (-968.386) (-946.043) -- 0:01:39
1840000 -- [-945.126] (-941.489) (-952.311) (-961.573) -- 0:01:33
1850000 -- (-940.284) [-939.721] (-936.626) (-961.766) -- 0:01:27
1860000 -- (-949.464) (-934.013) (-953.510) [-943.063] -- 0:01:21
1870000 -- (-956.328) (-950.925) (-931.317) [-938.571] -- 0:01:15
1880000 -- (-947.882) [-942.491] (-967.223) (-951.251) -- 0:01:10
1890000 -- (-957.727) [-936.348] (-944.914) (-943.402) -- 0:01:04
1900000 -- (-944.964) (-952.184) (-965.366) [-934.786] -- 0:00:58
1910000 -- (-959.411) [-943.075] (-944.435) (-943.762) -- 0:00:52
1920000 -- [-933.605] (-936.004) (-954.864) (-947.715) -- 0:00:46
1930000 -- (-955.836) (-947.938) [-941.532] (-959.244) -- 0:00:40
1940000 -- (-949.072) [-930.812] (-952.141) (-942.085) -- 0:00:34
1950000 -- (-947.068) [-938.693] (-947.294) (-947.478) -- 0:00:29
1960000 -- (-943.164) (-933.278) (-941.903) [-931.363] -- 0:00:23
1970000 -- (-942.522) (-958.734) [-947.760] (-945.780) -- 0:00:17
1980000 -- [-934.918] (-962.867) (-946.354) (-935.704) -- 0:00:11
1990000 -- [-938.773] (-932.182) (-952.449) (-948.470) -- 0:00:05
2000000 -- (-937.128) [-940.276] (-935.883) (-953.540) -- 0:00:00

Continue with analysis? (yes/no): n

Analysis completed in 19 mins 24 seconds
Analysis used 1070.60 seconds of CPU time
Log likelihood of best state for "cold" chain was -919.19

Acceptance rates for the moves in the "cold" chain:
   With prob.   (last 100)   chain accepted proposals by move
      40.1 %     ( 33 %)     Multiplier(Alpha)
      18.6 %     ( 14 %)     ExtSprClock(Tau,V)
      44.8 %     ( 54 %)     NNIClock(Tau,V)
      15.5 %     ( 10 %)     ParsSPRClock(Tau,V)
      70.3 %     ( 69 %)     NodesliderClock(V)
      70.6 %     ( 24 %)     TreeStretch(V)

Chain swap information:
```

```
            1       2       3       4
      ---------------------------------
    1 |           0.53    0.25    0.11
    2 |  333780           0.60    0.32
    3 |  332922  333333           0.64
    4 |  333623  333364  332978

 Upper diagonal: Proportion of successful state exchanges between chains
 Lower diagonal: Number of attempted state exchanges between chains

 Chain information:

   ID -- Heat
  -----------
    1 -- 1.00  (cold chain)
    2 -- 0.91
    3 -- 0.83
    4 -- 0.77

 Heat = 1 / (1 + T * (ID - 1))
    (where T = 0.10 is the temperature and ID is the chain number)


MrBayes > sumt relburnin=yes

 Using relative burnin (a fraction of samples discarded).
 Summarizing trees in file "DY-26Dec-strict-Yout.t"
 Using relative burnin ('relburnin=yes'), discarding the first 25 % of sampled trees
 Writing statistics to files DY-26Dec-strict-Yout.<parts|tstat|vstat|trprobs|con>
 Examining file ...
 Found one tree block in file "DY-26Dec-strict-Yout.t" with 4001 trees in last block

 Tree reading status:

 0      10      20      30      40      50      60      70      80      90     100
 v-------v-------v-------v-------v-------v-------v-------v-------v-------v-------v
 *********************************************************************************

 Read 4001 trees from last tree block (sampling 3001 of them)

 General explanation:

 In an unrooted tree, a taxon bipartition (split) is specified by removing a
 branch, thereby dividing the species into those to the left and those to the
 right of the branch. Here, taxa to one side of the removed branch are denoted
 '.' and those to the other side are denoted '*'. Specifically, the '.' symbol
 is used for the taxa on the same side as the outgroup.

 In a rooted or clock tree, the tree is rooted using the model and not by
 reference to an outgroup. Each bipartition therefore corresponds to a clade,
 that is, a group that includes all the descendants of a particular branch in
 the tree.  Taxa that are included in each clade are denoted using '*', and
 taxa that are not included are denoted using the '.' symbol.

 The output first includes a key to all the bipartitions with frequency larger
 or equual to (Minpartfreq) in at least one run. Minpartfreq is a paramiter to
 sumt command and currently it is set to 0.10.  This is followed by a table
 with statistics for the informative bipartitions (those including at least
 two taxa), sorted from highest to lowest probability. For each bipartition,
 the table gives the number of times the partition or split was observed in all
 runs (#obs) and the posterior probability of the bipartition (Probab.), which
```

is the same as the split frequency. If several runs are summarized, this is followed by the minimum split frequency (Min(s)), the maximum frequency (Max(s)), and the standard deviation of frequencies (Stddev(s)) across runs. The latter value should approach 0 for all bipartitions as MCMC runs converge.

This is followed by a table summarizing branch lengths, node heights (if a clock model was used) and relaxed clock parameters (if a relaxed clock model was used). The mean, variance, and 95 % credible interval are given for each of these parameters. If several runs are summarized, the potential scale reduction factor (PSRF) is also given; it should approach 1 as runs converge. Node heights will take calibration points into account, if such points were used in the analysis.

Note that Stddev may be unreliable if the partition is not present in all runs (the last column indicates the number of runs that sampled the partition if more than one run is summarized). The PSRF is not calculated at all if the partition is not present in all runs.The PSRF is also sensitive to small sample sizes and it should only be considered a rough guide to convergence since some of the assumptions allowing one to interpret it as a true potential scale reduction factor are violated in MrBayes.

List of taxa in bipartitions:

```
   1 -- gwi
   2 -- dgr
   3 -- scsh
   4 -- xsl
   5 -- bea
   6 -- crx
   7 -- chp
   8 -- txc
   9 -- haa
  10 -- ing
  11 -- kuu
  12 -- hoi
  13 -- koy
  14 -- taa
  15 -- aht
  16 -- tfn
  17 -- kkz
  18 -- tcb
  19 -- tau
  20 -- ttmN
  21 -- tceS
  22 -- eya
  23 -- tli
  24 -- gce
  25 -- tol
  26 -- cco
  27 -- hup
  28 -- mtl
  29 -- wlk
  30 -- kto
  31 -- apc
  32 -- apw
  33 -- apj
  34 -- nav
  35 -- apk
  36 -- apl
  37 -- srs
  38 -- ket
```

```
  39 -- zko

Key to taxon bipartitions (saved to file "DY-26Dec-strict-Yout.parts"):

 ID -- Partition
----------------------------------------------
  0 -- ***************************************
  1 -- *......................................
  2 -- .*.....................................
  3 -- ..*....................................
  4 -- ...*...................................
  5 -- ....*..................................
  6 -- .....*.................................
  7 -- ......*................................
  8 -- .......*...............................
  9 -- ........*..............................
 10 -- .........*.............................
 11 -- ..........*............................
 12 -- ...........*...........................
 13 -- ............*..........................
 14 -- .............*.........................
 15 -- ..............*........................
 16 -- ...............*.......................
 17 -- ................*......................
 18 -- .................*.....................
 19 -- ..................*....................
 20 -- ...................*...................
 21 -- ....................*..................
 22 -- .....................*.................
 23 -- ......................*................
 24 -- .......................*...............
 25 -- ........................*..............
 26 -- .........................*.............
 27 -- ..........................*............
 28 -- ...........................*...........
 29 -- ............................*..........
 30 -- .............................*.........
 31 -- ..............................*........
 32 -- ...............................*.......
 33 -- ................................*......
 34 -- .................................*.....
 35 -- ..................................*....
 36 -- ...................................*...
 37 -- ....................................*..
 38 -- .....................................*.
 39 -- ......................................*
 40 -- *************************************..
 41 -- .....................................**
 42 -- ..................................**...
 43 -- ..............................*******..
 44 -- .....................**................
 45 -- .............*...**....................
 46 -- ..........................****.........
 47 -- ..............................****.....
 48 -- ............................**.........
 49 -- .........*.**..........................
 50 -- ..............................******...
 51 -- ...*..*.*....*..*****..................
 52 -- ..............................***......
 53 -- ***.**.................................
 54 -- ...*............*......................
```

```
 55 -- ..............................**.......
 56 -- ....**.................................
 57 -- ...........**..........................
 58 -- *******.*....*..*****..................
 59 -- .......................***.............
 60 -- ......*............**..................
 61 -- ...*..*.*.......*..**..................
 62 -- .............*...*.....................
 63 -- .......................**..............
 64 -- **************************....*******..
 65 -- ...................**..................
 66 -- *********************..***....*******..
 67 -- ...........................***.........
 68 -- .........****..........................
 69 -- **.....................................
 70 -- ........................**.............
 71 -- ..........................*.**.........
 72 -- .................**....................
 73 -- ..............**.......................
 74 -- .**....................................
 75 -- ..*.**.................................
 76 -- *********************.........*******..
 77 -- ...*..*.**.***..*****..................
 78 -- ..*..*.................................
 79 -- .........*.*...........................
 80 -- *********************..................
 81 -- ...*....*.......*......................
 82 -- .........*.***...**....................
 83 -- ..............*...............*******..
 84 -- *.*.**.................................
 85 -- ..............................*.*......
 86 -- ......*.............*..................
 87 -- ......*............*...................
 88 -- .......................***....*******..
 89 -- ...............................**......
 90 -- *...**.................................
 91 -- *******.*....*..*****.........*******..
 92 -- ***..*.................................
 93 -- *********************..**************..
 94 -- .......*.......*.......................
 95 -- ...*..*.........*..**..................
 96 -- ..................................***..
 97 -- .............*....*....................
 98 -- ***********************.......*******..
 99 -- .........****............*.............
100 -- .........*..*..........................
101 -- .......*......**.......................
102 -- .......*..*............................
103 -- ..............................**.*.....
104 -- *********************....*....*******..
105 -- ..........................**...........
106 -- *******.**.***..*****..................
107 -- ......*.*..........**..................
108 -- ........*....*...**....................
109 -- ***....................................
110 -- ...*...............*...................
111 -- ..........*....*.......................
112 -- .......................*******.........
113 -- .......*......*........................
-----------------------------------------------
```

Summary statistics for informative taxon bipartitions (clades)
(saved to file "DY-26Dec-strict-Yout.tstat"):

ID	#obs	Probab.
40	3001	1.000000
41	3001	1.000000
42	3000	0.999667
43	2767	0.922026
44	2696	0.898367
45	2622	0.873709
46	2539	0.846051
47	2522	0.840387
48	2518	0.839054
49	2396	0.798401
50	2217	0.738754
51	2067	0.688770
52	2040	0.679773
53	2037	0.678774
54	1801	0.600133
55	1767	0.588804
56	1715	0.571476
57	1686	0.561813
58	1647	0.548817
59	1608	0.535821
60	1594	0.531156
61	1580	0.526491
62	1577	0.525492
63	1384	0.461180
64	1361	0.453515
65	1309	0.436188
66	1282	0.427191
67	1272	0.423859
68	1225	0.408197
69	1202	0.400533
70	1136	0.378540
71	1131	0.376874
72	943	0.314229
73	869	0.289570
74	855	0.284905
75	849	0.282906
76	822	0.273909
77	777	0.258914
78	768	0.255915
79	756	0.251916
80	741	0.246918
81	690	0.229923
82	609	0.202932
83	606	0.201933
84	589	0.196268
85	585	0.194935
86	564	0.187937
87	554	0.184605
88	553	0.184272
89	550	0.183272
90	533	0.177607
91	532	0.177274
92	526	0.175275
93	520	0.173276
94	498	0.165945
95	481	0.160280

96	471	0.156948
97	417	0.138954
98	384	0.127957
99	363	0.120960
100	358	0.119294
101	357	0.118960
102	355	0.118294
103	353	0.117627
104	351	0.116961
105	351	0.116961
106	347	0.115628
107	347	0.115628
108	344	0.114628
109	341	0.113629
110	328	0.109297
111	327	0.108964
112	326	0.108630
113	326	0.108630

Summary statistics for branch and node parameters
(saved to file "DY-26Dec-strict-Yout.vstat"):

Parameter	Mean	Variance	95% HPD Interval Lower	95% HPD Interval Upper	Median
length[1]	0.048019	0.000374	0.012385	0.084269	0.046128
length[2]	0.050230	0.000529	0.012989	0.098331	0.046047
length[3]	0.043713	0.000352	0.011244	0.080542	0.041384
length[4]	0.034120	0.000235	0.007774	0.063780	0.032147
length[5]	0.042629	0.000654	0.007309	0.098330	0.036828
length[6]	0.032730	0.000234	0.008190	0.062968	0.030478
length[7]	0.043334	0.000253	0.015299	0.074194	0.041820
length[8]	0.098497	0.000888	0.038457	0.152049	0.098401
length[9]	0.047581	0.000233	0.018304	0.077108	0.047113
length[10]	0.047953	0.000478	0.011545	0.089664	0.044194
length[11]	0.077381	0.000905	0.021743	0.138188	0.075436
length[12]	0.035402	0.000248	0.007991	0.066125	0.033154
length[13]	0.036355	0.000273	0.007897	0.067311	0.033864
length[14]	0.027673	0.000214	0.005571	0.058083	0.024950
length[15]	0.094470	0.000881	0.039486	0.152294	0.094379
length[16]	0.091397	0.000887	0.035261	0.147808	0.091357
length[17]	0.036138	0.000258	0.009314	0.067995	0.034239
length[18]	0.024711	0.000170	0.004349	0.049862	0.022045
length[19]	0.030469	0.000239	0.005634	0.061502	0.027956
length[20]	0.033689	0.000195	0.010236	0.062464	0.032016
length[21]	0.035897	0.000223	0.009668	0.064411	0.034185
length[22]	0.097014	0.001172	0.037673	0.165628	0.093576
length[23]	0.099618	0.001388	0.033374	0.172188	0.094912
length[24]	0.087039	0.001057	0.032204	0.154714	0.083948
length[25]	0.075774	0.000895	0.025982	0.137985	0.071399
length[26]	0.083066	0.000993	0.025470	0.143234	0.080296
length[27]	0.090926	0.001201	0.027278	0.159159	0.086512
length[28]	0.085646	0.001044	0.023525	0.149109	0.081732
length[29]	0.044674	0.000420	0.011405	0.084643	0.041759
length[30]	0.046055	0.000510	0.011240	0.091675	0.042223
length[31]	0.008930	0.000063	0.000006	0.024855	0.006662
length[32]	0.008922	0.000062	0.000006	0.024552	0.006644
length[33]	0.014789	0.000096	0.000359	0.033375	0.012848
length[34]	0.028931	0.000173	0.006547	0.053453	0.027011

length[35]	0.005296	0.000029	0.000003	0.016085	0.003673
length[36]	0.005295	0.000029	0.000003	0.016085	0.003673
length[37]	0.065006	0.000574	0.023191	0.112532	0.062697
length[38]	0.082629	0.000685	0.029604	0.130430	0.080783
length[39]	0.082629	0.000685	0.029604	0.130430	0.080783
length[40]	0.015233	0.000188	0.000033	0.042705	0.011254
length[41]	0.100513	0.000891	0.041529	0.158148	0.099646
length[42]	0.038389	0.000276	0.007112	0.070377	0.037017
length[43]	0.042779	0.000500	0.000046	0.083674	0.040379
length[44]	0.055368	0.000791	0.004774	0.107841	0.052653
length[45]	0.026570	0.000212	0.000607	0.053665	0.024396
length[46]	0.054482	0.000800	0.004102	0.108383	0.051798
length[47]	0.019756	0.000169	0.000598	0.046417	0.017162
length[48]	0.038371	0.000530	0.002140	0.081966	0.035095
length[49]	0.031431	0.000401	0.001405	0.072738	0.027591
length[50]	0.026179	0.000319	0.000044	0.059619	0.022812
length[51]	0.021532	0.000139	0.000729	0.044020	0.019821
length[52]	0.015401	0.000135	0.000003	0.036953	0.013223
length[53]	0.023205	0.000301	0.000051	0.055923	0.019721
length[54]	0.019928	0.000164	0.000360	0.043855	0.017711
length[55]	0.012946	0.000094	0.000004	0.032079	0.011010
length[56]	0.026804	0.000323	0.000098	0.062123	0.023185
length[57]	0.019686	0.000217	0.000052	0.047390	0.015915
length[58]	0.028779	0.000247	0.000101	0.056998	0.026723
length[59]	0.034202	0.000483	0.000062	0.077597	0.030534
length[60]	0.012524	0.000078	0.000027	0.030315	0.010607
length[61]	0.017483	0.000149	0.000040	0.040461	0.015166
length[62]	0.012607	0.000109	0.000051	0.033104	0.009986
length[63]	0.038788	0.000689	0.000175	0.089016	0.033689
length[64]	0.017258	0.000140	0.000021	0.039979	0.015023
length[65]	0.013889	0.000122	0.000002	0.035238	0.011429
length[66]	0.018661	0.000164	0.000095	0.043868	0.015718
length[67]	0.035299	0.000652	0.000041	0.084239	0.029609
length[68]	0.032652	0.000422	0.000092	0.070390	0.030493
length[69]	0.024231	0.000245	0.000014	0.054359	0.021549
length[70]	0.036348	0.000562	0.000155	0.080607	0.032760
length[71]	0.029276	0.000442	0.000065	0.069815	0.024828
length[72]	0.013899	0.000168	0.000056	0.040138	0.010248
length[73]	0.028093	0.000406	0.000029	0.064145	0.024507
length[74]	0.022612	0.000199	0.000098	0.049096	0.020718
length[75]	0.021048	0.000280	0.000105	0.054404	0.017250
length[76]	0.016613	0.000167	0.000076	0.042528	0.013884
length[77]	0.025208	0.000200	0.003817	0.053030	0.022373
length[78]	0.020315	0.000202	0.000066	0.045146	0.017497
length[79]	0.013234	0.000149	0.000056	0.036768	0.009841
length[80]	0.014984	0.000116	0.000168	0.034014	0.012560
length[81]	0.009515	0.000067	0.000049	0.026433	0.007322
length[82]	0.022697	0.000287	0.000079	0.057042	0.018823
length[83]	0.025407	0.000279	0.000014	0.056485	0.022031
length[84]	0.021671	0.000214	0.000001	0.048279	0.018881
length[85]	0.007412	0.000051	0.000006	0.020630	0.005538
length[86]	0.007471	0.000058	0.000006	0.022737	0.005196
length[87]	0.007702	0.000055	0.000002	0.022872	0.005376
length[88]	0.020505	0.000200	0.000055	0.047507	0.017428
length[89]	0.007775	0.000055	0.000015	0.021750	0.005669
length[90]	0.018841	0.000228	0.000058	0.048340	0.015343
length[91]	0.016014	0.000121	0.000270	0.037523	0.013499
length[92]	0.019608	0.000223	0.000042	0.047617	0.016453
length[93]	0.014640	0.000114	0.000083	0.035698	0.012171
length[94]	0.020340	0.000314	0.000000	0.054471	0.015342
length[95]	0.007781	0.000059	0.000004	0.023178	0.005455

length[96]	0.014836	0.000123	0.000146	0.036064	0.012021
length[97]	0.007722	0.000067	0.000002	0.025652	0.005010
length[98]	0.011334	0.000069	0.000078	0.027048	0.009232
length[99]	0.031997	0.000343	0.000752	0.065665	0.029096
length[100]	0.008762	0.000073	0.000088	0.025437	0.006355
length[101]	0.017243	0.000188	0.000278	0.044028	0.013108
length[102]	0.021578	0.000284	0.000136	0.053594	0.018178
length[103]	0.007575	0.000064	0.000006	0.023018	0.004943
length[104]	0.018035	0.000162	0.000342	0.044510	0.015812
length[105]	0.017165	0.000315	0.000005	0.052348	0.011462
length[106]	0.015828	0.000155	0.000031	0.041464	0.013052
length[107]	0.006146	0.000040	0.000010	0.019642	0.004216
length[108]	0.011048	0.000080	0.000061	0.027799	0.008875
length[109]	0.012929	0.000152	0.000008	0.038329	0.009130
length[110]	0.018592	0.000159	0.000017	0.041913	0.016385
length[111]	0.024416	0.000405	0.000060	0.061403	0.019069
length[112]	0.020929	0.000245	0.000073	0.054000	0.017564
length[113]	0.019135	0.000217	0.000014	0.045780	0.016715
height[0]	0.183142	0.001638	0.087549	0.254876	0.186298
height[1]	0.000000	0.000000	0.000000	0.000000	0.000000
height[2]	0.000000	0.000000	0.000000	0.000000	0.000000
height[3]	0.000000	0.000000	0.000000	0.000000	0.000000
height[4]	0.000000	0.000000	0.000000	0.000000	0.000000
height[5]	0.000000	0.000000	0.000000	0.000000	0.000000
height[6]	0.000000	0.000000	0.000000	0.000000	0.000000
height[7]	0.000000	0.000000	0.000000	0.000000	0.000000
height[8]	0.000000	0.000000	0.000000	0.000000	0.000000
height[9]	0.000000	0.000000	0.000000	0.000000	0.000000
height[10]	0.000000	0.000000	0.000000	0.000000	0.000000
height[11]	0.000000	0.000000	0.000000	0.000000	0.000000
height[12]	0.000000	0.000000	0.000000	0.000000	0.000000
height[13]	0.000000	0.000000	0.000000	0.000000	0.000000
height[14]	0.000000	0.000000	0.000000	0.000000	0.000000
height[15]	0.000000	0.000000	0.000000	0.000000	0.000000
height[16]	0.000000	0.000000	0.000000	0.000000	0.000000
height[17]	0.000000	0.000000	0.000000	0.000000	0.000000
height[18]	0.000000	0.000000	0.000000	0.000000	0.000000
height[19]	0.000000	0.000000	0.000000	0.000000	0.000000
height[20]	0.000000	0.000000	0.000000	0.000000	0.000000
height[21]	0.000000	0.000000	0.000000	0.000000	0.000000
height[22]	0.000000	0.000000	0.000000	0.000000	0.000000
height[23]	0.000000	0.000000	0.000000	0.000000	0.000000
height[24]	0.000000	0.000000	0.000000	0.000000	0.000000
height[25]	0.000000	0.000000	0.000000	0.000000	0.000000
height[26]	0.000000	0.000000	0.000000	0.000000	0.000000
height[27]	0.000000	0.000000	0.000000	0.000000	0.000000
height[28]	0.000000	0.000000	0.000000	0.000000	0.000000
height[29]	0.000000	0.000000	0.000000	0.000000	0.000000
height[30]	0.000000	0.000000	0.000000	0.000000	0.000000
height[31]	0.000000	0.000000	0.000000	0.000000	0.000000
height[32]	0.000000	0.000000	0.000000	0.000000	0.000000
height[33]	0.000000	0.000000	0.000000	0.000000	0.000000
height[34]	0.000000	0.000000	0.000000	0.000000	0.000000
height[35]	0.000000	0.000000	0.000000	0.000000	0.000000
height[36]	0.000000	0.000000	0.000000	0.000000	0.000000
height[37]	0.000000	0.000000	0.000000	0.000000	0.000000
height[38]	0.000000	0.000000	0.000000	0.000000	0.000000
height[39]	0.000000	0.000000	0.000000	0.000000	0.000000
height[40]	0.167909	0.001317	0.081373	0.229592	0.171755
height[41]	0.082629	0.000685	0.029604	0.130430	0.080783
height[42]	0.005295	0.000029	0.000003	0.016085	0.003673

height[43]	0.066481	0.000417	0.026557	0.105882	0.065416
height[44]	0.093881	0.001021	0.037673	0.159094	0.091440
height[45]	0.033116	0.000189	0.009595	0.061063	0.031191
height[46]	0.102391	0.001026	0.042778	0.166595	0.100010
height[47]	0.028533	0.000125	0.009584	0.051721	0.027206
height[48]	0.041036	0.000301	0.011369	0.074123	0.038808
height[49]	0.048285	0.000327	0.015818	0.083738	0.045882
height[50]	0.044176	0.000220	0.017174	0.072169	0.042834
height[51]	0.065150	0.000245	0.033008	0.095937	0.065179
height[52]	0.014978	0.000068	0.001897	0.030781	0.013598
height[53]	0.069300	0.000407	0.028284	0.107602	0.069172
height[54]	0.029660	0.000167	0.007284	0.054593	0.028261
height[55]	0.006038	0.000032	0.000006	0.017760	0.004275
height[56]	0.028693	0.000177	0.005489	0.053387	0.026349
height[57]	0.031158	0.000179	0.007897	0.057390	0.029416
height[58]	0.089558	0.000362	0.049131	0.126202	0.090132
height[59]	0.094737	0.000763	0.041322	0.147139	0.093611
height[60]	0.039682	0.000156	0.016490	0.065003	0.038708
height[61]	0.054537	0.000230	0.022797	0.082004	0.054715
height[62]	0.022444	0.000118	0.005005	0.044961	0.020652
height[63]	0.072667	0.000687	0.023511	0.122876	0.069770
height[64]	0.150508	0.001044	0.072353	0.204358	0.153304
height[65]	0.029260	0.000144	0.009668	0.053869	0.028344
height[66]	0.140656	0.000767	0.081936	0.195689	0.143319
height[67]	0.074506	0.000579	0.029684	0.121958	0.072843
height[68]	0.075365	0.000549	0.029981	0.119853	0.073731
height[69]	0.041171	0.000285	0.011326	0.072419	0.039508
height[70]	0.067330	0.000550	0.025042	0.111354	0.066585
height[71]	0.076314	0.000603	0.028932	0.122504	0.074988
height[72]	0.023432	0.000150	0.004356	0.048067	0.020790
height[73]	0.079767	0.000637	0.032454	0.126123	0.078404
height[74]	0.040709	0.000248	0.012978	0.073608	0.039228
height[75]	0.048123	0.000264	0.014921	0.076852	0.047198
height[76]	0.127393	0.000859	0.057519	0.173912	0.130760
height[77]	0.077643	0.000466	0.036648	0.119126	0.077535
height[78]	0.032515	0.000176	0.011618	0.059946	0.030826
height[79]	0.035833	0.000219	0.012425	0.067251	0.033711
height[80]	0.122428	0.000711	0.067489	0.176501	0.124422
height[81]	0.045036	0.000189	0.016559	0.069046	0.044812
height[82]	0.064196	0.000385	0.027431	0.101606	0.063986
height[83]	0.092099	0.000568	0.041530	0.136175	0.092127
height[84]	0.055955	0.000260	0.021386	0.086542	0.055506
height[85]	0.009033	0.000042	0.000579	0.022113	0.007801
height[86]	0.036777	0.000182	0.013609	0.063915	0.035280
height[87]	0.035356	0.000153	0.012215	0.058953	0.033885
height[88]	0.115153	0.000671	0.060249	0.164516	0.116463
height[89]	0.008526	0.000035	0.000145	0.020558	0.007203
height[90]	0.049973	0.000248	0.017945	0.079136	0.049378
height[91]	0.110590	0.000582	0.057274	0.154344	0.111678
height[92]	0.057250	0.000317	0.023696	0.089045	0.055594
height[93]	0.162172	0.000987	0.096265	0.222731	0.164429
height[94]	0.088287	0.000755	0.036193	0.134163	0.087953
height[95]	0.049884	0.000195	0.024840	0.078307	0.049030
height[96]	0.045371	0.000212	0.018361	0.072299	0.045225
height[97]	0.025406	0.000146	0.005634	0.048365	0.023209
height[98]	0.138191	0.001087	0.062999	0.191624	0.140927
height[99]	0.086759	0.000547	0.041920	0.128979	0.085942
height[100]	0.037749	0.000217	0.011230	0.065227	0.035481
height[101]	0.100380	0.000585	0.050811	0.146473	0.101642
height[102]	0.075899	0.000651	0.030729	0.124125	0.074569
height[103]	0.022240	0.000073	0.008026	0.039545	0.020861

height[104]	0.131501	0.000779	0.071591	0.183411	0.134467
height[105]	0.080572	0.000745	0.034494	0.133221	0.078131
height[106]	0.098979	0.000726	0.046480	0.145191	0.099383
height[107]	0.049165	0.000216	0.021797	0.077075	0.048240
height[108]	0.047920	0.000161	0.024001	0.072701	0.047379
height[109]	0.055402	0.000344	0.017284	0.086528	0.053967
height[110]	0.027531	0.000147	0.006280	0.050178	0.026656
height[111]	0.074238	0.000682	0.028201	0.123897	0.072333
height[112]	0.131160	0.001156	0.068856	0.203331	0.131808
height[113]	0.090919	0.000641	0.036343	0.135972	0.092054

Clade credibility values:

```
                                                           /---------- ket (38)
/----------------------------100---------------------------+
|                                                          \---------- zko (39)
|
|                                                /-------------------- gwi (1)
|                                                |
|                                                |-------------------- dgr (2)
|                                                |
|                             /--------68--------+-------------------- scsh (3)
|                             |                  |
|                             |                  |         /---------- bea (5)
|                             |                  \----57---+
|                             |                            \---------- crx (6)
|                             |
|                             |                            /---------- xsl (4)
|                             |                  /----60---+
|                             |                  |         \---------- kkz (17)
|         /---------55--------+                  |
|         |                   |                  |         /---------- chp (7)
|         |                   |                  |         |
|         |                   |        /----53---+----53---+---------- ttmN (20)
|         |                   |        |         |         |
|         |                   |        |         |         \---------- tceS (21)
|         |                   |        |         |
+         |                   |        |         \-------------------- haa (9)
|         |                   \---69---+
|         |                            |                   /---------- taa (14)
|         |                            |         /----53---+
|         |                            |         |         \---------- tcb (18)
|         |                            \----87---+
|         |                                      \-------------------- tau (19)
|         |
|         |----------------------------------------------------------- txc (8)
|         |
|         |                                      /-------------------- ing (10)
|         |                                      |
|         |------------------80------------------+         /---------- hoi (12)
|         |                                      \----56---+
|         |                                                \---------- koy (13)
|         |
|         |----------------------------------------------------------- kuu (11)
|         |
|         |----------------------------------------------------------- aht (15)
|         |
```

```
|         |----------------------------------------------------------- tfn (16)
|         |
\---100---+                                                /---------- eya (22)
          |-----------------------90-----------------------+
          |                                                \---------- tli (23)
          |
          |                                                /---------- gce (24)
          |                                                |
          |-----------------------54-----------------------+---------- tol (25)
          |                                                |
          |                                                \---------- cco (26)
          |
          |                                      /-------------------- hup (27)
          |                                      |
          |                                      |-------------------- mtl (28)
          |------------------85------------------+
          |                                      |         /---------- wlk (29)
          |                                      \----84---+
          |                                                \---------- kto (30)
          |
          |                                                /---------- apc (31)
          |                                      /----59---+
          |                                      |         \---------- apw (32)
          |                            /----68---+
          |                            |         \-------------------- apj (33)
          |                   /---84---+
          |                   |        \------------------------------ nav (34)
          |         /----74---+
          |         |         |                            /---------- apk (35)
          |         |         \------------100------------+
          \----92---+                                      \---------- apl (36)
                    |
                    \------------------------------------------------- srs (37)
```

Phylogram (based on median node depths):

```
                                       /------------------------------ ket (38)
/--------------------------------------+
|                                      \------------------------------ zko (39)
|
|                                          /-------------------------- gwi (1)
|                                          |
|                                          |-------------------------- dgr (2)
|                                          |
|                                   /------+-------------------------- scsh (3)
|                                   |      |
|                                   |      |               /---------- bea (5)
|                                   |      \---------------+
|                                   |                      \---------- crx (6)
|                                   |
|                                   |                      /---------- xsl (4)
|                                   |            /---------+
|                                   |            |         \---------- kkz (17)
|    /------------------------------+            |
|    |                              |            |     /-------------- chp (7)
|    |                              |            |     |
|    |                              |        /---+-----+-------------- ttmN (20)
|    |                              |        |   |     |
|    |                              |        |   |     \-------------- tceS (21)
|    |                              |        |   |
```

```
+    |                              |        |   \-------------------- haa (9)
|    |                              \--------+
|    |                                       |               /-------- taa (14)
|    |                                       |           /---+
|    |                                       |           |   \-------- tcb (18)
|    |                                       \-----------+
|    |                                                   \------------ tau (19)
|    |
|    |---------------------------------------------------------------- txc (8)
|    |
|    |                                              /----------------- ing (10)
|    |                                              |
|    |----------------------------------------------+     /----------- hoi (12)
|    |                                              \-----+
|    |                                                    \----------- koy (13)
|    |
|    |---------------------------------------------------------------- kuu (11)
|    |
|    |---------------------------------------------------------------- aht (15)
|    |
|    |---------------------------------------------------------------- tfn (16)
|    |
\----+                             /---------------------------------- eya (22)
     |-----------------------------+
     |                             \---------------------------------- tli (23)
     |
     |                            /----------------------------------- gce (24)
     |                            |
     |----------------------------+----------------------------------- tol (25)
     |                            |
     |                            \----------------------------------- cco (26)
     |
     |                          /------------------------------------- hup (27)
     |                          |
     |                          |------------------------------------- mtl (28)
     |--------------------------+
     |                          |                      /-------------- wlk (29)
     |                          \----------------------+
     |                                                 \-------------- kto (30)
     |
     |                                                             /-- apc (31)
     |                                                          /--+
     |                                                          |  \-- apw (32)
     |                                                     /----+
     |                                                     |    \----- apj (33)
     |                                               /-----+
     |                                               |     \---------- nav (34)
     |                                       /-------+
     |                                       |       |              /- apk (35)
     |                                       |       \--------------+
     \---------------------------------------+                      \- apl (36)
                                             |
                                             \------------------------ srs (37)

--------------|-----------------|------------------|-----------------|
            0.15              0.10               0.05              0.00

[Expected changes per site]

Calculating tree probabilities...
```

```
   Credible sets of trees (3001 trees sampled):
      50 % credible set contains 1501 trees
      90 % credible set contains 2701 trees
      95 % credible set contains 2851 trees
      99 % credible set contains 2971 trees


MrBayes > sump relburnin=yes

   Using relative burnin (a fraction of samples discarded).
   Summarizing parameters in file DY-26Dec-strict-Yout.p
   Writing summary statistics to file DY-26Dec-strict-Yout.pstat
   Using relative burnin ('relburnin=yes'), discarding the first 25 % of samples

   Below is a rough plot of the generation (x-axis) versus the log
   probability of observing the data (y-axis). You can use this
   graph to determine what the burn in for your analysis should be.
   When the log probability starts to plateau you may be at station-
   arity. Sample trees and parameters after the log probability
   plateaus. Of course, this is not a guarantee that you are at sta-
   analysis should be. When the log probability starts to plateau
   tionarity. When possible, run multiple analyses starting from dif-
   ferent random trees; if the inferences you make for independent
   analyses are the same, this is reasonable evidence that the chains
   have converged. You can use MrBayes to run several independent
   analyses simultaneously. During such a run, MrBayes will monitor
   the convergence of topologies. After the run has been completed,
   the 'sumt' and 'sump' functions will provide additional conver-
   gence diagnostics for all the parameters in your model. Remember
   that the burn in is the number of samples to discard. There are
   a total of ngen / samplefreq samples taken during a MCMC analysis.

   +------------------------------------------------------------+ -937.661
   |                                                 *   *      |
   |                     *  *                       *           |
   |*               *     *               *   *   *        *  **|
   |                    *                      *          *     |
   |     **  *  **                *         **          **      |
   |    *      *     ***   *    *  ****             *       *   |
   |  *                        *       **        *  *       *   |
   | * *    * *   **          *          *                      |
   |       *                 *   *           *                  |
   |                                                            |
   |                                                            |
   |                                                            |
   |                                                            |
   |                                                            |
   |                                            *               |
   +------+-----+-----+-----+-----+-----+-----+-----+-----+-----+ -945.106
   ^                                                            ^
   500000                                                       2000000


   Estimated marginal likelihoods for run sampled in file "DY-26Dec-strict-Yout.p":
      (Use the harmonic mean for Bayes factor comparisons of models)
      (Values are saved to the file /Users/msicoli/Yeniseian-NaDene-Typlogical_noHAX.nex.lstat)

   Arithmetic mean   Harmonic mean
   --------------------------------
       -928.25          -957.98
   --------------------------------
```

```
   Model parameter summaries for run sampled in file "DY-26Dec-strict-Yout":
      Based on a total of 3001 samples out of a total of 4001 samples
         from this analysis.
      Parameter summaries saved to file "/Users/msicoli/Yeniseian-NaDene-Typlogical_noHAX.nex.pstat".

                                          95% HPD Interval
                                        --------------------
   Parameter      Mean      Variance     Lower       Upper       Median     ESS*
   ------------------------------------------------------------------------------
   TH          0.183142    0.001638    0.087549    0.254876    0.186298    283.23
   TL          2.917361    0.341884    1.479322    3.862267    3.003610    258.33
   alpha       7.814915  618.921256    0.128622   34.662511    1.840312   2060.16
   ------------------------------------------------------------------------------
   * Convergence diagnostic (ESS = Estimated Sample Size); ESS value
     below 100 may indicate that the parameter is undersampled.



MrBayes > ssp ngen=100000 diagnfreq=1000 filename=YND-Typ-NoHax-Yout-ss

   Setting number of generations to 100000
   Setting diagnosing frequency to 1000

MrBayes > ss

   Setting chain output file names to "YND-Typ-NoHax-Yout-ss.<p/t>"
   Running Markov chain
   MCMC stamp = 9313293518
   Seed = 500640120
   Swapseed = 1325479247
   Model settings:

      Data not partitioned --
         Datatype  = Standard
         Coding    = Variable
         # States  = Variable, up to 10
                     State frequencies are fixed to be equal
         Rates     = Gamma
                     Gamma shape parameter is uniformly dist-
                     ributed on the interval (0.00,200.00).
                     Gamma distribution is approximated using 4 categories.
                     Likelihood summarized over all rate categories in each generation.

   Active parameters:

      Parameters
      ------------------
      Statefreq        1
      Shape            2
      Ratemultiplier   3
      Topology         4
      Brlens           5
      Clockrate        6
      ------------------

      1 --  Parameter  = Alpha_symdir
            Type       = Symmetric diricihlet/beta distribution alpha_i parameter
            Prior      = Symmetric dirichlet with fixed(-1.00) variance parameter
```

```
   2 --  Parameter  = Alpha
         Type       = Shape of scaled gamma distribution of site rates
         Prior      = Uniform(0.00,200.00)

   3 --  Parameter  = Ratemultiplier
         Type       = Partition-specific rate multiplier
         Prior      = Fixed(1.0)

   4 --  Parameter  = Tau
         Type       = Topology
         Prior      = Prior on topologies obeys constraints
         Subparam.  = V

   5 --  Parameter  = V
         Type       = Branch lengths
         Prior      = Clock:Uniform
                      Tree age has an Exponential(1.000) distribution
                      Node ages are not constrained

   6 --  Parameter  = Clockrate
         Type       = Base rate of clock
         Prior      = Fixed(1.000000)
                      The clock rate is constant (strict clock)


Number of taxa = 39
Number of characters = 116

The MCMC sampler will use the following moves:
   With prob.  Chain will use move
      2.38 %   Multiplier(Alpha)
     11.90 %   ExtSprClock(Tau,V)
     23.81 %   NNIClock(Tau,V)
     11.90 %   ParsSPRClock(Tau,V)
     47.62 %   NodesliderClock(V)
      2.38 %   TreeStretch(V)

Division 1 has 78 unique site patterns
Initializing conditional likelihoods
Using standard non-SSE likelihood calculator for division 1 (single-precision)

Initial log likelihoods and log prior probs:
   Chain 1 -- -940.276105 -- -25.105876
   Chain 2 -- -937.127525 -- -30.598855
   Chain 3 -- -953.540022 -- -35.491175
   Chain 4 -- -935.883526 -- -31.235408


Starting stepping-stone sampling to estimate marginal likelihood.
50 steps will be used with 1500 generations (3 samples) within each step.
Total of 76500 generations (153 samples) will be collected while first
1500 generations (3 samples) will be discarded as initial burnin.
Additionally at the begining of each step 0 generations (0 samples)
will be discarded as burnin.
Sampling from posterior to prior, i.e. first step samples from close to
posterior.
NOTE: Number of generation of each step is reduced to the closest multiple
of sampling frequency. That is why, in total it will be taken 76500 gene-
rations instead of requested 100000.
```

```
Chain results (76500 generations requested):

    0 -- [-940.276] (-937.128) (-953.540) (-935.884)

Sampling step 1 out of 50 steps...

Sampling step 2 out of 50 steps...

Sampling step 3 out of 50 steps...

Sampling step 4 out of 50 steps...

Sampling step 5 out of 50 steps...

Sampling step 6 out of 50 steps...

10000 -- (-983.462) (-954.663) [-960.939] (-953.831) -- 0:00:33
Sampling step 7 out of 50 steps...

Sampling step 8 out of 50 steps...

Sampling step 9 out of 50 steps...

Sampling step 10 out of 50 steps...

Sampling step 11 out of 50 steps...

Sampling step 12 out of 50 steps...

Sampling step 13 out of 50 steps...

20000 -- (-992.849) [-982.415] (-1116.329) (-1053.194) -- 0:00:31
Sampling step 14 out of 50 steps...

Sampling step 15 out of 50 steps...

Sampling step 16 out of 50 steps...

Sampling step 17 out of 50 steps...

Sampling step 18 out of 50 steps...

Sampling step 19 out of 50 steps...

30000 -- (-1116.901) (-1228.345) (-1069.047) [-1082.594] -- 0:00:24
Sampling step 20 out of 50 steps...

Sampling step 21 out of 50 steps...

Sampling step 22 out of 50 steps...

Sampling step 23 out of 50 steps...

Sampling step 24 out of 50 steps...

Sampling step 25 out of 50 steps...

Sampling step 26 out of 50 steps...

40000 -- (-1183.647) (-1240.637) (-1265.053) [-1159.604] -- 0:00:19
Sampling step 27 out of 50 steps...
```

```
Sampling step 28 out of 50 steps...

Sampling step 29 out of 50 steps...

Sampling step 30 out of 50 steps...

Sampling step 31 out of 50 steps...

Sampling step 32 out of 50 steps...

Sampling step 33 out of 50 steps...

50000 -- (-1257.997) (-1328.011) (-1322.755) [-1307.306] -- 0:00:14
Sampling step 34 out of 50 steps...

Sampling step 35 out of 50 steps...

Sampling step 36 out of 50 steps...

Sampling step 37 out of 50 steps...

Sampling step 38 out of 50 steps...

Sampling step 39 out of 50 steps...

60000 -- (-1312.262) (-1318.707) (-1303.586) [-1328.344] -- 0:00:09
Sampling step 40 out of 50 steps...

Sampling step 41 out of 50 steps...

Sampling step 42 out of 50 steps...

Sampling step 43 out of 50 steps...

Sampling step 44 out of 50 steps...

Sampling step 45 out of 50 steps...

Sampling step 46 out of 50 steps...

70000 -- (-2065.694) (-2060.396) (-2043.780) [-1442.593] -- 0:00:03
Sampling step 47 out of 50 steps...

Sampling step 48 out of 50 steps...

Sampling step 49 out of 50 steps...

Sampling step 50 out of 50 steps...


Analysis completed in 41 seconds
Analysis used 40.26 seconds of CPU time
Log likelihood of best state for "cold" chain was -926.40

Marginal likelihood (in natural log units) estimated using stepping-stone sampling based on
50 steps with 1500 generations (3 samples) within each step.

    Run   Marginal likelihood (ln)
    ------------------------------
      1     -1044.58
```

```
    ------------------------------
More statistics on stepping-stone sampling is dumped to YND-Typ-NoHax-Yout-ss.ss file.

Plot of average standard deviation of split frequencies across steps.
Points at -1.0 (y-axis) indicate that there were no splits
above minimum frequency for corresponding step.
+------------------------------------------------------------+ 0.000
|                                                            |
|                                                            |
|                                                            |
|                                                            |
|                                                            |
|                                                            |
|                                                            |
|                                                            |
|                                                            |
|                                                            |
|                                                            |
|                                                            |
|                                                            |
|                                                            |
|***** **** ***** **** ***** **** ***** **** ***** **** *****|
+------+-----+-----+-----+-----+-----+-----+-----+-----+-----+ 0.000
^                                                            ^
1                                                           50


Acceptance rates for the moves in the "cold" chain:
   With prob.   (last 100)   chain accepted proposals by move
      44.7 %     ( 43 %)     Multiplier(Alpha)
      42.8 %     ( 62 %)     ExtSprClock(Tau,V)
      78.3 %     ( 96 %)     NNIClock(Tau,V)
      33.9 %     ( 32 %)     ParsSPRClock(Tau,V)
      86.4 %     ( 97 %)     NodesliderClock(V)
      52.6 %     ( 83 %)     TreeStretch(V)

Chain swap information:

            1      2      3      4
     ------------------------------
   1 |          0.26   0.09   0.04
   2 |  12529          0.33   0.10
   3 |  12772  12801          0.41
   4 |  12816  12721  12861

Upper diagonal: Proportion of successful state exchanges between chains
Lower diagonal: Number of attempted state exchanges between chains

Chain information:

  ID -- Heat
 -----------
   1 -- 1.00  (cold chain)
   2 -- 0.91
   3 -- 0.83
   4 -- 0.77

Heat = 1 / (1 + T * (ID - 1))
   (where T = 0.10 is the temperature and ID is the chain number)
```

```
                            MrBayes v3.2.1 x64

                      (Bayesian Analysis of Phylogeny)

               Distributed under the GNU General Public License


                Type "help" or "help <command>" for information
                      on the commands that are available.

                    Type "about" for authorship and general
                        information about the program.


MrBayes > exe /Users/msicoli/Yeniseian-NaDene-Typlogical_noHAX.nex

   Executing file "/Users/msicoli/Yeniseian-NaDene-Typlogical_noHAX.nex"
   UNIX line termination
   Longest line length = 123
   Parsing file
   Expecting NEXUS formatted file
   Reading data block
      Allocated taxon set
      Allocated matrix
      Defining new matrix with 39 taxa and 116 characters
      Data is Standard
      Missing data coded as ?
      Gaps coded as -
      Data matrix is not interleaved
      Taxon  1 -> gwi
      Taxon  2 -> dgr
      Taxon  3 -> scsh
      Taxon  4 -> xsl
      Taxon  5 -> bea
      Taxon  6 -> crx
      Taxon  7 -> chp
      Taxon  8 -> txc
      Taxon  9 -> haa
      Taxon 10 -> ing
      Taxon 11 -> kuu
      Taxon 12 -> hoi
      Taxon 13 -> koy
      Taxon 14 -> taa
      Taxon 15 -> aht
      Taxon 16 -> tfn
      Taxon 17 -> kkz
      Taxon 18 -> tcb
      Taxon 19 -> tau
      Taxon 20 -> ttmN
      Taxon 21 -> tceS
      Taxon 22 -> eya
      Taxon 23 -> tli
      Taxon 24 -> gce
      Taxon 25 -> tol
      Taxon 26 -> cco
      Taxon 27 -> hup
      Taxon 28 -> mtl
      Taxon 29 -> wlk
```

```
      Taxon 30 -> kto
      Taxon 31 -> apc
      Taxon 32 -> apw
      Taxon 33 -> apj
      Taxon 34 -> nav
      Taxon 35 -> apk
      Taxon 36 -> apl
      Taxon 37 -> srs
      Taxon 38 -> ket
      Taxon 39 -> zko
      Successfully read matrix
      Setting default partition (does not divide up characters)
      Setting model defaults
      Seed (for generating default start values) = 1387987200
      Adding dummy characters (unobserved site patterns) for division 1
      WARNING: There are 32 characters incompatible with the specified
               coding bias. These characters will be excluded.
      Setting output file names to "/Users/msicoli/Yeniseian-NaDene-Typlogical_noHAX.nex.run<i>.<p|
t>"
   Exiting data block
   Reached end of file

MrBayes > lset nst=6 rates=gamma

   Setting Rates to Gamma
   Successfully set likelihood model parameters
   Adding dummy characters (unobserved site patterns) for division 1
   WARNING: There are 32 characters incompatible with the specified
            coding bias. These characters will be excluded.

MrBayes > prset brlenspr=clock:uniform

   Setting Brlenspr to Clock:Uniform
   Successfully set prior model parameters
   Adding dummy characters (unobserved site patterns) for division 1
   WARNING: There are 32 characters incompatible with the specified
            coding bias. These characters will be excluded.

MrBayes > mcmcp ngen=2000000 printfreq=10000 samplefreq=500 nruns=1 nchains=4 savebrlens=yes
filename=DY-25Dec-strictNOHAX

   Setting number of generations to 2000000
   Setting print frequency to 10000
   Setting sample frequency to 500
   Setting number of runs to 1
   Setting number of chains to 4
   Setting chain output file names to "DY-25Dec-strictNOHAX.<p/t>"
   Successfully set chain parameters

MrBayes > mcmc

   Running Markov chain
   MCMC stamp = 9243191534
   Seed = 1101468095
   Swapseed = 1387987200
   Model settings:

      Data not partitioned --
         Datatype  = Standard
```

```
      Coding    = Variable
      # States  = Variable, up to 10
                  State frequencies are fixed to be equal
      Rates     = Gamma
                  Gamma shape parameter is uniformly dist-
                  ributed on the interval (0.00,200.00).
                  Gamma distribution is approximated using 4 categories.
                  Likelihood summarized over all rate categories in each generation.

Active parameters:

   Parameters
   ------------------
   Statefreq        1
   Shape            2
   Ratemultiplier   3
   Topology         4
   Brlens           5
   Clockrate        6
   ------------------

   1 --  Parameter  = Alpha_symdir
         Type       = Symmetric diricihlet/beta distribution alpha_i parameter
         Prior      = Symmetric dirichlet with fixed(-1.00) variance parameter

   2 --  Parameter  = Alpha
         Type       = Shape of scaled gamma distribution of site rates
         Prior      = Uniform(0.00,200.00)

   3 --  Parameter  = Ratemultiplier
         Type       = Partition-specific rate multiplier
         Prior      = Fixed(1.0)

   4 --  Parameter  = Tau
         Type       = Topology
         Prior      = All topologies equally probable a priori
         Subparam.  = V

   5 --  Parameter  = V
         Type       = Branch lengths
         Prior      = Clock:Uniform
                      Tree age has an Exponential(1.000) distribution
                      Node ages are not constrained

   6 --  Parameter  = Clockrate
         Type       = Base rate of clock
         Prior      = Fixed(1.000000)
                      The clock rate is constant (strict clock)


Number of taxa = 39
Number of characters = 116

The MCMC sampler will use the following moves:
   With prob.  Chain will use move
      2.38 %   Multiplier(Alpha)
     11.90 %   ExtSprClock(Tau,V)
     23.81 %   NNIClock(Tau,V)
     11.90 %   ParsSPRClock(Tau,V)
```

```
     47.62 %   NodesliderClock(V)
      2.38 %   TreeStretch(V)

Division 1 has 78 unique site patterns
Initializing conditional likelihoods
Using standard non-SSE likelihood calculator for division 1 (single-precision)

Initial log likelihoods and log prior probs:
   Chain 1 -- -1491.863997 -- -90.228070
   Chain 2 -- -1591.318217 -- -90.228070
   Chain 3 -- -1549.036671 -- -90.228070
   Chain 4 -- -1553.401708 -- -90.228070


Chain results (2000000 generations requested):

    0 -- [-1491.864] (-1591.318) (-1549.037) (-1553.402)
10000 -- [-937.355] (-942.024) (-963.513) (-947.137) -- 0:16:35
20000 -- [-931.394] (-947.608) (-935.664) (-968.341) -- 0:16:30
30000 -- (-937.840) [-945.915] (-946.888) (-952.444) -- 0:16:25
40000 -- [-941.067] (-956.564) (-946.847) (-953.556) -- 0:16:20
50000 -- (-954.203) (-947.643) (-944.162) [-952.468] -- 0:16:53
60000 -- (-932.417) (-956.754) (-958.387) [-929.299] -- 0:16:42
70000 -- (-949.555) (-958.891) (-950.307) [-941.312] -- 0:17:00
80000 -- (-948.222) (-951.925) (-948.616) [-941.422] -- 0:16:47
90000 -- [-950.302] (-947.418) (-942.583) (-964.886) -- 0:16:37
100000 -- [-936.393] (-949.996) (-961.094) (-938.256) -- 0:16:27
110000 -- (-942.820) (-957.695) [-942.194] (-937.556) -- 0:16:36
120000 -- [-957.475] (-936.398) (-952.990) (-941.147) -- 0:16:26
130000 -- (-966.704) (-956.605) (-961.169) [-941.583] -- 0:16:18
140000 -- (-951.292) (-962.371) (-933.908) [-943.302] -- 0:16:09
150000 -- [-928.330] (-952.126) (-961.511) (-936.169) -- 0:16:01
160000 -- [-939.073] (-949.622) (-959.640) (-971.102) -- 0:15:54
170000 -- (-953.075) (-950.311) [-935.609] (-943.604) -- 0:15:47
180000 -- [-940.858] (-945.358) (-938.486) (-976.296) -- 0:15:40
190000 -- (-958.537) (-939.033) [-947.339] (-958.251) -- 0:15:33
200000 -- (-939.163) (-981.545) (-949.729) [-935.578] -- 0:15:27
210000 -- [-938.314] (-944.417) (-953.403) (-953.384) -- 0:15:20
220000 -- [-953.410] (-951.328) (-950.109) (-947.759) -- 0:15:14
230000 -- [-943.901] (-931.957) (-962.056) (-967.190) -- 0:15:08
240000 -- (-953.986) (-947.102) (-980.836) [-944.537] -- 0:15:02
250000 -- (-949.951) (-950.770) (-942.813) [-934.273] -- 0:14:56
260000 -- (-934.000) (-960.175) (-949.966) [-937.244] -- 0:14:50
270000 -- (-938.609) (-956.120) (-949.767) [-946.458] -- 0:14:44
280000 -- (-944.272) [-943.586] (-968.907) (-937.138) -- 0:14:38
290000 -- (-955.054) (-951.905) (-949.548) [-934.545] -- 0:14:32
300000 -- (-966.969) (-948.120) (-962.669) [-934.747] -- 0:14:27
310000 -- (-967.104) (-958.022) [-954.944] (-959.656) -- 0:14:21
320000 -- (-948.597) (-935.504) (-943.957) [-923.184] -- 0:14:21
330000 -- [-969.896] (-938.042) (-948.680) (-943.430) -- 0:14:15
340000 -- (-943.719) (-961.604) (-944.355) [-949.185] -- 0:14:09
350000 -- (-951.234) (-953.650) (-943.576) [-946.564] -- 0:14:13
360000 -- (-956.263) (-963.897) [-933.043] (-938.962) -- 0:14:11
370000 -- (-937.073) (-951.267) [-937.434] (-956.318) -- 0:14:05
380000 -- (-959.523) (-967.166) [-945.313] (-954.123) -- 0:13:59
390000 -- (-954.761) (-945.539) [-946.972] (-955.073) -- 0:13:53
400000 -- (-952.331) [-933.233] (-949.177) (-958.077) -- 0:13:47
410000 -- (-949.442) [-931.422] (-953.286) (-943.410) -- 0:13:42
420000 -- (-946.281) (-942.367) (-959.965) [-938.005] -- 0:13:40
```

```
430000 -- (-948.190) (-931.362) (-951.487) [-942.069] -- 0:13:34
440000 -- (-956.410) (-949.344) (-955.221) [-945.501] -- 0:13:28
450000 -- (-949.060) (-963.003) (-944.955) [-940.969] -- 0:13:22
460000 -- (-953.972) (-944.687) (-943.767) [-958.391] -- 0:13:16
470000 -- (-965.498) (-955.830) [-933.433] (-950.290) -- 0:13:11
480000 -- (-950.727) (-959.634) (-952.038) [-946.686] -- 0:13:05
490000 -- [-952.605] (-980.507) (-945.860) (-946.559) -- 0:12:59
500000 -- (-941.620) (-946.275) (-952.934) [-945.884] -- 0:12:54
510000 -- (-960.510) (-956.775) [-951.614] (-959.034) -- 0:12:45
520000 -- (-953.020) (-966.495) (-968.744) [-943.081] -- 0:12:39
530000 -- (-961.576) [-942.323] (-968.338) (-964.420) -- 0:12:34
540000 -- [-945.171] (-953.074) (-969.792) (-950.026) -- 0:12:28
550000 -- (-967.644) [-942.136] (-951.312) (-950.994) -- 0:12:23
560000 -- [-935.048] (-936.408) (-956.076) (-942.144) -- 0:12:18
570000 -- (-965.434) [-935.725] (-956.447) (-956.846) -- 0:12:12
580000 -- (-951.101) [-936.082] (-944.782) (-957.836) -- 0:12:07
590000 -- (-943.283) (-947.447) [-945.814] (-949.558) -- 0:12:01
600000 -- (-965.503) (-950.470) [-932.551] (-953.454) -- 0:11:56
610000 -- (-961.870) (-953.709) (-964.860) [-953.055] -- 0:11:50
620000 -- (-946.166) (-940.598) (-956.277) [-940.207] -- 0:11:43
630000 -- (-967.548) [-939.136] (-938.238) (-937.428) -- 0:11:38
640000 -- (-947.072) (-953.853) [-945.162] (-961.193) -- 0:11:32
650000 -- [-947.103] (-945.870) (-942.390) (-967.242) -- 0:11:27
660000 -- (-952.460) (-947.100) [-934.427] (-947.625) -- 0:11:22
670000 -- (-974.147) [-948.559] (-952.161) (-953.197) -- 0:11:16
680000 -- (-951.343) (-961.887) [-937.799] (-952.649) -- 0:11:11
690000 -- (-959.785) [-940.668] (-961.349) (-933.157) -- 0:11:04
700000 -- [-951.860] (-944.644) (-936.300) (-940.611) -- 0:10:59
710000 -- [-933.585] (-943.479) (-940.807) (-971.696) -- 0:10:54
720000 -- (-944.020) [-932.168] (-950.626) (-963.962) -- 0:10:48
730000 -- (-979.578) [-935.104] (-946.969) (-976.455) -- 0:10:43
740000 -- (-943.342) [-947.840] (-956.041) (-957.019) -- 0:10:38
750000 -- (-955.619) (-956.604) [-938.917] (-950.457) -- 0:10:33
760000 -- (-933.708) (-945.322) (-960.303) [-934.581] -- 0:10:28
770000 -- (-953.175) (-952.847) (-942.552) [-960.200] -- 0:10:21
780000 -- (-947.945) [-952.417] (-941.755) (-944.997) -- 0:10:16
790000 -- (-953.298) (-951.212) [-949.528] (-949.182) -- 0:10:11
800000 -- (-964.228) (-937.635) (-970.198) [-946.546] -- 0:10:06
810000 -- [-952.428] (-957.455) (-955.931) (-938.665) -- 0:10:00
820000 -- (-959.384) [-931.940] (-952.530) (-939.868) -- 0:09:55
830000 -- (-950.404) (-943.402) [-940.504] (-936.578) -- 0:09:50
840000 -- (-948.214) (-951.265) [-948.531] (-939.789) -- 0:09:45
850000 -- [-936.333] (-940.802) (-963.623) (-956.137) -- 0:09:39
860000 -- [-943.295] (-949.373) (-931.339) (-944.642) -- 0:09:33
870000 -- [-944.600] (-971.124) (-956.322) (-941.198) -- 0:09:28
880000 -- (-952.086) (-958.467) [-944.276] (-944.528) -- 0:09:23
890000 -- (-935.019) (-963.331) (-954.709) [-934.909] -- 0:09:18
900000 -- [-955.367] (-961.971) (-940.293) (-946.221) -- 0:09:13
910000 -- [-944.956] (-952.784) (-948.711) (-946.135) -- 0:09:08
920000 -- [-941.351] (-958.436) (-941.965) (-965.884) -- 0:09:03
930000 -- [-943.045] (-949.997) (-957.026) (-966.243) -- 0:08:57
940000 -- [-947.958] (-952.685) (-948.906) (-966.185) -- 0:08:52
950000 -- [-943.547] (-944.791) (-953.795) (-958.219) -- 0:08:47
960000 -- [-948.664] (-937.475) (-953.963) (-951.974) -- 0:08:42
970000 -- (-945.228) (-943.000) [-940.391] (-958.682) -- 0:08:37
980000 -- (-942.721) [-955.005] (-943.118) (-948.029) -- 0:08:32
990000 -- (-949.308) (-958.432) [-944.956] (-947.329) -- 0:08:27
1000000 -- (-963.763) [-942.600] (-939.282) (-941.277) -- 0:08:21
1010000 -- [-956.026] (-952.028) (-958.046) (-951.130) -- 0:08:16
```

```
1020000 -- (-946.793) (-941.185) [-944.532] (-941.661) -- 0:08:11
1030000 -- (-952.455) (-954.924) [-950.183] (-960.060) -- 0:08:05
1040000 -- (-941.923) [-929.049] (-953.460) (-951.416) -- 0:08:00
1050000 -- (-938.974) (-952.326) [-944.670] (-948.231) -- 0:07:56
1060000 -- [-956.726] (-965.371) (-946.292) (-952.556) -- 0:07:52
1070000 -- (-951.692) [-942.686] (-960.545) (-945.504) -- 0:07:47
1080000 -- (-943.267) (-952.522) [-946.081] (-937.700) -- 0:07:42
1090000 -- [-938.702] (-949.284) (-961.352) (-956.833) -- 0:07:37
1100000 -- (-948.645) (-945.578) (-943.055) [-935.429] -- 0:07:32
1110000 -- [-931.289] (-951.192) (-965.766) (-940.369) -- 0:07:27
1120000 -- [-948.976] (-952.158) (-955.938) (-943.702) -- 0:07:22
1130000 -- [-938.595] (-941.782) (-953.220) (-947.949) -- 0:07:17
1140000 -- (-949.733) [-949.839] (-958.227) (-955.826) -- 0:07:12
1150000 -- (-947.964) [-948.141] (-941.553) (-961.317) -- 0:07:07
1160000 -- (-938.667) (-964.196) (-969.126) [-942.027] -- 0:07:02
1170000 -- (-951.838) (-947.635) (-943.859) [-934.886] -- 0:06:57
1180000 -- (-958.206) [-955.667] (-944.763) (-975.749) -- 0:06:52
1190000 -- [-943.004] (-940.644) (-957.882) (-938.882) -- 0:06:46
1200000 -- [-942.800] (-961.546) (-931.666) (-947.273) -- 0:06:41
1210000 -- [-945.910] (-949.287) (-955.417) (-953.544) -- 0:06:36
1220000 -- (-937.509) [-929.336] (-953.669) (-962.570) -- 0:06:31
1230000 -- (-941.420) (-947.795) (-971.376) [-936.714] -- 0:06:26
1240000 -- (-947.906) [-938.212] (-945.288) (-955.439) -- 0:06:21
1250000 -- (-958.908) (-966.160) (-964.825) [-947.414] -- 0:06:16
1260000 -- (-932.794) [-939.340] (-978.736) (-941.724) -- 0:06:11
1270000 -- [-954.204] (-957.737) (-954.729) (-950.189) -- 0:06:06
1280000 -- [-940.857] (-948.973) (-964.532) (-969.281) -- 0:06:01
1290000 -- (-938.503) (-951.940) [-932.657] (-945.145) -- 0:05:56
1300000 -- [-934.086] (-946.934) (-967.909) (-950.704) -- 0:05:51
1310000 -- (-942.838) [-932.611] (-951.848) (-970.486) -- 0:05:46
1320000 -- [-942.097] (-944.045) (-943.316) (-954.315) -- 0:05:41
1330000 -- (-946.721) (-947.238) [-938.817] (-950.817) -- 0:05:36
1340000 -- (-956.542) (-937.005) (-937.420) [-938.133] -- 0:05:30
1350000 -- (-937.701) [-952.295] (-951.341) (-947.539) -- 0:05:25
1360000 -- (-949.538) (-946.034) [-940.022] (-939.297) -- 0:05:20
1370000 -- (-946.998) (-956.339) (-953.340) [-952.229] -- 0:05:15
1380000 -- (-950.207) (-944.814) (-947.502) [-936.955] -- 0:05:10
1390000 -- (-948.517) (-955.098) [-936.029] (-957.723) -- 0:05:05
1400000 -- [-938.637] (-949.683) (-935.370) (-966.963) -- 0:05:00
1410000 -- [-937.853] (-950.536) (-946.847) (-962.059) -- 0:04:55
1420000 -- (-959.374) (-942.046) (-957.943) [-945.828] -- 0:04:50
1430000 -- [-930.024] (-937.028) (-965.919) (-962.063) -- 0:04:45
1440000 -- (-945.985) (-944.604) (-969.445) [-946.893] -- 0:04:40
1450000 -- [-950.006] (-952.739) (-951.027) (-941.568) -- 0:04:35
1460000 -- (-932.211) (-949.716) (-941.173) [-944.489] -- 0:04:30
1470000 -- (-950.342) [-941.645] (-951.928) (-957.010) -- 0:04:25
1480000 -- (-951.031) (-968.137) (-950.399) [-938.305] -- 0:04:20
1490000 -- (-951.054) (-966.496) (-957.142) [-952.299] -- 0:04:15
1500000 -- (-955.589) [-958.318] (-946.912) (-948.679) -- 0:04:10
1510000 -- (-948.730) (-944.100) [-951.469] (-954.367) -- 0:04:05
1520000 -- (-977.313) (-942.205) (-967.360) [-930.039] -- 0:03:59
1530000 -- (-939.329) (-961.150) [-936.775] (-950.428) -- 0:03:54
1540000 -- (-945.835) (-955.589) [-943.800] (-940.947) -- 0:03:49
1550000 -- (-957.611) (-971.595) [-945.675] (-952.787) -- 0:03:44
1560000 -- (-951.647) [-934.829] (-959.374) (-953.416) -- 0:03:39
1570000 -- (-965.877) (-946.653) (-956.057) [-937.952] -- 0:03:34
1580000 -- (-954.157) (-950.216) (-940.594) [-931.524] -- 0:03:29
1590000 -- [-931.065] (-939.935) (-970.260) (-950.905) -- 0:03:24
1600000 -- (-959.996) (-949.427) (-937.061) [-938.640] -- 0:03:19
```

```
1610000 -- (-962.705) (-952.874) [-949.204] (-959.354) -- 0:03:15
1620000 -- [-929.687] (-947.367) (-950.111) (-940.536) -- 0:03:10
1630000 -- (-948.250) [-939.399] (-934.502) (-953.434) -- 0:03:05
1640000 -- (-962.785) (-941.939) (-946.483) [-936.005] -- 0:03:00
1650000 -- (-955.376) [-937.256] (-966.304) (-954.228) -- 0:02:55
1660000 -- (-954.255) (-959.875) [-950.611] (-937.879) -- 0:02:49
1670000 -- (-951.161) (-968.658) [-946.802] (-943.666) -- 0:02:44
1680000 -- (-939.441) (-947.441) (-955.454) [-947.561] -- 0:02:39
1690000 -- (-965.142) [-943.410] (-936.175) (-948.568) -- 0:02:34
1700000 -- (-947.082) [-935.016] (-938.505) (-940.893) -- 0:02:29
1710000 -- (-949.647) (-954.703) (-941.333) [-938.033] -- 0:02:24
1720000 -- (-948.931) (-953.071) (-950.412) [-938.602] -- 0:02:19
1730000 -- (-940.418) (-947.802) (-940.079) [-958.788] -- 0:02:14
1740000 -- (-943.354) (-953.244) (-972.695) [-947.118] -- 0:02:09
1750000 -- (-970.025) [-951.800] (-987.106) (-950.513) -- 0:02:04
1760000 -- (-974.234) (-940.658) (-960.836) [-942.011] -- 0:01:59
1770000 -- (-959.013) (-961.940) [-944.245] (-946.239) -- 0:01:54
1780000 -- (-951.159) (-954.037) (-953.819) [-943.614] -- 0:01:49
1790000 -- [-943.729] (-938.111) (-943.569) (-960.270) -- 0:01:44
1800000 -- (-951.347) [-945.395] (-951.588) (-958.375) -- 0:01:39
1810000 -- (-978.809) (-940.294) (-951.540) [-944.028] -- 0:01:34
1820000 -- [-949.341] (-954.698) (-963.215) (-943.387) -- 0:01:29
1830000 -- (-947.986) (-953.897) (-942.228) [-944.163] -- 0:01:24
1840000 -- (-946.008) (-964.946) (-965.080) [-945.631] -- 0:01:19
1850000 -- (-936.012) (-947.276) (-950.202) [-940.311] -- 0:01:14
1860000 -- (-962.804) [-937.911] (-942.993) (-945.219) -- 0:01:09
1870000 -- (-935.915) (-941.509) [-956.017] (-938.777) -- 0:01:04
1880000 -- [-943.399] (-947.969) (-951.893) (-954.331) -- 0:00:59
1890000 -- [-943.498] (-952.457) (-937.397) (-949.951) -- 0:00:54
1900000 -- (-952.150) (-949.121) [-936.722] (-951.804) -- 0:00:49
1910000 -- [-947.379] (-965.785) (-946.878) (-958.752) -- 0:00:44
1920000 -- (-955.702) (-968.372) [-948.499] (-952.332) -- 0:00:39
1930000 -- [-961.133] (-958.015) (-969.360) (-950.285) -- 0:00:34
1940000 -- (-939.164) [-944.908] (-959.496) (-950.614) -- 0:00:29
1950000 -- (-942.063) [-950.652] (-962.126) (-961.547) -- 0:00:24
1960000 -- [-942.198] (-954.845) (-960.291) (-945.832) -- 0:00:19
1970000 -- [-936.716] (-938.010) (-951.131) (-960.392) -- 0:00:14
1980000 -- (-949.791) [-929.682] (-940.695) (-955.712) -- 0:00:09
1990000 -- (-944.227) (-943.052) (-945.202) [-941.295] -- 0:00:04
2000000 -- (-941.922) (-945.921) (-968.723) [-937.554] -- 0:00:00

Continue with analysis? (yes/no): n

Analysis completed in 16 mins 36 seconds
Analysis used 968.47 seconds of CPU time
Log likelihood of best state for "cold" chain was -919.51

Acceptance rates for the moves in the "cold" chain:
   With prob.   (last 100)   chain accepted proposals by move
      39.6 %     ( 24 %)     Multiplier(Alpha)
      18.4 %     ( 15 %)     ExtSprClock(Tau,V)
      44.3 %     ( 41 %)     NNIClock(Tau,V)
      15.1 %     ( 16 %)     ParsSPRClock(Tau,V)
      70.0 %     ( 77 %)     NodesliderClock(V)
      69.9 %     ( 33 %)     TreeStretch(V)

Chain swap information:

             1       2       3       4
```

```
      ----------------------------------
    1 |           0.51    0.24    0.10
    2 |  333100           0.59    0.31
    3 |  332474  333374           0.62
    4 |  333812  334026  333214

 Upper diagonal: Proportion of successful state exchanges between chains
 Lower diagonal: Number of attempted state exchanges between chains

 Chain information:

   ID -- Heat
  -----------
    1 -- 1.00  (cold chain)
    2 -- 0.91
    3 -- 0.83
    4 -- 0.77

 Heat = 1 / (1 + T * (ID - 1))
    (where T = 0.10 is the temperature and ID is the chain number)


MrBayes > sumt relburnin=yes

 Using relative burnin (a fraction of samples discarded).
 Summarizing trees in file "DY-25Dec-strictNOHAX.t"
 Using relative burnin ('relburnin=yes'), discarding the first 25 % of sampled trees
 Writing statistics to files DY-25Dec-strictNOHAX.<parts|tstat|vstat|trprobs|con>
 Examining file ...
 Found one tree block in file "DY-25Dec-strictNOHAX.t" with 4001 trees in last block

 Tree reading status:

 0      10      20      30      40      50      60      70      80      90     100
 v-------v-------v-------v-------v-------v-------v-------v-------v-------v-------v
 *********************************************************************************

 Read 4001 trees from last tree block (sampling 3001 of them)

 General explanation:

 In an unrooted tree, a taxon bipartition (split) is specified by removing a
 branch, thereby dividing the species into those to the left and those to the
 right of the branch. Here, taxa to one side of the removed branch are denoted
 '.' and those to the other side are denoted '*'. Specifically, the '.' symbol
 is used for the taxa on the same side as the outgroup.

 In a rooted or clock tree, the tree is rooted using the model and not by
 reference to an outgroup. Each bipartition therefore corresponds to a clade,
 that is, a group that includes all the descendants of a particular branch in
 the tree.  Taxa that are included in each clade are denoted using '*', and
 taxa that are not included are denoted using the '.' symbol.

 The output first includes a key to all the bipartitions with frequency larger
 or equual to (Minpartfreq) in at least one run. Minpartfreq is a paramiter to
 sumt command and currently it is set to 0.10.  This is followed by a table
 with statistics for the informative bipartitions (those including at least
 two taxa), sorted from highest to lowest probability. For each bipartition,
 the table gives the number of times the partition or split was observed in all
```

runs (#obs) and the posterior probability of the bipartition (Probab.), which is the same as the split frequency. If several runs are summarized, this is followed by the minimum split frequency (Min(s)), the maximum frequency (Max(s)), and the standard deviation of frequencies (Stddev(s)) across runs. The latter value should approach 0 for all bipartitions as MCMC runs converge.

This is followed by a table summarizing branch lengths, node heights (if a clock model was used) and relaxed clock parameters (if a relaxed clock model was used). The mean, variance, and 95 % credible interval are given for each of these parameters. If several runs are summarized, the potential scale reduction factor (PSRF) is also given; it should approach 1 as runs converge. Node heights will take calibration points into account, if such points were used in the analysis.

Note that Stddev may be unreliable if the partition is not present in all runs (the last column indicates the number of runs that sampled the partition if more than one run is summarized). The PSRF is not calculated at all if the partition is not present in all runs.The PSRF is also sensitive to small sample sizes and it should only be considered a rough guide to convergence since some of the assumptions allowing one to interpret it as a true potential scale reduction factor are violated in MrBayes.

List of taxa in bipartitions:

```
   1 -- gwi
   2 -- dgr
   3 -- scsh
   4 -- xsl
   5 -- bea
   6 -- crx
   7 -- chp
   8 -- txc
   9 -- haa
  10 -- ing
  11 -- kuu
  12 -- hoi
  13 -- koy
  14 -- taa
  15 -- aht
  16 -- tfn
  17 -- kkz
  18 -- tcb
  19 -- tau
  20 -- ttmN
  21 -- tceS
  22 -- eya
  23 -- tli
  24 -- gce
  25 -- tol
  26 -- cco
  27 -- hup
  28 -- mtl
  29 -- wlk
  30 -- kto
  31 -- apc
  32 -- apw
  33 -- apj
  34 -- nav
  35 -- apk
```

```
  36 -- apl
  37 -- srs
  38 -- ket
  39 -- zko

Key to taxon bipartitions (saved to file "DY-25Dec-strictNOHAX.parts"):

 ID -- Partition
----------------------------------------------
  0 -- ***************************************
  1 -- *......................................
  2 -- .*.....................................
  3 -- ..*....................................
  4 -- ...*...................................
  5 -- ....*..................................
  6 -- .....*.................................
  7 -- ......*................................
  8 -- .......*...............................
  9 -- ........*..............................
 10 -- .........*.............................
 11 -- ..........*............................
 12 -- ...........*...........................
 13 -- ............*..........................
 14 -- .............*.........................
 15 -- ..............*........................
 16 -- ...............*.......................
 17 -- ................*......................
 18 -- .................*.....................
 19 -- ..................*....................
 20 -- ...................*...................
 21 -- ....................*..................
 22 -- .....................*.................
 23 -- ......................*................
 24 -- .......................*...............
 25 -- ........................*..............
 26 -- .........................*.............
 27 -- ..........................*............
 28 -- ...........................*...........
 29 -- ............................*..........
 30 -- .............................*.........
 31 -- ..............................*........
 32 -- ...............................*.......
 33 -- ................................*......
 34 -- .................................*.....
 35 -- ..................................*....
 36 -- ...................................*...
 37 -- ....................................*..
 38 -- .....................................*.
 39 -- ......................................*
 40 -- .....................................**
 41 -- ..................................**...
 42 -- ..............................*******..
 43 -- .....................**................
 44 -- .............*...**....................
 45 -- ............................**.........
 46 -- ..............................****.....
 47 -- .........*.**..........................
 48 -- ..........................****.........
 49 -- ..............................******...
```

```
50 -- ***.**.................................
51 -- ..............................***......
52 -- ...*..*.*....*..*****..................
53 -- ...*............*......................
54 -- ..........................****.......**
55 -- ..............................**.......
56 -- ....**.................................
57 -- ...........**..........................
58 -- ...*..*.*.......*..**..................
59 -- .............*...*.....................
60 -- .......................***.............
61 -- ......*............**..................
62 -- *******.*....*..*****..................
63 -- .......................**..............
64 -- *********************..***....*******..
65 -- ...................**..................
66 -- **************************....*******..
67 -- ..........................*.**.........
68 -- .........****..........................
69 -- ...........................***.........
70 -- ........................**.............
71 -- **.....................................
72 -- .................**....................
73 -- .**....................................
74 -- ..............**.......................
75 -- ...*..*.**.***..*****..................
76 -- *********************..................
77 -- ..*.**.................................
78 -- .........*.*...........................
79 -- ..*..*.................................
80 -- *********************.........*******..
81 -- .........*.***...**....................
82 -- .......................***....*******..
83 -- ...*....*.......*......................
84 -- *.*.**.................................
85 -- *...**.................................
86 -- *************************************..
87 -- ...............................**......
88 -- ..............*...............*******..
89 -- ......*.............*..................
90 -- ..............................*.*......
91 -- ..................................***..
92 -- ......*............*...................
93 -- ...*..*.........*..**..................
94 -- .......*.......*.......................
95 -- ***..*.................................
96 -- *******.*....*..*****.........*******..
97 -- .....................**...****.......**
98 -- *******.**.***..*****..................
99 -- ***....................................
100 -- .......*..*............................
101 -- .............*....*....................
102 -- .........*..*..........................
103 -- ...*...............*...................
104 -- ......*.*..........**..................
105 -- *******.*.......*..**..................
106 -- ..............................**.*.....
107 -- ***********************.......*******..
108 -- .......*......*........................
```

```
109 -- .........****............*.............
110 -- ..........................**...........
111 -- .......*......**.......................
112 -- ........*....*...**....................
113 -- *********************....*....*******..
114 -- ..........*....*.......................
----------------------------------------------
```

Summary statistics for informative taxon bipartitions (clades)
(saved to file "DY-25Dec-strictNOHAX.tstat"):

ID	#obs	Probab.
40	3001	1.000000
41	3001	1.000000
42	2784	0.927691
43	2711	0.903366
44	2584	0.861046
45	2541	0.846718
46	2519	0.839387
47	2342	0.780407
48	2334	0.777741
49	2173	0.724092
50	2132	0.710430
51	1993	0.664112
52	1920	0.639787
53	1840	0.613129
54	1809	0.602799
55	1804	0.601133
56	1779	0.592802
57	1639	0.546151
58	1629	0.542819
59	1627	0.542153
60	1624	0.541153
61	1554	0.517827
62	1535	0.511496
63	1422	0.473842
64	1381	0.460180
65	1338	0.445851
66	1256	0.418527
67	1249	0.416195
68	1196	0.398534
69	1187	0.395535
70	1180	0.393202
71	1134	0.377874
72	941	0.313562
73	930	0.309897
74	869	0.289570
75	845	0.281573
76	824	0.274575
77	808	0.269244
78	785	0.261579
79	736	0.245252
80	699	0.232922
81	697	0.232256
82	688	0.229257
83	660	0.219927
84	601	0.200267
85	573	0.190936

86	571	0.190270
87	557	0.185605
88	556	0.185272
89	555	0.184938
90	548	0.182606
91	526	0.175275
92	524	0.174608
93	520	0.173276
94	493	0.164279
95	469	0.156281
96	431	0.143619
97	429	0.142952
98	412	0.137288
99	401	0.133622
100	397	0.132289
101	385	0.128291
102	381	0.126958
103	373	0.124292
104	372	0.123959
105	363	0.120960
106	354	0.117961
107	345	0.114962
108	345	0.114962
109	335	0.111629
110	329	0.109630
111	327	0.108964
112	318	0.105965
113	315	0.104965
114	313	0.104299

Summary statistics for branch and node parameters
(saved to file "DY-25Dec-strictNOHAX.vstat"):

Parameter	Mean	Variance	95% HPD Interval Lower	Upper	Median
length[1]	0.047745	0.000397	0.012193	0.085901	0.045633
length[2]	0.049778	0.000533	0.011875	0.095301	0.046044
length[3]	0.043071	0.000362	0.011180	0.081444	0.040694
length[4]	0.033754	0.000242	0.007330	0.063727	0.031843
length[5]	0.040812	0.000601	0.005040	0.090257	0.035076
length[6]	0.032351	0.000250	0.005307	0.063883	0.030330
length[7]	0.043983	0.000278	0.014654	0.076065	0.042258
length[8]	0.098026	0.000954	0.034813	0.153322	0.098731
length[9]	0.047818	0.000243	0.015838	0.075966	0.047380
length[10]	0.048222	0.000497	0.009746	0.090153	0.044319
length[11]	0.077783	0.000976	0.018206	0.136765	0.076645
length[12]	0.035971	0.000260	0.008248	0.066786	0.034006
length[13]	0.036680	0.000282	0.007374	0.068520	0.034504
length[14]	0.027762	0.000223	0.004573	0.058179	0.024764
length[15]	0.095056	0.000962	0.034479	0.153684	0.094637
length[16]	0.091624	0.000941	0.029883	0.145360	0.091530
length[17]	0.035727	0.000253	0.007822	0.066391	0.033831
length[18]	0.024754	0.000182	0.003119	0.051212	0.022009
length[19]	0.030594	0.000251	0.004945	0.061505	0.027774
length[20]	0.033979	0.000207	0.008007	0.061703	0.032561
length[21]	0.035973	0.000216	0.008395	0.063505	0.034805

length[22]	0.096436	0.001113	0.033295	0.162128	0.093967
length[23]	0.098471	0.001297	0.033295	0.174096	0.094757
length[24]	0.084747	0.001057	0.023190	0.145752	0.081983
length[25]	0.074344	0.000896	0.021608	0.135425	0.070490
length[26]	0.082588	0.001041	0.022756	0.144016	0.078882
length[27]	0.090931	0.001161	0.028041	0.155953	0.087646
length[28]	0.089269	0.001050	0.025352	0.148093	0.087852
length[29]	0.045199	0.000442	0.010522	0.086571	0.041672
length[30]	0.046070	0.000506	0.009128	0.088738	0.041893
length[31]	0.009049	0.000068	0.000008	0.025752	0.006545
length[32]	0.009029	0.000067	0.000008	0.025733	0.006585
length[33]	0.015090	0.000107	0.000150	0.033786	0.013283
length[34]	0.029360	0.000203	0.006176	0.056253	0.027332
length[35]	0.005409	0.000033	0.000001	0.016819	0.003675
length[36]	0.005409	0.000033	0.000001	0.016819	0.003675
length[37]	0.064516	0.000621	0.021164	0.115220	0.062793
length[38]	0.068721	0.000499	0.020120	0.108231	0.067288
length[39]	0.068721	0.000499	0.020120	0.108231	0.067288
length[40]	0.078948	0.000766	0.025699	0.130694	0.076875
length[41]	0.037808	0.000296	0.006534	0.069679	0.036135
length[42]	0.043431	0.000506	0.002170	0.085166	0.041100
length[43]	0.054738	0.000744	0.003005	0.103709	0.052842
length[44]	0.027150	0.000232	0.001245	0.055630	0.025221
length[45]	0.038945	0.000562	0.001805	0.084212	0.035141
length[46]	0.019598	0.000174	0.000277	0.045492	0.016943
length[47]	0.031059	0.000416	0.000834	0.070308	0.026727
length[48]	0.041952	0.000762	0.000041	0.092359	0.037808
length[49]	0.026481	0.000328	0.000010	0.060815	0.023169
length[50]	0.025144	0.000354	0.000000	0.061014	0.021503
length[51]	0.015595	0.000136	0.000035	0.037948	0.012802
length[52]	0.021806	0.000144	0.001635	0.043750	0.020126
length[53]	0.019903	0.000170	0.000058	0.044614	0.017497
length[54]	0.021216	0.000171	0.000307	0.046973	0.019083
length[55]	0.013071	0.000103	0.000010	0.032335	0.010608
length[56]	0.026425	0.000306	0.000077	0.059736	0.023153
length[57]	0.020015	0.000212	0.000000	0.048973	0.017161
length[58]	0.017708	0.000152	0.000028	0.041109	0.015616
length[59]	0.012539	0.000124	0.000002	0.033673	0.009609
length[60]	0.035596	0.000516	0.000057	0.076962	0.031963
length[61]	0.012066	0.000067	0.000035	0.026753	0.010349
length[62]	0.030673	0.000282	0.000082	0.059964	0.028927
length[63]	0.039302	0.000699	0.000022	0.088364	0.033813
length[64]	0.018177	0.000162	0.000007	0.041267	0.015395
length[65]	0.013279	0.000115	0.000034	0.032791	0.010841
length[66]	0.014033	0.000114	0.000000	0.034335	0.011538
length[67]	0.030355	0.000451	0.000166	0.074401	0.026654
length[68]	0.032829	0.000478	0.000401	0.075776	0.028367
length[69]	0.033600	0.000614	0.000007	0.082183	0.027659
length[70]	0.035638	0.000590	0.000344	0.082215	0.030678
length[71]	0.022881	0.000216	0.000108	0.048601	0.020829
length[72]	0.014831	0.000207	0.000005	0.043495	0.010513
length[73]	0.022379	0.000207	0.001286	0.051233	0.019563
length[74]	0.028751	0.000399	0.000023	0.067547	0.025319
length[75]	0.024530	0.000182	0.001824	0.048269	0.022277
length[76]	0.015502	0.000132	0.000192	0.037206	0.012693
length[77]	0.021196	0.000260	0.000201	0.052896	0.018138
length[78]	0.012706	0.000125	0.000014	0.034066	0.009854
length[79]	0.018969	0.000197	0.000022	0.044661	0.015658
length[80]	0.015601	0.000144	0.000013	0.037268	0.012381

length[81]	0.022881	0.000314	0.000033	0.057784	0.017572
length[82]	0.022380	0.000231	0.000009	0.051348	0.019434
length[83]	0.009999	0.000079	0.000014	0.028500	0.007436
length[84]	0.021881	0.000214	0.000369	0.051007	0.018568
length[85]	0.018607	0.000215	0.000117	0.045925	0.015094
length[86]	0.014686	0.000178	0.000073	0.041646	0.010910
length[87]	0.007390	0.000051	0.000002	0.020994	0.005171
length[88]	0.026450	0.000269	0.000121	0.055423	0.023479
length[89]	0.007985	0.000063	0.000002	0.025727	0.005415
length[90]	0.007679	0.000051	0.000039	0.021924	0.005402
length[91]	0.015195	0.000141	0.000039	0.038210	0.012350
length[92]	0.008481	0.000065	0.000001	0.023178	0.006031
length[93]	0.007473	0.000049	0.000002	0.020801	0.005320
length[94]	0.021296	0.000297	0.000023	0.054232	0.016988
length[95]	0.019179	0.000207	0.000051	0.049124	0.015973
length[96]	0.017382	0.000136	0.000043	0.039105	0.016118
length[97]	0.009117	0.000055	0.000038	0.023782	0.007327
length[98]	0.016307	0.000146	0.000053	0.040050	0.012861
length[99]	0.012157	0.000124	0.000027	0.032898	0.008471
length[100]	0.023908	0.000341	0.000049	0.057715	0.020063
length[101]	0.008470	0.000092	0.000008	0.028994	0.005377
length[102]	0.009177	0.000088	0.000052	0.026222	0.006538
length[103]	0.018680	0.000164	0.000354	0.044446	0.015757
length[104]	0.006476	0.000044	0.000088	0.020659	0.004306
length[105]	0.032742	0.000323	0.002786	0.064638	0.030082
length[106]	0.006786	0.000049	0.000002	0.020563	0.004338
length[107]	0.010552	0.000062	0.000090	0.023547	0.009176
length[108]	0.020489	0.000259	0.000002	0.053071	0.016829
length[109]	0.032602	0.000422	0.000151	0.070349	0.029065
length[110]	0.017008	0.000265	0.000028	0.050733	0.011978
length[111]	0.017787	0.000238	0.000155	0.047918	0.014802
length[112]	0.010261	0.000078	0.000025	0.027335	0.007870
length[113]	0.016491	0.000145	0.000001	0.038375	0.014200
length[114]	0.022781	0.000343	0.000144	0.060629	0.018659
height[0]	0.167205	0.001492	0.064714	0.226702	0.171668
height[1]	0.000000	0.000000	0.000000	0.000000	0.000000
height[2]	0.000000	0.000000	0.000000	0.000000	0.000000
height[3]	0.000000	0.000000	0.000000	0.000000	0.000000
height[4]	0.000000	0.000000	0.000000	0.000000	0.000000
height[5]	0.000000	0.000000	0.000000	0.000000	0.000000
height[6]	0.000000	0.000000	0.000000	0.000000	0.000000
height[7]	0.000000	0.000000	0.000000	0.000000	0.000000
height[8]	0.000000	0.000000	0.000000	0.000000	0.000000
height[9]	0.000000	0.000000	0.000000	0.000000	0.000000
height[10]	0.000000	0.000000	0.000000	0.000000	0.000000
height[11]	0.000000	0.000000	0.000000	0.000000	0.000000
height[12]	0.000000	0.000000	0.000000	0.000000	0.000000
height[13]	0.000000	0.000000	0.000000	0.000000	0.000000
height[14]	0.000000	0.000000	0.000000	0.000000	0.000000
height[15]	0.000000	0.000000	0.000000	0.000000	0.000000
height[16]	0.000000	0.000000	0.000000	0.000000	0.000000
height[17]	0.000000	0.000000	0.000000	0.000000	0.000000
height[18]	0.000000	0.000000	0.000000	0.000000	0.000000
height[19]	0.000000	0.000000	0.000000	0.000000	0.000000
height[20]	0.000000	0.000000	0.000000	0.000000	0.000000
height[21]	0.000000	0.000000	0.000000	0.000000	0.000000
height[22]	0.000000	0.000000	0.000000	0.000000	0.000000
height[23]	0.000000	0.000000	0.000000	0.000000	0.000000
height[24]	0.000000	0.000000	0.000000	0.000000	0.000000

height[25]	0.000000	0.000000	0.000000	0.000000	0.000000
height[26]	0.000000	0.000000	0.000000	0.000000	0.000000
height[27]	0.000000	0.000000	0.000000	0.000000	0.000000
height[28]	0.000000	0.000000	0.000000	0.000000	0.000000
height[29]	0.000000	0.000000	0.000000	0.000000	0.000000
height[30]	0.000000	0.000000	0.000000	0.000000	0.000000
height[31]	0.000000	0.000000	0.000000	0.000000	0.000000
height[32]	0.000000	0.000000	0.000000	0.000000	0.000000
height[33]	0.000000	0.000000	0.000000	0.000000	0.000000
height[34]	0.000000	0.000000	0.000000	0.000000	0.000000
height[35]	0.000000	0.000000	0.000000	0.000000	0.000000
height[36]	0.000000	0.000000	0.000000	0.000000	0.000000
height[37]	0.000000	0.000000	0.000000	0.000000	0.000000
height[38]	0.000000	0.000000	0.000000	0.000000	0.000000
height[39]	0.000000	0.000000	0.000000	0.000000	0.000000
height[40]	0.068721	0.000499	0.020120	0.108231	0.067288
height[41]	0.005409	0.000033	0.000001	0.016819	0.003675
height[42]	0.066068	0.000460	0.023255	0.107670	0.065163
height[43]	0.093094	0.000967	0.031159	0.150516	0.091382
height[44]	0.032751	0.000195	0.009594	0.061247	0.030640
height[45]	0.041491	0.000312	0.010309	0.075752	0.039546
height[46]	0.028614	0.000139	0.007752	0.051422	0.027382
height[47]	0.048160	0.000328	0.013861	0.082856	0.046490
height[48]	0.102051	0.000939	0.033904	0.156231	0.101894
height[49]	0.044083	0.000245	0.014362	0.072970	0.042818
height[50]	0.067635	0.000394	0.025494	0.101953	0.068815
height[51]	0.014883	0.000072	0.001696	0.030505	0.013577
height[52]	0.065575	0.000278	0.028271	0.095905	0.065644
height[53]	0.029567	0.000161	0.007006	0.054390	0.027800
height[54]	0.138499	0.001136	0.056367	0.196568	0.140161
height[55]	0.006196	0.000034	0.000008	0.017342	0.004467
height[56]	0.028441	0.000175	0.006407	0.054938	0.026317
height[57]	0.031869	0.000194	0.007556	0.059325	0.030383
height[58]	0.055037	0.000241	0.023028	0.084464	0.054538
height[59]	0.022243	0.000123	0.003119	0.044169	0.020116
height[60]	0.094014	0.000792	0.039607	0.145940	0.094147
height[61]	0.040063	0.000170	0.016169	0.064510	0.039496
height[62]	0.089967	0.000444	0.042770	0.130448	0.091306
height[63]	0.071851	0.000714	0.024197	0.124905	0.069595
height[64]	0.139751	0.000944	0.066074	0.195552	0.143003
height[65]	0.029817	0.000156	0.008111	0.055325	0.028712
height[66]	0.152294	0.001113	0.075566	0.212945	0.156844
height[67]	0.075794	0.000602	0.028172	0.122614	0.074579
height[68]	0.075022	0.000607	0.025254	0.118959	0.074053
height[69]	0.076377	0.000605	0.025417	0.121906	0.075703
height[70]	0.067284	0.000576	0.022105	0.111580	0.064454
height[71]	0.040578	0.000298	0.008586	0.073806	0.038921
height[72]	0.024041	0.000163	0.003347	0.049360	0.021855
height[73]	0.040567	0.000267	0.011390	0.071229	0.038506
height[74]	0.079740	0.000707	0.034338	0.136401	0.078959
height[75]	0.079515	0.000510	0.027455	0.118641	0.079666
height[76]	0.122705	0.000711	0.065935	0.168773	0.125567
height[77]	0.046325	0.000286	0.013999	0.076954	0.045748
height[78]	0.036370	0.000226	0.008586	0.063334	0.035161
height[79]	0.031399	0.000190	0.007349	0.059235	0.030636
height[80]	0.125868	0.000969	0.052778	0.173624	0.131379
height[81]	0.066169	0.000439	0.020229	0.104472	0.064944
height[82]	0.116137	0.000708	0.061777	0.168776	0.117383
height[83]	0.044714	0.000187	0.021529	0.076030	0.044518

height[84]	0.055646	0.000304	0.022615	0.088766	0.053772
height[85]	0.049451	0.000286	0.014809	0.080123	0.047656
height[86]	0.163766	0.001472	0.073452	0.233565	0.168505
height[87]	0.008914	0.000047	0.000150	0.022318	0.007113
height[88]	0.092677	0.000671	0.035970	0.140772	0.092801
height[89]	0.036326	0.000166	0.014451	0.064632	0.035690
height[90]	0.009052	0.000044	0.000299	0.021780	0.007337
height[91]	0.045046	0.000258	0.015595	0.074431	0.044284
height[92]	0.036242	0.000160	0.013659	0.059686	0.035125
height[93]	0.050038	0.000216	0.019222	0.074613	0.049952
height[94]	0.085524	0.000675	0.034813	0.135786	0.085037
height[95]	0.056276	0.000366	0.021489	0.095489	0.055095
height[96]	0.112398	0.000808	0.050272	0.164706	0.114740
height[97]	0.154441	0.001031	0.078728	0.214931	0.158091
height[98]	0.098028	0.000791	0.033909	0.140934	0.099526
height[99]	0.054545	0.000306	0.021656	0.084401	0.053863
height[100]	0.076106	0.000682	0.030469	0.125618	0.076927
height[101]	0.026118	0.000144	0.007708	0.053814	0.024012
height[102]	0.036828	0.000200	0.008715	0.062788	0.035531
height[103]	0.027954	0.000160	0.006132	0.052216	0.026366
height[104]	0.048838	0.000178	0.018887	0.069881	0.049055
height[105]	0.081396	0.000339	0.044713	0.119850	0.081532
height[106]	0.022686	0.000097	0.006773	0.040808	0.021766
height[107]	0.137247	0.001148	0.057494	0.187945	0.143259
height[108]	0.089987	0.000650	0.039422	0.144913	0.089487
height[109]	0.087314	0.000569	0.037736	0.135565	0.088147
height[110]	0.081851	0.000821	0.031732	0.132058	0.080694
height[111]	0.098839	0.000638	0.050438	0.149961	0.099060
height[112]	0.050226	0.000205	0.024122	0.078019	0.051091
height[113]	0.134872	0.000845	0.073295	0.186875	0.140082
height[114]	0.076121	0.000753	0.025953	0.130851	0.073749

Clade credibility values:

```
/--------------------------------------------------------------------- txc (8)
|
|--------------------------------------------------------------------- kuu (11)
|
|--------------------------------------------------------------------- aht (15)
|
|--------------------------------------------------------------------- tfn (16)
|
|                                                         /----------- apc (31)
|                                             /-----60----+
|                                             |           \----------- apw (32)
|                                  /----66----+
|                                  |          \----------------------- apj (33)
|                      /-----84----+
|                      |           \---------------------------------- nav (34)
|           /----72----+
|           |          |                                  /----------- apk (35)
|           |          \----------------100---------------+
|-----93----+                                             \----------- apl (36)
|           |
|           \--------------------------------------------------------- srs (37)
```

```
|
|                                                         /----------- eya (22)
|----------------------------90---------------------------+
|                                                         \----------- tli (23)
|
|                                             /----------------------- ing (10)
|                                             |
|----------------------78---------------------+           /----------- hoi (12)
|                                             \-----55----+
|                                                         \----------- koy (13)
|
+                                             /----------------------- hup (27)
|                                             |
|                                             |----------------------- mtl (28)
|                                  /----78----+
|                                  |          |           /----------- wlk (29)
|                                  |          \-----85----+
|----------------60----------------+                      \----------- kto (30)
|                                  |
|                                  |                      /----------- ket (38)
|                                  \----------100---------+
|                                                         \----------- zko (39)
|
|                                                         /----------- gce (24)
|                                                         |
|----------------------------54---------------------------+----------- tol (25)
|                                                         |
|                                                         \----------- cco (26)
|
|                                             /----------------------- gwi (1)
|                                             |
|                                             |----------------------- dgr (2)
|                                             |
|                      /----------71----------+----------------------- scsh (3)
|                      |                      |
|                      |                      |           /----------- bea (5)
|                      |                      \-----59----+
|                      |                                  \----------- crx (6)
|                      |
|                      |                                  /----------- xsl (4)
|                      |                      /-----61----+
|                      |                      |           \----------- kkz (17)
\----------51----------+                      |
                       |                      |           /----------- chp (7)
                       |                      |           |
                       |           /----54----+-----52----+----------- ttmN (20)
                       |           |          |           |
                       |           |          |           \----------- tceS (21)
                       |           |          |
                       |           |          \----------------------- haa (9)
                       \-----64----+
                                   |                      /----------- taa (14)
                                   |          /-----54----+
                                   |          |           \----------- tcb (18)
                                   \----86----+
                                              \----------------------- tau (19)
```

Phylogram (based on median node depths):

```
/-------------------------------------------------------------------- txc (8)
|
|-------------------------------------------------------------------- kuu (11)
|
|-------------------------------------------------------------------- aht (15)
|
|-------------------------------------------------------------------- tfn (16)
|
|                                                                 /-- apc (31)
|                                                              /--+
|                                                              |  \-- apw (32)
|                                                        /-----+
|                                                        |     \----- apj (33)
|                                                  /-----+
|                                                  |     \----------- nav (34)
|                                         /--------+
|                                         |        |               /- apk (35)
|                                         |        \---------------+
|-----------------------------------------+                        \- apl (36)
|                                         |
|                                         \-------------------------- srs (37)
|
|                              /------------------------------------- eya (22)
|------------------------------+
|                              \------------------------------------- tli (23)
|
|                                                /------------------- ing (10)
|                                                |
|------------------------------------------------+      /------------ hoi (12)
|                                                \------+
|                                                       \------------ koy (13)
|
+                          /----------------------------------------- hup (27)
|                          |
|                          |----------------------------------------- mtl (28)
|           /--------------+
|           |              |                        /---------------- wlk (29)
|           |              \------------------------+
|------------+                                      \---------------- kto (30)
|           |
|           |                            /--------------------------- ket (38)
|           \----------------------------+
|                                        \--------------------------- zko (39)
|
|                             /-------------------------------------- gce (24)
|                             |
|-----------------------------+-------------------------------------- tol (25)
|                             |
|                             \-------------------------------------- cco (26)
|
|                                       /---------------------------- gwi (1)
|                                       |
|                                       |---------------------------- dgr (2)
|                                       |
|                              /--------+---------------------------- scsh (3)
|                              |        |
|                              |        |                /----------- bea (5)
|                              |        \----------------+
```

```
   |                              |                         \----------- crx (6)
   |                              |
   |                              |                         /----------- xsl (4)
   |                              |              /----------+
   |                              |              |          \----------- kkz (17)
   \------------------------------+              |
                                  |              |     /---------------- chp (7)
                                  |              |     |
                                  |          /---+-----+---------------- ttmN (20)
                                  |          |   |     |
                                  |          |   |     \---------------- tceS (21)
                                  |          |   |
                                  |          |   \---------------------- haa (9)
                                  \----------+
                                             |                  /-------- taa (14)
                                             |             /---+
                                             |             |   \-------- tcb (18)
                                             \-------------+
                                                           \------------ tau (19)

   ---------|-------------------|-------------------|-------------------|
          0.15                0.10                0.05                0.00

   [Expected changes per site]

   Calculating tree probabilities...

   Credible sets of trees (3001 trees sampled):
      50 % credible set contains 1501 trees
      90 % credible set contains 2701 trees
      95 % credible set contains 2851 trees
      99 % credible set contains 2971 trees


MrBayes > sump relburnin=yes

   Using relative burnin (a fraction of samples discarded).
   Summarizing parameters in file DY-25Dec-strictNOHAX.p
   Writing summary statistics to file DY-25Dec-strictNOHAX.pstat
   Using relative burnin ('relburnin=yes'), discarding the first 25 % of samples

   Below is a rough plot of the generation (x-axis) versus the log
   probability of observing the data (y-axis). You can use this
   graph to determine what the burn in for your analysis should be.
   When the log probability starts to plateau you may be at station-
   arity. Sample trees and parameters after the log probability
   plateaus. Of course, this is not a guarantee that you are at sta-
   analysis should be. When the log probability starts to plateau
   tionarity. When possible, run multiple analyses starting from dif-
   ferent random trees; if the inferences you make for independent
   analyses are the same, this is reasonable evidence that the chains
   have converged. You can use MrBayes to run several independent
   analyses simultaneously. During such a run, MrBayes will monitor
   the convergence of topologies. After the run has been completed,
   the 'sumt' and 'sump' functions will provide additional conver-
   gence diagnostics for all the parameters in your model. Remember
   that the burn in is the number of samples to discard. There are
   a total of ngen / samplefreq samples taken during a MCMC analysis.
```

```
+------------------------------------------------------------+ -939.946
|      *                                    *                |
|                                                        * * |
|     *             *    *                                   |
|         *     *                 *                          |
|       **  *  *              *     **   **  *  *   *        |
|             *                                  *      * *  |
|                            *         *   *           *     |
|  **      *            *       *  *  *           *   *      |
|            *        *    *                  *              |
|                * *             *      *                   *|
|                    *    * *  *                   *         |
|**                                            *             |
|    *                 *                                     |
|                                                    *       |
|                 *                                          |
+------+-----+-----+-----+-----+-----+-----+-----+-----+-----+ -945.476
^                                                            ^
500000                                                      2000000
```

Estimated marginal likelihoods for run sampled in file "DY-25Dec-strictNOHAX.p":
(Use the harmonic mean for Bayes factor comparisons of models)
(Values are saved to the file /Users/msicoli/Yeniseian-NaDene-Typlogical_noHAX.nex.lstat)

Arithmetic mean	Harmonic mean
-929.96	-965.14

Model parameter summaries for run sampled in file "DY-25Dec-strictNOHAX":
Based on a total of 3001 samples out of a total of 4001 samples
from this analysis.
Parameter summaries saved to file "/Users/msicoli/Yeniseian-NaDene-Typlogical_noHAX.nex.pstat".

Parameter	Mean	Variance	95% HPD Interval Lower	Upper	Median	ESS*
TH	0.167205	0.001492	0.064714	0.226702	0.171668	319.44
TL	2.854755	0.366821	1.394540	3.892517	2.963303	284.19
alpha	9.210757	772.797996	0.087496	56.401044	1.903306	2500.96

* Convergence diagnostic (ESS = Estimated Sample Size); ESS value
below 100 may indicate that the parameter is undersampled.

MrBayes > ssp ngen-100000 diagnfreq=1000 filename=YND-Typ-NoHax-ss

Could not find parameter "ngen-100000"

MrBayes > ssp ngen=100000 diagnfreq=1000 filename=YND-Typ-NoHax-ss

Setting number of generations to 100000
Setting diagnosing frequency to 1000

```
MrBayes > ss

   Setting chain output file names to "YND-Typ-NoHax-ss.<p/t>"
   Running Markov chain
   MCMC stamp = 7908113950
   Seed = 1878978649
   Swapseed = 1031120143
   Model settings:

      Data not partitioned --
         Datatype  = Standard
         Coding    = Variable
         # States  = Variable, up to 10
                     State frequencies are fixed to be equal
         Rates     = Gamma
                     Gamma shape parameter is uniformly dist-
                     ributed on the interval (0.00,200.00).
                     Gamma distribution is approximated using 4 categories.
                     Likelihood summarized over all rate categories in each generation.

   Active parameters:

      Parameters
      ------------------
      Statefreq        1
      Shape            2
      Ratemultiplier   3
      Topology         4
      Brlens           5
      Clockrate        6
      ------------------

      1 --  Parameter  = Alpha_symdir
            Type       = Symmetric diricihlet/beta distribution alpha_i parameter
            Prior      = Symmetric dirichlet with fixed(-1.00) variance parameter

      2 --  Parameter  = Alpha
            Type       = Shape of scaled gamma distribution of site rates
            Prior      = Uniform(0.00,200.00)

      3 --  Parameter  = Ratemultiplier
            Type       = Partition-specific rate multiplier
            Prior      = Fixed(1.0)

      4 --  Parameter  = Tau
            Type       = Topology
            Prior      = All topologies equally probable a priori
            Subparam.  = V

      5 --  Parameter  = V
            Type       = Branch lengths
            Prior      = Clock:Uniform
                         Tree age has an Exponential(1.000) distribution
                         Node ages are not constrained

      6 --  Parameter  = Clockrate
            Type       = Base rate of clock
            Prior      = Fixed(1.000000)
                         The clock rate is constant (strict clock)
```

```
Number of taxa = 39
Number of characters = 116

The MCMC sampler will use the following moves:
   With prob.  Chain will use move
      2.38 %   Multiplier(Alpha)
     11.90 %   ExtSprClock(Tau,V)
     23.81 %   NNIClock(Tau,V)
     11.90 %   ParsSPRClock(Tau,V)
     47.62 %   NodesliderClock(V)
      2.38 %   TreeStretch(V)

Division 1 has 78 unique site patterns
Initializing conditional likelihoods
Using standard non-SSE likelihood calculator for division 1 (single-precision)

Initial log likelihoods and log prior probs:
   Chain 1 -- -937.554390 -- -17.664652
   Chain 2 -- -941.921722 -- -36.467720
   Chain 3 -- -945.920556 -- -31.420523
   Chain 4 -- -968.722535 -- -27.744449


Starting stepping-stone sampling to estimate marginal likelihood.
50 steps will be used with 1500 generations (3 samples) within each step.
Total of 76500 generations (153 samples) will be collected while first
1500 generations (3 samples) will be discarded as initial burnin.
Additionally at the begining of each step 0 generations (0 samples)
will be discarded as burnin.
Sampling from posterior to prior, i.e. first step samples from close to
posterior.
NOTE: Number of generation of each step is reduced to the closest multiple
of sampling frequency. That is why, in total it will be taken 76500 gene-
rations instead of requested 100000.


Chain results (76500 generations requested):

    0 -- [-937.554] (-941.922) (-945.921) (-968.723)

Sampling step 1 out of 50 steps...

Sampling step 2 out of 50 steps...

Sampling step 3 out of 50 steps...

Sampling step 4 out of 50 steps...

Sampling step 5 out of 50 steps...

Sampling step 6 out of 50 steps...

10000 -- (-966.909) [-971.576] (-960.096) (-1012.739) -- 0:00:33
Sampling step 7 out of 50 steps...

Sampling step 8 out of 50 steps...
```

```
Sampling step 9 out of 50 steps...

Sampling step 10 out of 50 steps...

Sampling step 11 out of 50 steps...

Sampling step 12 out of 50 steps...

Sampling step 13 out of 50 steps...

20000 -- (-1040.425) (-1044.518) [-1011.347] (-1010.900) -- 0:00:28
Sampling step 14 out of 50 steps...

Sampling step 15 out of 50 steps...

Sampling step 16 out of 50 steps...

Sampling step 17 out of 50 steps...

Sampling step 18 out of 50 steps...

Sampling step 19 out of 50 steps...

30000 -- [-1056.473] (-1214.950) (-1120.707) (-1102.046) -- 0:00:23
Sampling step 20 out of 50 steps...

Sampling step 21 out of 50 steps...

Sampling step 22 out of 50 steps...

Sampling step 23 out of 50 steps...

Sampling step 24 out of 50 steps...

Sampling step 25 out of 50 steps...

Sampling step 26 out of 50 steps...

40000 -- (-1295.848) (-1294.247) [-1179.106] (-1254.753) -- 0:00:18
Sampling step 27 out of 50 steps...

Sampling step 28 out of 50 steps...

Sampling step 29 out of 50 steps...

Sampling step 30 out of 50 steps...

Sampling step 31 out of 50 steps...

Sampling step 32 out of 50 steps...

Sampling step 33 out of 50 steps...

50000 -- (-1389.502) (-1378.487) (-1326.617) [-1299.880] -- 0:00:13
Sampling step 34 out of 50 steps...

Sampling step 35 out of 50 steps...

Sampling step 36 out of 50 steps...
```

```
Sampling step 37 out of 50 steps...

Sampling step 38 out of 50 steps...

Sampling step 39 out of 50 steps...

60000 -- (-1391.943) (-1388.422) (-1364.142) [-1477.828] -- 0:00:08
Sampling step 40 out of 50 steps...

Sampling step 41 out of 50 steps...

Sampling step 42 out of 50 steps...

Sampling step 43 out of 50 steps...

Sampling step 44 out of 50 steps...

Sampling step 45 out of 50 steps...

Sampling step 46 out of 50 steps...

70000 -- (-1987.933) (-2082.221) (-2084.316) [-1834.352] -- 0:00:03
Sampling step 47 out of 50 steps...

Sampling step 48 out of 50 steps...

Sampling step 49 out of 50 steps...

Sampling step 50 out of 50 steps...


Analysis completed in 39 seconds
Analysis used 37.13 seconds of CPU time
Log likelihood of best state for "cold" chain was -929.82

Marginal likelihood (in natural log units) estimated using stepping-stone sampling based on
50 steps with 1500 generations (3 samples) within each step.

    Run   Marginal likelihood (ln)
    ------------------------------
      1     -1050.61
    ------------------------------
More statistics on stepping-stone sampling is dumped to YND-Typ-NoHax-ss.ss file.

Plot of average standard deviation of split frequencies across steps.
Points at -1.0 (y-axis) indicate that there were no splits
above minimum frequency for corresponding step.
+------------------------------------------------------------+ 0.000
|                                                            |
|                                                            |
|                                                            |
|                                                            |
|                                                            |
|                                                            |
|                                                            |
|                                                            |
|                                                            |
|                                                            |
```

```
|                                                            |
|                                                            |
|                                                            |
|                                                            |
|***** **** ***** **** ***** **** ***** **** ***** **** *****|
+------+-----+-----+-----+-----+-----+-----+-----+-----+-----+ 0.000
^                                                            ^
1                                                           50


Acceptance rates for the moves in the "cold" chain:
   With prob.   (last 100)   chain accepted proposals by move
      44.6 %     ( 36 %)     Multiplier(Alpha)
      43.5 %     ( 63 %)     ExtSprClock(Tau,V)
      78.2 %     (100 %)     NNIClock(Tau,V)
      33.3 %     ( 35 %)     ParsSPRClock(Tau,V)
      85.7 %     ( 99 %)     NodesliderClock(V)
      52.1 %     ( 80 %)     TreeStretch(V)

Chain swap information:

            1      2      3      4
     ------------------------------
   1 |          0.25   0.07   0.02
   2 |  12661          0.32   0.10
   3 |  12779  12794          0.40
   4 |  12805  12805  12656

Upper diagonal: Proportion of successful state exchanges between chains
Lower diagonal: Number of attempted state exchanges between chains

Chain information:

  ID -- Heat
 -----------
   1 -- 1.00  (cold chain)
   2 -- 0.91
   3 -- 0.83
   4 -- 0.77

Heat = 1 / (1 + T * (ID - 1))
   (where T = 0.10 is the temperature and ID is the chain number)


MrBayes >
```

```
                            MrBayes v3.2.1 x64

                      (Bayesian Analysis of Phylogeny)

              Distributed under the GNU General Public License


               Type "help" or "help <command>" for information
                     on the commands that are available.

                   Type "about" for authorship and general
                       information about the program.


MrBayes > exe /Users/msicoli/Ket-NaDene-Typlogical_noHAX.nex

   Executing file "/Users/msicoli/Ket-NaDene-Typlogical_noHAX.nex"
   UNIX line termination
   Longest line length = 123
   Parsing file
   Expecting NEXUS formatted file
   Reading data block
      Allocated taxon set
      Allocated matrix
      Defining new matrix with 38 taxa and 116 characters
      Data is Standard
      Missing data coded as ?
      Gaps coded as -
      Data matrix is not interleaved
      Taxon  1 -> gwi
      Taxon  2 -> dgr
      Taxon  3 -> scsh
      Taxon  4 -> xsl
      Taxon  5 -> bea
      Taxon  6 -> crx
      Taxon  7 -> chp
      Taxon  8 -> txc
      Taxon  9 -> haa
      Taxon 10 -> ing
      Taxon 11 -> kuu
      Taxon 12 -> hoi
      Taxon 13 -> koy
      Taxon 14 -> taa
      Taxon 15 -> aht
      Taxon 16 -> tfn
      Taxon 17 -> kkz
      Taxon 18 -> tcb
      Taxon 19 -> tau
      Taxon 20 -> ttmN
      Taxon 21 -> tceS
      Taxon 22 -> eya
      Taxon 23 -> tli
      Taxon 24 -> gce
      Taxon 25 -> tol
      Taxon 26 -> cco
      Taxon 27 -> hup
      Taxon 28 -> mtl
      Taxon 29 -> wlk
      Taxon 30 -> kto
```

```
      Taxon 31 -> apc
      Taxon 32 -> apw
      Taxon 33 -> apj
      Taxon 34 -> nav
      Taxon 35 -> apk
      Taxon 36 -> apl
      Taxon 37 -> srs
      Taxon 38 -> ket
      Successfully read matrix
      Setting default partition (does not divide up characters)
      Setting model defaults
      Seed (for generating default start values) = 1388163552
      Adding dummy characters (unobserved site patterns) for division 1
      WARNING: There are 38 characters incompatible with the specified
               coding bias. These characters will be excluded.
      Setting output file names to "/Users/msicoli/Ket-NaDene-Typlogical_noHAX.nex.run<i>.<p|t>"
   Exiting data block
   Reached end of file

MrBayes > lset nst=6 rates=gamma

   Setting Rates to Gamma
   Successfully set likelihood model parameters
   Adding dummy characters (unobserved site patterns) for division 1
   WARNING: There are 38 characters incompatible with the specified
            coding bias. These characters will be excluded.

MrBayes > prset brlenspr=clock:uniform

   Setting Brlenspr to Clock:Uniform
   Successfully set prior model parameters
   Adding dummy characters (unobserved site patterns) for division 1
   WARNING: There are 38 characters incompatible with the specified
            coding bias. These characters will be excluded.

MrBayes > mcmcp ngen=2000000 printfreq=10000 samplefreq=500 nruns=1 nchains=4 savebrlens=yes
filename=DY-27Dec-strict-H-KottOut

   Setting number of generations to 2000000
   Setting print frequency to 10000
   Setting sample frequency to 500
   Setting number of runs to 1
   Setting number of chains to 4
   Setting chain output file names to "DY-27Dec-strict-H-KottOut.<p/t>"
   Successfully set chain parameters

MrBayes > mcmc

   Running Markov chain
   MCMC stamp = 2292685654
   Seed = 2059386170
   Swapseed = 1388163552
   Model settings:

      Data not partitioned --
         Datatype  = Standard
         Coding    = Variable
         # States  = Variable, up to 10
                     State frequencies are fixed to be equal
         Rates     = Gamma
                     Gamma shape parameter is uniformly dist-
```

```
                  ributed on the interval (0.00,200.00).
                  Gamma distribution is approximated using 4 categories.
                  Likelihood summarized over all rate categories in each generation.

Active parameters:

   Parameters
   ------------------
   Statefreq        1
   Shape            2
   Ratemultiplier   3
   Topology         4
   Brlens           5
   Clockrate        6
   ------------------

   1 --  Parameter  = Alpha_symdir
         Type       = Symmetric diricihlet/beta distribution alpha_i parameter
         Prior      = Symmetric dirichlet with fixed(-1.00) variance parameter

   2 --  Parameter  = Alpha
         Type       = Shape of scaled gamma distribution of site rates
         Prior      = Uniform(0.00,200.00)

   3 --  Parameter  = Ratemultiplier
         Type       = Partition-specific rate multiplier
         Prior      = Fixed(1.0)

   4 --  Parameter  = Tau
         Type       = Topology
         Prior      = All topologies equally probable a priori
         Subparam.  = V

   5 --  Parameter  = V
         Type       = Branch lengths
         Prior      = Clock:Uniform
                      Tree age has an Exponential(1.000) distribution
                      Node ages are not constrained

   6 --  Parameter  = Clockrate
         Type       = Base rate of clock
         Prior      = Fixed(1.000000)
                      The clock rate is constant (strict clock)


Number of taxa = 38
Number of characters = 116

The MCMC sampler will use the following moves:
   With prob.  Chain will use move
      2.38 %   Multiplier(Alpha)
     11.90 %   ExtSprClock(Tau,V)
     23.81 %   NNIClock(Tau,V)
     11.90 %   ParsSPRClock(Tau,V)
     47.62 %   NodesliderClock(V)
      2.38 %   TreeStretch(V)

Division 1 has 71 unique site patterns
Initializing conditional likelihoods
Using standard non-SSE likelihood calculator for division 1 (single-precision)
```

```
   Initial log likelihoods and log prior probs:
      Chain 1 -- -1417.300608 -- -87.230988
      Chain 2 -- -1434.196678 -- -87.230988
      Chain 3 -- -1393.517948 -- -87.230988
      Chain 4 -- -1476.315409 -- -87.230988


   Chain results (2000000 generations requested):

       0 -- [-1417.301] (-1434.197) (-1393.518) (-1476.315)

FILE TRUNCATED

   2000000 -- [-887.857] (-889.472) (-887.144) (-885.737) -- 0:00:00

   Continue with analysis? (yes/no): n

   Analysis completed in 15 mins 30 seconds
   Analysis used 866.52 seconds of CPU time
   Log likelihood of best state for "cold" chain was -855.15

   Acceptance rates for the moves in the "cold" chain:
      With prob.   (last 100)   chain accepted proposals by move
         46.0 %     ( 27 %)     Multiplier(Alpha)
         19.5 %     ( 23 %)     ExtSprClock(Tau,V)
         45.6 %     ( 50 %)     NNIClock(Tau,V)
         16.2 %     ( 21 %)     ParsSPRClock(Tau,V)
         71.0 %     ( 73 %)     NodesliderClock(V)
         69.6 %     ( 27 %)     TreeStretch(V)

   Chain swap information:

                1       2       3       4
        ----------------------------------
      1 |            0.57    0.29    0.13
      2 |  333501            0.62    0.34
      3 |  334725  332669            0.64
      4 |  333645  333423  332037

   Upper diagonal: Proportion of successful state exchanges between chains
   Lower diagonal: Number of attempted state exchanges between chains

   Chain information:

     ID -- Heat
    -----------
      1 -- 1.00  (cold chain)
      2 -- 0.91
      3 -- 0.83
      4 -- 0.77

   Heat = 1 / (1 + T * (ID - 1))
      (where T = 0.10 is the temperature and ID is the chain number)


MrBayes > sumt relburnin=yes

   Using relative burnin (a fraction of samples discarded).
   Summarizing trees in file "DY-27Dec-strict-H-KottOut.t"
   Using relative burnin ('relburnin=yes'), discarding the first 25 % of sampled trees
   Writing statistics to files DY-27Dec-strict-H-KottOut.<parts|tstat|vstat|trprobs|con>
```

Examining file ...
Found one tree block in file "DY-27Dec-strict-H-KottOut.t" with 4001 trees in last block

Tree reading status:

```
0      10      20      30      40      50      60      70      80      90     100
v-------v-------v-------v-------v-------v-------v-------v-------v-------v-------v
*********************************************************************************
```

Read 4001 trees from last tree block (sampling 3001 of them)

General explanation:

In an unrooted tree, a taxon bipartition (split) is specified by removing a branch, thereby dividing the species into those to the left and those to the right of the branch. Here, taxa to one side of the removed branch are denoted '.' and those to the other side are denoted '*'. Specifically, the '.' symbol is used for the taxa on the same side as the outgroup.

In a rooted or clock tree, the tree is rooted using the model and not by reference to an outgroup. Each bipartition therefore corresponds to a clade, that is, a group that includes all the descendants of a particular branch in the tree. Taxa that are included in each clade are denoted using '*', and taxa that are not included are denoted using the '.' symbol.

The output first includes a key to all the bipartitions with frequency larger or equual to (Minpartfreq) in at least one run. Minpartfreq is a paramiter to sumt command and currently it is set to 0.10. This is followed by a table with statistics for the informative bipartitions (those including at least two taxa), sorted from highest to lowest probability. For each bipartition, the table gives the number of times the partition or split was observed in all runs (#obs) and the posterior probability of the bipartition (Probab.), which is the same as the split frequency. If several runs are summarized, this is followed by the minimum split frequency (Min(s)), the maximum frequency (Max(s)), and the standard deviation of frequencies (Stddev(s)) across runs. The latter value should approach 0 for all bipartitions as MCMC runs converge.

This is followed by a table summarizing branch lengths, node heights (if a clock model was used) and relaxed clock parameters (if a relaxed clock model was used). The mean, variance, and 95 % credible interval are given for each of these parameters. If several runs are summarized, the potential scale reduction factor (PSRF) is also given; it should approach 1 as runs converge. Node heights will take calibration points into account, if such points were used in the analysis.

Note that Stddev may be unreliable if the partition is not present in all runs (the last column indicates the number of runs that sampled the partition if more than one run is summarized). The PSRF is not calculated at all if the partition is not present in all runs.The PSRF is also sensitive to small sample sizes and it should only be considered a rough guide to convergence since some of the assumptions allowing one to interpret it as a true potential scale reduction factor are violated in MrBayes.

List of taxa in bipartitions:

```
   1 -- gwi
   2 -- dgr
   3 -- scsh
   4 -- xsl
   5 -- bea
   6 -- crx
```

```
   7 -- chp
   8 -- txc
   9 -- haa
  10 -- ing
  11 -- kuu
  12 -- hoi
  13 -- koy
  14 -- taa
  15 -- aht
  16 -- tfn
  17 -- kkz
  18 -- tcb
  19 -- tau
  20 -- ttmN
  21 -- tceS
  22 -- eya
  23 -- tli
  24 -- gce
  25 -- tol
  26 -- cco
  27 -- hup
  28 -- mtl
  29 -- wlk
  30 -- kto
  31 -- apc
  32 -- apw
  33 -- apj
  34 -- nav
  35 -- apk
  36 -- apl
  37 -- srs
  38 -- ket
```

Key to taxon bipartitions (saved to file "DY-27Dec-strict-H-KottOut.parts"):

```
 ID -- Partition
---------------------------------------------
  0 -- **************************************
  1 -- *.....................................
  2 -- .*....................................
  3 -- ..*...................................
  4 -- ...*..................................
  5 -- ....*.................................
  6 -- .....*................................
  7 -- ......*...............................
  8 -- .......*..............................
  9 -- ........*.............................
 10 -- .........*............................
 11 -- ..........*...........................
 12 -- ...........*..........................
 13 -- ............*.........................
 14 -- .............*........................
 15 -- ..............*.......................
 16 -- ...............*......................
 17 -- ................*.....................
 18 -- .................*....................
 19 -- ..................*...................
 20 -- ...................*..................
 21 -- ....................*.................
 22 -- .....................*................
 23 -- ......................*...............
```

```
24 -- .......................*..............
25 -- ........................*.............
26 -- .........................*............
27 -- ..........................*...........
28 -- ...........................*..........
29 -- ............................*.........
30 -- .............................*........
31 -- ..............................*.......
32 -- ...............................*......
33 -- ................................*.....
34 -- .................................*....
35 -- ..................................*...
36 -- ...................................*..
37 -- ....................................*.
38 -- .....................................*
39 -- ..................................**..
40 -- ..............................*******.
41 -- .....................**...............
42 -- .............*...**...................
43 -- ............................**........
44 -- ..........................****.......*
45 -- ..............................****....
46 -- ..............................******..
47 -- .........*.**.........................
48 -- ..............................***.....
49 -- ***.**................................
50 -- ...*..*.*....*..*****.................
51 -- ..........................****........
52 -- ..............................**......
53 -- ...*............*.....................
54 -- ....**................................
55 -- .......................***............
56 -- *******.*....*..*****.................
57 -- ...........**.........................
58 -- ...*..*.*.......*..**.................
59 -- .............*...*....................
60 -- ......*............**.................
61 -- .......................**.............
62 -- ..........................*.**........
63 -- ...................**.................
64 -- ........................**............
65 -- .........****.........................
66 -- **....................................
67 -- ...........................***........
68 -- .**...................................
69 -- *********************.................
70 -- .................**...................
71 -- *********************..***....*******.
72 -- ..............**......................
73 -- ..*.**................................
74 -- .........*.*..........................
75 -- **************************....*******.
76 -- ..*..*................................
77 -- .......................***....*******.
78 -- *********************.........*******.
79 -- .........*.***...**...................
80 -- .....................**...****.......*
81 -- ...*....*.......*.....................
82 -- *...**................................
83 -- *******.*.......*..**.................
84 -- ......*.............*.................
```

```
 85 -- *.*.**................................
 86 -- ..............................*.*.....
 87 -- ......*............*..................
 88 -- ...............................**.....
 89 -- ...*..*.**.***..*****.................
 90 -- .......*.......*......................
 91 -- ..............*...............*******.
 92 -- *******.*....*..*****.........*******.
 93 -- .......................*******.......*
 94 -- ***..*................................
 95 -- ..................................***.
 96 -- ...*..*.........*..**.................
 97 -- .............*....*...................
 98 -- ...*...............*..................
 99 -- ......*.*..........**.................
100 -- ***...................................
101 -- .......*..*...........................
102 -- .......*......**......................
103 -- .......*.****.**......................
104 -- ..............................**.*....
105 -- .......*......*.......................
106 -- *********************..***************
107 -- ..........................**..........
108 -- .........*..*.........................
109 -- ........*....*...**...................
110 -- ***********************.......*******.
111 -- *******.**.***..*****.................
---------------------------------------------

Summary statistics for informative taxon bipartitions (clades)
   (saved to file "DY-27Dec-strict-H-KottOut.tstat"):

 ID   #obs    Probab.
----------------------
 39  2999    0.999334
 40  2808    0.935688
 41  2736    0.911696
 42  2734    0.911030
 43  2579    0.859380
 44  2469    0.822726
 45  2427    0.808730
 46  2232    0.743752
 47  2176    0.725092
 48  2028    0.675775
 49  1942    0.647118
 50  1923    0.640786
 51  1923    0.640786
 52  1839    0.612796
 53  1784    0.594469
 54  1771    0.590137
 55  1715    0.571476
 56  1676    0.558481
 57  1664    0.554482
 58  1585    0.528157
 59  1584    0.527824
 60  1454    0.484505
 61  1406    0.468510
 62  1331    0.443519
 63  1258    0.419194
 64  1234    0.411196
 65  1221    0.406864
```

66	1131	0.376874
67	1076	0.358547
68	1007	0.335555
69	969	0.322892
70	959	0.319560
71	927	0.308897
72	822	0.273909
73	817	0.272243
74	773	0.257581
75	714	0.237921
76	702	0.233922
77	694	0.231256
78	688	0.229257
79	670	0.223259
80	631	0.210263
81	605	0.201599
82	592	0.197268
83	586	0.195268
84	583	0.194269
85	551	0.183605
86	536	0.178607
87	522	0.173942
88	514	0.171276
89	507	0.168944
90	505	0.168277
91	495	0.164945
92	491	0.163612
93	475	0.158281
94	467	0.155615
95	465	0.154948
96	444	0.147951
97	428	0.142619
98	405	0.134955
99	402	0.133955
100	391	0.130290
101	369	0.122959
102	369	0.122959
103	343	0.114295
104	340	0.113296
105	339	0.112962
106	329	0.109630
107	328	0.109297
108	321	0.106964
109	312	0.103965
110	310	0.103299
111	301	0.100300

Summary statistics for branch and node parameters
(saved to file "DY-27Dec-strict-H-KottOut.vstat"):

Parameter	Mean	Variance	95% HPD Interval Lower	Upper	Median
length[1]	0.054449	0.000423	0.018219	0.095083	0.052436
length[2]	0.055576	0.000605	0.014802	0.102980	0.051390
length[3]	0.048364	0.000402	0.012873	0.086119	0.046138
length[4]	0.037867	0.000271	0.010269	0.071812	0.036148
length[5]	0.046347	0.000705	0.008326	0.103953	0.039523
length[6]	0.036925	0.000287	0.007704	0.070282	0.034539

length[7]	0.049322	0.000297	0.019337	0.084931	0.047776
length[8]	0.111573	0.000954	0.050016	0.169368	0.113302
length[9]	0.053438	0.000251	0.022560	0.084042	0.053188
length[10]	0.056867	0.000568	0.017443	0.104730	0.053392
length[11]	0.087011	0.001061	0.021213	0.146107	0.086051
length[12]	0.041888	0.000311	0.012407	0.076381	0.039884
length[13]	0.042657	0.000321	0.011516	0.076691	0.040719
length[14]	0.030081	0.000243	0.005024	0.060563	0.027108
length[15]	0.109421	0.000973	0.047858	0.170871	0.109624
length[16]	0.104962	0.000964	0.046271	0.165348	0.104496
length[17]	0.040388	0.000296	0.010935	0.074685	0.038735
length[18]	0.026681	0.000191	0.004379	0.053134	0.024092
length[19]	0.032489	0.000249	0.004412	0.063337	0.029896
length[20]	0.038338	0.000229	0.011747	0.068454	0.037193
length[21]	0.041123	0.000258	0.010135	0.070766	0.039669
length[22]	0.102785	0.000988	0.045962	0.167986	0.100090
length[23]	0.104147	0.001110	0.045323	0.176707	0.100463
length[24]	0.097111	0.001100	0.037343	0.164630	0.094226
length[25]	0.084026	0.000924	0.031620	0.150746	0.079829
length[26]	0.093943	0.001101	0.034862	0.160939	0.090343
length[27]	0.093961	0.000871	0.043750	0.157698	0.092037
length[28]	0.094750	0.000840	0.035292	0.147798	0.094445
length[29]	0.049341	0.000448	0.014590	0.091968	0.046311
length[30]	0.050470	0.000532	0.010872	0.095009	0.046651
length[31]	0.010093	0.000084	0.000005	0.028549	0.007484
length[32]	0.010243	0.000088	0.000005	0.028808	0.007595
length[33]	0.017168	0.000126	0.000670	0.038675	0.015014
length[34]	0.033660	0.000208	0.007665	0.062437	0.031529
length[35]	0.006208	0.000044	0.000002	0.019561	0.004109
length[36]	0.006206	0.000044	0.000002	0.019561	0.004109
length[37]	0.073655	0.000668	0.024360	0.123816	0.071672
length[38]	0.130551	0.001076	0.070848	0.201383	0.127650
length[39]	0.042672	0.000346	0.007468	0.078398	0.041266
length[40]	0.051389	0.000596	0.007229	0.098172	0.049300
length[41]	0.057985	0.000691	0.009456	0.109463	0.057551
length[42]	0.031162	0.000286	0.001631	0.061658	0.028856
length[43]	0.040067	0.000524	0.002073	0.083808	0.036867
length[44]	0.031167	0.000267	0.003498	0.063077	0.029304
length[45]	0.022066	0.000199	0.000063	0.049142	0.019226
length[46]	0.030234	0.000411	0.000101	0.068430	0.026662
length[47]	0.036706	0.000546	0.000067	0.080759	0.032316
length[48]	0.017726	0.000162	0.000030	0.042078	0.015193
length[49]	0.024810	0.000358	0.000001	0.061527	0.020683
length[50]	0.024220	0.000162	0.001545	0.048528	0.022528
length[51]	0.032316	0.000621	0.000019	0.080861	0.026772
length[52]	0.014878	0.000130	0.000005	0.037135	0.012318
length[53]	0.022679	0.000185	0.000143	0.048112	0.021055
length[54]	0.030317	0.000356	0.000055	0.064430	0.027206
length[55]	0.037110	0.000523	0.000168	0.078891	0.034026
length[56]	0.032923	0.000309	0.002772	0.065433	0.030538
length[57]	0.022488	0.000261	0.000102	0.053702	0.019761
length[58]	0.020654	0.000179	0.000021	0.044863	0.018649
length[59]	0.013267	0.000119	0.000025	0.035063	0.010518
length[60]	0.013513	0.000087	0.000090	0.031203	0.011711
length[61]	0.041834	0.000746	0.000012	0.094633	0.037036
length[62]	0.027900	0.000350	0.000157	0.064470	0.023927
length[63]	0.015266	0.000145	0.000060	0.038694	0.012761
length[64]	0.040994	0.000724	0.000035	0.089510	0.036625
length[65]	0.037971	0.000539	0.000100	0.081479	0.034941
length[66]	0.027138	0.000279	0.000040	0.057357	0.024815
length[67]	0.029657	0.000429	0.000054	0.069379	0.026347

length[68]	0.026477	0.000263	0.000840	0.057212	0.023941
length[69]	0.016362	0.000127	0.000444	0.038932	0.014060
length[70]	0.015507	0.000209	0.000031	0.044570	0.010729
length[71]	0.015888	0.000122	0.000135	0.037971	0.013718
length[72]	0.030459	0.000478	0.000068	0.069929	0.026451
length[73]	0.024863	0.000314	0.000022	0.057158	0.022064
length[74]	0.016257	0.000240	0.000076	0.042638	0.012599
length[75]	0.010750	0.000080	0.000067	0.029945	0.008432
length[76]	0.021499	0.000226	0.000123	0.048659	0.018963
length[77]	0.021349	0.000209	0.000239	0.049232	0.018843
length[78]	0.015384	0.000143	0.000184	0.038514	0.012595
length[79]	0.031338	0.000485	0.000571	0.075600	0.025821
length[80]	0.011779	0.000083	0.000012	0.028151	0.009912
length[81]	0.011318	0.000103	0.000010	0.029903	0.008608
length[82]	0.020761	0.000262	0.000019	0.052820	0.017270
length[83]	0.034349	0.000351	0.003689	0.072965	0.032539
length[84]	0.008866	0.000078	0.000014	0.025897	0.006174
length[85]	0.024760	0.000256	0.000165	0.054461	0.022623
length[86]	0.009192	0.000078	0.000006	0.027520	0.006575
length[87]	0.009517	0.000083	0.000032	0.028384	0.006834
length[88]	0.008865	0.000068	0.000023	0.025952	0.006487
length[89]	0.026860	0.000237	0.002719	0.054176	0.023358
length[90]	0.025015	0.000455	0.000002	0.068353	0.018659
length[91]	0.031328	0.000371	0.000143	0.063948	0.028606
length[92]	0.019639	0.000135	0.000590	0.039571	0.017539
length[93]	0.013364	0.000098	0.000188	0.032557	0.011265
length[94]	0.018921	0.000211	0.000075	0.045661	0.015176
length[95]	0.017093	0.000177	0.000042	0.043613	0.014086
length[96]	0.008680	0.000065	0.000012	0.023806	0.006735
length[97]	0.008920	0.000096	0.000038	0.029424	0.005854
length[98]	0.020598	0.000172	0.001586	0.045050	0.018578
length[99]	0.007228	0.000042	0.000010	0.020461	0.005926
length[100]	0.014138	0.000166	0.000010	0.040139	0.010138
length[101]	0.025287	0.000449	0.000061	0.069402	0.019860
length[102]	0.017802	0.000227	0.000027	0.045997	0.013972
length[103]	0.018385	0.000237	0.000027	0.046422	0.014892
length[104]	0.007535	0.000064	0.000005	0.022641	0.005268
length[105]	0.021322	0.000291	0.000094	0.052097	0.016363
length[106]	0.005706	0.000032	0.000001	0.017623	0.004038
length[107]	0.018733	0.000218	0.000083	0.047045	0.015195
length[108]	0.011491	0.000128	0.000015	0.034212	0.008149
length[109]	0.012310	0.000094	0.000025	0.032929	0.009964
length[110]	0.009118	0.000054	0.000122	0.024559	0.007388
length[111]	0.018784	0.000199	0.000015	0.043957	0.016432
height[0]	0.172294	0.000916	0.113402	0.233284	0.174672
height[1]	0.000000	0.000000	0.000000	0.000000	0.000000
height[2]	0.000000	0.000000	0.000000	0.000000	0.000000
height[3]	0.000000	0.000000	0.000000	0.000000	0.000000
height[4]	0.000000	0.000000	0.000000	0.000000	0.000000
height[5]	0.000000	0.000000	0.000000	0.000000	0.000000
height[6]	0.000000	0.000000	0.000000	0.000000	0.000000
height[7]	0.000000	0.000000	0.000000	0.000000	0.000000
height[8]	0.000000	0.000000	0.000000	0.000000	0.000000
height[9]	0.000000	0.000000	0.000000	0.000000	0.000000
height[10]	0.000000	0.000000	0.000000	0.000000	0.000000
height[11]	0.000000	0.000000	0.000000	0.000000	0.000000
height[12]	0.000000	0.000000	0.000000	0.000000	0.000000
height[13]	0.000000	0.000000	0.000000	0.000000	0.000000
height[14]	0.000000	0.000000	0.000000	0.000000	0.000000
height[15]	0.000000	0.000000	0.000000	0.000000	0.000000
height[16]	0.000000	0.000000	0.000000	0.000000	0.000000

height[17]	0.000000	0.000000	0.000000	0.000000	0.000000
height[18]	0.000000	0.000000	0.000000	0.000000	0.000000
height[19]	0.000000	0.000000	0.000000	0.000000	0.000000
height[20]	0.000000	0.000000	0.000000	0.000000	0.000000
height[21]	0.000000	0.000000	0.000000	0.000000	0.000000
height[22]	0.000000	0.000000	0.000000	0.000000	0.000000
height[23]	0.000000	0.000000	0.000000	0.000000	0.000000
height[24]	0.000000	0.000000	0.000000	0.000000	0.000000
height[25]	0.000000	0.000000	0.000000	0.000000	0.000000
height[26]	0.000000	0.000000	0.000000	0.000000	0.000000
height[27]	0.000000	0.000000	0.000000	0.000000	0.000000
height[28]	0.000000	0.000000	0.000000	0.000000	0.000000
height[29]	0.000000	0.000000	0.000000	0.000000	0.000000
height[30]	0.000000	0.000000	0.000000	0.000000	0.000000
height[31]	0.000000	0.000000	0.000000	0.000000	0.000000
height[32]	0.000000	0.000000	0.000000	0.000000	0.000000
height[33]	0.000000	0.000000	0.000000	0.000000	0.000000
height[34]	0.000000	0.000000	0.000000	0.000000	0.000000
height[35]	0.000000	0.000000	0.000000	0.000000	0.000000
height[36]	0.000000	0.000000	0.000000	0.000000	0.000000
height[37]	0.000000	0.000000	0.000000	0.000000	0.000000
height[38]	0.000000	0.000000	0.000000	0.000000	0.000000
height[39]	0.006176	0.000042	0.000002	0.019496	0.004107
height[40]	0.075504	0.000475	0.033638	0.119355	0.073986
height[41]	0.099606	0.000829	0.046304	0.158104	0.098127
height[42]	0.036252	0.000213	0.011514	0.067249	0.034137
height[43]	0.045474	0.000318	0.016017	0.083808	0.043495
height[44]	0.129177	0.000808	0.074924	0.187367	0.128079
height[45]	0.032771	0.000154	0.010833	0.057402	0.031153
height[46]	0.049798	0.000245	0.021172	0.083455	0.048169
height[47]	0.055858	0.000335	0.022757	0.094216	0.054965
height[48]	0.017229	0.000087	0.001658	0.034822	0.015796
height[49]	0.077481	0.000381	0.041351	0.118199	0.077334
height[50]	0.073777	0.000239	0.046019	0.107582	0.073646
height[51]	0.104035	0.000631	0.053738	0.154049	0.103298
height[52]	0.006830	0.000040	0.000005	0.019640	0.004936
height[53]	0.033004	0.000186	0.008262	0.059734	0.031309
height[54]	0.032521	0.000195	0.008177	0.059552	0.030822
height[55]	0.106386	0.000772	0.056017	0.164955	0.105898
height[56]	0.100228	0.000357	0.060474	0.135097	0.100577
height[57]	0.037022	0.000237	0.009514	0.066090	0.035231
height[58]	0.060966	0.000222	0.025971	0.087120	0.061002
height[59]	0.024394	0.000139	0.004379	0.048558	0.022386
height[60]	0.045366	0.000178	0.020036	0.070539	0.044119
height[61]	0.081918	0.000744	0.034047	0.143477	0.079375
height[62]	0.080808	0.000475	0.034338	0.119651	0.079734
height[63]	0.033673	0.000176	0.010832	0.060398	0.032046
height[64]	0.077174	0.000661	0.029068	0.127486	0.075138
height[65]	0.084764	0.000602	0.040011	0.138355	0.083398
height[66]	0.046331	0.000303	0.017622	0.083144	0.044396
height[67]	0.081315	0.000550	0.040575	0.131587	0.079822
height[68]	0.045314	0.000301	0.012314	0.077890	0.043398
height[69]	0.134530	0.000775	0.075396	0.188005	0.136123
height[70]	0.025066	0.000149	0.004412	0.048929	0.023026
height[71]	0.155112	0.000657	0.102648	0.202915	0.157768
height[72]	0.091931	0.000727	0.041464	0.142478	0.091708
height[73]	0.052192	0.000286	0.018739	0.083597	0.050842
height[74]	0.042274	0.000256	0.015405	0.075982	0.040700
height[75]	0.164974	0.000812	0.099025	0.210920	0.167270
height[76]	0.036072	0.000240	0.009060	0.067098	0.034397
height[77]	0.129710	0.000727	0.065657	0.173948	0.131747

height[78]	0.145461	0.000657	0.089954	0.193435	0.147078
height[79]	0.074778	0.000476	0.032087	0.117219	0.074423
height[80]	0.156308	0.000765	0.103381	0.208767	0.157763
height[81]	0.049558	0.000181	0.023883	0.075394	0.049311
height[82]	0.056849	0.000296	0.024353	0.090738	0.055694
height[83]	0.088830	0.000331	0.053595	0.125468	0.089348
height[84]	0.040518	0.000202	0.013979	0.066708	0.039533
height[85]	0.062392	0.000293	0.028514	0.091915	0.062108
height[86]	0.009967	0.000049	0.000428	0.024448	0.008626
height[87]	0.040952	0.000163	0.018814	0.068462	0.040491
height[88]	0.010039	0.000052	0.000719	0.023641	0.008723
height[89]	0.090147	0.000510	0.042683	0.133620	0.090709
height[90]	0.096927	0.000661	0.043267	0.142313	0.097523
height[91]	0.106244	0.000605	0.069138	0.161721	0.105791
height[92]	0.127540	0.000461	0.091617	0.172530	0.126691
height[93]	0.146658	0.000828	0.086873	0.203633	0.148551
height[94]	0.062662	0.000356	0.025930	0.094709	0.061964
height[95]	0.051165	0.000294	0.019690	0.085856	0.049554
height[96]	0.056599	0.000176	0.032749	0.087259	0.055717
height[97]	0.028235	0.000152	0.005905	0.052280	0.026791
height[98]	0.031112	0.000168	0.008715	0.057093	0.030661
height[99]	0.055622	0.000205	0.027309	0.085748	0.055357
height[100]	0.061707	0.000347	0.023095	0.094037	0.060873
height[101]	0.089084	0.000744	0.039672	0.144949	0.087597
height[102]	0.112629	0.000585	0.064082	0.159265	0.114852
height[103]	0.129570	0.000560	0.087017	0.177854	0.130853
height[104]	0.026445	0.000103	0.009616	0.049237	0.024905
height[105]	0.101570	0.000637	0.059218	0.154763	0.100521
height[106]	0.167112	0.000933	0.106008	0.230799	0.170105
height[107]	0.087263	0.000559	0.038374	0.132959	0.087746
height[108]	0.045025	0.000247	0.021776	0.081468	0.042960
height[109]	0.053791	0.000186	0.026654	0.077140	0.053404
height[110]	0.156358	0.000872	0.089920	0.215197	0.158977
height[111]	0.113166	0.000721	0.049077	0.163481	0.114773

```
----------------------------------------------------------------------
```

Clade credibility values:

```
/--------------------------------------------------------------------- txc (8)
|
|--------------------------------------------------------------------- kuu (11)
|
|--------------------------------------------------------------------- aht (15)
|
|--------------------------------------------------------------------- tfn (16)
|
|                                                         /----------- apc (31)
|                                             /-----61----+
|                                             |           \----------- apw (32)
|                                  /----68----+
|                                  |          \----------------------- apj (33)
|                      /-----81----+
|                      |           \---------------------------------- nav (34)
|           /----74----+
|           |          |                                  /----------- apk (35)
|           |          \----------------100---------------+
|-----94----+                                             \----------- apl (36)
|           |
```

```
|           \--------------------------------------------------------- srs (37)
|
|                                                         /----------- eya (22)
|---------------------------91----------------------------+
|                                                         \----------- tli (23)
|
|                                             /----------------------- hup (27)
|                                             |
|                                             |----------------------- mtl (28)
|                                  /----64----+
|                                  |          |           /----------- wlk (29)
+                                  |          \-----86----+
|----------------82----------------+                      \----------- kto (30)
|                                  |
|                                  \---------------------------------- ket (38)
|
|                                             /----------------------- ing (10)
|                                             |
|----------------------73---------------------+           /----------- hoi (12)
|                                             \-----55----+
|                                                         \----------- koy (13)
|
|                                                         /----------- gce (24)
|                                                         |
|---------------------------57----------------------------+----------- tol (25)
|                                                         |
|                                                         \----------- cco (26)
|
|                                             /----------------------- gwi (1)
|                                             |
|                                             |----------------------- dgr (2)
|                                             |
|                      /----------65----------+----------------------- scsh (3)
|                      |                      |
|                      |                      |           /----------- bea (5)
|                      |                      \-----59----+
|                      |                                  \----------- crx (6)
|                      |
|                      |                                  /----------- xsl (4)
|                      |                      /-----59----+
|                      |                      |           \----------- kkz (17)
\----------56----------+                      |
                       |                      |----------------------- chp (7)
                       |                      |
                       |           /----53----+----------------------- haa (9)
                       |           |          |
                       |           |          |----------------------- ttmN (20)
                       |           |          |
                       |           |          \----------------------- tceS (21)
                       \-----64----+
                                   |                      /----------- taa (14)
                                   |          /-----53----+
                                   |          |           \----------- tcb (18)
                                   \----91----+
                                              \----------------------- tau (19)
```

Phylogram (based on median node depths):

```
/--------------------------------------------------------------------- txc (8)
|
```

```
|--------------------------------------------------------------------- kuu (11)
|
|--------------------------------------------------------------------- aht (15)
|
|--------------------------------------------------------------------- tfn (16)
|
|                                                                  /-- apc (31)
|                                                              /---+
|                                                              |   \-- apw (32)
|                                                        /-----+
|                                                        |     \------ apj (33)
|                                                 /------+
|                                                 |      \------------ nav (34)
|                                       /---------+
|                                       |         |                /-- apk (35)
|                                       |         \----------------+
|---------------------------------------+                          \-- apl (36)
|                                       |
|                                       \----------------------------- srs (37)
|
|                             /--------------------------------------- eya (22)
|-----------------------------+
|                             \--------------------------------------- tli (23)
|
|                           /----------------------------------------- hup (27)
|                           |
|                           |----------------------------------------- mtl (28)
|                 /---------+
|                 |         |                       /----------------- wlk (29)
+                 |         \-----------------------+
|-----------------+                                 \----------------- kto (30)
|                 |
|                 \--------------------------------------------------- ket (38)
|
|                                              /---------------------- ing (10)
|                                              |
|----------------------------------------------+       /-------------- hoi (12)
|                                              \-------+
|                                                      \-------------- koy (13)
|
|                          /------------------------------------------ gce (24)
|                          |
|--------------------------+------------------------------------------ tol (25)
|                          |
|                          \------------------------------------------ cco (26)
|
|                                     /------------------------------- gwi (1)
|                                     |
|                                     |------------------------------- dgr (2)
|                                     |
|                            /--------+------------------------------- scsh (3)
|                            |        |
|                            |        |                  /------------ bea (5)
|                            |        \------------------+
|                            |                           \------------ crx (6)
|                            |
|                            |                           /------------ xsl (4)
|                            |               /-----------+
|                            |               |           \------------ kkz (17)
\----------------------------+               |
                             |               |------------------------ chp (7)
```

```
                              |               |
                              |          /----+------------------------ haa (9)
                              |          |    |
                              |          |    |------------------------ ttmN (20)
                              |          |    |
                              |          |    \------------------------ tceS (21)
                              \----------+
                                         |                 /--------- taa (14)
                                         |             /---+
                                         |             |   \--------- tcb (18)
                                         \-------------+
                                                       \------------- tau (19)

   ----------|-------------------|-------------------|------------------|
           0.15                0.10                0.05               0.00

   [Expected changes per site]

   Calculating tree probabilities...

   Credible sets of trees (3001 trees sampled):
      50 % credible set contains 1501 trees
      90 % credible set contains 2701 trees
      95 % credible set contains 2851 trees
      99 % credible set contains 2971 trees




                            MrBayes v3.2.1 x64

                      (Bayesian Analysis of Phylogeny)

              Distributed under the GNU General Public License


               Type "help" or "help <command>" for information
                     on the commands that are available.

                   Type "about" for authorship and general
                       information about the program.


MrBayes > exe /Users/msicoli/Kott-NaDene-Typlogical_noHAX.nex

   Executing file "/Users/msicoli/Kott-NaDene-Typlogical_noHAX.nex"
   UNIX line termination
   Longest line length = 123
   Parsing file
   Expecting NEXUS formatted file
   Reading data block
      Allocated taxon set
      Allocated matrix
      Defining new matrix with 38 taxa and 116 characters
      Data is Standard
      Missing data coded as ?
      Gaps coded as -
      Data matrix is not interleaved
      Taxon  1 -> gwi
      Taxon  2 -> dgr
```

```
      Taxon  3 -> scsh
      Taxon  4 -> xsl
      Taxon  5 -> bea
      Taxon  6 -> crx
      Taxon  7 -> chp
      Taxon  8 -> txc
      Taxon  9 -> haa
      Taxon 10 -> ing
      Taxon 11 -> kuu
      Taxon 12 -> hoi
      Taxon 13 -> koy
      Taxon 14 -> taa
      Taxon 15 -> aht
      Taxon 16 -> tfn
      Taxon 17 -> kkz
      Taxon 18 -> tcb
      Taxon 19 -> tau
      Taxon 20 -> ttmN
      Taxon 21 -> tceS
      Taxon 22 -> eya
      Taxon 23 -> tli
      Taxon 24 -> gce
      Taxon 25 -> tol
      Taxon 26 -> cco
      Taxon 27 -> hup
      Taxon 28 -> mtl
      Taxon 29 -> wlk
      Taxon 30 -> kto
      Taxon 31 -> apc
      Taxon 32 -> apw
      Taxon 33 -> apj
      Taxon 34 -> nav
      Taxon 35 -> apk
      Taxon 36 -> apl
      Taxon 37 -> srs
      Taxon 38 -> zko
      Successfully read matrix
      Setting default partition (does not divide up characters)
      Setting model defaults
      Seed (for generating default start values) = 1388166360
      Adding dummy characters (unobserved site patterns) for division 1
      WARNING: There are 37 characters incompatible with the specified
               coding bias. These characters will be excluded.
      Setting output file names to "/Users/msicoli/Kott-NaDene-Typlogical_noHAX.nex.run<i>.<p|t>"
   Exiting data block
   Reached end of file

MrBayes > lset nst=6 rates=gamma

   Setting Rates to Gamma
   Successfully set likelihood model parameters
   Adding dummy characters (unobserved site patterns) for division 1
   WARNING: There are 37 characters incompatible with the specified
            coding bias. These characters will be excluded.

MrBayes > prset brlenspr=clock:uniform

   Setting Brlenspr to Clock:Uniform
   Successfully set prior model parameters
   Adding dummy characters (unobserved site patterns) for division 1
   WARNING: There are 37 characters incompatible with the specified
```

```
            coding bias. These characters will be excluded.

MrBayes > mcmcp ngen=2000000 printfreq=10000 samplefreq=500 nruns=1 nchains=4 savebrlens=yes
filename=DY-27Dec-strict-H-Ketout

   Setting number of generations to 2000000
   Setting print frequency to 10000
   Setting sample frequency to 500
   Setting number of runs to 1
   Setting number of chains to 4
   Setting chain output file names to "DY-27Dec-strict-H-Ketout.<p/t>"
   Successfully set chain parameters

MrBayes > mcmc

   Running Markov chain
   MCMC stamp = 3571666900
   Seed = 498838383
   Swapseed = 1388166360
   Model settings:

      Data not partitioned --
         Datatype  = Standard
         Coding    = Variable
         # States  = Variable, up to 10
                     State frequencies are fixed to be equal
         Rates     = Gamma
                     Gamma shape parameter is uniformly dist-
                     ributed on the interval (0.00,200.00).
                     Gamma distribution is approximated using 4 categories.
                     Likelihood summarized over all rate categories in each generation.

   Active parameters:

      Parameters
      ------------------
      Statefreq        1
      Shape            2
      Ratemultiplier   3
      Topology         4
      Brlens           5
      Clockrate        6
      ------------------

      1 --  Parameter  = Alpha_symdir
            Type       = Symmetric diricihlet/beta distribution alpha_i parameter
            Prior      = Symmetric dirichlet with fixed(-1.00) variance parameter

      2 --  Parameter  = Alpha
            Type       = Shape of scaled gamma distribution of site rates
            Prior      = Uniform(0.00,200.00)

      3 --  Parameter  = Ratemultiplier
            Type       = Partition-specific rate multiplier
            Prior      = Fixed(1.0)

      4 --  Parameter  = Tau
            Type       = Topology
            Prior      = All topologies equally probable a priori
            Subparam.  = V
```

```
   5 --  Parameter  = V
         Type       = Branch lengths
         Prior      = Clock:Uniform
                      Tree age has an Exponential(1.000) distribution
                      Node ages are not constrained

   6 --  Parameter  = Clockrate
         Type       = Base rate of clock
         Prior      = Fixed(1.000000)
                      The clock rate is constant (strict clock)


Number of taxa = 38
Number of characters = 116

The MCMC sampler will use the following moves:
   With prob.  Chain will use move
      2.38 %   Multiplier(Alpha)
     11.90 %   ExtSprClock(Tau,V)
     23.81 %   NNIClock(Tau,V)
     11.90 %   ParsSPRClock(Tau,V)
     47.62 %   NodesliderClock(V)
      2.38 %   TreeStretch(V)

Division 1 has 73 unique site patterns
Initializing conditional likelihoods
Using standard non-SSE likelihood calculator for division 1 (single-precision)

Initial log likelihoods and log prior probs:
   Chain 1 -- -1436.616125 -- -87.230988
   Chain 2 -- -1499.059192 -- -87.230988
   Chain 3 -- -1473.869674 -- -87.230988
   Chain 4 -- -1572.409710 -- -87.230988


Chain results (2000000 generations requested):

    0 -- [-1436.616] (-1499.059) (-1473.870) (-1572.410)

FILE TRUNCATED

2000000 -- (-900.513) (-901.708) (-905.373) [-897.352] -- 0:00:00

Continue with analysis? (yes/no): n

Analysis completed in 15 mins 25 seconds
Analysis used 898.11 seconds of CPU time
Log likelihood of best state for "cold" chain was -867.29

Acceptance rates for the moves in the "cold" chain:
   With prob.   (last 100)   chain accepted proposals by move
      39.8 %     ( 30 %)     Multiplier(Alpha)
      19.9 %     ( 17 %)     ExtSprClock(Tau,V)
      45.9 %     ( 48 %)     NNIClock(Tau,V)
      16.2 %     ( 16 %)     ParsSPRClock(Tau,V)
      71.2 %     ( 74 %)     NodesliderClock(V)
      69.8 %     ( 28 %)     TreeStretch(V)

Chain swap information:

             1       2       3       4
```

```
     ----------------------------------
   1 |           0.51    0.25    0.11
   2 |  332145           0.61    0.33
   3 |  334117  333593           0.63
   4 |  333322  333079  333744

  Upper diagonal: Proportion of successful state exchanges between chains
  Lower diagonal: Number of attempted state exchanges between chains

  Chain information:

    ID -- Heat
   -----------
     1 -- 1.00  (cold chain)
     2 -- 0.91
     3 -- 0.83
     4 -- 0.77

  Heat = 1 / (1 + T * (ID - 1))
     (where T = 0.10 is the temperature and ID is the chain number)


MrBayes > sumt relburnin=yes

  Using relative burnin (a fraction of samples discarded).
  Summarizing trees in file "DY-27Dec-strict-H-Ketout.t"
  Using relative burnin ('relburnin=yes'), discarding the first 25 % of sampled trees
  Writing statistics to files DY-27Dec-strict-H-Ketout.<parts|tstat|vstat|trprobs|con>
  Examining file ...
  Found one tree block in file "DY-27Dec-strict-H-Ketout.t" with 4001 trees in last block

  Tree reading status:

  0      10      20      30      40      50      60      70      80      90     100
  v-------v-------v-------v-------v-------v-------v-------v-------v-------v-------v
  *********************************************************************************

  Read 4001 trees from last tree block (sampling 3001 of them)

  General explanation:

  In an unrooted tree, a taxon bipartition (split) is specified by removing a
  branch, thereby dividing the species into those to the left and those to the
  right of the branch. Here, taxa to one side of the removed branch are denoted
  '.' and those to the other side are denoted '*'. Specifically, the '.' symbol
  is used for the taxa on the same side as the outgroup.

  In a rooted or clock tree, the tree is rooted using the model and not by
  reference to an outgroup. Each bipartition therefore corresponds to a clade,
  that is, a group that includes all the descendants of a particular branch in
  the tree.  Taxa that are included in each clade are denoted using '*', and
  taxa that are not included are denoted using the '.' symbol.

  The output first includes a key to all the bipartitions with frequency larger
  or equual to (Minpartfreq) in at least one run. Minpartfreq is a paramiter to
  sumt command and currently it is set to 0.10.  This is followed by a table
  with statistics for the informative bipartitions (those including at least
  two taxa), sorted from highest to lowest probability. For each bipartition,
  the table gives the number of times the partition or split was observed in all
  runs (#obs) and the posterior probability of the bipartition (Probab.), which
  is the same as the split frequency. If several runs are summarized, this is
```

followed by the minimum split frequency (Min(s)), the maximum frequency (Max(s)), and the standard deviation of frequencies (Stddev(s)) across runs. The latter value should approach 0 for all bipartitions as MCMC runs converge.

This is followed by a table summarizing branch lengths, node heights (if a clock model was used) and relaxed clock parameters (if a relaxed clock model was used). The mean, variance, and 95 % credible interval are given for each of these parameters. If several runs are summarized, the potential scale reduction factor (PSRF) is also given; it should approach 1 as runs converge. Node heights will take calibration points into account, if such points were used in the analysis.

Note that Stddev may be unreliable if the partition is not present in all runs (the last column indicates the number of runs that sampled the partition if more than one run is summarized). The PSRF is not calculated at all if the partition is not present in all runs.The PSRF is also sensitive to small sample sizes and it should only be considered a rough guide to convergence since some of the assumptions allowing one to interpret it as a true potential scale reduction factor are violated in MrBayes.

List of taxa in bipartitions:

```
   1 -- gwi
   2 -- dgr
   3 -- scsh
   4 -- xsl
   5 -- bea
   6 -- crx
   7 -- chp
   8 -- txc
   9 -- haa
  10 -- ing
  11 -- kuu
  12 -- hoi
  13 -- koy
  14 -- taa
  15 -- aht
  16 -- tfn
  17 -- kkz
  18 -- tcb
  19 -- tau
  20 -- ttmN
  21 -- tceS
  22 -- eya
  23 -- tli
  24 -- gce
  25 -- tol
  26 -- cco
  27 -- hup
  28 -- mtl
  29 -- wlk
  30 -- kto
  31 -- apc
  32 -- apw
  33 -- apj
  34 -- nav
  35 -- apk
  36 -- apl
  37 -- srs
  38 -- zko
```

Key to taxon bipartitions (saved to file "DY-27Dec-strict-H-Ketout.parts"):

```
 ID -- Partition
---------------------------------------------
  0 -- **************************************
  1 -- *.....................................
  2 -- .*....................................
  3 -- ..*...................................
  4 -- ...*..................................
  5 -- ....*.................................
  6 -- .....*................................
  7 -- ......*...............................
  8 -- .......*..............................
  9 -- ........*.............................
 10 -- .........*............................
 11 -- ..........*...........................
 12 -- ...........*..........................
 13 -- ............*.........................
 14 -- .............*........................
 15 -- ..............*.......................
 16 -- ...............*......................
 17 -- ................*.....................
 18 -- .................*....................
 19 -- ..................*...................
 20 -- ...................*..................
 21 -- ....................*.................
 22 -- .....................*................
 23 -- ......................*...............
 24 -- .......................*..............
 25 -- ........................*.............
 26 -- .........................*............
 27 -- ..........................*...........
 28 -- ...........................*..........
 29 -- ............................*.........
 30 -- .............................*........
 31 -- ..............................*.......
 32 -- ...............................*......
 33 -- ................................*.....
 34 -- .................................*....
 35 -- ..................................*...
 36 -- ...................................*..
 37 -- ....................................*.
 38 -- .....................................*
 39 -- ..................................**..
 40 -- ..............................*******.
 41 -- .....................**...............
 42 -- .............*...**...................
 43 -- ............................**........
 44 -- ..............................****....
 45 -- ..........................****........
 46 -- .........*.**.........................
 47 -- ..............................******..
 48 -- ***.**................................
 49 -- ..............................***.....
 50 -- ...*..*.*....*..*****.................
 51 -- ...*............*.....................
 52 -- ..............................**......
 53 -- ....**................................
 54 -- ...........**.........................
 55 -- ...*..*.*.......*..**.................
 56 -- .......................***............
```

```
 57 -- ......*............**.................
 58 -- .............*...*....................
 59 -- *******.*....*..*****.................
 60 -- ...................**.................
 61 -- ........................**............
 62 -- .......................**.............
 63 -- ...........................***........
 64 -- .........****.........................
 65 -- ..........................*.**........
 66 -- ..........................****.......*
 67 -- **....................................
 68 -- .................**...................
 69 -- .**...................................
 70 -- *********************..***....*******.
 71 -- ..*.**................................
 72 -- ...*..*.**.***..*****.................
 73 -- ..............**....................
 74 -- *************************************.
 75 -- .........*.*..........................
 76 -- .........*.***...**...................
 77 -- ..*..*................................
 78 -- **************************....*******.
 79 -- ...*....*.......*.....................
 80 -- *********************.................
 81 -- *********************.........*******.
 82 -- *.*.**................................
 83 -- *...**................................
 84 -- ..............................*.*.....
 85 -- ......*............*..................
 86 -- ..............*...............*******.
 87 -- ......*.............*.................
 88 -- .......................***....*******.
 89 -- ...............................**.....
 90 -- ..................................***.
 91 -- .......*.......*......................
 92 -- ...*..*.........*..**.................
 93 -- ***..*................................
 94 -- *******.*....*..*****.........*******.
 95 -- ..........................**..........
 96 -- ***...................................
 97 -- .......................*******........
 98 -- *******.**.***..*****.................
 99 -- ..............................**.*....
100 -- .............*...*...................
101 -- .......*..*...........................
102 -- *******.*.......*..**.................
103 -- ......*.*..........**.................
104 -- *********************..**************.
105 -- ..........*....*......................
106 -- ...*...............*..................
107 -- .......*......*.......................
108 -- .....................**...****.......*
109 -- .........*..*.........................
110 -- .......*..*....*......................
111 -- .......*......**......................
---------------------------------------------
```

Summary statistics for informative taxon bipartitions (clades)
   (saved to file "DY-27Dec-strict-H-Ketout.tstat"):

 ID   #obs   Probab.

39	3000	0.999667
40	2770	0.923026
41	2616	0.871709
42	2579	0.859380
43	2536	0.845052
44	2493	0.830723
45	2469	0.822726
46	2273	0.757414
47	2208	0.735755
48	2091	0.696768
49	1990	0.663112
50	1873	0.624125
51	1848	0.615795
52	1826	0.608464
53	1802	0.600467
54	1723	0.574142
55	1642	0.547151
56	1611	0.536821
57	1536	0.511829
58	1529	0.509497
59	1492	0.497168
60	1310	0.436521
61	1275	0.424858
62	1247	0.415528
63	1172	0.390536
64	1146	0.381873
65	1144	0.381206
66	1129	0.376208
67	1110	0.369877
68	1052	0.350550
69	945	0.314895
70	920	0.306564
71	859	0.286238
72	843	0.280906
73	778	0.259247
74	758	0.252582
75	741	0.246918
76	725	0.241586
77	712	0.237254
78	701	0.233589
79	680	0.226591
80	674	0.224592
81	664	0.221260
82	580	0.193269
83	576	0.191936
84	554	0.184605
85	552	0.183939
86	543	0.180940
87	537	0.178940
88	533	0.177607
89	512	0.170610
90	488	0.162612
91	474	0.157947
92	471	0.156948
93	452	0.150616
94	420	0.139953
95	418	0.139287
96	416	0.138620
97	413	0.137621
98	403	0.134289

99	378	0.125958
100	373	0.124292
101	371	0.123625
102	366	0.121959
103	356	0.118627
104	351	0.116961
105	344	0.114628
106	339	0.112962
107	337	0.112296
108	316	0.105298
109	311	0.103632
110	308	0.102632
111	303	0.100966

Summary statistics for branch and node parameters
(saved to file "DY-27Dec-strict-H-Ketout.vstat"):

			95% HPD Interval		
Parameter	Mean	Variance	Lower	Upper	Median
length[1]	0.052545	0.000471	0.011730	0.093428	0.050256
length[2]	0.053626	0.000632	0.014504	0.105335	0.049081
length[3]	0.046066	0.000415	0.011731	0.085941	0.044023
length[4]	0.035983	0.000287	0.008153	0.070499	0.033569
length[5]	0.044289	0.000742	0.005749	0.101774	0.037527
length[6]	0.035011	0.000301	0.006534	0.069443	0.032513
length[7]	0.046579	0.000323	0.015862	0.082167	0.044973
length[8]	0.106981	0.001158	0.037343	0.167190	0.107333
length[9]	0.051025	0.000296	0.016946	0.082941	0.050555
length[10]	0.052613	0.000605	0.011542	0.100596	0.048617
length[11]	0.085711	0.001215	0.022021	0.152710	0.084126
length[12]	0.038011	0.000299	0.008160	0.071208	0.035729
length[13]	0.038860	0.000316	0.008114	0.072726	0.036443
length[14]	0.029709	0.000258	0.004829	0.062037	0.026441
length[15]	0.103577	0.001155	0.035865	0.166033	0.102898
length[16]	0.100245	0.001154	0.037135	0.165366	0.099380
length[17]	0.037992	0.000308	0.008153	0.072077	0.035703
length[18]	0.025773	0.000195	0.003009	0.052544	0.023337
length[19]	0.031665	0.000271	0.004877	0.064799	0.028605
length[20]	0.036646	0.000248	0.008868	0.067156	0.035516
length[21]	0.038464	0.000260	0.009657	0.068485	0.037315
length[22]	0.100333	0.001277	0.033498	0.170833	0.096747
length[23]	0.103392	0.001574	0.033635	0.187098	0.097154
length[24]	0.092903	0.001244	0.030557	0.162659	0.090141
length[25]	0.079551	0.001012	0.024023	0.144246	0.076243
length[26]	0.087651	0.001107	0.025270	0.150085	0.085602
length[27]	0.087018	0.001072	0.027935	0.153990	0.083961
length[28]	0.084351	0.001013	0.025498	0.147576	0.082180
length[29]	0.044622	0.000425	0.009227	0.083197	0.041859
length[30]	0.046102	0.000491	0.007559	0.088895	0.042465
length[31]	0.008993	0.000068	0.000007	0.025746	0.006587
length[32]	0.009297	0.000074	0.000007	0.026664	0.006799
length[33]	0.015643	0.000115	0.000154	0.036355	0.013337
length[34]	0.030651	0.000203	0.007526	0.058370	0.028582
length[35]	0.005603	0.000036	0.000001	0.017584	0.003782
length[36]	0.005600	0.000036	0.000001	0.017581	0.003782
length[37]	0.069449	0.000734	0.019469	0.118805	0.067191
length[38]	0.158643	0.001994	0.054699	0.234909	0.160000
length[39]	0.040365	0.000341	0.006959	0.075273	0.038765

length[40]	0.047753	0.000635	0.005567	0.097586	0.044548
length[41]	0.057113	0.000835	0.006556	0.112697	0.054881
length[42]	0.029107	0.000297	0.000382	0.060378	0.026958
length[43]	0.037934	0.000539	0.001025	0.081658	0.034029
length[44]	0.020890	0.000199	0.000087	0.048830	0.017930
length[45]	0.048450	0.000856	0.000101	0.102490	0.044797
length[46]	0.035039	0.000538	0.000010	0.081008	0.030077
length[47]	0.029151	0.000400	0.000122	0.068535	0.025530
length[48]	0.027100	0.000404	0.000083	0.066414	0.022999
length[49]	0.016751	0.000150	0.000010	0.040393	0.014357
length[50]	0.024557	0.000188	0.001331	0.051284	0.022423
length[51]	0.021586	0.000190	0.000001	0.047066	0.019168
length[52]	0.013709	0.000115	0.000020	0.034331	0.011414
length[53]	0.028674	0.000380	0.000041	0.065200	0.024605
length[54]	0.021312	0.000261	0.000007	0.051954	0.017408
length[55]	0.019254	0.000195	0.000033	0.044893	0.016565
length[56]	0.036245	0.000556	0.000085	0.079819	0.032101
length[57]	0.013407	0.000092	0.000071	0.032521	0.011174
length[58]	0.013132	0.000118	0.000023	0.034532	0.009872
length[59]	0.034345	0.000356	0.001157	0.068458	0.031979
length[60]	0.014242	0.000135	0.000010	0.037572	0.011360
length[61]	0.037941	0.000684	0.000000	0.087112	0.033009
length[62]	0.037205	0.000659	0.000002	0.087156	0.032786
length[63]	0.029123	0.000503	0.000009	0.072601	0.024051
length[64]	0.035636	0.000565	0.000172	0.081616	0.031328
length[65]	0.026188	0.000403	0.000021	0.064782	0.021879
length[66]	0.021513	0.000217	0.000031	0.049017	0.018573
length[67]	0.025137	0.000272	0.000098	0.055208	0.022450
length[68]	0.015626	0.000215	0.000000	0.044657	0.011387
length[69]	0.024578	0.000264	0.000150	0.056420	0.021325
length[70]	0.019466	0.000179	0.000027	0.046735	0.016864
length[71]	0.022772	0.000343	0.000032	0.057702	0.018628
length[72]	0.027075	0.000256	0.003510	0.056837	0.023768
length[73]	0.031277	0.000461	0.000032	0.070870	0.026828
length[74]	0.016037	0.000248	0.000003	0.046983	0.011055
length[75]	0.013923	0.000164	0.000035	0.036641	0.010066
length[76]	0.026362	0.000460	0.000012	0.069172	0.020725
length[77]	0.021290	0.000238	0.000013	0.050616	0.018045
length[78]	0.013461	0.000132	0.000035	0.036084	0.010575
length[79]	0.010412	0.000086	0.000007	0.030265	0.007803
length[80]	0.015800	0.000148	0.000070	0.038033	0.013217
length[81]	0.016367	0.000176	0.000173	0.039703	0.012740
length[82]	0.023551	0.000267	0.000175	0.054458	0.020202
length[83]	0.019116	0.000239	0.000081	0.049405	0.015598
length[84]	0.009020	0.000074	0.000001	0.025915	0.006577
length[85]	0.009002	0.000075	0.000003	0.026362	0.006414
length[86]	0.029110	0.000371	0.000494	0.068445	0.026299
length[87]	0.009116	0.000079	0.000049	0.026989	0.005927
length[88]	0.023412	0.000251	0.000283	0.054809	0.020612
length[89]	0.007789	0.000060	0.000021	0.023030	0.005276
length[90]	0.015092	0.000146	0.000003	0.038309	0.012185
length[91]	0.023236	0.000406	0.000002	0.062712	0.017451
length[92]	0.008634	0.000075	0.000000	0.025915	0.005878
length[93]	0.020249	0.000229	0.000009	0.047593	0.018242
length[94]	0.019433	0.000179	0.000040	0.043558	0.016450
length[95]	0.016932	0.000306	0.000007	0.052635	0.011106
length[96]	0.015610	0.000196	0.000037	0.041176	0.011961
length[97]	0.022687	0.000262	0.000148	0.051975	0.020971
length[98]	0.018785	0.000250	0.000009	0.050099	0.014486
length[99]	0.006943	0.000049	0.000004	0.020906	0.004536
length[100]	0.008817	0.000100	0.000012	0.027927	0.005339

length[101]	0.024428	0.000449	0.000030	0.068631	0.019036
length[102]	0.036297	0.000401	0.003337	0.072715	0.033933
length[103]	0.007381	0.000047	0.000007	0.019778	0.005420
length[104]	0.014441	0.000138	0.000051	0.037904	0.011421
length[105]	0.025124	0.000476	0.000088	0.069624	0.018548
length[106]	0.019591	0.000169	0.000150	0.041711	0.017566
length[107]	0.022613	0.000316	0.000063	0.057674	0.018731
length[108]	0.009497	0.000069	0.000011	0.026328	0.007257
length[109]	0.010402	0.000117	0.000011	0.027493	0.007713
length[110]	0.024203	0.000335	0.000080	0.058589	0.021363
length[111]	0.020345	0.000327	0.000053	0.051040	0.015072
height[0]	0.175506	0.001777	0.067404	0.244953	0.179710
height[1]	0.000000	0.000000	0.000000	0.000000	0.000000
height[2]	0.000000	0.000000	0.000000	0.000000	0.000000
height[3]	0.000000	0.000000	0.000000	0.000000	0.000000
height[4]	0.000000	0.000000	0.000000	0.000000	0.000000
height[5]	0.000000	0.000000	0.000000	0.000000	0.000000
height[6]	0.000000	0.000000	0.000000	0.000000	0.000000
height[7]	0.000000	0.000000	0.000000	0.000000	0.000000
height[8]	0.000000	0.000000	0.000000	0.000000	0.000000
height[9]	0.000000	0.000000	0.000000	0.000000	0.000000
height[10]	0.000000	0.000000	0.000000	0.000000	0.000000
height[11]	0.000000	0.000000	0.000000	0.000000	0.000000
height[12]	0.000000	0.000000	0.000000	0.000000	0.000000
height[13]	0.000000	0.000000	0.000000	0.000000	0.000000
height[14]	0.000000	0.000000	0.000000	0.000000	0.000000
height[15]	0.000000	0.000000	0.000000	0.000000	0.000000
height[16]	0.000000	0.000000	0.000000	0.000000	0.000000
height[17]	0.000000	0.000000	0.000000	0.000000	0.000000
height[18]	0.000000	0.000000	0.000000	0.000000	0.000000
height[19]	0.000000	0.000000	0.000000	0.000000	0.000000
height[20]	0.000000	0.000000	0.000000	0.000000	0.000000
height[21]	0.000000	0.000000	0.000000	0.000000	0.000000
height[22]	0.000000	0.000000	0.000000	0.000000	0.000000
height[23]	0.000000	0.000000	0.000000	0.000000	0.000000
height[24]	0.000000	0.000000	0.000000	0.000000	0.000000
height[25]	0.000000	0.000000	0.000000	0.000000	0.000000
height[26]	0.000000	0.000000	0.000000	0.000000	0.000000
height[27]	0.000000	0.000000	0.000000	0.000000	0.000000
height[28]	0.000000	0.000000	0.000000	0.000000	0.000000
height[29]	0.000000	0.000000	0.000000	0.000000	0.000000
height[30]	0.000000	0.000000	0.000000	0.000000	0.000000
height[31]	0.000000	0.000000	0.000000	0.000000	0.000000
height[32]	0.000000	0.000000	0.000000	0.000000	0.000000
height[33]	0.000000	0.000000	0.000000	0.000000	0.000000
height[34]	0.000000	0.000000	0.000000	0.000000	0.000000
height[35]	0.000000	0.000000	0.000000	0.000000	0.000000
height[36]	0.000000	0.000000	0.000000	0.000000	0.000000
height[37]	0.000000	0.000000	0.000000	0.000000	0.000000
height[38]	0.000000	0.000000	0.000000	0.000000	0.000000
height[39]	0.005598	0.000036	0.000001	0.017581	0.003782
height[40]	0.070546	0.000521	0.024824	0.113309	0.069506
height[41]	0.096035	0.001063	0.033078	0.157832	0.093395
height[42]	0.035063	0.000237	0.008716	0.064776	0.032826
height[43]	0.041080	0.000306	0.009227	0.074904	0.039126
height[44]	0.030106	0.000142	0.009682	0.053512	0.028861
height[45]	0.096388	0.000895	0.037071	0.156540	0.095968
height[46]	0.051984	0.000392	0.016107	0.090249	0.050045
height[47]	0.046539	0.000264	0.016012	0.077386	0.045258
height[48]	0.073894	0.000498	0.026705	0.113156	0.074036
height[49]	0.015475	0.000080	0.002167	0.033140	0.013926

height[50]	0.070000	0.000330	0.027764	0.103749	0.070663
height[51]	0.030955	0.000176	0.007243	0.056265	0.029637
height[52]	0.006166	0.000036	0.000007	0.017729	0.004427
height[53]	0.030665	0.000207	0.003762	0.056070	0.028766
height[54]	0.033764	0.000227	0.007979	0.063297	0.031969
height[55]	0.058786	0.000279	0.022270	0.089275	0.059107
height[56]	0.098171	0.000887	0.035942	0.154210	0.097524
height[57]	0.043263	0.000193	0.014805	0.068307	0.042165
height[58]	0.023407	0.000131	0.004829	0.045259	0.021514
height[59]	0.097420	0.000529	0.037773	0.139202	0.098567
height[60]	0.031387	0.000173	0.008406	0.056079	0.030108
height[61]	0.072485	0.000733	0.023187	0.125389	0.070613
height[62]	0.075607	0.000766	0.020701	0.127181	0.073541
height[63]	0.074016	0.000662	0.026871	0.128030	0.072219
height[64]	0.081000	0.000752	0.027597	0.136047	0.079122
height[65]	0.075536	0.000634	0.022272	0.119883	0.074744
height[66]	0.141550	0.001380	0.054439	0.205282	0.142910
height[67]	0.044331	0.000337	0.009929	0.079042	0.043348
height[68]	0.024403	0.000171	0.004083	0.050417	0.022120
height[69]	0.042589	0.000299	0.012988	0.076899	0.040856
height[70]	0.149513	0.000992	0.073298	0.206985	0.153690
height[71]	0.049354	0.000325	0.011570	0.080186	0.048637
height[72]	0.084253	0.000612	0.035427	0.127244	0.086878
height[73]	0.086946	0.000783	0.028589	0.138012	0.086898
height[74]	0.174625	0.001496	0.076570	0.236859	0.180371
height[75]	0.038617	0.000255	0.011002	0.067744	0.037385
height[76]	0.070196	0.000515	0.022996	0.109591	0.069875
height[77]	0.034571	0.000238	0.008066	0.065436	0.032796
height[78]	0.162191	0.001329	0.062813	0.216334	0.167387
height[79]	0.047557	0.000220	0.016148	0.073427	0.046565
height[80]	0.133679	0.000891	0.069927	0.188526	0.134690
height[81]	0.138111	0.001124	0.056916	0.190241	0.140454
height[82]	0.059711	0.000350	0.022364	0.098649	0.059525
height[83]	0.052639	0.000335	0.019845	0.087214	0.051678
height[84]	0.008796	0.000039	0.000154	0.020835	0.007537
height[85]	0.038442	0.000192	0.013523	0.063719	0.036791
height[86]	0.098362	0.000769	0.038239	0.147950	0.096122
height[87]	0.039674	0.000197	0.011878	0.065341	0.039156
height[88]	0.121085	0.000770	0.065843	0.180370	0.121971
height[89]	0.009257	0.000044	0.000295	0.021205	0.007762
height[90]	0.047547	0.000326	0.014852	0.081660	0.045284
height[91]	0.092107	0.000818	0.039431	0.146440	0.091431
height[92]	0.054643	0.000244	0.021972	0.081453	0.053921
height[93]	0.062018	0.000377	0.025389	0.097117	0.062810
height[94]	0.124813	0.000792	0.047915	0.172920	0.125033
height[95]	0.075601	0.000718	0.020908	0.120488	0.076288
height[96]	0.059064	0.000381	0.021210	0.093331	0.058259
height[97]	0.130981	0.001107	0.068152	0.192161	0.132690
height[98]	0.106012	0.000990	0.045389	0.161259	0.108178
height[99]	0.023756	0.000113	0.008362	0.045296	0.023040
height[100]	0.028709	0.000189	0.006840	0.054340	0.026400
height[101]	0.084943	0.000930	0.032067	0.140022	0.083032
height[102]	0.089364	0.000455	0.041786	0.126451	0.089983
height[103]	0.051298	0.000251	0.018781	0.080930	0.050855
height[104]	0.165373	0.001252	0.062379	0.218460	0.169097
height[105]	0.084173	0.000898	0.034226	0.148660	0.080961
height[106]	0.029997	0.000197	0.004478	0.052793	0.029039
height[107]	0.097259	0.000838	0.048826	0.158514	0.095729
height[108]	0.160286	0.001221	0.073906	0.218610	0.164361
height[109]	0.041097	0.000283	0.010404	0.071341	0.039817
height[110]	0.096661	0.000990	0.031982	0.147012	0.097060

```
height[111]    0.108685    0.000756    0.049595    0.154040    0.109443
----------------------------------------------------------------------
```

Clade credibility values:

```
/---------------------------------------------------------------------- txc (8)
|
|---------------------------------------------------------------------- kuu (11)
|
|---------------------------------------------------------------------- aht (15)
|
|---------------------------------------------------------------------- tfn (16)
|
|---------------------------------------------------------------------- zko (38)
|
|                                                           /----------- apc (31)
|                                               /-----61----+
|                                               |           \----------- apw (32)
|                                    /----66----+
|                                    |          \----------------------- apj (33)
|                        /-----83----+
|                        |           \---------------------------------- nav (34)
|           /----74-----+
|           |           |                                   /----------- apk (35)
|           |           \----------------100---------------+
|-----92----+                                               \----------- apl (36)
|           |
|           \---------------------------------------------------------- srs (37)
|
|                                                           /----------- eya (22)
|-----------------------------87--------------------------+
|                                                           \----------- tli (23)
|
|                                               /----------------------- hup (27)
|                                               |
|                                               |----------------------- mtl (28)
|----------------------82---------------------+
|                                               |           /----------- wlk (29)
|                                               \-----85----+
|                                                           \----------- kto (30)
|
+                                               /----------------------- ing (10)
|                                               |
|----------------------76---------------------+           /----------- hoi (12)
|                                               \-----57----+
|                                                           \----------- koy (13)
|
|                                               /----------------------- gwi (1)
|                                               |
|                                               |----------------------- dgr (2)
|                                               |
|----------------------70---------------------+----------------------- scsh (3)
|                                               |
|                                               |           /----------- bea (5)
|                                               \-----60----+
|                                                           \----------- crx (6)
|
|                                                           /----------- xsl (4)
```

```
|                                             /-----62----+
|                                             |           \----------- kkz (17)
|                                             |
|                                             |           /----------- chp (7)
|                                             |           |
|                                  /----55----+-----51----+----------- ttmN (20)
|                                  |          |           |
|                                  |          |           \----------- tceS (21)
|                                  |          |
|                                  |          \----------------------- haa (9)
|----------------62----------------+
|                                  |                      /----------- taa (14)
|                                  |          /-----51----+
|                                  |          |           \----------- tcb (18)
|                                  \----86----+
|                                             \----------------------- tau (19)
|
|                                                         /----------- gce (24)
|                                                         |
\----------------------------54---------------------------+----------- tol (25)
                                                          |
                                                          \----------- cco (26)
```

Phylogram (based on median node depths):

```
/--------------------------------------------------------------------- txc (8)
|
|--------------------------------------------------------------------- kuu (11)
|
|--------------------------------------------------------------------- aht (15)
|
|--------------------------------------------------------------------- tfn (16)
|
|--------------------------------------------------------------------- zko (38)
|
|                                                                  /-- apc (31)
|                                                               /--+
|                                                               |  \-- apw (32)
|                                                         /-----+
|                                                         |     \----- apj (33)
|                                                   /-----+
|                                                   |     \----------- nav (34)
|                                         /---------+
|                                         |         |               /- apk (35)
|                                         |         \---------------+
|-----------------------------------------+                        \- apl (36)
|                                         |
|                                         \--------------------------- srs (37)
|
|                                /------------------------------------ eya (22)
|--------------------------------+
|                                \------------------------------------ tli (23)
|
|                               /------------------------------------- hup (27)
|                               |
|                               |------------------------------------- mtl (28)
|-------------------------------+
|                               |                     /--------------- wlk (29)
|                               \---------------------+
|                                                     \--------------- kto (30)
|
```

```
|
+                                                  /------------------- ing (10)
|                                                  |
|--------------------------------------------------+      /------------ hoi (12)
|                                                  \------+
|                                                         \------------ koy (13)
|
|                                         /---------------------------- gwi (1)
|                                         |
|                                         |---------------------------- dgr (2)
|                                         |
|-----------------------------------------+---------------------------- scsh (3)
|                                         |
|                                         |                /----------- bea (5)
|                                         \----------------+
|                                                          \----------- crx (6)
|
|                                                          /----------- xsl (4)
|                                              /-----------+
|                                              |           \----------- kkz (17)
|                                              |
|                                              |      /---------------- chp (7)
|                                              |      |
|                                          /---+------+---------------- ttmN (20)
|                                          |   |      |
|                                          |   |      \---------------- tceS (21)
|                                          |   |
|                                          |   \----------------------- haa (9)
|------------------------------------------+
|                                          |                  /-------- taa (14)
|                                          |             /----+
|                                          |             |    \-------- tcb (18)
|                                          \-------------+
|                                                        \------------- tau (19)
|
|                                /------------------------------------- gce (24)
|                                |
\--------------------------------+------------------------------------- tol (25)
                                 |
                                 \------------------------------------- cco (26)

------------|------------------|------------------|------------------|
          0.15               0.10               0.05               0.00
```

[Expected changes per site]

Calculating tree probabilities...

Credible sets of trees (3001 trees sampled):
   50 % credible set contains 1501 trees
   90 % credible set contains 2701 trees
   95 % credible set contains 2851 trees
   99 % credible set contains 2971 trees

```
                             MrBayes v3.2.1 x64

                      (Bayesian Analysis of Phylogeny)

              Distributed under the GNU General Public License


               Type "help" or "help <command>" for information
                     on the commands that are available.

                   Type "about" for authorship and general
                       information about the program.


MrBayes > exe /Users/msicoli/Yeniseian-NaDene-Typ_noHAXNoEYA.nex

   Executing file "/Users/msicoli/Yeniseian-NaDene-Typ_noHAXNoEYA.nex"
   UNIX line termination
   Longest line length = 123
   Parsing file
   Expecting NEXUS formatted file
   Reading data block
      Allocated taxon set
      Allocated matrix
      Defining new matrix with 38 taxa and 116 characters
      Data is Standard
      Missing data coded as ?
      Gaps coded as -
      Data matrix is not interleaved
      Taxon  1 -> gwi
      Taxon  2 -> dgr
      Taxon  3 -> scsh
      Taxon  4 -> xsl
      Taxon  5 -> bea
      Taxon  6 -> crx
      Taxon  7 -> chp
      Taxon  8 -> txc
      Taxon  9 -> haa
      Taxon 10 -> ing
      Taxon 11 -> kuu
      Taxon 12 -> hoi
      Taxon 13 -> koy
      Taxon 14 -> taa
      Taxon 15 -> aht
      Taxon 16 -> tfn
      Taxon 17 -> kkz
      Taxon 18 -> tcb
      Taxon 19 -> tau
      Taxon 20 -> ttmN
      Taxon 21 -> tceS
      Taxon 22 -> tli
      Taxon 23 -> gce
      Taxon 24 -> tol
      Taxon 25 -> cco
      Taxon 26 -> hup
      Taxon 27 -> mtl
      Taxon 28 -> wlk
      Taxon 29 -> kto
      Taxon 30 -> apc
      Taxon 31 -> apw
      Taxon 32 -> apj
      Taxon 33 -> nav
      Taxon 34 -> apk
      Taxon 35 -> apl
```

```
      Taxon 36 -> srs
      Taxon 37 -> ket
      Taxon 38 -> zko
      Successfully read matrix
      Setting default partition (does not divide up characters)
      Setting model defaults
      Seed (for generating default start values) = 1388169385
      Adding dummy characters (unobserved site patterns) for division 1
      WARNING: There are 32 characters incompatible with the specified
               coding bias. These characters will be excluded.
      Setting output file names to "/Users/msicoli/Yeniseian-NaDene-Typ_noHAXNoEYA.nex.run<i>.<p|t>"
   Exiting data block
   Reached end of file

MrBayes > lset nst=6 rates=gamma

   Setting Rates to Gamma
   Successfully set likelihood model parameters
   Adding dummy characters (unobserved site patterns) for division 1
   WARNING: There are 32 characters incompatible with the specified
            coding bias. These characters will be excluded.

MrBayes > prset brlenspr=clock:uniform

   Setting Brlenspr to Clock:Uniform
   Successfully set prior model parameters
   Adding dummy characters (unobserved site patterns) for division 1
   WARNING: There are 32 characters incompatible with the specified
            coding bias. These characters will be excluded.

MrBayes > mcmcp ngen=2000000 printfreq=10000 samplefreq=500 nruns=1 nchains=4 savebrlens=yes
filename=DY-27Dec-strict-H-EOut

   Setting number of generations to 2000000
   Setting print frequency to 10000
   Setting sample frequency to 500
   Setting number of runs to 1
   Setting number of chains to 4
   Setting chain output file names to "DY-27Dec-strict-H-EOut.<p/t>"
   Successfully set chain parameters

MrBayes > mcmc

   Running Markov chain
   MCMC stamp = 3445309595
   Seed = 583554439
   Swapseed = 1388169385
   Model settings:

      Data not partitioned --
         Datatype  = Standard
         Coding    = Variable
         # States  = Variable, up to 10
                     State frequencies are fixed to be equal
         Rates     = Gamma
                     Gamma shape parameter is uniformly dist-
                     ributed on the interval (0.00,200.00).
                     Gamma distribution is approximated using 4 categories.
                     Likelihood summarized over all rate categories in each generation.

   Active parameters:

      Parameters
      ------------------
      Statefreq        1
```

```
      Shape            2
      Ratemultiplier   3
      Topology         4
      Brlens           5
      Clockrate        6
      ------------------

      1 --  Parameter  = Alpha_symdir
            Type       = Symmetric diricihlet/beta distribution alpha_i parameter
            Prior      = Symmetric dirichlet with fixed(-1.00) variance parameter

      2 --  Parameter  = Alpha
            Type       = Shape of scaled gamma distribution of site rates
            Prior      = Uniform(0.00,200.00)

      3 --  Parameter  = Ratemultiplier
            Type       = Partition-specific rate multiplier
            Prior      = Fixed(1.0)

      4 --  Parameter  = Tau
            Type       = Topology
            Prior      = All topologies equally probable a priori
            Subparam.  = V

      5 --  Parameter  = V
            Type       = Branch lengths
            Prior      = Clock:Uniform
                         Tree age has an Exponential(1.000) distribution
                         Node ages are not constrained

      6 --  Parameter  = Clockrate
            Type       = Base rate of clock
            Prior      = Fixed(1.000000)
                         The clock rate is constant (strict clock)


   Number of taxa = 38
   Number of characters = 116

   The MCMC sampler will use the following moves:
      With prob.  Chain will use move
         2.38 %   Multiplier(Alpha)
        11.90 %   ExtSprClock(Tau,V)
        23.81 %   NNIClock(Tau,V)
        11.90 %   ParsSPRClock(Tau,V)
        47.62 %   NodesliderClock(V)
         2.38 %   TreeStretch(V)

   Division 1 has 77 unique site patterns
   Initializing conditional likelihoods
   Using standard non-SSE likelihood calculator for division 1 (single-precision)

   Initial log likelihoods and log prior probs:
      Chain 1 -- -1529.584402 -- -87.230988
      Chain 2 -- -1536.001745 -- -87.230988
      Chain 3 -- -1493.848213 -- -87.230988
      Chain 4 -- -1568.572229 -- -87.230988


   Chain results (2000000 generations requested):

       0 -- [-1529.584] (-1536.002) (-1493.848) (-1568.572)

[OMITTED GENERATIONS]
```

```
   2000000 -- (-915.505) (-930.645) [-921.682] (-928.871) -- 0:00:00

   Continue with analysis? (yes/no): n

   Analysis completed in 16 mins 36 seconds
   Analysis used 951.77 seconds of CPU time
   Log likelihood of best state for "cold" chain was -893.59

   Acceptance rates for the moves in the "cold" chain:
      With prob.   (last 100)   chain accepted proposals by move
         38.2 %     ( 25 %)     Multiplier(Alpha)
         19.3 %     ( 27 %)     ExtSprClock(Tau,V)
         45.0 %     ( 43 %)     NNIClock(Tau,V)
         15.4 %     ( 17 %)     ParsSPRClock(Tau,V)
         70.4 %     ( 71 %)     NodesliderClock(V)
         71.2 %     ( 26 %)     TreeStretch(V)

   Chain swap information:

                1       2       3       4
        ----------------------------------
      1 |            0.53    0.26    0.11
      2 |  334295            0.60    0.33
      3 |  332184  333798            0.64
      4 |  332815  334284  332624

   Upper diagonal: Proportion of successful state exchanges between chains
   Lower diagonal: Number of attempted state exchanges between chains

   Chain information:

     ID -- Heat
    -----------
      1 -- 1.00  (cold chain)
      2 -- 0.91
      3 -- 0.83
      4 -- 0.77

   Heat = 1 / (1 + T * (ID - 1))
      (where T = 0.10 is the temperature and ID is the chain number)


MrBayes > sumt relburnin=yes

   Using relative burnin (a fraction of samples discarded).
   Summarizing trees in file "DY-27Dec-strict-H-EOut.t"
   Using relative burnin ('relburnin=yes'), discarding the first 25 % of sampled trees
   Writing statistics to files DY-27Dec-strict-H-EOut.<parts|tstat|vstat|trprobs|con>
   Examining file ...
   Found one tree block in file "DY-27Dec-strict-H-EOut.t" with 4001 trees in last block

   Tree reading status:

   0      10      20      30      40      50      60      70      80      90     100
   v-------v-------v-------v-------v-------v-------v-------v-------v-------v-------v
   *********************************************************************************

   Read 4001 trees from last tree block (sampling 3001 of them)

   General explanation:

   In an unrooted tree, a taxon bipartition (split) is specified by removing a
   branch, thereby dividing the species into those to the left and those to the
   right of the branch. Here, taxa to one side of the removed branch are denoted
   '.' and those to the other side are denoted '*'. Specifically, the '.' symbol
```

is used for the taxa on the same side as the outgroup.

In a rooted or clock tree, the tree is rooted using the model and not by reference to an outgroup. Each bipartition therefore corresponds to a clade, that is, a group that includes all the descendants of a particular branch in the tree. Taxa that are included in each clade are denoted using '*', and taxa that are not included are denoted using the '.' symbol.

The output first includes a key to all the bipartitions with frequency larger or equual to (Minpartfreq) in at least one run. Minpartfreq is a paramiter to sumt command and currently it is set to 0.10. This is followed by a table with statistics for the informative bipartitions (those including at least two taxa), sorted from highest to lowest probability. For each bipartition, the table gives the number of times the partition or split was observed in all runs (#obs) and the posterior probability of the bipartition (Probab.), which is the same as the split frequency. If several runs are summarized, this is followed by the minimum split frequency (Min(s)), the maximum frequency (Max(s)), and the standard deviation of frequencies (Stddev(s)) across runs. The latter value should approach 0 for all bipartitions as MCMC runs converge.

This is followed by a table summarizing branch lengths, node heights (if a clock model was used) and relaxed clock parameters (if a relaxed clock model was used). The mean, variance, and 95 % credible interval are given for each of these parameters. If several runs are summarized, the potential scale reduction factor (PSRF) is also given; it should approach 1 as runs converge. Node heights will take calibration points into account, if such points were used in the analysis.

Note that Stddev may be unreliable if the partition is not present in all runs (the last column indicates the number of runs that sampled the partition if more than one run is summarized). The PSRF is not calculated at all if the partition is not present in all runs.The PSRF is also sensitive to small sample sizes and it should only be considered a rough guide to convergence since some of the assumptions allowing one to interpret it as a true potential scale reduction factor are violated in MrBayes.

Clade credibility values:

```
/--------------------------------------------------------------------- txc (8)
|
|--------------------------------------------------------------------- kuu (11)
|
|--------------------------------------------------------------------- aht (15)
|
|--------------------------------------------------------------------- tfn (16)
|
|--------------------------------------------------------------------- tli (22)
|
|                                                         /----------- ket (37)
|---------------------------100---------------------------+
|                                                         \----------- zko (38)
|
|                                                         /----------- apc (30)
|                                             /-----61----+
|                                             |           \----------- apw (31)
|                                  /----69----+
|                                  |          \----------------------- apj (32)
|                      /-----84----+
|                      |           \---------------------------------- nav (33)
|           /----73----+
|           |          |                                  /----------- apk (34)
|           |          \----------------100---------------+
|-----92----+                                             \----------- apl (35)
```

```
|          |
|          \-------------------------------------------------------- srs (36)
|
|                                           /----------------------- hup (26)
|                                           |
|                                           |----------------------- mtl (27)
|---------------------84--------------------+
|                                           |           /----------- wlk (28)
|                                           \-----84----+
+                                                       \----------- kto (29)
|
|                                           /----------------------- ing (10)
|                                           |
|---------------------75--------------------+           /----------- hoi (12)
|                                           \-----58----+
|                                                       \----------- koy (13)
|
|                                           /----------------------- gwi (1)
|                                           |
|                                           |----------------------- dgr (2)
|                                           |
|---------------------69--------------------+----------------------- scsh (3)
|                                           |
|                                           |           /----------- bea (5)
|                                           \-----55----+
|                                                       \----------- crx (6)
|
|                                                       /----------- gce (23)
|                                                       |
|--------------------------59---------------------------+----------- tol (24)
|                                                       |
|                                                       \----------- cco (25)
|
|                                                       /----------- xsl (4)
|                                           /-----62----+
|                                           |           \----------- kkz (17)
|                                           |
|                                           |           /----------- chp (7)
|                                           |           |
|                                /----59----+-----51----+----------- ttmN (20)
|                                |          |           |
|                                |          |           \----------- tceS (21)
|                                |          |
\---------------58---------------+          \----------------------- haa (9)
                                 |
                                 |                      /----------- taa (14)
                                 |                      |
                                 \----------81----------+----------- tcb (18)
                                                        |
                                                        \----------- tau (19)
```

Phylogram (based on median node depths):

```
/------------------------------------------------------------------- txc (8)
|
|------------------------------------------------------------------- kuu (11)
|
|------------------------------------------------------------------- aht (15)
|
|------------------------------------------------------------------- tfn (16)
|
|------------------------------------------------------------------- tli (22)
|
|                                       /--------------------------- ket (37)
```

```
|---------------------------------------+
|                                       \-------------------------- zko (38)
|
|                                                               /-- apc (30)
|                                                            /--+
|                                                            |  \-- apw (31)
|                                                       /----+
|                                                       |    \----- apj (32)
|                                                /------+
|                                                |      \---------- nav (33)
|                                        /-------+
|                                        |       |               /- apk (34)
|                                        |       \---------------+
|----------------------------------------+                       \- apl (35)
|                                        |
|                                        \------------------------- srs (36)
|
|                            /------------------------------------- hup (26)
|                            |
|                            |------------------------------------- mtl (27)
|----------------------------+
|                            |                     /--------------- wlk (28)
|                            \---------------------+
+                                                  \--------------- kto (29)
|
|                                               /------------------ ing (10)
|                                               |
|-----------------------------------------------+      /----------- hoi (12)
|                                               \------+
|                                                      \----------- koy (13)
|
|                                        /------------------------- gwi (1)
|                                        |
|                                        |------------------------- dgr (2)
|                                        |
|----------------------------------------+------------------------- scsh (3)
|                                        |
|                                        |              /---------- bea (5)
|                                        \--------------+
|                                                       \---------- crx (6)
|
|                               /---------------------------------- gce (23)
|                               |
|-------------------------------+---------------------------------- tol (24)
|                               |
|                               \---------------------------------- cco (25)
|
|                                                      /----------- xsl (4)
|                                            /---------+
|                                            |         \----------- kkz (17)
|                                            |
|                                            |     /--------------- chp (7)
|                                            |     |
|                                        /---+-----+--------------- ttmN (20)
|                                        |   |     |
|                                        |   |     \--------------- tceS (21)
|                                        |   |
\----------------------------------------+   \--------------------- haa (9)
                                         |
                                         |           /------------- taa (14)
                                         |           |
                                         \-----------+------------- tcb (18)
                                                     |
                                                     \------------- tau (19)
```

```
   -----------|------------------|-------------------|------------------|
            0.15               0.10                0.05               0.00

   [Expected changes per site]

   Calculating tree probabilities...

   Credible sets of trees (3001 trees sampled):
      50 % credible set contains 1501 trees
      90 % credible set contains 2701 trees
      95 % credible set contains 2851 trees
      99 % credible set contains 2971 trees


MrBayes > sump relburnin=yes

   Using relative burnin (a fraction of samples discarded).
   Summarizing parameters in file DY-27Dec-strict-H-EOut.p
   Writing summary statistics to file DY-27Dec-strict-H-EOut.pstat
   Using relative burnin ('relburnin=yes'), discarding the first 25 % of samples

   Below is a rough plot of the generation (x-axis) versus the log
   probability of observing the data (y-axis). You can use this
   graph to determine what the burn in for your analysis should be.
   When the log probability starts to plateau you may be at station-
   arity. Sample trees and parameters after the log probability
   plateaus. Of course, this is not a guarantee that you are at sta-
   analysis should be. When the log probability starts to plateau
   tionarity. When possible, run multiple analyses starting from dif-
   ferent random trees; if the inferences you make for independent
   analyses are the same, this is reasonable evidence that the chains
   have converged. You can use MrBayes to run several independent
   analyses simultaneously. During such a run, MrBayes will monitor
   the convergence of topologies. After the run has been completed,
   the 'sumt' and 'sump' functions will provide additional conver-
   gence diagnostics for all the parameters in your model. Remember
   that the burn in is the number of samples to discard. There are
   a total of ngen / samplefreq samples taken during a MCMC analysis.

   +------------------------------------------------------------+ -913.099
   |        *                                   * *     * **    |
   |                                    *                       |
   |  *  *            *           *           *                 |
   |                                                            |
   |*             *     *          *  *            **           |
   |             *   *    *               *      *   **         |
   |   *  ** * *       *   *                *          * *    * |
   |               **         ***    * *                    **  |
   | *                              *        * *               *|
   |                     *  **           *                      |
   |    *     *                            *                    |
   |            *                                               |
   |                                                            |
   |                                                            |
   |                             *                              |
   +------+-----+-----+-----+-----+-----+-----+-----+-----+-----+ -919.893
   ^                                                            ^
   500000                                                      2000000


   Estimated marginal likelihoods for run sampled in file "DY-27Dec-strict-H-EOut.p":
      (Use the harmonic mean for Bayes factor comparisons of models)
      (Values are saved to the file /Users/msicoli/Yeniseian-NaDene-Typ_noHAXNoEYA.nex.lstat)

   Arithmetic mean   Harmonic mean
```

-904.06 -935.45

Model parameter summaries for run sampled in file "DY-27Dec-strict-H-EOut":
Based on a total of 3001 samples out of a total of 4001 samples
from this analysis.
Parameter summaries saved to file "/Users/msicoli/Yeniseian-NaDene-Typ_noHAXNoEYA.nex.pstat".

Parameter	Mean	Variance	95% HPD Interval Lower	95% HPD Interval Upper	Median	ESS*
TH	0.175288	0.001509	0.086786	0.243660	0.176903	435.06
TL	2.788269	0.305848	1.474125	3.694101	2.854921	399.02
alpha	4.785785	305.551597	0.162164	9.106779	1.576508	2663.77

* Convergence diagnostic (ESS = Estimated Sample Size); ESS value
below 100 may indicate that the parameter is undersampled.
